# Melatonin Analogues Potently Inhibit MAO-B and Protect PC12 Cells against Oxidative Stress

**DOI:** 10.3390/antiox10101604

**Published:** 2021-10-12

**Authors:** Ahmed Elkamhawy, Jiyu Woo, Noha A. Gouda, Jushin Kim, Hossam Nada, Eun Joo Roh, Ki Duk Park, Jungsook Cho, Kyeong Lee

**Affiliations:** 1 College of Pharmacy, Dongguk University-Seoul, Goyang 10326, Korea; a_elkamhawy@mans.edu.eg (A.E.); wju2757@dgu.ac.kr (J.W.); noha.gooda@dongguk.edu (N.A.G.); hossam_hammouda@dgu.ac.kr (H.N.); 2 Department of Pharmaceutical Organic Chemistry, Faculty of Pharmacy, Mansoura University, Mansoura 35516, Egypt; 3 Convergence Research Center for Diagnosis, Treatment and Care System of Dementia, Korea Institute of Science and Technology (KIST), Seoul 02792, Korea; kimjs@kist.re.kr; 4 Department of Biotechnology, College of Life Science and Biotechnology, Yonsei University, Seoul 03722, Korea; 5 Pharmaceutical Chemistry Department, Faculty of Pharmacy, Badr University, Cairo 11829, Egypt; 6 Chemical Kinomics Research Center, Korea Institute of Science and Technology (KIST), Seoul 02792, Korea; r8636@kist.re.kr; 7 Division of Bio-Medical Science & Technology, KIST School, Korea University of Science and Technology, Seoul 02792, Korea

**Keywords:** bioactive molecules, oxidative stress, neurodegeneration, brain health, Parkinson’s disease, MAO-B, melatonin, neuroprotection, in silico docking simulation, PC12 cells

## Abstract

Monoamine oxidase B (MAO-B) metabolizes dopamine and plays an important role in oxidative stress by altering the redox state of neuronal and glial cells. MAO-B inhibitors are a promising therapeutical approach for Parkinson’s disease (PD). Herein, 24 melatonin analogues (**3a**–**x**) were synthesized as novel MAO-B inhibitors with the potential to counteract oxidative stress in neuronal PC12 cells. Structure elucidation, characterization, and purity of the synthesized compounds were performed using ^1^H-NMR, ^13^C-NMR, HRMS, and HPLC. At 10 µM, 12 compounds showed >50% MAO-B inhibition. Among them, compounds **3n**, **3r**, and **3u**–**w** showed >70% inhibition of MAO-B and IC_50_ values of 1.41, 0.91, 1.20, 0.66, and 2.41 µM, respectively. When compared with the modest selectivity index of rasagiline (**II**, a well-known MAO-B inhibitor, SI > 50), compounds **3n, 3r, 3u**, and **3v** demonstrated better selectivity indices (SI > 71, 109, 83, and 151, respectively). Furthermore, compounds **3n** and **3r** exhibited safe neurotoxicity profiles in PC12 cells and reversed 6-OHDA- and rotenone-induced neuronal oxidative stress. Both compounds significantly up-regulated the expression of the anti-oxidant enzyme, heme oxygenase (HO)-1. Treatment with Zn(II)-protoporphyrin IX (ZnPP), a selective HO-1 inhibitor, abolished the neuroprotective effects of the tested compounds, suggesting a critical role of HO-1 up-regulation. Both compounds increased the nuclear translocation of Nrf2, which is a key regulator of the antioxidative response. Taken together, these data show that compounds **3n** and **3r** could be further exploited for their multi-targeted role in oxidative stress-related PD therapy.

## 1. Introduction

Parkinson’s disease (PD) is the second most common age-related neurodegenerative disorder after Alzheimer’s disease (AD). It is characterized by tremor, bradykinesia, and muscle rigidity along with impaired gait and posture. The gradual degeneration and death of dopaminergic neurons in the substantia nigra pars compacta (SNpc) followed by dopamine depletion in the striatum are linked to the motor impairments that define PD. PD was first described over 200 years ago; however, it remains an incurable disorder because the mechanisms underlying neuronal degeneration are complicated and not fully understood. Nonetheless, several studies have indicated that oxidative stress significantly contributes to the degeneration of dopaminergic neurons leading to PD [1,2,3,4,5,6,7,8,9,10].

**Figure 1:**
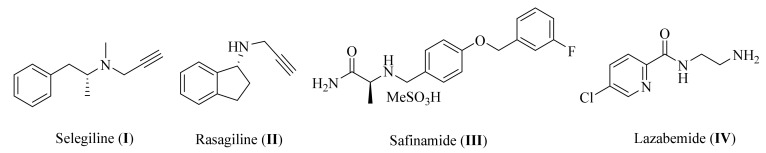
Chemical structure of some well-known MAO-B inhibitors.

Several sources and mechanisms for reactive oxygen species (ROS) generation are recognized including the metabolism of dopamine. Under normal conditions, dopamine levels are regulated via monoamine oxidase A (MAO-A), which is predominantly found in catecholaminergic neurons. However, when neuronal degeneration occurs in PD, monoamine oxidase B (MAO-B) increases in glial cells and becomes the primary enzyme to metabolize dopamine [11,12,13,14,15,16]. The degradation of dopamine by MAO-B, combined with ground state O_2_, leads to the generation of reactive oxygen species (ROS), and subsequently to oxidative stress. The products of this include 3,4-dihydroxyphenylacetaldehyde, an ammonium molecule, and hydrogen peroxide (H_2_O_2_), which reacts with the iron ion (Fe^+2^) in dopaminergic neurons to form hydroxyl radicals. This leads to oxidative stress and neuronal cell death [1,17,18]. Additionally, MAO-B is associated with the mitochondrial dysfunction caused by 1-methyl-4-phenyl-1,2,3,6-tetrahydropyridine (MPTP)-induced PD. MPTP is metabolized by MAO-B into 1-methyl-4-phenylpyridinuim (MPP^+^), which acts as a substrate for the dopamine transporter. It is selectively taken up into dopaminergic neurons, where it inhibits Complex I of the mitochondrial electron transport chain [19,20,21], leading to mitochondrial dysfunction. Accordingly, the inhibition of MAO-B is a promising strategy for relieving the symptoms of PD [22,23,24]. This is verified by the fact that several clinically prescribed substances improve the emerging motor symptoms of parkinsonism, including levodopa, dopamine agonists, and MAO-B inhibitors. Nevertheless, due to their favorable safety profile and putative neuroprotective capabilities, MAO-B inhibitors constitute a preferable therapeutic option for early PD. Furthermore, MAO-B inhibitors regulate the dopamine metabolism and reduce the generation of neurotoxic DA metabolites such as ROS and dopamine-derived aldehydes [17,24,25]. As illustrated in Figure 1, various potent small molecules such as selegiline (**I**), rasagiline (**II**), safinamide (**III**), and lazabemide (**IV**) can effectively block MAO-B [26,27,28]. Selegiline is FDA-approved as an adjunct treatment in the management of patients with PD and as a treatment for a major depressive disorder (MDD) in adults [29]. Rasagiline is a second-generation potent selective MAO-B inhibitor [30]. Safinamide is an orally active, selective, reversible MAO-B inhibitor with both dopaminergic and non-dopaminergic (glutamatergic) properties [31]. Lazabemide is a relatively short-acting, reversible, and selective MAO-B inhibitor [32]. Further studies have indicated that selegiline and rasagiline prevent MPTP toxicity in the nigral dopaminergic neurons via the inhibition of MPP+ production [33,34]. Therefore, MAO-B inhibitors are commonly prescribed for PD. However, many adverse effects of these inhibitors have been noticed in the long-term therapy of PD, including the formation of neurotoxic metabolites that lead to oxidative stress, sodium channel blockage, hallucinations, headaches, calcium channel modulation, and the suppression of triggered glutamate release [35,36,37]. As a result, the development of new multifunctional neuroprotective agents to inhibit MAO-B and protect neuronal cells against oxidative stress remains vital [38,39].

**Figure 2:**
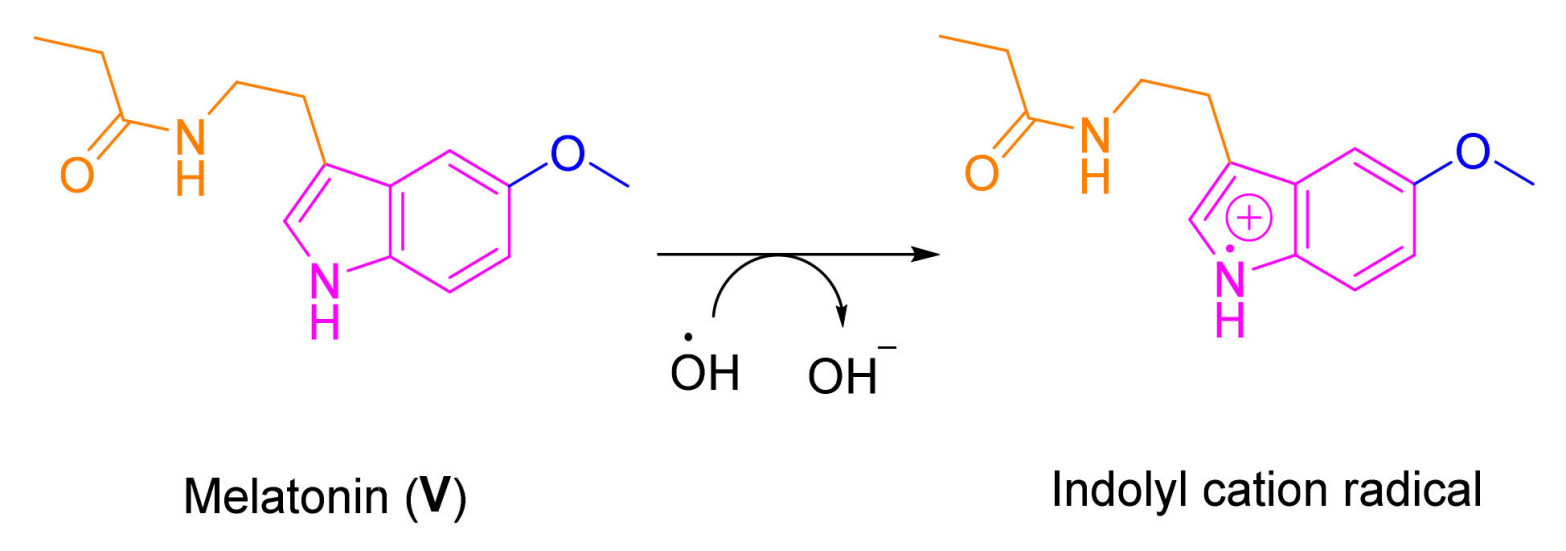
The electron-rich aromatic indole ring system of melatonin scavenges the hydroxyl radical (HO^•^).

Melatonin (**V**), which is a ubiquitous molecule present in almost every live being from bacteria to humans, has remarkable antioxidant activity [40,41]. It scavenges oxygen free radicals, such as super oxide radical, hydroxyl radical, peroxyl radical, and peroxynitrite anion. Melatonin plays a crucial role in the genesis of the neurodegenerative diseases due to a significant decline in its production with increasing age [42,43,44]. In addition to its antioxidant and neuroprotective effects [45,46,47,48,49,50], melatonin is anti-inflammatory [51], antibacterial [52,53], antitumor [54], pain modulatory [55], and has cardiovascular [56] and liver injury protective properties [57], modulating chronic kidney diseases [58], etc. [59,60,61]. Accordingly, different research groups have reported several synthetic derivatives of melatonin as potential modulators to different human disorders including neurodegenerative diseases [62,63,64,65,66,67,68,69]. Several studies have indicated that the potent ability of melatonin to scavenge ROS including ^1^O_2_, O_2_^•−^, H_2_O_2_, hydroxyl radical (HO^•^), and ROO^•^, is mainly due to the presence of the electron-rich aromatic indole ring system (Figure 2). This allows indoleamine to easily function as an electron donor, forming the indolyl cation [50,70,71,72].

**Figure 3:**
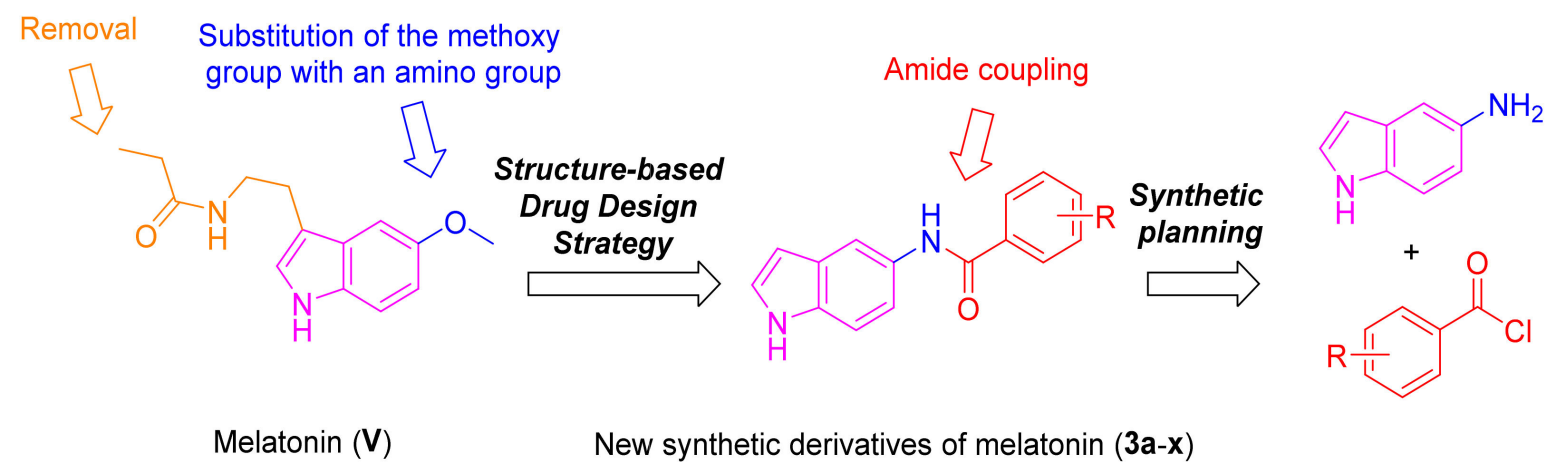
Design of structural modifications performed on melatonin and the synthetic planning to offer derivatives **3a**–**x**.

Recently, we have reported an indole-based small molecule (*N*-(1-(3-fluorobenzoyl)-1*H*-indol-5-yl)pyrazine-2-carboxamide) with selective and competitive inhibitory effect over MAO-B with an IC_50_ value of 0.78 µM and *K*i of 94.52 nM [73]. Inspired by the promising MAO-B activity of the indole-based compounds, structural modifications on the chemical structure of melatonin were performed to develop melatonin-derived analogues to possess potent MAO-B inhibition and anti-oxidative stress effects. This is a promising hypothesis to develop melatonin-derived neuroprotective agents for PD. As depicted in Figure 3, the methoxy group of melatonin was replaced with an amino group, followed by amide formation via reaction with different benzoyl chloride derivatives to yield the target derivatives of melatonin (**3a**–**x**). The choice of these substituted moieties was inspired by their presence in neuroprotectant small molecules that possess effects against MAO-B and/or oxidative stress [74,75,76].

## 2. Materials and Methods

### 2.1. Chemical Reagents, Purification, and Instrumentation

The general protocol used for the chemical synthesis, elucidation of the chemical structures, and purity of the synthesized melatonin derivatives are detailed in Supplementary Materials, as reported earlier [77,78,79,80].

### 2.2. Synthesis of Melatonin Analogues **3a**–**x**

To a dry round-bottom flask containing 5-aminoindole (1, 0.2 g, 1.5 mmol) and triethylamine (TEA, 0.31 mL, 1.5 mmol) dissolved in 9 mL of dichloromethane (DCM), the appropriate benzoyl chloride (2) was added (1.5 mmol). The reaction mixture was stirred for 2 h at room temperature. The excess solvent was evaporated, and the residue was partitioned between ethyl acetate (EA) and water with the aid of brine. The organic solution was evaporated, dried over magnesium sulfate anhydrous, and further purified using flash column chromatography (SiO_2_, hexane:EA = 9:1) to yield the target compounds.

#### 2.2.1. *N*-(1*H*-indol-5-yl)benzamide (**3a**)

Dark brown solid. mp: 165–166 °C. HPLC purity: 10 min, 99.65%. IR (KBr, ν cm^−1^): 3200–3400 (NHs), 1519 (CO). ^1^H NMR (400 MHz, DMSO-*d_6_*) *δ* 11.04 (s, 1H), 10.08 (s, 1H), 7.97 (d, *J* = 8.00 Hz, 3H), 7.57–7.50 (m, 3H), 7.40–7.33 (m, 3H), 6.41 (s, 1H). ^13^C NMR (100 MHz, DMSO-*d*_6_) *δ* 165.54, 135.88, 133.42, 131.60, 131.36, 128.74, 127.97, 127.82, 126.33, 116.54, 112.65, 111.45, 101.59. HRMS (ESI) *m/z* calculated for C_15_H_12_N_2_O [M + H]^+^: 237.1028, found: 237.1017. Reported [81,82].

#### 2.2.2. 2-Bromo-*N*-(1*H*-indol-5-yl)benzamide (**3b**)

Black solid. mp: 231–233 °C. HPLC purity: 11 min, 95.11%. ^1^H NMR (400 MHz, DMSO-*d_6_*) *δ* 11.03 (s, 1H), 10.24 (s, 1H), 8.00 (s, 1H), 7.70 (d, *J* = 8.00 Hz, 1H), 7.55 (dd, *J* = 7.5, 1.7 Hz, 1H), 7.48 (t, *J* = 7.3 Hz, 1H), 7.39 (td, *J* = 7.6, 1.6 Hz, 1H), 7.33 (dd, *J* = 6.3, 3.4 Hz, 3H), 6.41 (s, 1H). ^13^C NMR (100 MHz, DMSO-*d*_6_) *δ* 165.79, 140.13, 133.40, 133.09, 131.36, 131.29, 129.28, 128.09, 127.84, 126.43, 119.58, 115.56, 111.66, 111.57, 101.60. HRMS (ESI) *m/z* calculated for C_15_H_11_BrN_2_O [M + H]^+^: 315.0133, found: 315.0120.

#### 2.2.3. 4-Bromo-*N*-(1*H*-indol-5-yl)benzamide (**3c**)

Black solid. mp: 209–210 °C. HPLC purity: 14 min, 99.60%. ^1^H NMR (400 MHz, DMSO-*d_6_*) *δ* 11.09 (s, 1H), 10.19 (s, 1H), 8.00–7.89 (m, 3H), 7.74 (d, *J* = 7.60 Hz, 2H), 7.35 (d, *J* = 14.70 Hz, 3H), 6.41 (s, 1H). ^13^C NMR (100 MHz, DMSO-*d*_6_) *δ* 164.50, 134.88, 133.48, 131.71, 131.14, 130.18, 127.78, 126.36, 125.32, 116.50, 112.71, 111.48, 101.56. HRMS (ESI) *m/z* calculated for C_15_H_11_BrN_2_O [M + H]^+^: 315.0133, found: 315.0122.

#### 2.2.4. 2,4-Dichloro-*N*-(1*H*-indol-5-yl)benzamide (**3d**)

Black solid. mp: 161–162 °C. HPLC purity: 14 min, 99.78%. ^1^H NMR (400 MHz, DMSO-*d_6_*) *δ* 11.06 (s, 1H), 10.32 (s, 1H), 7.98 (s, 1H), 7.76 (s, 1H), 7.62 (d, *J* = 8.20 Hz, 1H), 7.55 (d, *J* = 8.10 Hz, 1H), 7.35–7.28 (m, 3H), 6.41 (s, 1H). ^13^C NMR (100 MHz, DMSO-*d*_6_) *δ* 163.91, 136.78, 134.90, 133.44, 131.71, 131.13, 130.75, 129.54, 127.82, 126.50, 115.46, 111.64, 101.61. HRMS (ESI) *m/z* calculated for C_15_H_10_Cl_2_N_2_O [M + H]^+^: 305.0248, found: 305.0235.

#### 2.2.5. *N*-(1*H*-indol-5-yl)-3,5-dinitrobenzamide (**3e**)

Yellow solid. mp: above 300 °C. HPLC purity: 14 min, 99.08%. ^1^H NMR (400 MHz, DMSO-*d_6_*) *δ* 11.13 (s, 1H), 10.75 (s, 1H), 9.20 (s, 2H), 9.00 (s, 1H), 8.02 (s, 1H), 7.41 (s, 2H), 7.37 (s, 1H), 6.45 (s, 1H). ^13^C NMR (100 MHz, DMSO-*d*_6_) *δ* 161.15, 148.50, 138.29, 133.76, 130.46, 128.29, 127.81, 126.62, 121.16, 116.40, 113.00, 111.64, 101.70. HRMS (ESI) *m/z* calculated for C_15_H_10_N_4_O_5_ [M + H]^+^: 327.0729, found: 327.0714.

#### 2.2.6. *N*-(1*H*-indol-5-yl)-3-nitrobenzamide (**3f**)

Black solid. mp: 214–215 °C. HPLC purity: 12 min, 99.57%. IR (KBr, ν cm^−1^): 3300–3400 (NHs), 1648 (CO). ^1^H NMR (400 MHz, DMSO-*d_6_*) *δ* 11.09 (s, 1H), 10.45 (s, 1H), 8.81 (s, 1H), 8.43 (d, *J* = 8.00 Hz, 2H), 8.00 (s, 1H), 7.83 (t, *J* = 8.00 Hz, 1H), 7.39 (s, 2H), 7.35 (s, 1H), 6.43 (s, 1H). ^13^C NMR (100 MHz, DMSO-*d*_6_) *δ* 163.27, 148.18, 137.20, 134.48, 133.62, 130.85, 130.53, 127.82, 126.49, 126.26, 122.75, 116.49, 112.88, 111.57, 101.66. HRMS (ESI) *m/z* calculated for C_15_H_11_NO_3_ [M + H]^+^: 282.0879, found: 282.0865. Reported [83].

#### 2.2.7. *N*-(1*H*-indol-5-yl)-4-nitrobenzamide (**3g**)

Brown solid. mp: 262–264 °C. HPLC purity: 12 min, 99.96%. IR (KBr, ν cm^−1^): 3300–3500 (NHs), 1590 (CO). ^1^H NMR (400 MHz, DMSO-*d*_6_) *δ* 11.09 (s, 1H), 10.42 (s, 1H), 8.37 (d, *J* = 8.00 Hz, 2H), 8.20 (d, *J* = 12.00 Hz, 2H), 8.01 (s, 1H), 7.39 (s, 2H), 7.35 (s, 1H), 6.43 (s, 1H). ^13^C NMR (100 MHz, DMSO-*d*_6_) *δ* 163.79, 149.37, 141.59, 133.59, 130.91, 129.50, 127.82, 126.50, 123.93, 116.38, 112.74, 111.58, 101.67. HRMS (ESI) *m/z* calculated for C_15_H_11_N_3_O_3_ [M + H]^+^: 282.0879, found: 282.0868.

#### 2.2.8. 2,6-Difluoro-*N*-(1*H*-indol-5-yl)benzamide (**3h**)

Pink solid. mp: 236–237 °C. HPLC purity: 10 min, 100%. ^1^H NMR (400 MHz, DMSO-*d_6_*) *δ* 11.07 (s, 1H), 10.55 (s, 1H), 7.98 (s, 1H), 7.63–7.48 (m, 1H), 7.35 (t, *J* = 6.60 Hz, 2H), 7.31–7.17 (m, 3H), 6.42 (s, 1H). ^13^C NMR (100 MHz, DMSO-*d*_6_) *δ* 160.55, 157.92, 133.49, 132.08, 130.96, 127.85, 126.60, 115.15, 112.55, 112.30, 111.76, 111.41, 101.67. HRMS (ESI) *m/z* calculated for C_15_H_10_F_2_N_2_O [M + H]^+^: 273.0839, found: 273.0829.

#### 2.2.9. 3-Fluoro-*N*-(1*H*-indol-5-yl)benzamide (**3i**)

Brown solid. mp: 166–168 °C. HPLC purity: 11 min, 99.94%. IR (KBr, ν cm^−1^): 3200–3250 (NHs), 1502 (CO). ^1^H NMR (400 MHz, DMSO-*d_6_*) *δ* 11.06 (s, 1H), 10.15 (s, 1H), 7.98 (s, 1H), 7.84–7.77 (m, 2H), 7.61–7.56 (m, 1H), 7.45–7.33 (m, 4H), 6.41 (s, 1H). ^13^C NMR (100 MHz, DMSO-*d*_6_) *δ* 163.59, 162.62, 138.18, 133.52, 131.05, 130.91, 127.81, 126.41, 124.16, 118.50, 116.51, 114.78, 112.76, 111.51, 101.62. HRMS (ESI) *m/z* calculated for C_15_H_11_FN_2_O [M + H]^+^: 255.0934, found: 255.0929.

#### 2.2.10. 4-Fluoro-*N*-(1*H*-indol-5-yl)benzamide (**3j**)

Brown solid. mp: 208–209 °C. HPLC purity: 11 min, 99.27%. IR (KBr, ν cm^−1^): 3210–3250 (NHs), 1498 (CO). ^1^H NMR (400 MHz, DMSO-*d_6_*) *δ* 11.04 (s, 1H), 10.10 (s, 1H), 8.07–8.03 (m, 2H), 7.96 (s, 1H), 7.38–7.33 (m, 5H), 6.41 (s, 1H). ^13^C NMR (100 MHz, DMSO-*d*_6_) *δ* 164.41, 164.28, 133.44, 132.25, 131.22, 130.63, 127.81, 126.35, 116.54, 115.65, 112.71, 111.46, 101.59. HRMS (ESI) *m/z* calculated for C_15_H_11_FN_2_O [M + H]^+^: 255.0934, found: 255.0940. Reported [84].

#### 2.2.11. 2-Fluoro-*N*-(1*H*-indol-5-yl)benzamide (**3k**)

White solid. mp: 179–181 °C. HPLC purity: 11 min, 99.98%. ^1^H NMR (400 MHz, DMSO-*d_6_*) *δ* 11.04 (s, 1H), 10.19 (s, 1H), 7.99 (s, 1H), 7.66 (t, *J* = 8.00 Hz, 1H), 7.59–7.53 (m, 1H), 7.36–7.31 (m, 4H), 6.41 (s, 1H). ^13^C NMR (100 MHz, DMSO-*d*_6_) *δ* 162.67, 159.29, 133.39, 132.52, 131.28, 130.30, 127.83, 126.44, 125.99, 124.90, 116.52, 1115.64, 111.73, 111.60, 101.63. HRMS (ESI) *m/z* calculated for C_15_H_11_FN_2_O [M + H]^+^: 255.0934, found: 255.0923.

#### 2.2.12. 4-Fluoro-*N*-(1*H*-indol-5-yl)-2-(trifluoromethyl)benzamide (**3l**)

White solid. mp: 153–154 °C. HPLC purity: 13 min, 98.80%. ^1^H NMR (400 MHz, DMSO-*d_6_*) *δ* 11.05 (s, 1H), 10.34 (s, 1H), 7.94 (s, 1H), 7.78 (d, *J* = 6.90 Hz, 2H), 7.68 (d, *J* = 7.90 Hz, 1H), 7.35 (d, *J* = 9.30 Hz, 2H), 7.27 (d, *J* = 9.70 Hz, 1H), 6.41 (s, 1H). ^13^C NMR (100 MHz, DMSO-*d*_6_) *δ* 164.59, 163.22, 160.90, 133.89, 133.45, 131.85, 131.77, 131.14, 127.82, 126.49, 119.98, 115.55, 114.53, 111.78, 111.60, 101.60. HRMS (ESI) *m/z* calculated for C_16_H_10_F_4_N_2_O [M + H]^+^: 323.0808, found: 323.0795.

#### 2.2.13. 3-Cyano-*N*-(1*H*-indol-5-yl)benzamide (**3m**)

Brown solid. mp: 188–189 °C. HPLC purity: 10 min, 97.06%. IR (KBr, ν cm^−1^): 3250–3400 (NHs), 1650 (CO). ^1^H NMR (400 MHz, DMSO-*d_6_*) *δ* 11.08 (s, 1H), 10.27 (s, 1H), 8.42 (s, 1H), 8.27 (d, *J* = 7.90 Hz, 1H), 8.05 (d, *J* = 7.80 Hz, 1H), 8.00 (s, 1H), 7.75 (t, *J* = 7.80 Hz, 1H), 7.38 (s, 2H), 7.34 (t, *J* = 2.7 Hz, 1H), 6.42 (s, 1H). ^13^C NMR (100 MHz, DMSO-*d*_6_) *δ* 163.57, 136.82, 135.05, 133.58, 132.84, 131.60, 130.97, 130.17, 127.84, 126.47, 118.87, 116.37, 112.69, 111.90, 111.59, 101.67. HRMS (ESI) *m/z* calculated for C_16_H_11_N_3_O [M + H]^+^: 262.0980, found: 262.0974.

#### 2.2.14. 3-Chloro-*N*-(1*H*-indol-5-yl)benzamide (**3n**)

Brown solid. mp: 190–191 °C. HPLC purity: 13 min, 98.79%. ^1^H NMR (400 MHz, DMSO-*d_6_*) *δ* 11.06 (s, 1H), 10.20 (s, 1H), 7.97 (dd, *J* = 26.20, 11.90 Hz, 3H), 7.65 (d, *J* = 8.90 Hz, 1H), 7.56 (t, *J* = 7.80 Hz, 1H), 7.34 (dd, *J* = 9.10, 6.20 Hz, 3H), 6.42 (s, 1H). ^13^C NMR (100 MHz, DMSO-*d*_6_) *δ* 164.02, 137.83, 133.61, 133.54, 131.44, 131.08, 130.73, 127.83, 127.77, 126.78, 126.41, 116.49, 112.76, 111.52, 101.64. HRMS (ESI) *m/z* calculated for C_15_H_11_ClN_2_O [M + H]^+^: 271.0638, found: 271.0627. Reported [82].

#### 2.2.15. 2-Chloro-*N*-(1*H*-indol-5-yl)benzamide (**3o**)

Brown solid. mp: 226–227 °C. HPLC purity: 11 min, 96.81%. IR (KBr, ν cm^−1^): 3200–3400 (NHs), 1510 (CO). ^1^H NMR (400 MHz, DMSO-*d_6_*) *δ* 11.05 (s, 1H), 10.28 (s, 1H), 8.00 (s, 1H), 7.56 (t, *J* = 8.00 Hz, 2H), 7.51–7.43 (m, 2H), 7.34 (s, 3H), 6.41 (s, 1H). ^13^C NMR (100 MHz, DMSO-*d*_6_) *δ* 164.89, 137.99, 133.39, 131.37, 131.19, 130.44, 130.02, 129.35, 127.85, 127.62, 126.44, 115.51, 111.61, 101.62. HRMS (ESI) *m/z* calculated for C_15_H_11_ClN_2_O [M + H]^+^: 271.0638, found: 271.0627.

#### 2.2.16. 4-Chloro-*N*-(1*H*-indol-5-yl)benzamide (**3p**)

Brown solid. mp: 220–222 °C. HPLC purity: 13 min, 97.74%. IR (KBr, ν cm^−1^): 3280–3310 (NHs), 1550 (CO). ^1^H NMR (400 MHz, DMSO-*d_6_*) *δ* 11.05 (s, 1H), 10.16 (s, 1H), 8.05–7.94 (m, 3H), 7.60 (d, *J* = 8.50 Hz, 2H), 7.38–7.31 (m, 3H), 6.41 (s, 1H). ^13^C NMR (100 MHz, DMSO-*d*_6_) *δ* 164.42, 136.44, 134.56, 133.51, 131.16, 129.94, 128.80, 127.84, 126.38, 116.54, 112.77, 111.51, 101.63. HRMS (ESI) *m/z* calculated for C_15_H_11_ClN_2_O [M + H]^+^: 271.0638, found: 271.0625.

#### 2.2.17. 4-(Tert-butyl)-*N*-(1*H*-indol-5-yl)benzamide (**3q**)

Gray solid. mp: 189–190 °C. HPLC purity: 17 min, 100.00%. ^1^H NMR (400 MHz, DMSO-*d_6_*) *δ* 11.02 (s, 1H), 10.00 (s, 1H), 7.97 (s, 1H), 7.90 (d, *J* = 8.30 Hz, 2H), 7.53 (d, *J* = 8.30 Hz, 2H), 7.39–7.35 (m, 2H), 7.33–7.31 (m, 1H), 6.40 (s, 1H), 1.33 (s, 9H). ^13^C NMR (100 MHz, DMSO-*d*_6_) *δ* 165.46, 154.36, 133.34, 133.19, 131.45, 127.80, 126.28, 125.49, 116.47, 112.51, 111.40, 101.55, 35.07, 31.41. HRMS (ESI) *m/z* calculated for C_19_H_20_N_2_O [M + H]^+^: 293.1654, found: 293.1642.

#### 2.2.18. 3-Bromo-*N*-(1*H*-indol-5-yl)benzamide (**3r**)

White solid. mp: 188–189 °C. HPLC purity: 14 min, 99.80%. ^1^H NMR (400 MHz, DMSO-*d_6_*) *δ* 11.06 (s, 1H), 10.20 (s, 1H), 8.16 (s, 1H), 7.98 (d, *J* = 7.00 Hz, 2H), 7.78 (d, *J* = 8.00 Hz, 1H), 7.50 (t, *J* = 7.90 Hz, 1H), 7.41–7.33 (m, 3H), 6.42 (s, 1H). ^13^C NMR (100 MHz, DMSO-*d*_6_) *δ* 163.89, 138.01, 134.33, 133.51, 131.05, 131.00, 130.57, 127.80, 127.14, 126.40, 122.08, 116.44, 112.70, 111.49, 101.61. HRMS (ESI) *m/z* calculated for C_15_H_13_N_3_O_3_S [M + H]^+^: 315.0533, found: 315.0126.

#### 2.2.19. *N*-(1*H*-indol-5-yl)-3-(trifluoromethoxy)benzamide (**3s**)

Purple solid. mp: 130–132 °C. HPLC purity: 15 min, 100.00%. ^1^H NMR (400 MHz, DMSO-*d_6_*) *δ* 11.07 (s, 1H), 10.24 (s, 1H), 8.03 (d, *J* = 7.90 Hz, 1H), 7.97 (s, 1H), 7.93 (s, 1H), 7.68 (t, *J* = 7.90 Hz, 1H), 7.60 (d, *J* = 8.00 Hz, 1H), 7.37 (s, 1H), 7.35–7.32 (m, 1H), 6.42 (s, 1H). ^13^C NMR (100 MHz, DMSO-*d*_6_) *δ* 163.77, 138.00, 133.57, 130.96, 130.92, 127.81, 127.09, 126.44, 124.13, 120.50, 116.54, 112.87, 111.51, 101.62. HRMS (ESI) *m/z* calculated for C_15_H_13_N_3_O_3_S [M + H]^+^: 321.0851, found: 321.0839. Reported [85].

#### 2.2.20. *N*-(1*H*-indol-5-yl)-4-(trifluoromethoxy)benzamide (**3t**)

White solid. mp: 195–196 °C. HPLC purity: 15 min, 99.65%. ^1^H NMR (400 MHz, DMSO-*d_6_*) *δ* 11.05 (s, 1H), 10.19 (s, 1H), 8.09 (d, *J* = 8.00 Hz, 2H), 7.98 (s, 1H), 7.52 (d, *J* = 8.00 Hz, 2H), 7.37–7.33 (m, 3H), 6.42 (s, 1H). ^13^C NMR (100 MHz, DMSO-*d*_6_) *δ* 164.31, 150.62, 135.03, 133.50, 131.14, 130.32, 127.82, 126.39, 121.72, 121.08, 119.16, 116.45, 112.68, 111.50, 101.61. HRMS (ESI) *m/z* calculated for C_16_H_11_F_3_N_2_O_2_ [M + H]^+^: 321.0851, found: 321.0840.

#### 2.2.21. *N*-(1*H*-indol-5-yl)-3-(trifluoromethyl)benzamide (**3u**)

White solid. mp: 148–150 °C. HPLC purity: 15 min, 99.74%. ^1^H NMR (400 MHz, DMSO-*d_6_*) *δ* 11.07 (s, 1H), 10.32 (s, 1H), 8.29 (d, *J* = 14.20 Hz, 2H), 7.96 (d, *J* = 15.70 Hz, 2H), 7.78 (s, 1H), 7.36 (d, *J* = 15.60 Hz, 2H), 6.43 (s, 1H). ^13^C NMR (100 MHz, DMSO-*d*_6_) *δ* 163.41, 136.13, 133.03, 131.58, 130.41, 129.53, 129.16, 128.84, 127.67, 127.28, 125.91, 124.03, 115.98, 112.31, 110.99, 101.08. HRMS (ESI) *m/z* calculated for C_16_H_11_F_3_N_2_O_2_ [M + H]^+^: 305.0902, found: 305.0890. Reported [85].

#### 2.2.22. *N*-(1*H*-indol-5-yl)-4-(trifluoromethyl)benzamide (**3v**)

Yellow solid. mp: 206–207 °C. HPLC purity: 15 min, 99.98%. ^1^H NMR (400 MHz, DMSO-*d_6_*) *δ* 11.03 (s, 1H), 10.27 (s, 1H), 8.13 (d, *J* = 8.00 Hz, 2H), 7.97 (s, 1H), 7.87 (d, *J* = 8.00 Hz, 2H), 7.37–7.30 (m, 3H), 6.39 (s, 1H). ^13^C NMR (100 MHz, DMSO-*d*_6_) *δ* 164.31, 139.69, 133.55, 131.00, 128.88, 127.80, 126.44, 125.76, 125.72, 125.69, 116.40, 112.70, 111.53, 101.63. HRMS (ESI) *m/z* calculated for C_16_H_11_F_3_N_2_O [M + H]^+^: 305.0902, found: 305.0890.

#### 2.2.23. 3,5-Dichloro-*N*-(1*H*-indol-5-yl)benzamide (**3w**)

Yellow solid. mp: 199–201 °C. HPLC purity: 17 min, 99.96%. ^1^H NMR (400 MHz, DMSO-*d_6_*) *δ* 11.07 (s, 1H), 10.27 (s, 1H), 8.00 (d, *J* = 1.90 Hz, 2H), 7.98 (s, 1H), 7.85 (s, 1H), 7.37 (s, 2H), 7.35–7.33 (m, 1H), 6.42 (s, 1H). ^13^C NMR (100 MHz, DMSO-*d*_6_) *δ* 162.03, 138.46, 134.13, 133.06, 130.45, 130.27, 127.26, 126.28, 125.95, 115.82, 112.20, 111.02, 101.12. HRMS (ESI) *m/z* calculated for C_16_H_11_F_3_N_2_O [M + H]^+^: 305.0248, found: 305.0237.

#### 2.2.24. 2,6-Dichloro-*N*-(1*H*-indol-5-yl)benzamide (**3x**)

White solid. mp: 221–223 °C. HPLC purity: 12 min, 100.00%. ^1^H NMR (400 MHz, DMSO-*d_6_*) *δ* 11.04 (s, 1H), 10.46 (s, 1H), 7.96 (s, 1H), 7.57 (d, *J* = 8.00 Hz, 2H), 7.50–7.46 (m, 1H), 7.37–7.27 (m, 3H), 6.42 (s, 1H). ^13^C NMR (100 MHz, DMSO-*d*_6_) *δ* 161.97, 137.29, 133.52, 131.76, 131.50, 130.92, 128.62, 127.88, 126.57, 115.41, 111.72, 111.68, 101.61. HRMS (ESI) *m/z* calculated for C_15_H_10_C_l2_N_2_O [M + H]^+^: 305.0248, found: 305.0237.

### 2.3. Monoamine Oxidase (MAO) Enzyme Assay

Biological evaluation of the synthesized melatonin derivatives over both MAO-A and MAO-B was performed as reported previously [73]. A detailed description of the protocol is in the Supplementary Materials.

### 2.4. Molecular Modeling

The crystal structures of MAO-A (PDB: 2Z5X) [86] and MAO-B (PDB: 2V5Z) [87] were obtained from the protein data bank. The downloaded MAO-B structure was complexed with the selective inhibitor safinamide. The MAO-A structure was complexed with harmine (7-methoxy-1-methyl-9h-beta-carboline). Protein structures were prepared utilizing the protein preparation wizard [29] of Schrodinger 2021 Suite (Schrödinger LLC, New York, NY, USA) using the default setting and 7.4 pH. The tested compounds were sketched employing ChemDraw Professional 16.0, saved in the structure data file format, and then imported into the Ligprep module. This was used to prepare all ligands and for further geometry optimization. Re-docking X-ray ligands confirmed the reproducibility of the docking program (data not shown). All minimized ligands conformations were docked into the binding site using Glide’s standard precision module [30]. Ten poses were produced for each ligand. The docking figures were visualized using the Discovery Studio Client 2019 package (Dassault Systèmes; BIOVIA. Discovery Studio Modeling Environment; Release 2019; Dassault Systèmes: San Diego, CA, USA, 2019). The docked poses with more negative Glide docking scores were selected for visualization.

### 2.5. In Vitro Cellular and Cell-Free Bio-Assays

#### 2.5.1. Materials

PC12 cells were purchased from the American Type Culture Collection (CRL-1721). RPMI 1640 medium, fetal bovine serum (FBS), horse serum (HS), and antibiotic–antimycotic agents were obtained from Gibco BRL (Grand Island, NY, USA). 6-Hydroxydopamine (6-OHDA) and Zn(II)-protoporphyrin IX (ZnPP) were supplied from Tocris Bioscience (Bristol, UK). Dimethyl sulfoxide (DMSO), laminin, 3-(4,5-dimethyldiazol-2-yl)-2,5-diphenyltetrazolium bromide (MTT), rasagiline mesylate trichloroacetic acid, 2-thiobarbituric acid (TBA), and anti-β-actin antibody (monoclonal, Cat# A5316, 1: 4000, RRID: AB 476743) were obtained from Sigma-Aldrich (St. Louis, MO, USA). Horseradish peroxidase (HRP)-conjugated anti-rabbit immunoglobulin G (IgG, Cat# 7074, 1:2000, RRID: AB 2099233) and anti-mouse IgG (Cat# 7076, 1:2000, RRID: AB 330924) were provided by Cell Signaling Technology (Danvers, MA, USA). Anti-heme oxygenase (HO)-1 antibody (Cat# ADI-SPA-895-J, 1:1000, RRID: AB 11180392) was purchased from Enzo Life Sciences, Inc. (Farmingdale, NY, USA) and anti-nuclear factor erythroid 2-related factor 2 (Nrf2, Cat# 16396-1-AP, 1:1000, RRID: AB 2782956) antibody was from Proteintech Biotechnologies (Pennsylvania, PA, USA). All other chemicals were of analytical grade.

#### 2.5.2. PC12 Cell Culture

PC12 cells were routinely maintained in RPMI-1640 media containing 100 IU/mL penicillin, 100 μg/mL streptomycin supplemented by 10% heat-inactivated HS, and 5% FBS in a humidified incubator containing 5% CO_2_ and 95% air at 37 °C [88]. The cell culture media was changed every two days. Cells were plated on poly-D-lysine/laminin-coated 24-well plates at a density of 3 × 10^5^ cells/well and incubated for at least 24 h to allow cell adhesion.

#### 2.5.3. Drug Treatment

To assess whether the tested compounds exhibited cytotoxicity, PC12 cells were incubated with the tested compounds in low serum media (1% HS, 1% FBS, 100 IU/mL penicillin, and 100 μg/mL streptomycin) at 10 and 30 μM for 24 h. Control cells were incubated in an equivalent volume of DMSO to a final concentration of 0.3%. Neuroprotective effects of the tested compounds on the toxicity induced by 6-OHDA or rotenone in PC12 cells were evaluated as described previously [2,3,4,5,6,7,8] with slight modifications. In brief, cells were pre-treated with the tested compounds or rasagiline at the indicated concentrations for 4 h. Next, they were exposed to 6-OHDA (50 μM) or rotenone (1 μM) for 24 h [89,90,91,92,93,94,95]. In another set of experiments, the influence of HO-1 inhibitor, ZnPP, on the neuroprotective effects of the tested compounds against 6-OHDA-induced oxidative damage was investigated as previously reported [96]. Briefly, the cells were pre-treated with the tested compounds for 4 h and then incubated with 0.2 µM of ZnPP for 30 min. Subsequently, 6-OHDA was added at a final concentration of 50 µM and incubated for 24 h.

#### 2.5.4. Cell Viability Measurements

After the desired treatment, cell viability was determined by MTT assay as described previously [97]. Briefly, MTT was added to the cells at the final concentration of 0.5 mg/mL, which was followed by incubation for 3 h at 37 °C. The supernatants were carefully removed, and then, 300 μL of DMSO was added to each well to dissolve formazan precipitate. The absorbance of the dissolved solution was measured at 550 nm using a microplate reader (SpectraMax M2^e^, Molecular Devices, Sunnyvale, CA, USA). The cell viability was presented as the percentage of the absorbance measured in the vehicle-treated control cells.

#### 2.5.5. Western Blotting

PC12 cells were seeded on 35 mm dishes at a density of 2 × 10^6^ cells/dish and kept at 37 °C in humidified incubator for at least 24 h. Cells were treated with a series of concentrations of the tested compounds for 12 h or with the tested compounds (10 μM) for the indicated time points. Control cells were treated with serum-free media containing 0.3% DMSO instead. Total cell lysates were prepared according to the methods described previously [98], and stored at −20 °C until used. To evaluate the effects of the tested compounds on the nuclear translocation of Nrf2, nuclear fraction was separated from cytosolic fraction using NE-PER Nuclear and Cytoplasmic Extraction Reagents (Rockford, IL, USA), following the manufacturer’s directions. The protein concentrations were assessed using a BioRad DC protein assay kit (BioRad, Hercules, CA, USA). Equal amounts of proteins (20 µg) were resolved on 12% or 8% gels via sodium dodecyl sulfate-polyacrylamide gel electrophoresis and electrophoretically transferred onto nitrocellulose membrane (Whatman, Clifton, NJ, USA). Subsequently, immunoblotting was performed using anti-HO-1 or anti-Nrf2 antibodies, respectively, according to the procedures reported previously [98]. Beta-actin, a highly conserved cytoskeletal protein, was used as a loading control of cell lysates to normalize the level of HO-1, while nuclear protein lamin B was used as a nuclear loading control to normalize the level of Nrf2 in the nuclear fraction. Finally, the immunoreactive bands corresponding to HO-1 or Nrf2 proteins were visualized using Clarity^TM^ Western ECL substrate (Bio-Rad, Hercules, CA, USA) using the BioRad ChemiDoc XRS imaging system (Bio-Rad, Hercules, CA, USA).

#### 2.5.6. Assessment of Lipid Peroxidation (LPO) in Rat Brain Homogenates

The effects of the investigated compounds on the LPO levels in rat forebrain homogenates were evaluated as previously described [99]. Sprague–Dawley (SD) rats were obtained from Daehan Biolink (Chungbuk, Korea) and maintained in the animal facility with controlled temperature (22 ± 2 °C) and relative humidity (40–60%). All the experimental steps and protocols of the animal experiments were approved by the Institutional Animal Ethical Committee of Dongguk University (Approval Number: IACUC-2019-001-2). To initiate LPO reaction, 3–4 mg protein/mL diluent of SD rat forebrain homogenates in 20 mM Tris-HCl buffer (pH 7.4) and several concentrations of the tested compounds dissolved in DMSO were added to a reaction mixture with 10 µM Fe^2+^ and 100 µM L-ascorbic acid and incubated for 60 min at 37 °C. After halting the reaction by adding 28% *w*/*v* trichloroacetic acid and 1% *w*/*v* TBA in succession, the solution was heated for 15 min at 100 °C, which was followed by centrifugation at 3000 rpm for 10 min at 4 °C to separate the precipitates. The supernatant was carefully removed, and its absorbance was determined at 532 nm on a microplate reader (SpectraMax M2^e^, Molecular Devices, Sunnyvale, CA, USA). The percentage of inhibition was calculated by the following formula:

Inhibition (%) = 100 × (1 − Abs_sample_/Abs_control_)

(Abs_control_, the absorbance of control without tested compounds; Abs_sample_, the absorbance of sample with tested compounds).

#### 2.5.7. Statistical Analysis

All data are presented as the mean ± SEM of at least three independent experiments. Statistical significances were analyzed by one-way analysis of variance (ANOVA) followed by Tukey’s post hoc test using SigmaPlot 12.5 software (Systat Software Inc., San Jose, CA, USA). A value of *p* < 0.05 was considered statistically significant.

## 3. Results and Discussion

### 3.1. Chemical Synthesis

**Scheme 1:**
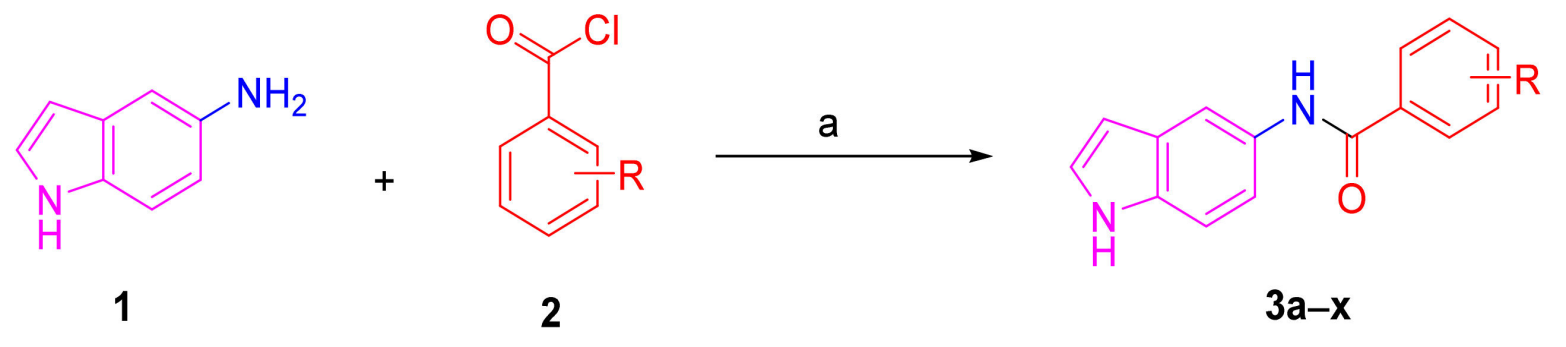
Reagents and conditions: a) appropriate benzoyl chloride, TEA, DCM, rt, 2 h.

A series of twenty-four melatonin analogues **3a**–**x** was synthesized (Scheme 1). Starting from the commercially available 5-aminoindole (**1**), the amide formation reaction was performed using a variety of commercially available benzoyl chlorides (**2**) in the presence of TEA as a catalytic organic base and DCM solvent. Thus, a variety of melatonin analogues bearing various substituents were readily obtained in suitable yields (Table 1).

The chemical structures of final compounds were elucidated using spectroscopic techniques, such as IR, ^1^H NMR, ^13^C NMR, and HRMS. In addition, the purity of all target compounds was detected via HPLC analysis (the purity of all final compounds was ≥95%). The ^1^H NMR spectra of compounds **3a**–**x** were characterized by the presence of two peaks; one of them was at 10.05–10.75 ppm, which is attributable to the indole NH. The second peak was at 11.04–11.20 ppm, which is attributable to the NH of the amide group. The IR spectra of compounds **3a**–**x** showed absorption bands attributed to the NH groups at 3150–3500 cm^−1^ and a carbonyl band at 1500–1700 cm^−1^. Compounds **3e**–**g** exhibited an extra absorption band at 1300–1600 cm^−1^ associated with the N–O stretching of the nitro group. Moreover, ^13^C NMR spectra demonstrated signals resonating at 163.00–166.00 ppm characteristic to the carbonyl group. These findings together with the other spectral data confirmed the formation of the desired melatonin derivatives **3a**–**x**.

### 3.2. In Silico Druggability Studies of the Synthesized Melatonin Analogues **3a**–**x**

A potent antagonistic interaction of an inhibitor with its respective receptor protein or enzyme does not guarantee its capability as a drug candidate. This may be due to poor ADME characteristics and/or unfavorable pharmacokinetic (PK) properties. These challenges may lead to the failure of many generated inhibitors, making the medication development process exceedingly expensive. Consequently, the PK properties of all the synthesized final target compounds (**3a**–**x**) were calculated using SwissADME, which is a freely accessible web server where a machine learning platform is utilized to predict small-molecule PK properties through the use of distance/pharmacophore patterns encoded as graph-based signatures [100]. Several vital properties can be predicted using this server such as the solubility of the target compounds, gastrointestinal absorption, and brain access. These are the main PK keys in the prediction of the results during various stages of the drug discovery processes [101]. An additional significant PK property is the polar surface area (PSA) or topological polar surface area (TPSA) of a molecule. This is characterized as the surface sum over the entire polar atoms or molecules, mainly oxygen and nitrogen, and comprising their accompanying hydrogen atoms. PSA is frequently utilized as a medicinal chemistry metric for enhancing drug capability to permeate the targeted cells. This improves the effectiveness of the synthesized molecule or drug. Molecules possessing a polar surface area >140 Å^2^ are predicted to be incapable of crossing cell membranes. Furthermore, a PSA <90 Å^2^ is believed to be necessary for a molecule to cross the BBB [102]. In addition, compliance of the synthesized compounds with the Lipinski rule of five is usually a good indicator on whether a molecule can be taken orally. The results of the in silico pharmacokinetics study are demonstrated in Table 2.

The predicted PK properties showed that among the 24 synthesized compounds, only compound **3e** was predicted to have poor gastrointestinal absorption and BBB permeability. Similarly, only three compounds (**3f**, **3g**, and **3l**) were predicted to be unable to cross the BBB. Most synthesized compounds were predicted to cross the BBB (TPSA < 90 Å^2^), indicating their capability of being able to exert their intended effect in the brain to combat parkinsonism. Taken together, these results indicate the pharmacokinetic stability of the synthesized series.

### 3.3. MAO Assays

#### 3.3.1. Primary Screening of Melatonin Analogues **3a**–**x** over MAO-B

First, the synthesized melatonin analogues (**3a**–**x**) were biologically evaluated over an MAO-B recombinant enzyme. The experiment was performed using Amplex^®^ Red reagent technology. This technology offers quantitative enzyme activity sensitive detection, a wider dynamic range, and fluorescence emission outside the range of compound autofluorescence (if any). The Amplex^®^ Red reagent is a colorless substrate that reacts with H_2_O_2_ in a 1:1 stoichiometry to produce a highly fluorescent resorufin; therefore, the induced inhibitory effects of the melatonin analogues over MAO-B were examined via spectrophotometry where the fluorescence rate of resorufin dye formation was measured. Compounds **3a**–**x** were initially assessed at a single dose concentration of 10 µM over MAO-B. IC_50_ values over MAO-A and B were further calculated for the most active compounds (Table 3).

Our main goal was to explore the effects of introducing various aromatic functional groups attached via an amide linker to position 5 of the electron-rich indole ring system. Out of 24 compounds, 12 derivatives showed >50% of MAO-B inhibition. Generally, it was noted that meta and para substitutions on the benzamide ring led to an obvious increase in the inhibitory activity against MAO-B, for example in derivatives **3g**, **3j**, **3n**, **3p**, **3q**, **3r**, **3t**, and **3u**–**w**. Among them, analogues **3n, 3r**, and **3u**–**w** incorporating 3-chloro, 3-bromo, 3-trifluoromethyl, 4-trifluoromethyl, and 3,5-dichloro moieties elicited >70% MAO-B inhibition (76.79 ± 0.27, 87.00 ± 0.03, 85.12 ± 0.22, 91.22 ± 0.12, and 72.63 ± 0.32%, respectively). Interestingly, **3u** and **3v**, which have the same substituent (trifluoromethyl) on the meta and para positions of the benzamide ring, respectively, were the most active MAO-B inhibitors in this series. By contrast, when the substitution was changed to the ortho position, the MAO-B inhibitory activity was dramatically decreased as in derivatives **3b, 3h, 3o, and 3x** (0.27 ± 0.81, −0.02 ± 0.26, 2.39 ± 0.31, and 0.89 ± 0.31%, respectively). Similarly, the 3,5-dichloro substitution on the benzamide moiety (**3w**) showed higher activity than 2,6-dichlorobenzamide derivative **3x** (72.63 ± 0.32 and 0.89 ± 0.31%, respectively). Similar outcomes were revealed by the inactive derivatives, **3b** and **3o**, which possess ortho-halo groups when compared with the highly active ones that have meta-halo groups (**3r** and **3n**). Thus, the type and position of substitution on the benzamide moiety are essential factors to exert high MAO-B inhibitory activity. Consequently, the highly active derivatives (**3n**, **3r**, and **3u**–**w**) were further evaluated.

#### 3.3.2. Dose-Dependent Assay of the Most Active Melatonin Analogues over MAO-B

The inhibitory potencies (IC_50_ values) of the melatonin analogues that exhibited MAO-B inhibition >70% at 10 µM were measured using five doses assay with 10, 1, 0.1, 0.01, and 0.001 μM concentrations of the tested compounds. Accordingly, compounds **3n, 3r,** and **3u**–**w** were further tested to determine their IC_50_ values over MAO-B in triplicate from the dose–response inhibition curves using Sigma-Plot software 13.0. Compounds **3n**, **3u**, and **3w** exerted low micromolar IC_50_ values of 1.41, 1.20, and 2.41 µM, respectively (Table 3). A higher potency in the sub-micromolar range was elicited by **3r** and **3v** (0.91 and 0.66 µM, respectively).

#### 3.3.3. Selectivity Assay of Compounds **3r**, **3n**, and **3u**–**w**

Non-selective (MAO-A/B) inhibitors can create various complications when coupled with tyramine-containing nutrients because MAO-A inhibition may lead to a risky boost in serum tyramine expression, which can induce hypertensive symptoms. By contrast, selective MAO-B inhibitors can avoid this issue by preferentially suppressing MAO-B. Therefore, we assessed the selectivity of the most active derivatives for the MAO-B isoform and calculated the selectivity index (SI) as the ratio of IC_50_ (MAO-A)/IC_50_ (MAO-B). The IC_50_ values of compounds **3r**, **3n**, and **3u**–**w** over MAO-A were identified as >100 µM (Table 3). Consequently, the selectivity indices were calculated (Table 3). Compounds **3n**, **3u**, and **3w** showed good selectivity indices (>71, 83, and 41, respectively). Compounds **3r** and **3v** demonstrated higher SI values (>109 and 151, respectively). Thus, compared with the modest selectivity index of rasagiline (**II**, calculated SI > 50), all the tested compounds except **3w** were more selective for MAO-B isoform.

### 3.4. Molecular Modeling

#### 3.4.1. Molecular Docking Study over MAO-B

**Figure 4:**
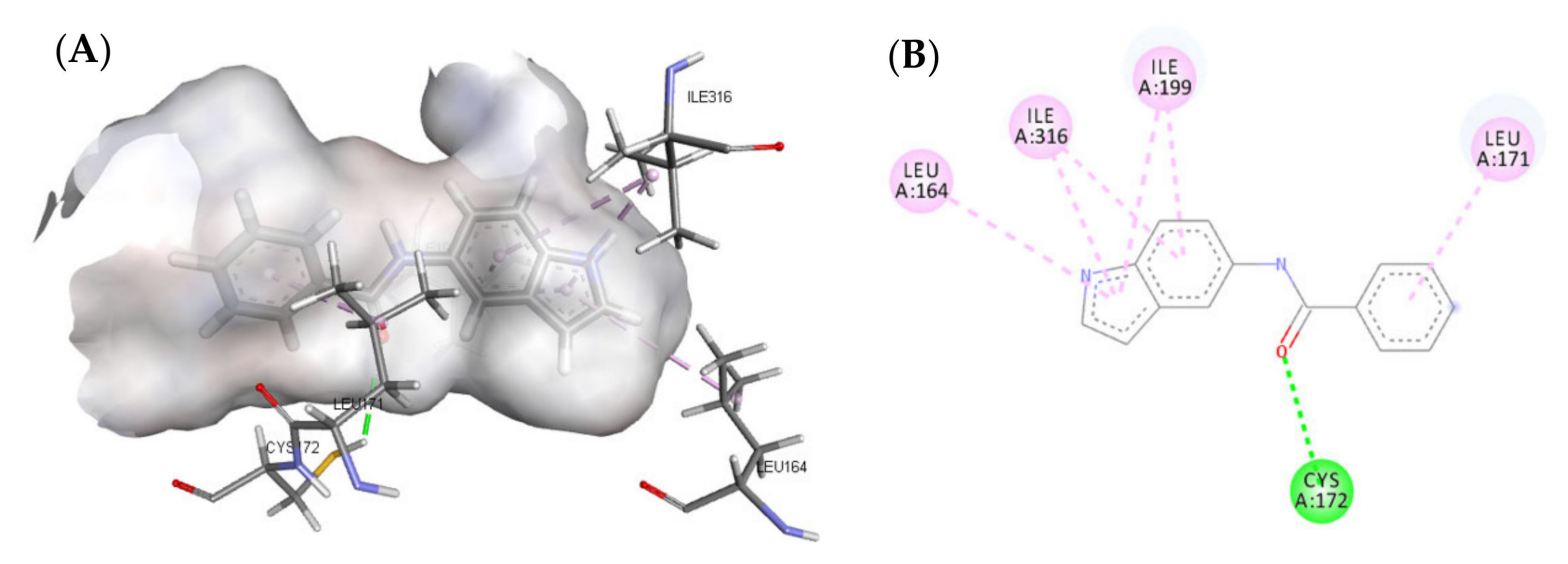
(**A**) Three-dimensional (3D) representation of the interactions of compound **3a** with MAO-B; (**B**) Two-dimensional (2D) interaction model compound **3a** with MAO-B.

A molecular docking study was performed to predict the binding mode(s) of a selection of the synthesized melatonin analogues inside the binding cavity of MAO-B. The absence of any substitutions on the benzamide moiety in compound **3a** resulted in poor MAO-B inhibitory activity (14.86% of inhibition), as highlighted by the docked poses of compound **3a** (Figure 4), where only one hydrogen bond was formed between the carbonyl moiety of the amide group and CYS172. The presence of only one hydrogen bond accompanied by weak hydrophobic interactions resulted in weak binding affinity to the active site residue.

**Figure 5:**
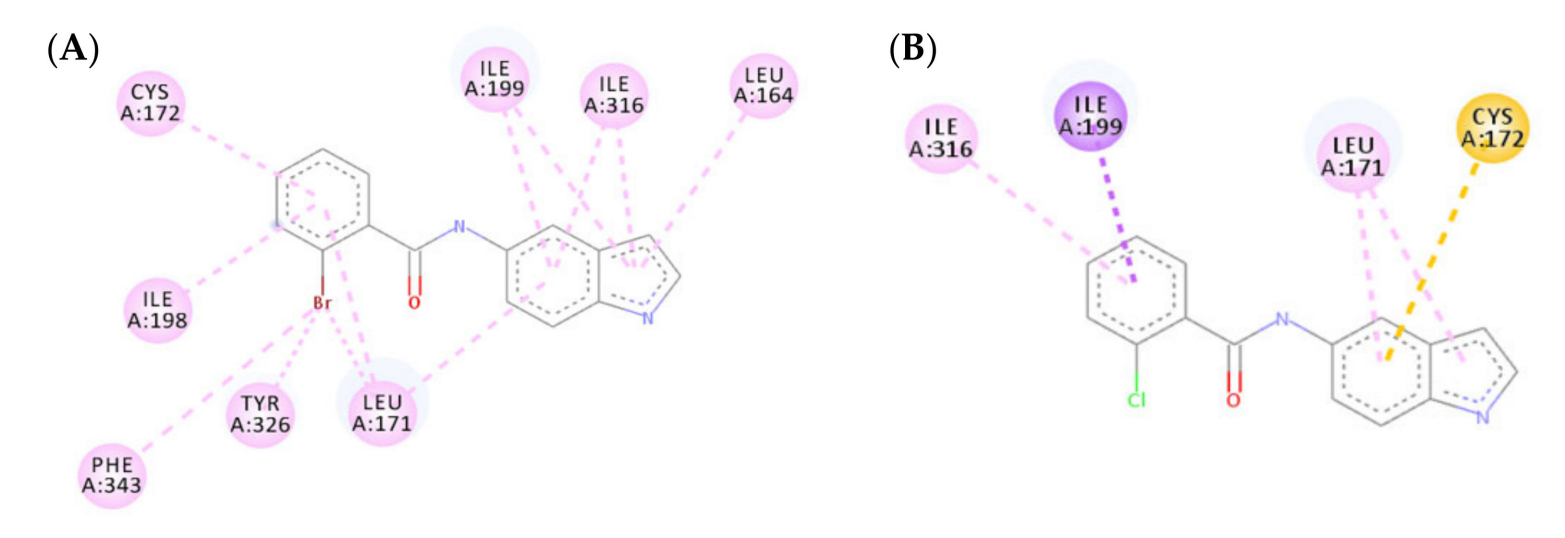
Two-dimensional (2D) interaction models of compounds **3b** (**A**) and **3o** (**B**) with MAO-B.

**Figure 6:**
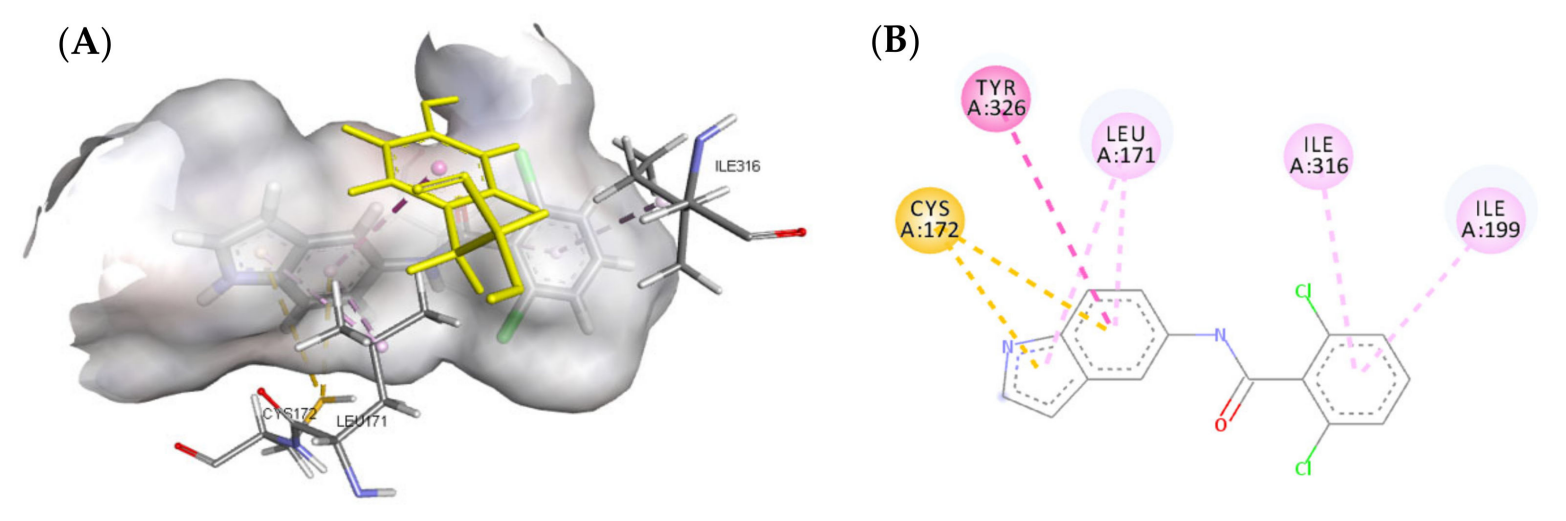
(**A**) Three-dimensional (3D) representation of the interactions of compound **3x** with MAO-B; (**B**) Two-dimensional (2D) interaction model compound **3x** with MAO-B.

The mono-substitution of the benzene ring with a halogen, as in the case of compounds **3b** and **3o**, led to an almost total loss of activity. This is due to the bulky nature of the halogen groups when situated in the ortho position, preventing the formation of the hydrogen bond between the carbonyl moiety of the amide group and CYS172 (Figure 5). This loss in MAO-B inhibitory activity by the ortho-substituted analogues validated the importance of the presence of a hydrogen bond between the carbonyl group and CYS172 for the activity. Similarly, the incorporation of di-halogen substitutions on the benzamide ring in the ortho position resulted in a loss of MAO-B inhibitory activity, which was illustrated by the low number of interactions formed by compound **3x** (Figure 6).

**Figure 7:**
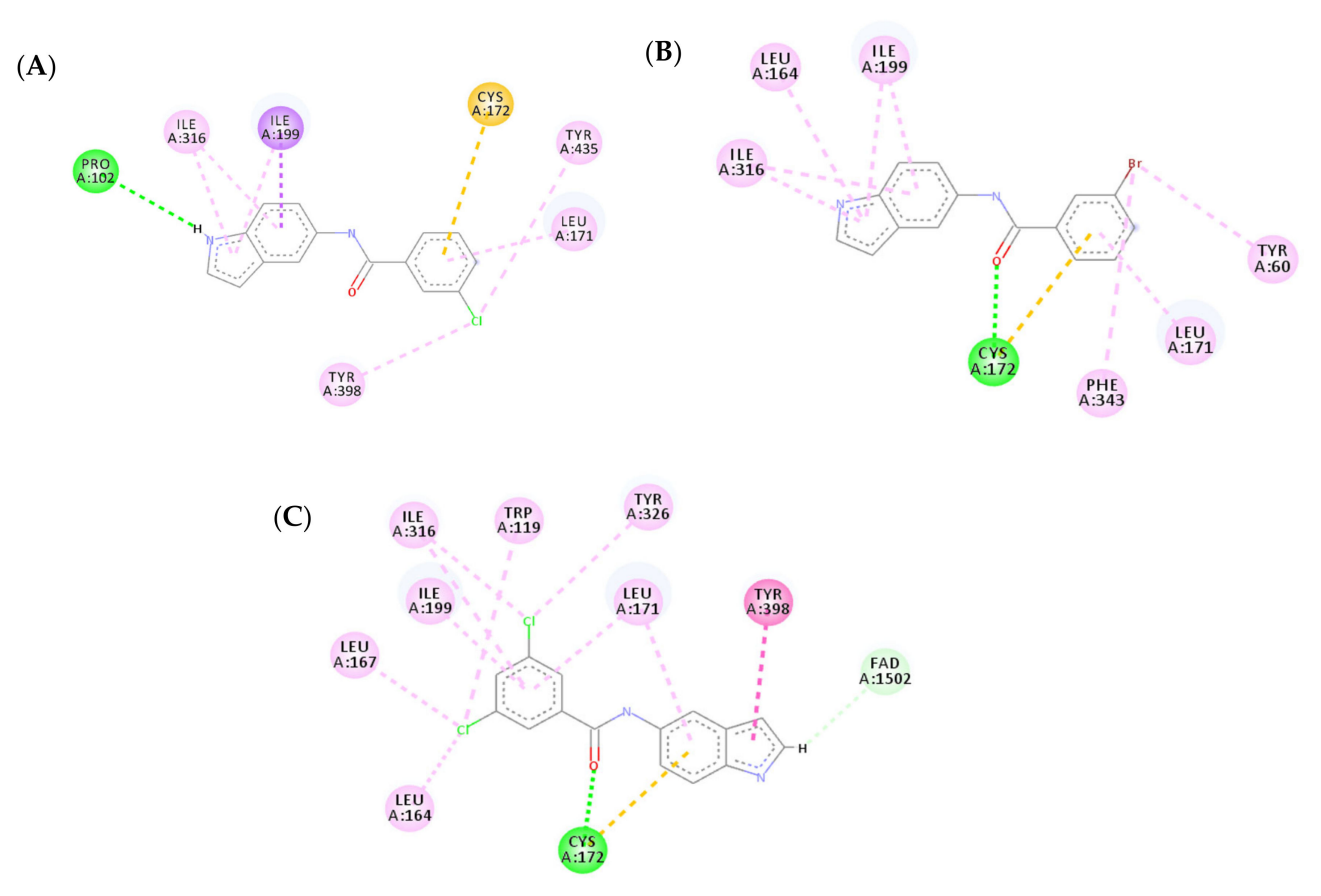
Two-dimensional (2D) interaction models of compounds **3n** (**A**), **3r** (**B**), and **3w** (**C**) with MAO-B.

Altering the substitution on the benzamide ring from the ortho to meta position resulted in a substantial increase in the MAO-B inhibitory activity, which was shown in compounds **3n**, **3r**, and **3w**. Compound **3n** failed to form the essential hydrogen bond between the carbonyl moiety of the amide group and CYS172; however, it formed a strong hydrogen bond between the indole NH and PRO102. This led to a strong binding affinity to the active site residue. Both compounds **3r** and **3w** successfully formed a hydrogen bond between the carbonyl moiety of the amide group and CYS172 as well as forming multiple hydrophobic interactions between the halogen group and the active site residue (Figure 7).

**Figure 8:**
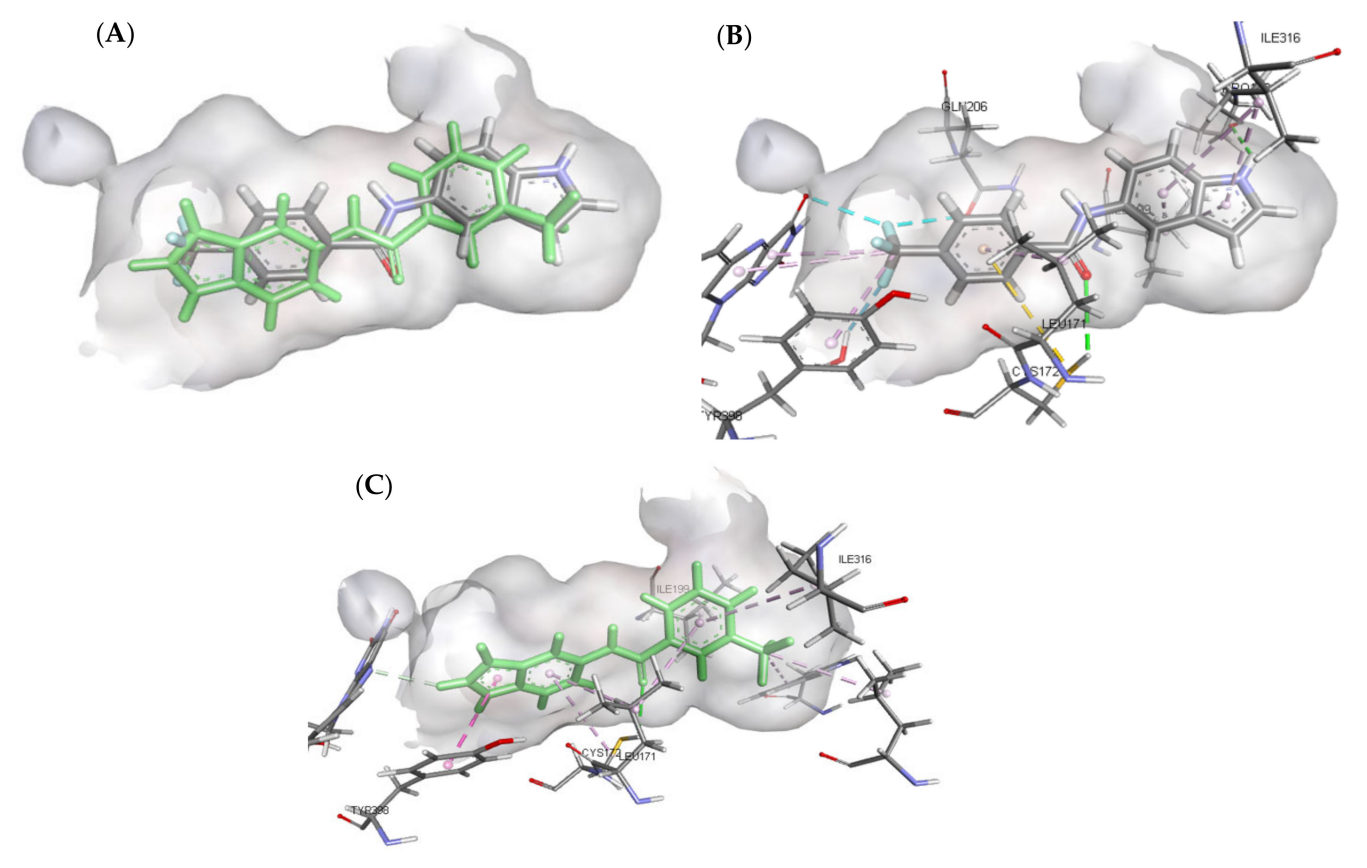
Predicted interactions of compounds **3v** and **3u** into the active site MAO-B. (**A**) Both compounds overlayed in the active site; (**B**) Interactions formed by compound **3v** (gray); (**C**) Interactions formed by compound **3u** (green).

**Figure 9:**
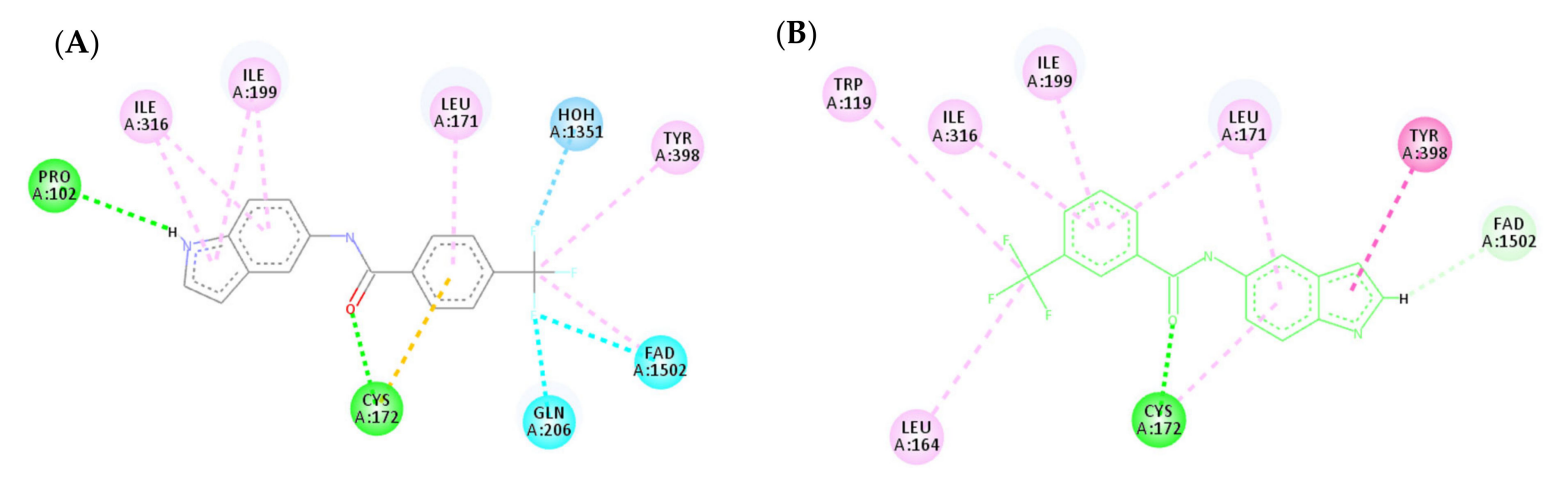
Two-dimensional (2D) interaction models of compounds **3v** (**A**) and **3u** (**B**) into the active site of MAO-B.

The docking models of the most active compounds **3v** and **3u** are depicted in Figure 8. Although both compounds exhibited different orientations in the active site residue, both compounds showed a similar binding affinity to the active site. This strong binding affinity originates from the various interactions that both molecules were able to establish with the active site residue. Compound **3v** established 14 favorable interactions with the active site including three hydrogen bonds; one hydrogen bond was formed between the carbonyl moiety of the amide group and CYS172, a second hydrogen bond was established between the ‘NH’ of the indole ring and PRO102, and the last hydrogen bond was a water-mediated hydrogen bond between one of the fluoro atoms in the trifluoromethyl group and water1351. Additionally, eight favorable π-alkyl interactions were found between compound **3v** and MAO-B active site residue, four of which were formed between the indole ring and ILE316 and ILE199, another two π-alkyl interactions were established by the phenyl ring and LEU171 and CYS172, and the last two π-alkyl interactions were formed between the trifluoromethyl group, TYR398, and FAD. The trifluoromethyl group on the benzamide ring formed another two halogen interactions with FAD and GLN206, making a total number of four favorable interactions. The importance of the trifluoromethyl group is further demonstrated in compound **3u**, which contributed to two π-alkyl interactions with TRP119 and LEU164. The two-dimensional (2D) interaction models of compounds **3v** and **3u** are depicted in Figure 9.

#### 3.4.2. Molecular Docking Study over MAO-A

**Figure 10:**
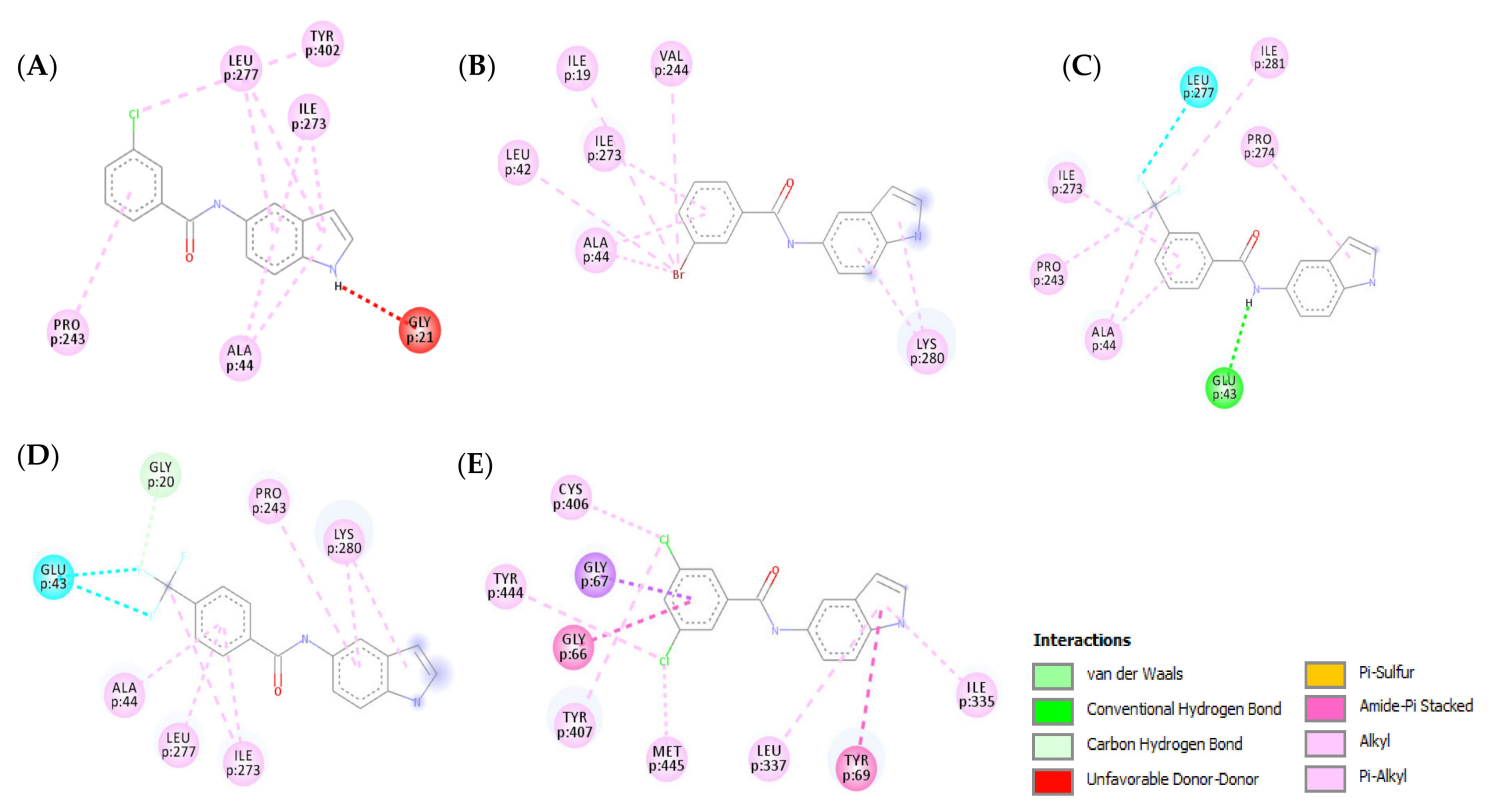
Two-dimensional (2D) interaction models of compounds **3n** (**A**), **3r** (**B**), **3u** (**C**), **3v** (**D**), and **3w** (**E**) into the active site of MAO-A.

A molecular docking study over MAO-A was carried out for compounds **3n**, **3r**, and **3u**–**w** to explain their selectivity. All five compounds showed similar binding modes with MAO-A by exhibiting similar orientation inside the active site. The lowered activity of the five compounds over MAO-A is due to the absence or decrease of hydrogen bonds formed within the binding pocket of MAO-A. Compounds **3n**, **3r,** and **3w** (Figure 10A,B,E, respectively) were unable to establish any hydrogen bonds with the active site of MAO-A, showing a marked decrease in affinity when compared to their MAO-B binding modes. Additionally, compound **3n** formed an unfavorable donor–donor bond with GLY21, further illustrating its low affinity for MAO-A. Compound **3u** exhibited one less hydrogen bond with MAO-A when compared to MAO-B (Figure 10C). Compound **3v** on the other hand only managed to establish one carbon–hydrogen bond between the CF_3_ moiety and GLY20 (Figure 10D). In conclusion, the absence of strong binding interactions between the examined compounds and MAO-A clarifies their weak biological activity.

### 3.5. Biological Evaluation over PC12 Cells

#### 3.5.1. Cytotoxicity Profiles of Compounds **3n**, **3r**, and **3u**–**w** in PC12 Cells

**Figure 11:**
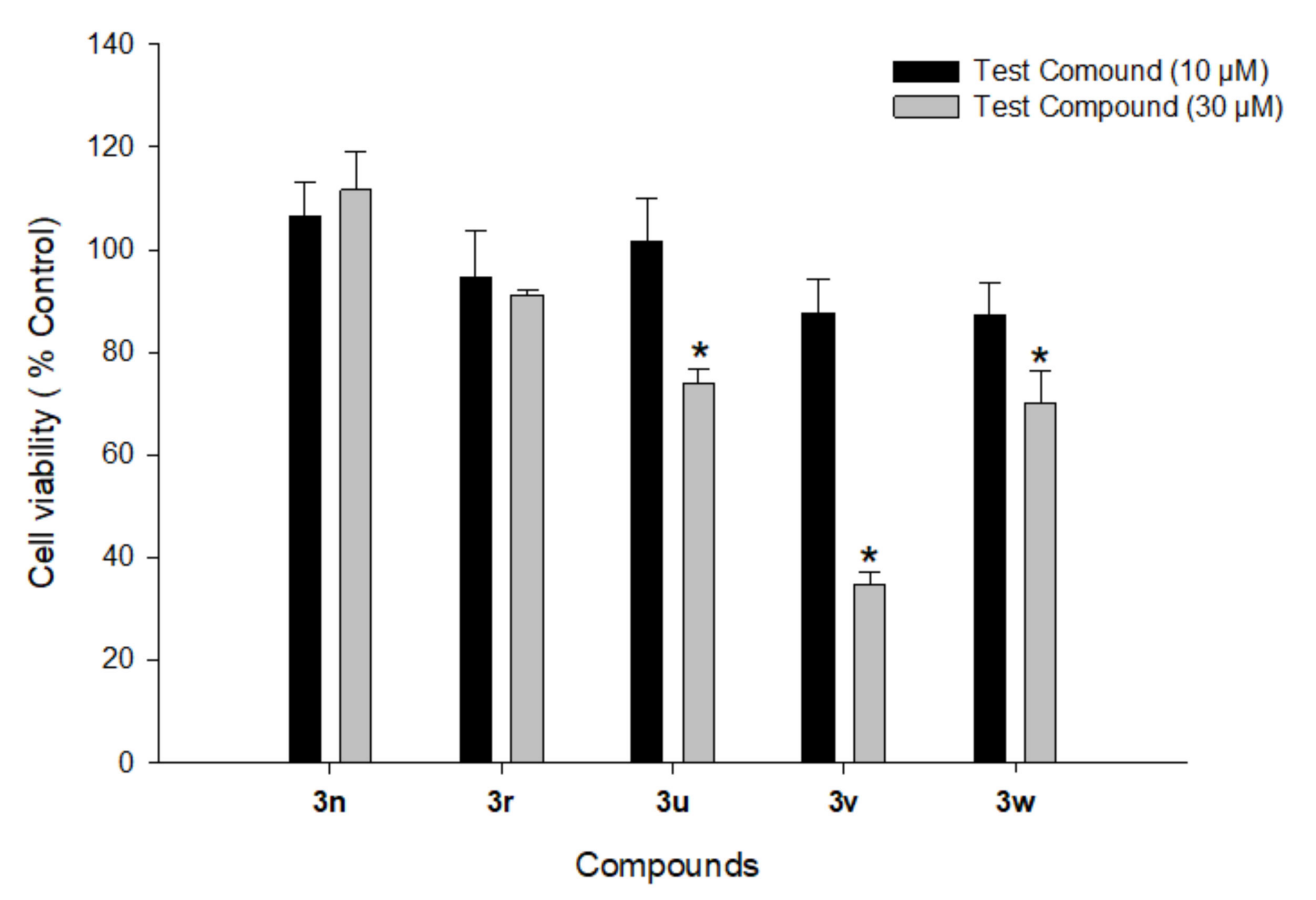
Effects of compounds **3n**, **3r,** and **3u**–**w** on the viability of PC12 cells. PC12 cells were treated with test compounds at 10 and 30 μM for 24 h. MTT assays were performed as described in the Materials and Methods section. Data are expressed as the mean ± SEM of at least three independent experiments. * *p* < 0.05 vs. vehicle-treated control cells.

PD mostly affects dopaminergic neurons in the substantia nigra pars compacta. The primary cultures of central dopaminergic neurons are difficult to acquire and maintain; therefore, we used PC12 cells, a cell line derived from the rat pheochromocytoma, for the biological evaluation of our compounds. PC12 cells are frequently used as an in vitro model in PD research to assess the neuronal toxicities of drug candidates on central dopaminergic neurons and PD-related neurotherapeutic studies [103,104,105]. Before investigating the potential neuroprotective effects of the most active compounds (**3n**, **3r,** and **3u**–**w**), their cytotoxic profiles were assessed in PC12 cells. The cells were treated with each compound at 10 and 30 μM for 24 h. Cell viabilities were measured by MTT assay. Out of five tested compounds, **3n** and **3r** did not show any significant cytotoxic effects on PC12 cells at both tested concentrations (Figure 11). However, the other three compounds (**3u**–**w**) had a significant cytotoxic effect at 30 μM. Accordingly, only compounds **3n** and **3r** were further investigated for their neuroprotective effects against oxidative toxicity induced by 6-OHDA or rotenone in PC12 cells.

#### 3.5.2. Neuroprotective Effects of Compounds **3n** and **3r** on 6-OHDA-Induced Oxidative Toxicity in PC12 Cells

**Figure 12:**
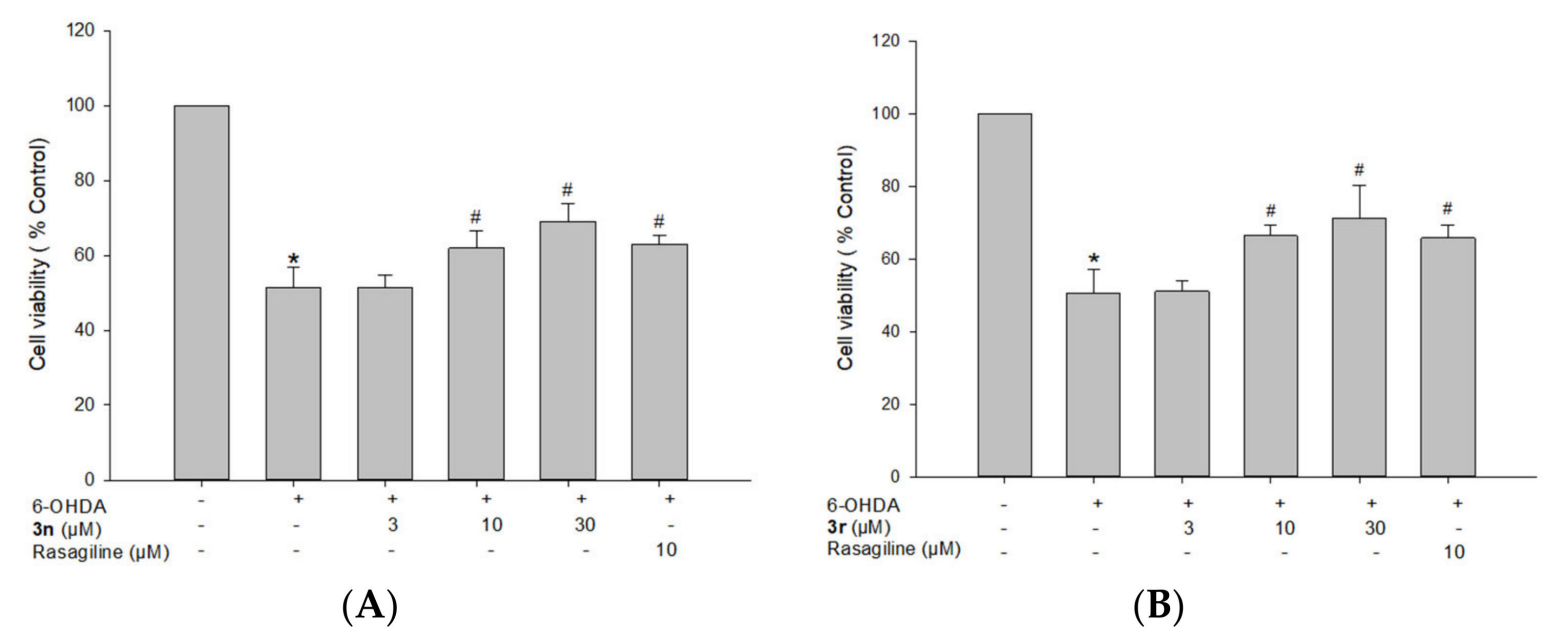
Neuroprotective effects of compounds **3n** (**A**) and **3r** (**B**) on 6-OHDA-induced oxidative toxicity in PC12 cells. PC12 cells were pre-treated with the test compounds or rasagiline at the indicated concentrations for 4 h and exposed to 6-OHDA (50 μM) for an additional 24 h. Cell viability was evaluated by MTT assay as described in the Materials and Methods section. Data are expressed as the mean ± SEM of at least three independent experiments. * *p* < 0.05 and # *p* < 0.05 vs. the vehicle-treated control and 6-OHDA-treated cells, respectively. 6-OHDA, 6-hydroxydopamine.

6-OHDA-induced PC12 cells are widely used as a model of PD to evaluate the neuroprotective potential of compounds in the early stages of preclinical drug development [106,107,108]. 6-OHDA induces dopaminergic neurotoxicity after cellular uptake by producing ROS, which contributes to cell death. Accordingly, the neuroprotective capabilities against 6-OHDA-induced cell damage in PC12 cells were assessed for the two selected compounds **3n** and **3r**. Cells treated with 50 µM 6-OHDA showed reduced cell viability to 50.9 ± 3.1% (Figure 12). However, pre-treatment with compounds **3n** and **3r** markedly reversed this neurotoxicity. Compared with the control, the cell viability increased to 71.3 ± 9.0% and 69.1 ± 4.7% at 30 μM, respectively. Meanwhile, 10 μM of **3n** and **3r** increased cell viability up to 66.4 ± 2.8% and 61.9 ± 4.6%, respectively. Rasagiline, an irreversible MAO-B inhibitor used as a positive reference drug, elevated cell viability to 61.0 ± 1.4% at 10 μM. We further assessed the neuroprotective effects of compounds **3n** and **3r** on rotenone-induced toxicity in PC12 cells to validate our findings.

#### 3.5.3. Neuroprotective Effects of Compounds **3n** and **3r** on Rotenone-Induced Toxicity in PC12 Cells

**Figure 13:**
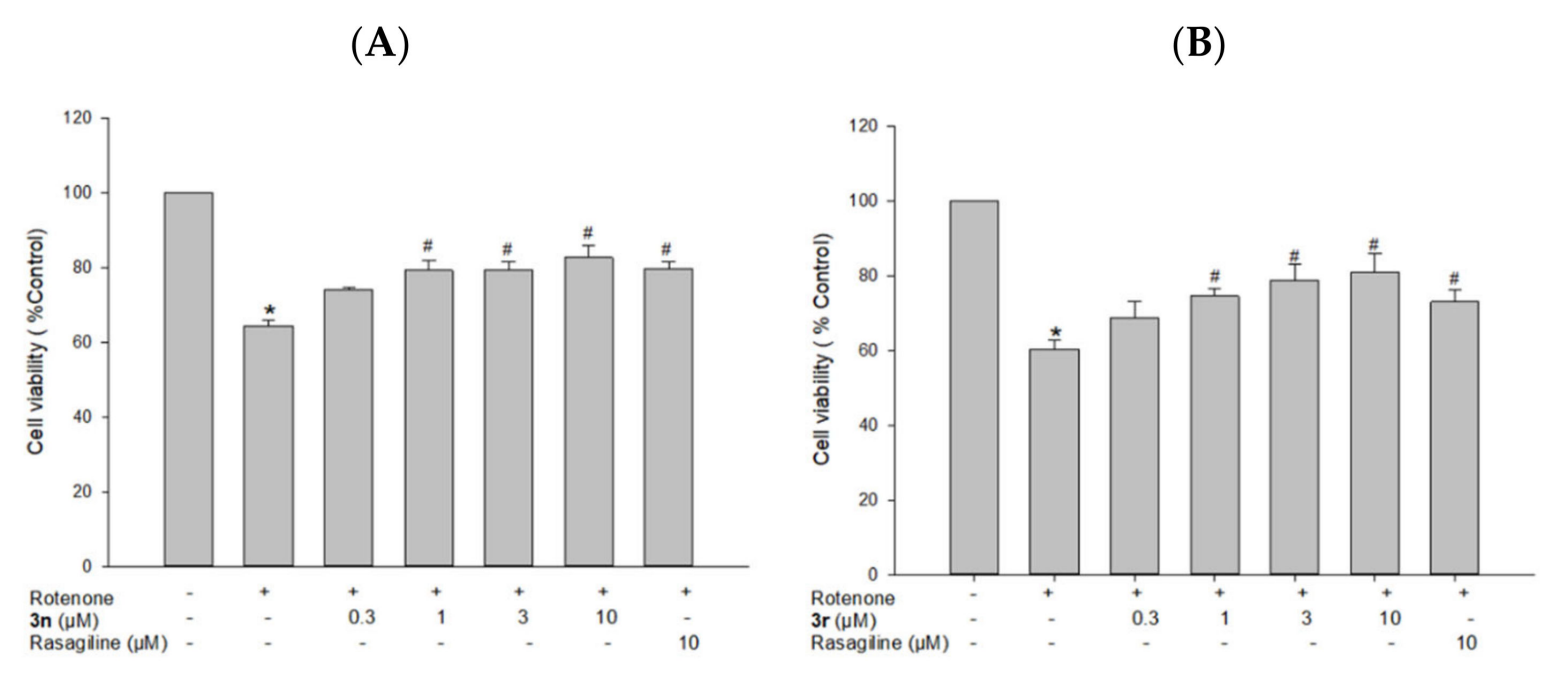
Neuroprotective effects of compounds **3n** (**A**) and **3r** (**B**) on rotenone-induced toxicity in PC12 cells. PC12 cells were pre-treated with the test compounds or rasagiline at the indicated concentrations for 4 h and exposed to rotenone (1 μM) for an additional 24 h. Cell viability was evaluated by MTT assay as described in the Materials and Methods section. Data are expressed as the mean ± SEM of at least three independent experiments. * *p* < 0.05 and # *p* < 0.05 vs. the vehicle-treated control and rotenone-treated cells, respectively.

Rotenone, a well-known potent mitochondrial complex I inhibitor, is commonly used to produce neurotoxicity mimicking pathogenetic processes of PD [109,110]. To confirm the neuroprotective effects of the melatonin analogues, **3n** and **3r**, we further evaluated their effects on rotenone-induced toxicity in PC12 cells. As shown in Figure 13, the exposure of PC12 cells to rotenone at 1 μM resulted in the cell viability of 64.4 ± 1.4% compared with the control cells. Pre-treatment with **3n** significantly protected cells from the rotenone-induced toxicity, exhibiting a maximum cell viability up to 82.7 ± 3.2% at 10 μM (Figure 13A). The calculated IC_50_ value was 1.4 μM. Similarly, **3r** protected cells from the rotenone-induced toxicity, with a maximum cell viability of 80.9 ± 4.9% at 10 μM (Figure 13B) and an IC_50_ value of 0.87 μM. In addition, rasagiline at 10 μM increased cell viability to approximately 75%.

#### 3.5.4. Effects of Compounds **3n** and **3r** on LPO

**Figure 14:**
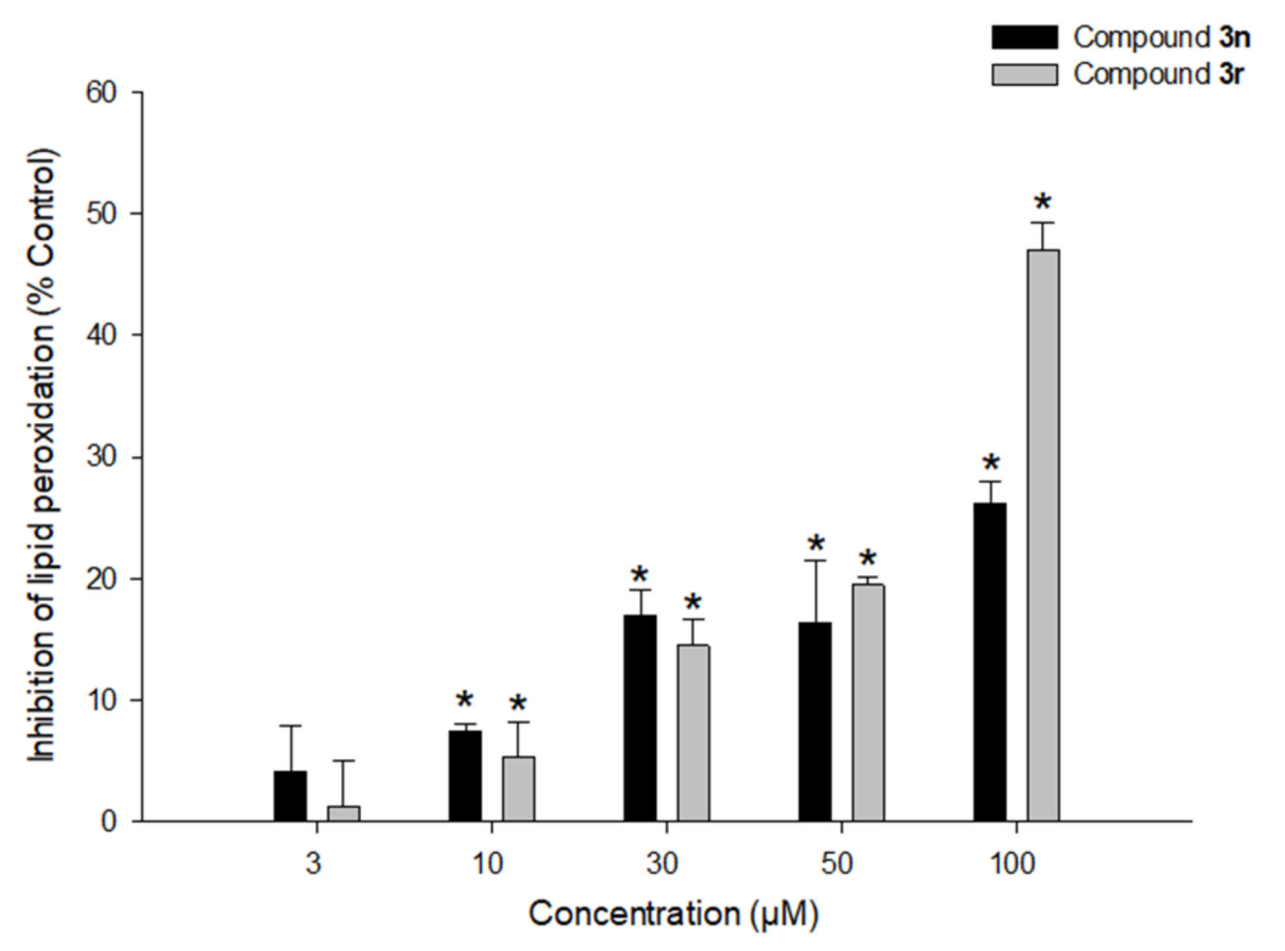
Effects of compounds **3n** and **3r** on LPO. Inhibition of LPO caused by Fe^2+^ (10 µM) and L-ascorbic acid (100 µM) in rat forebrain homogenates by the tested compounds at the indicated concentrations were measured as described in the Materials and Methods section. Each data point represents the mean ± SEM from at least three independent experiments conducted in duplicate (* *p* < 0.05 vs. LPO measured in the absence of the test compounds).

The antioxidant potential of compounds **3n** and **3r** was further explored by assessing their ability to prevent LPO induced by Fe^2+^ and L-ascorbic acid in rat brain homogenates. Compound **3r** partly reduced lipid peroxide formation in rat brain homogenates by 44.4 ± 2.2% at 100 μM, while compound **3n** showed 23.5 ± 1.7% inhibition at the same concentration (Figure 14).

#### 3.5.5. Up-Regulation of HO-1 Expression by Compounds **3n** and **3r** and Its Role in Neuroprotection

**Figure 15:**
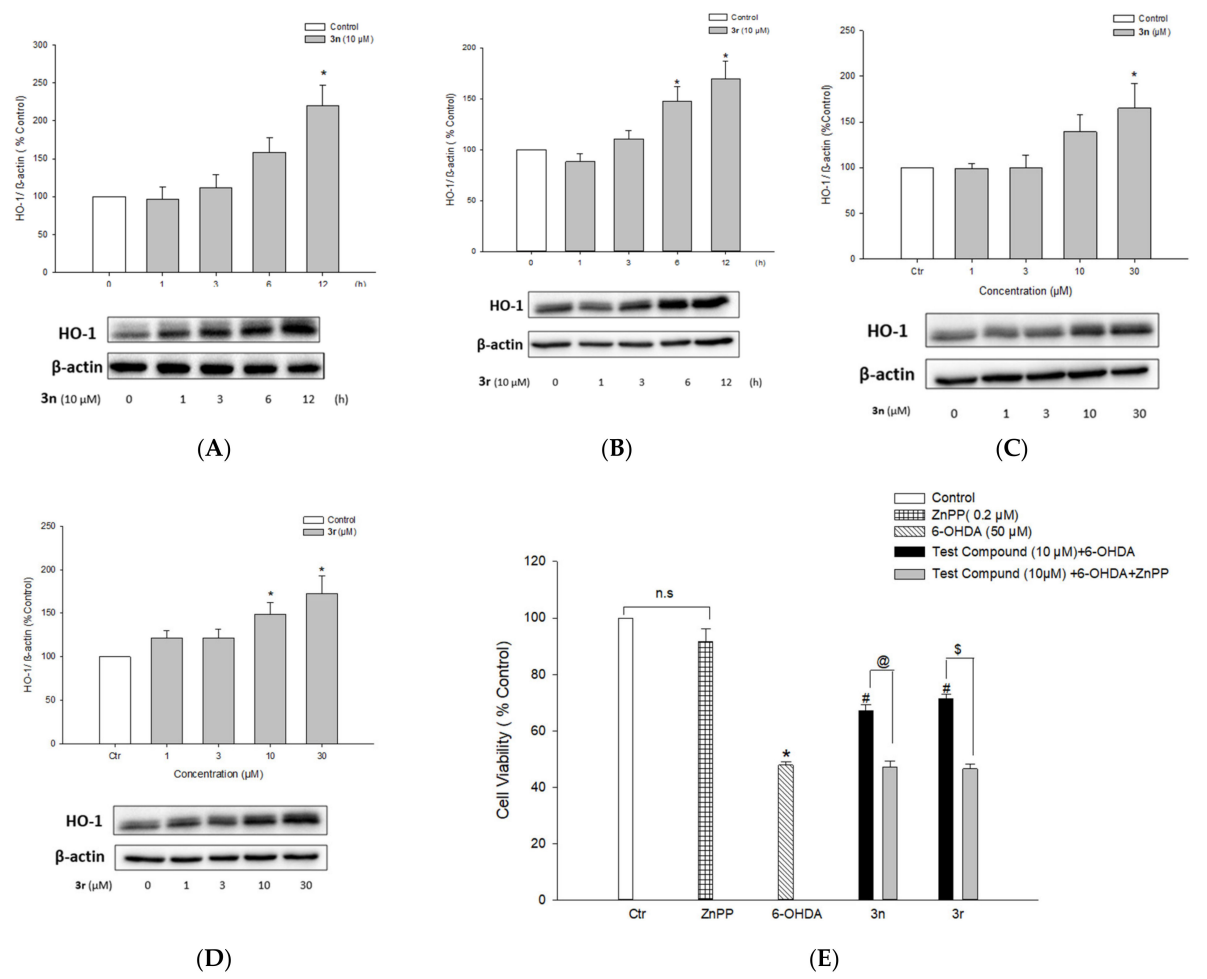
Up-regulation of HO-1 expression by compounds **3n** and **3r** and its role in neuroprotective effects. (**A**–**D**) PC12 cells were treated with compound **3n** (**A**) or **3r** (**B**) at 10 µM for the indicated periods of time or the cells were treated with a series of concentrations of compound **3n** (**C**) or **3r** (**D**) for 12 h. The HO-1 protein expression was evaluated by Western blotting as described in the Materials and Methods section. Band intensity was quantified by densitometric analyses and normalized to β-actin. (**E**) PC12 cells pre-treated with 0.2 µM ZnPP for 30 min were exposed to the test compounds for 4 h, which was followed by incubation with 6-OHDA at the final concentration of 50 µM for an additional 24 h. Cell viability was assessed by MTT reduction assay. Each data point represents the mean ± SEM from at least three measurements performed in duplicate (Ctr, control, * *p* < 0.05 vs. the vehicle-treated control cells; # *p* < 0.05 vs. 6-OHDA-treated cells; @ *p* < 0.05 vs. the cells treated with compound **3n** and 6-OHDA without ZnPP; $ *p* < 0.05 vs. the cells treated with compound **3r** and 6-OHDA without ZnPP; n.s., not significant). 6-OHDA, 6-hydroxydopamine.

The effect of compounds **3n** and **3r** on the expression of HO-1 was studied to understand the molecular mechanism(s) underlying their neuroprotective effects. PC12 cells were treated with various concentrations of **3n** or **3r** (1, 3, 10, and 30 μM) for 12 h or with 10 μM of the investigated compounds for the indicated periods of time (from 1 to 12 h). Protein expression of HO-1 was determined by Western blotting. As shown in Figure 15A,B, treatment with compounds **3n** and **3r** resulted in time-dependent increases in HO-1 protein expression, exhibiting statistically significant increases at 6–12 h of treatment. In addition, there were concentration-dependent inductions in HO-1 expression, showing 1.6- and 1.7-fold increases at 30 μM of compounds **3n** and **3r**, respectively (Figure 15C,D). To further elucidate the role of HO-1 induction by compounds **3n** and **3r**, cells were treated with ZnPP, a well-known inhibitor of HO-1, and the cell viabilities were assessed by MTT assay. ZnPP completely abolished the protective effects of **3n** and **3r** against 6**-**OHDA-induced toxicity (Figure 15E). The viability of cells treated with ZnPP and **3n** or **3r** was similar to that of cells treated with 6-OHDA alone. Treatment with ZnPP alone did not significantly affect PC12 cell viability (Figure 15E). These findings indicated that compounds **3n** and **3r** counteract 6-OHDA-induced oxidative toxicity in PC12 cells through the up-regulation of HO-1 expression.

#### 3.5.6. Activation of Nrf2 Signaling by Compounds **3n** and **3r**

**Figure 16:**
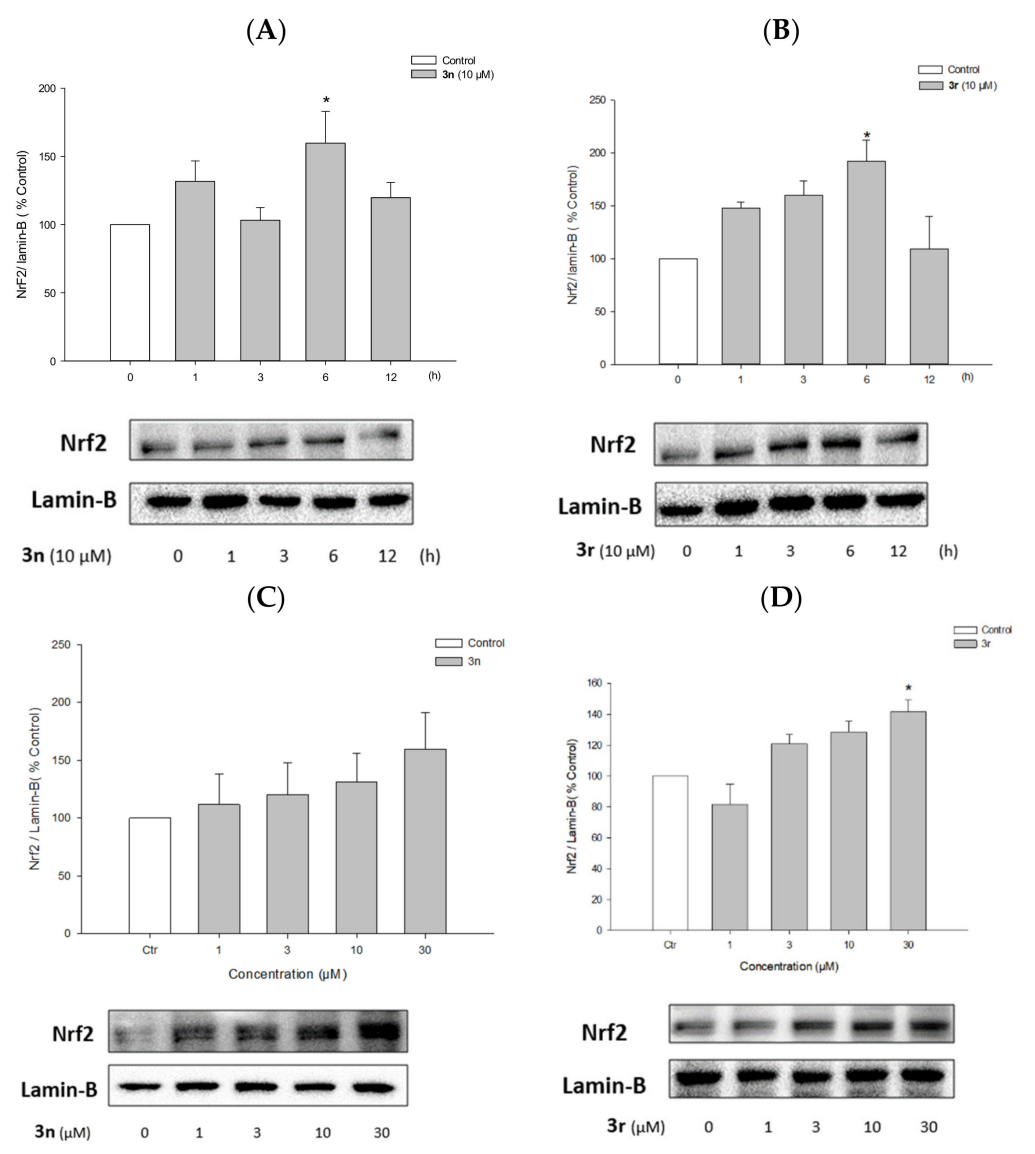
Effects of compounds **3n** and **3r** on the nuclear translocation of Nrf2. (**A**,**B**) PC12 cells were treated with compounds **3n** (**A**) and **3r** (**B**) at 10 μM for the indicated periods of time. (**C**,**D**) PC12 cells were treated with compounds **3n** (**C**) and **3r** (**D**) for 6 h at the indicated concentrations. Nrf2 protein expression in the nuclear fraction was analyzed by Western blotting as described in the Materials and Methods section. Lamin-B was used as the internal control. (Ctr, control, * *p* < 0.05 vs. the vehicle-treated control cells).

Nrf2 is a transcription factor involved in the expression of antioxidant defense enzymes in response to oxidative stress. Upon nuclear translocation, Nrf2 binds to antioxidant response elements (AREs), leading to the transcription of ARE genes such as HO-1 [111]. Compounds **3n** and **3r** up-regulated HO-1 expression in PC12 cells (Figure 15A–D); therefore, we examined whether these compounds could enhance Nrf2 nuclear translocation. Western blotting revealed that compounds **3n** and **3r** increased the nuclear translocation of Nrf2 in PC12 cells (Figure 16). Nrf2 protein expression peaked after 6 h of treatment with each compound at 10 μM (Figure 15A,B). Reaching the maximal translocation of Nrf2 at the earlier time point than HO-1 expression indicated that Nrf2 is an upstream signal of HO-1 transcription. The nuclear translocation of Nrf2 was increased by **3n** and **3r** in concentration-dependent manners (Figure 16C,D).

## 4. Conclusions

As a step toward the development of multi-targeted candidates for oxidative stress-related PD therapy, we successfully synthesized 24 melatonin analogues (**3a**–**x**). Among them, 12 compounds suppressed MAO-B by >50% at a single dosage concentration of 10 µM. In dose-dependent assay, compounds **3n**, **3r**, and **3u**–**w** demonstrated potent IC_50_ values over MAO-B (1.41, 0.91, 1.20, 0.66, and 2.41 µM, respectively) with significant better selectivity indices compared with rasagiline. Most synthesized compounds were predicted to have a good solubility profile and cross the BBB. Furthermore, no violations of the Lipinski rule of five were found. This means that the synthesized series is expected to be pharmacokinetically stable. The simulation docking studies provided a reasonable explanation of the elicited biological activities and highlighted the promising potential of the most active compounds for further evaluation. Compounds **3n** and **3r** showed a safe toxicity profile and effectively reduced 6-OHDA- and rotenone-induced oxidative toxicity in PC12 cells through the up-regulation of the Nrf2/HO-1 signaling pathway. Accordingly, we report compounds **3n** and **3r** as new multi-targeted candidates worthy of further development for oxidative stress-related PD therapy. To further validate and expand our findings, additional studies using different experimental models of PD, including primary cultures of dopaminergic neurons, are under consideration.

## Figures and Tables

**Table 1:** Chemical structures and yields of the synthesized melatonin analogues **3a**–**x**.

Entry	Comp.	R	Isolated Yield (%)
1	**3a**	H	94
2	**3b**	2-bromo	31
3	**3c**	4-bromo	84
4	**3d**	2,4-dichloro	72
5	**3e**	3,5-dinitro	87
6	**3f**	3-nitro	85
7	**3g**	4-nitro	85
8	**3h**	2,6-difluoro	49
9	**3i**	3-fluoro	75
10	**3j**	4-fluoro	21
11	**3k**	2-fluoro	62
12	**3l**	2-trifluoromethyl, 4-fluoro	50
13	**3m**	3-cyano	79
14	**3n**	3-chloro	88
15	**3o**	2-chloro	100
16	**3p**	4-chloro	94
17	**3q**	4-*tert*-butyl	99
18	**3r**	3-bromo	34
19	**3s**	3-trifluoromethoxy	91
20	**3t**	4-trifluoromethoxy	86
21	**3u**	3-trifluoromethyl	66
22	**3v**	4-trifluoromethyl	86
23	**3w**	3,5-dichloro	52
24	**3x**	2,6-dichloro	78

**Table 2:** Predicted physicochemical, lipophilicity, and drug-likeness properties for compounds **3a**–**x**.

Comp.	MW	TPSA	GTI Absorption	BBB Permeability	Lipinski #Violations	ESOL Log S	ESOL Class
**3a**	236.27	44.89	High	Yes	0	−3.12	Soluble
**3b**	315.16	44.89	High	Yes	0	−4.43	Moderately soluble
**3c**	315.16	44.89	High	Yes	0	−4.01	Moderately soluble
**3d**	305.16	44.89	High	Yes	0	−4.70	Moderately soluble
**3e**	326.26	136.53	Low	No	0	−3.17	Soluble
**3f**	281.27	90.71	High	No	0	−3.56	Soluble
**3g**	281.27	90.71	High	No	0	−3.56	Soluble
**3h**	272.25	44.89	High	Yes	0	−3.83	Soluble
**3i**	254.26	44.89	High	Yes	0	−3.68	Soluble
**3j**	254.26	44.89	High	Yes	0	−3.26	Soluble
**3k**	254.26	44.89	High	Yes	0	−3.68	Soluble
**3l**	322.26	44.89	High	No	0	−4.49	Moderately soluble
**3m**	261.28	68.68	High	Yes	0	−3.45	Soluble
**3n**	270.71	44.89	High	Yes	0	−4.12	Moderately soluble
**3o**	270.71	44.89	High	Yes	0	−4.12	Moderately soluble
**3p**	270.71	44.89	High	Yes	0	−3.69	Soluble
**3q**	292.37	44.89	High	Yes	0	−4.77	Moderately soluble
**3r**	315.16	44.89	High	Yes	0	−4.43	Moderately soluble
**3s**	320.27	54.12	High	Yes	0	−4.54	Moderately soluble
**3t**	320.27	54.12	High	Yes	0	−4.54	Moderately soluble
**3u**	304.27	44.89	High	Yes	0	−4.34	Moderately soluble
**3v**	304.27	44.89	High	Yes	0	−4.34	Moderately soluble
**3w**	305.16	44.89	High	Yes	0	−4.28	Moderately soluble
**3x**	305.16	44.89	High	Yes	0	−4.70	Moderately soluble

**Table 3:** Inhibitory effects of the melatonin analogues **3a**–**x** against MAO enzymes.

Entry	Comp.	% Inhibition of MAO-B at 10 µM	MAO-B IC_50_ (µM)	MAO-A IC_50_ (µM)	Selectivity Index (SI) ^a^
1	**3a**	14.86 ± 0.80	-	-	-
2	**3b**	0.27 ± 0.81	-	-	-
3	**3c**	37.09 ± 0.70	-	-	-
4	**3d**	22.92 ± 0.70	-	-	-
5	**3e**	2.75 ± 0.20	-	-	-
6	**3f**	54.25 ± 0.37	-	-	-
7	**3g**	60.98 ± 0.69	-	-	-
8	**3h**	−0.02 ± 0.26	-	-	-
9	**3i**	35.29 ± 0.76	-	-	-
10	**3j**	58.33 ± 1.02	-	-	-
11	**3k**	37.86 ± 0.33	-	-	-
12	**3l**	1.49 ± 0.26	-	-	-
13	**3m**	8.33 ± 0.59	-	-	-
14	**3n**	76.79 ± 0.27	1.41	>100	>71
15	**3o**	2.39 ± 0.31	-	-	-
16	**3p**	57.98 ± 0.07	-	-	-
17	**3q**	50.01 ± 0.04	-	-	-
18	**3r**	87.00 ± 0.03	0.91	>100	>109
19	**3s**	68.34 ± 0.25	-	-	-
20	**3t**	63.23 ± 0.45	-	-	-
21	**3u**	85.12 ± 0.22	1.20	>100	>83
22	**3v**	91.22 ± 0.11	0.66	>100	>151
23	**3w**	72.63 ± 0.32	2.41	>100	>41
24	**3x**	0.89 ± 0.31	-	-	

^a^ SI  = selectivity index, the selectivity for the MAO-B isoform (IC_50_ ratio (MAO-A)/IC_50_ (MAO-B)); -: Not tested.

## Data Availability

All of the data are contained within the article and the Supplementary Materials.

## Supplementary Materials

The following are available online at https://www.mdpi.com/article/10.3390/antiox10101604/s1, ^1^HNMR, ^13^CNMR, purity and HRMS data of the compounds reported in this study, in addition to the detailed Material and methods of chemical synthesis and biological evaluations over MAO enzymes.
