# Supplementary material for: Melatonin Analogues Potently Inhibit MAO-B and Protect PC12 Cells against Oxidative Stress
Source: Antioxidants (Basel). 2021 Oct 12;10(10):1604. doi: 10.3390/antiox10101604 (PMC8533333; doi:10.3390/antiox10101604)
Supplement: Supplementary file 1 [file antioxidants-10-01604-s001.zip › antioxidants-1405240-supplementary.pdf]

Supplementary material

Melatonin Analogues Potently Inhibit MAO-B and Protect PC12 Cells against Oxidative Stress

Contents

1. General methods and instruments (Chemistry)
2. ^{1}HNMR and ^{13}CNMR charts
3. HPLC purity data charts
4. HRMS charts
5. IR charts
6. Materials and methods of monoamine oxidase (MAO) enzyme assay

References

1. General methods and instruments (Chemistry)

All of the commercial chemicals were of reagent grade and were used without further purification. Melting points were measured on the Thermo Scientific 9200 apparatus. Proton nuclear magnetic resonance (^{1}H NMR) spectra were determined on a Varian (400 MHz) spectrometer (Varian Medical Systems, Inc., Palo Alto, CA, U.S.A.). Multiplicity is indicated by the following abbreviations: singlet (s), doublet (d), doublet of doublet (dd), triplet (t), quartet (q), multiplet (m), and broad (b). The values of the chemical shifts are expressed in δ values (ppm), and the coupling constants (J) are reported in hertz. ^{13}C NMR spectra were recorded on a Varian (100 MHz) spectrometer. Chemical shifts are provided in parts per million (ppm) downfield from tetramethylsilane (internal standard) with coupling constants in hertz. Mass spectra were recorded using high-resolution mass spectrometry (HRMS, ESI−MS), obtained on a G2 QTOF mass spectrometer (Waters Corporation, Milford, MA, U.S.A.). Infrared (IR) spectra were recorded as KBr disks using a Shimadzu FT-IR 8400S infrared spectrophotometer. Products were purified by column or flash column chromatography (Biotage, Sweden) using silica gel 60 (230–400 mesh Kieselgel 60). Additionally, thin-layer chromatography on 0.25 mm silica plates (E. Merck, silica gel 60 F254) was used to monitor reactions. Spots were detected by viewing under ultraviolet (UV) light. The optical purity of the synthesized compounds was established by chiral high-performance liquid chromatography (HPLC) analysis: Chiralpak IG-3 (4.6 × 150, 3), hexane/EtOH/ MeOH = 85:10:5, 1.5 mL/min, and λ = 280 nm.

2. ^{1}HNMR and ^{13}CNMR charts

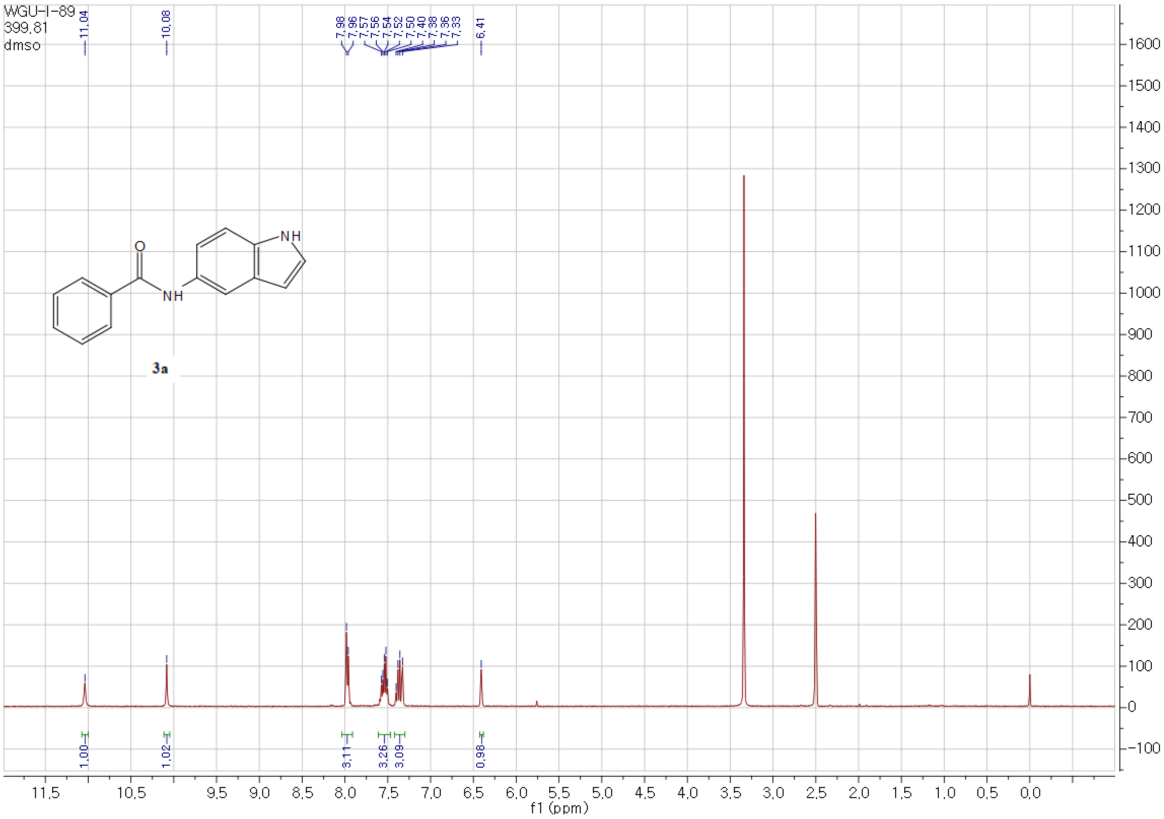

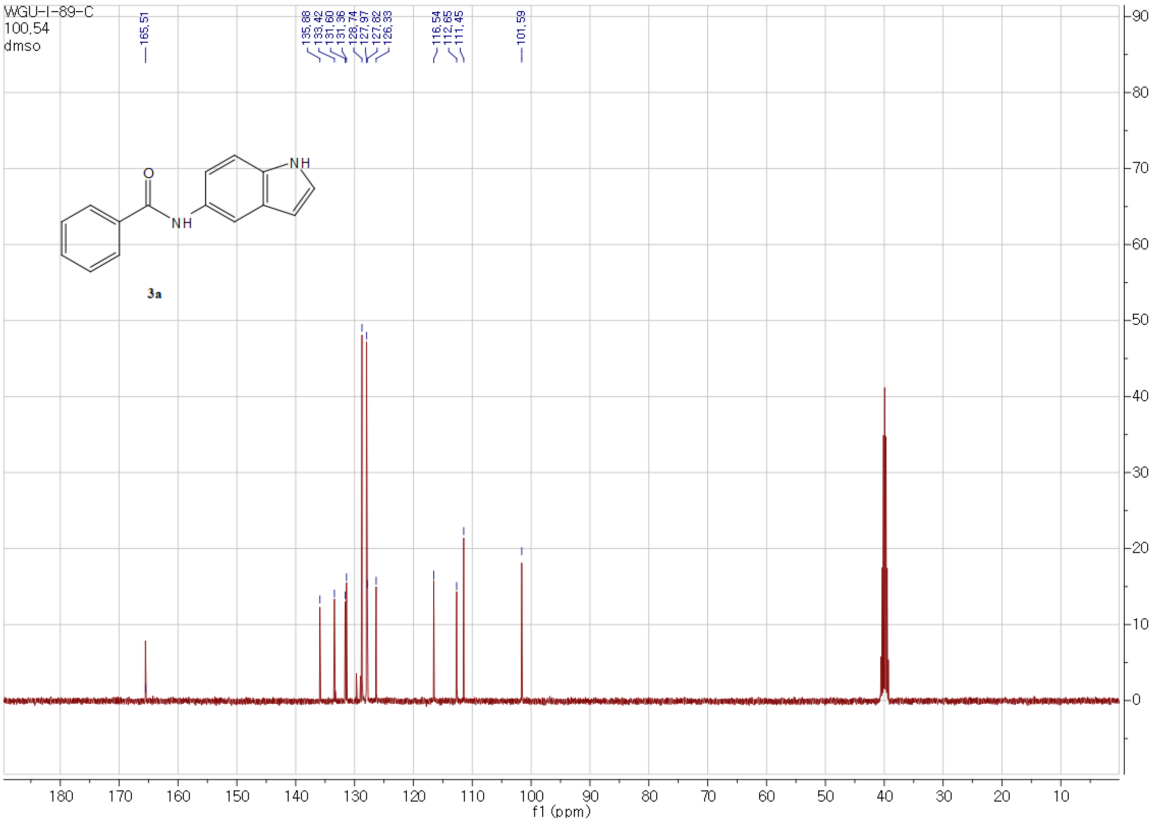

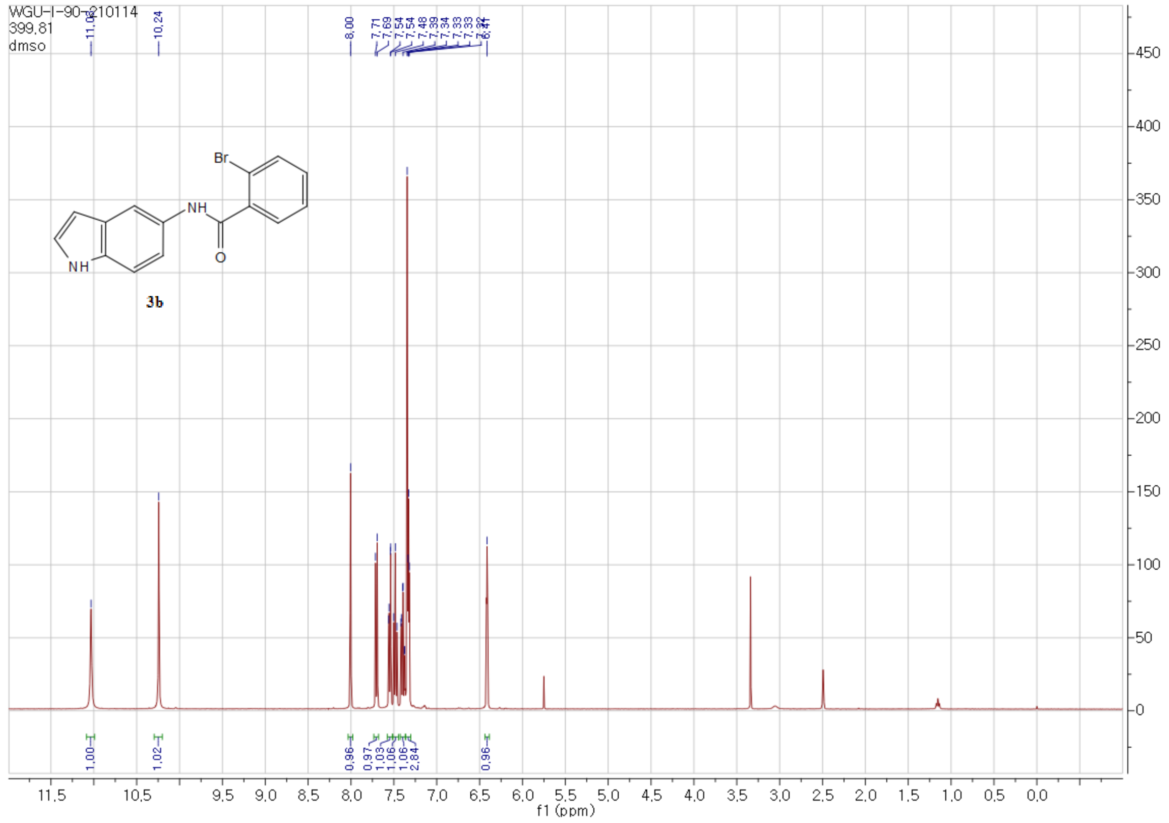
WGU-I-90-210114
399.81
dmso
11.04
10.24
8.00
7.71
7.69
7.54
7.54
7.48
7.39
7.34
7.33
7.33
7.32
6.41
Br
NH
O
NH
3b
1.00
1.02
0.96
0.97
1.03
1.06
1.06
2.84
0.96
f1 (ppm)

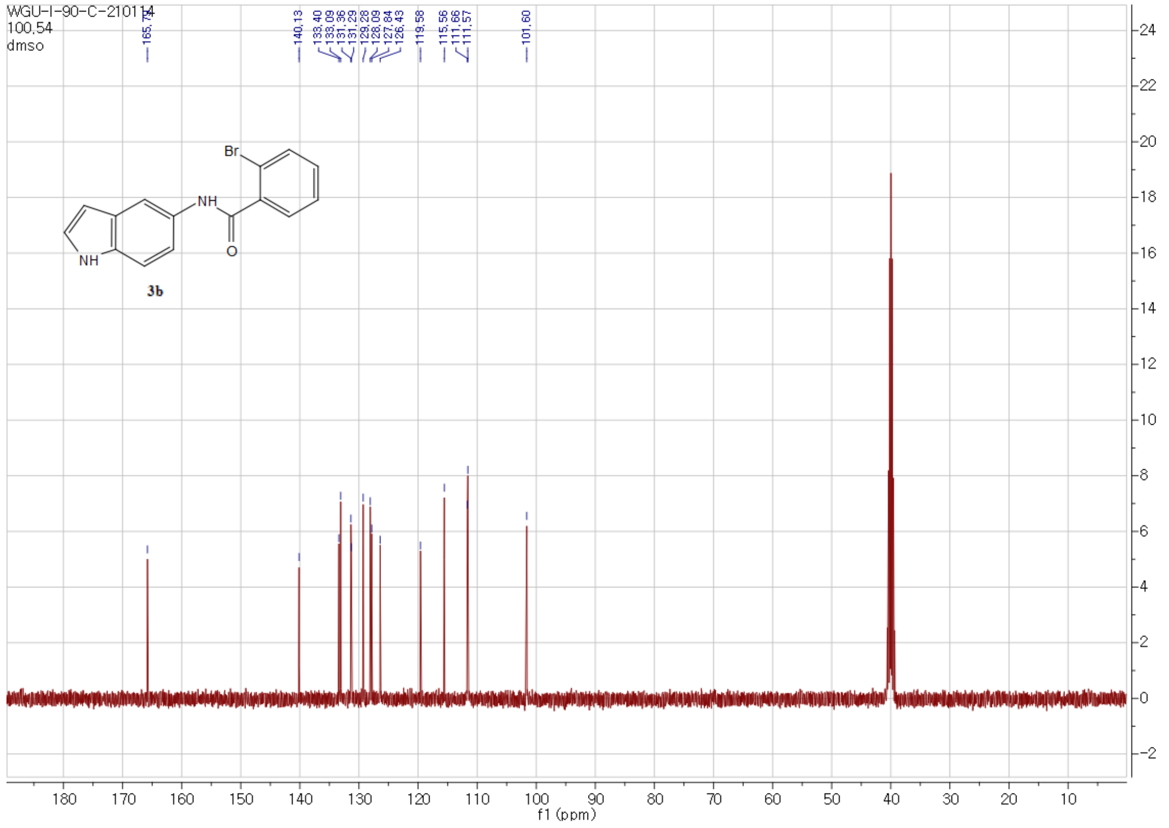
WGU-I-90-C-210114
100.54
dmso
165.74
140.13
133.40
133.09
131.36
131.29
129.28
128.09
127.84
126.43
119.58
115.56
111.66
111.57
101.60
Br
NH
O
NH
3b
f1 (ppm)

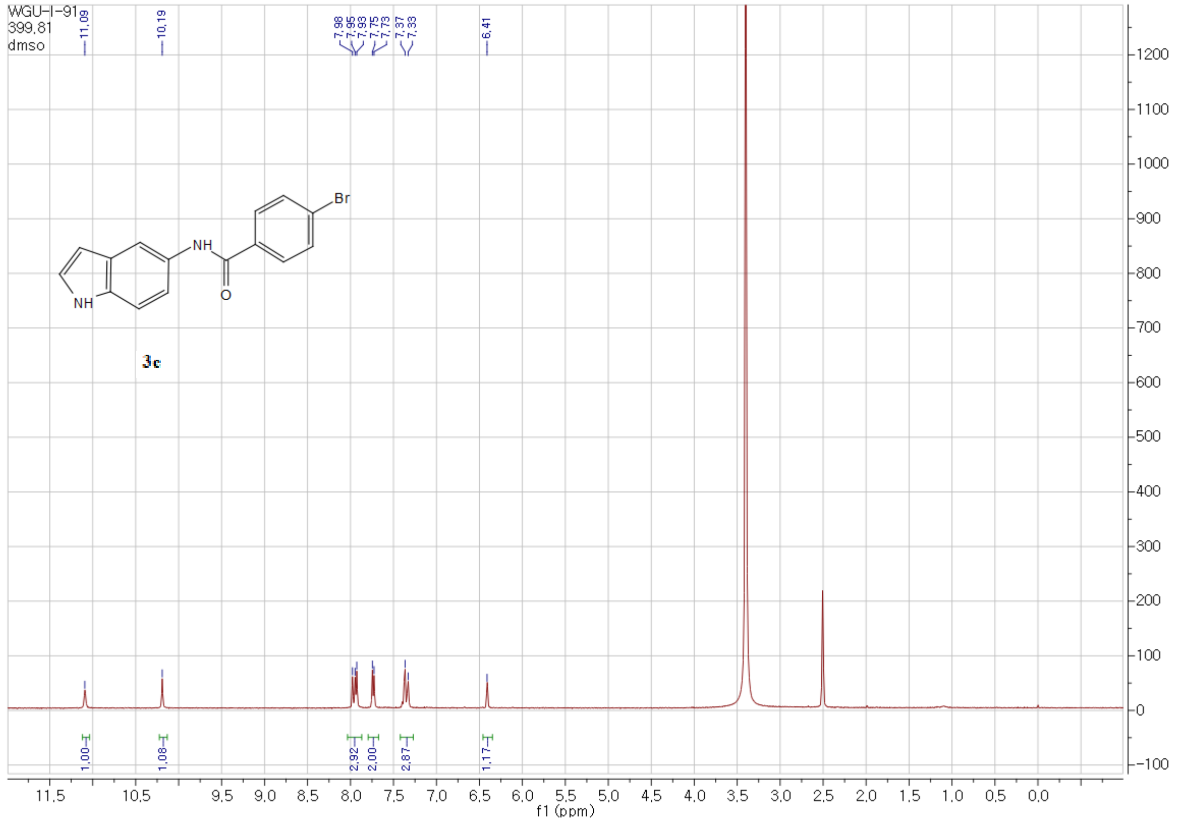
WGU-I-91
399.81
dmso
11.09
10.19
7.98
7.95
7.93
7.75
7.73
7.37
7.33
6.41
NH
Br
NH
O
3c
1.00
1.08
2.92
2.00
2.87
1.17
f1 (ppm)

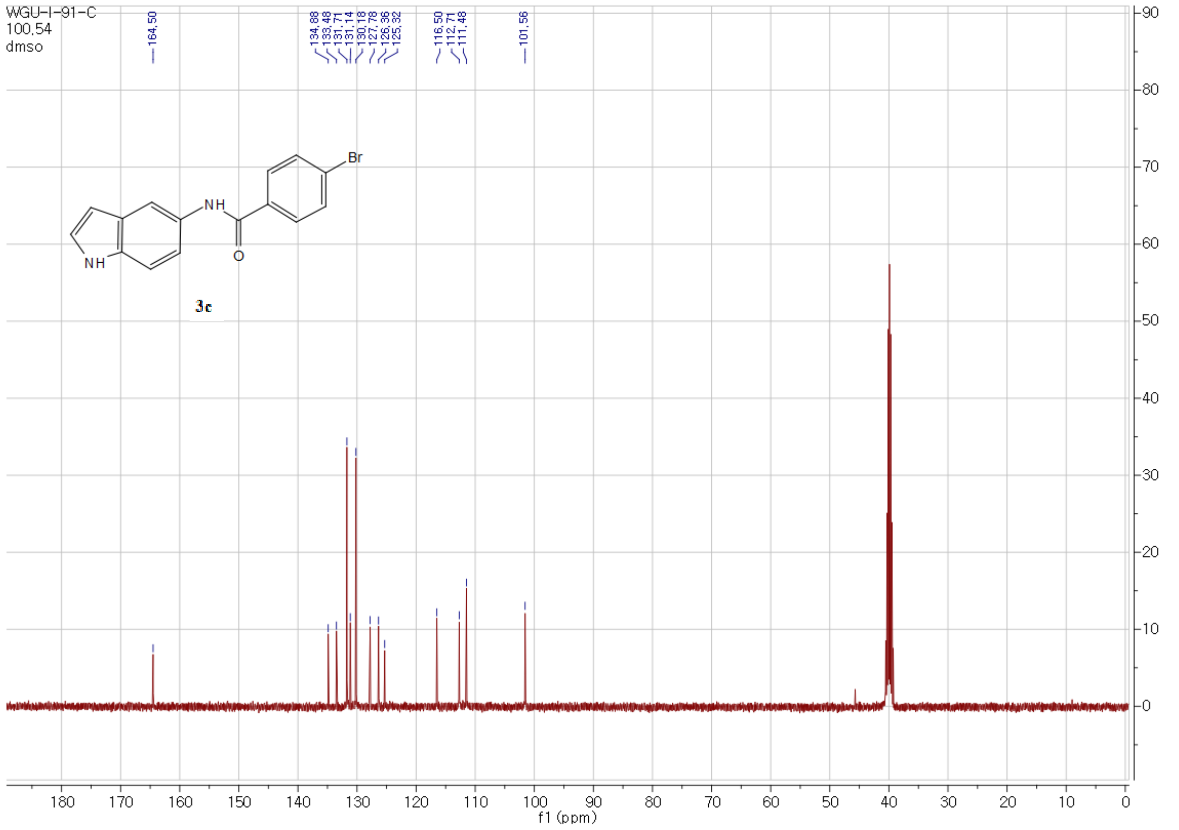
WGU-I-91-C
100.54
dmso
164.50
134.88
133.48
131.71
131.14
130.18
127.78
126.36
125.32
116.50
112.71
111.48
101.56
NH
Br
NH
O
3c
f1 (ppm)

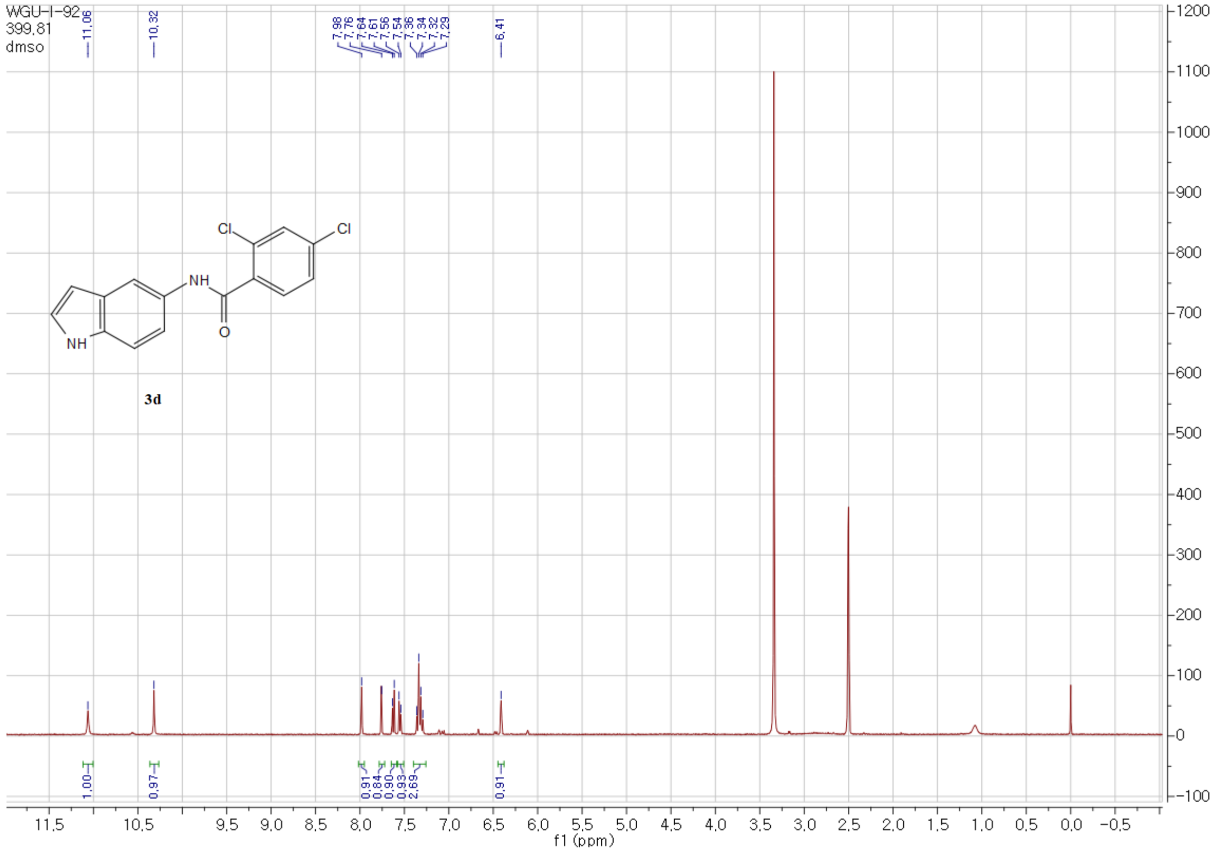
WGU-I-92
399,81
dmso
11,06
10,32
7,98 7,76 7,64 7,61 7,56 7,54 7,36 7,34 7,32 7,29
6,41
Cl
Cl
NH
O
NH
3d
1,00 0,97 0,91 0,84 0,90 0,93 2,69 0,91
f1 (ppm)

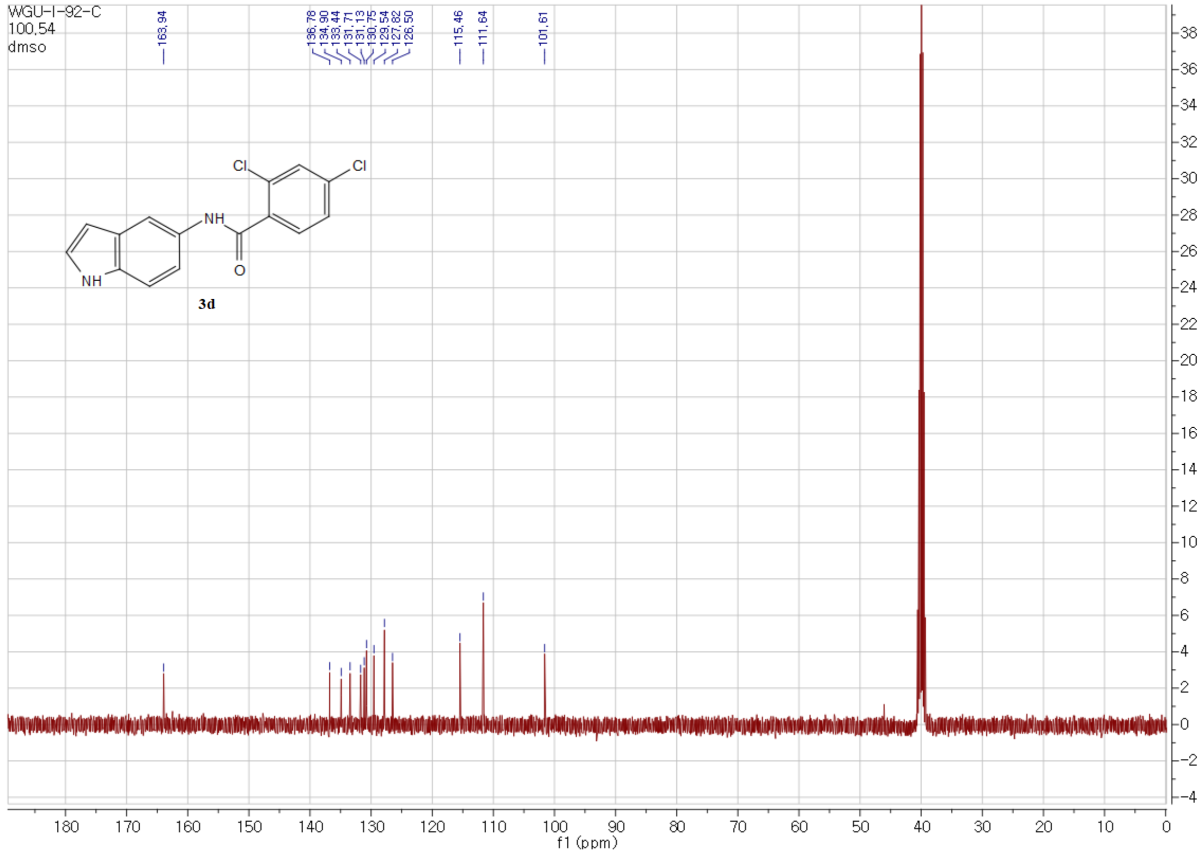
WGU-I-92-C
100,54
dmso
163,94
136,78 134,90 133,44 131,71 131,13 130,75 129,54 127,82 126,50
115,46
111,64
101,61
Cl
Cl
NH
O
NH
3d
f1 (ppm)

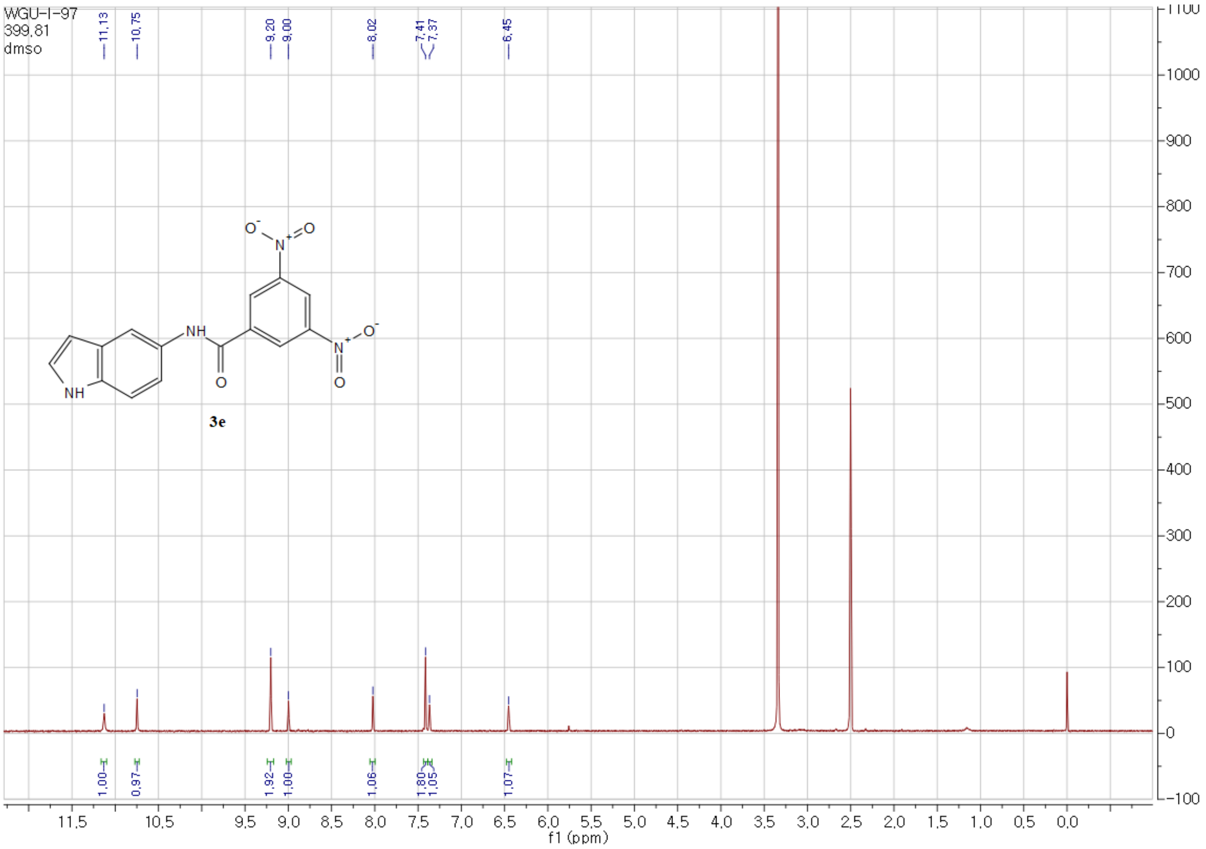
WGU-I-97
399.81
dmso
11.13
10.75
9.20
9.00
8.02
7.41
7.37
6.45
1.00
0.97
1.92
1.00
1.06
1.80
1.05
1.07
NH
NH
O
3e
f1 (ppm)

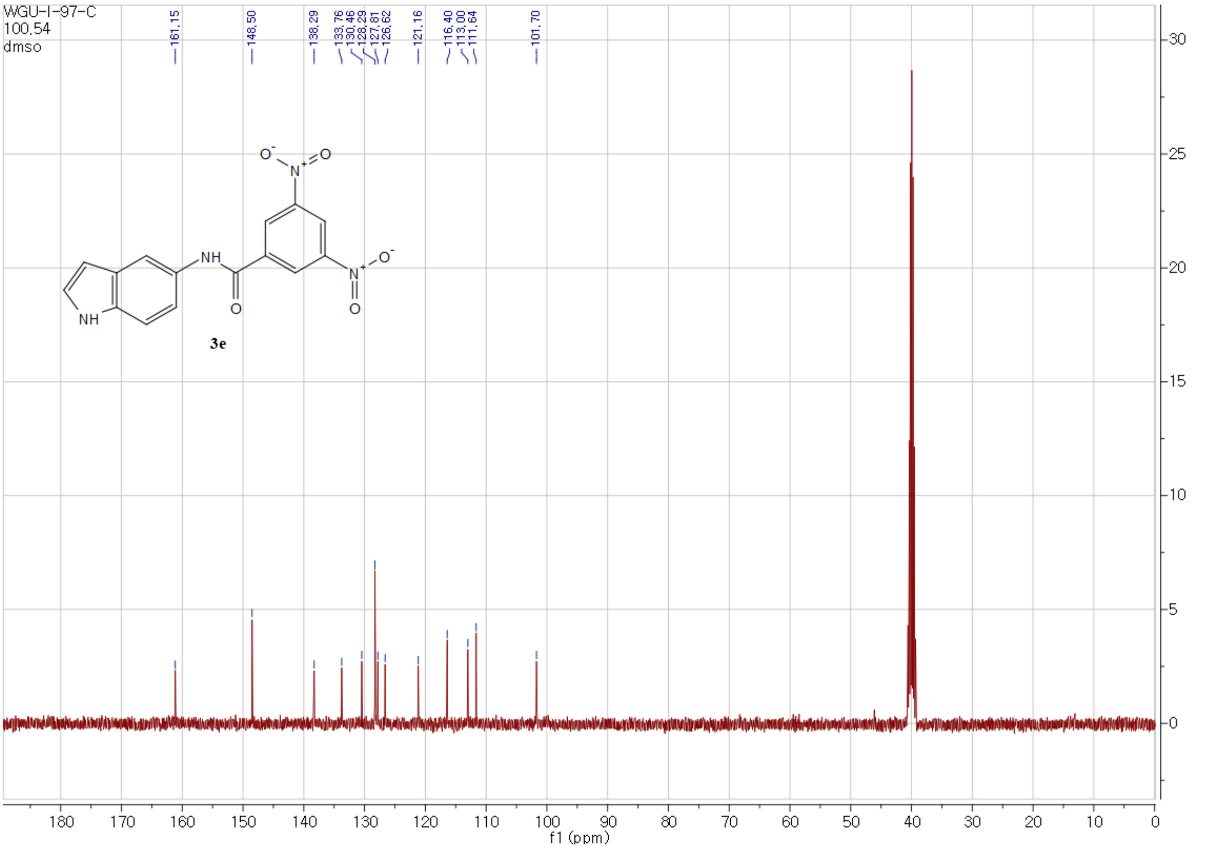
WGU-I-97-C
100.54
dmso
161.15
148.50
138.29
133.76
130.46
128.29
127.81
126.62
121.16
116.40
113.00
111.64
101.70
NH
NH
O
3e
f1 (ppm)

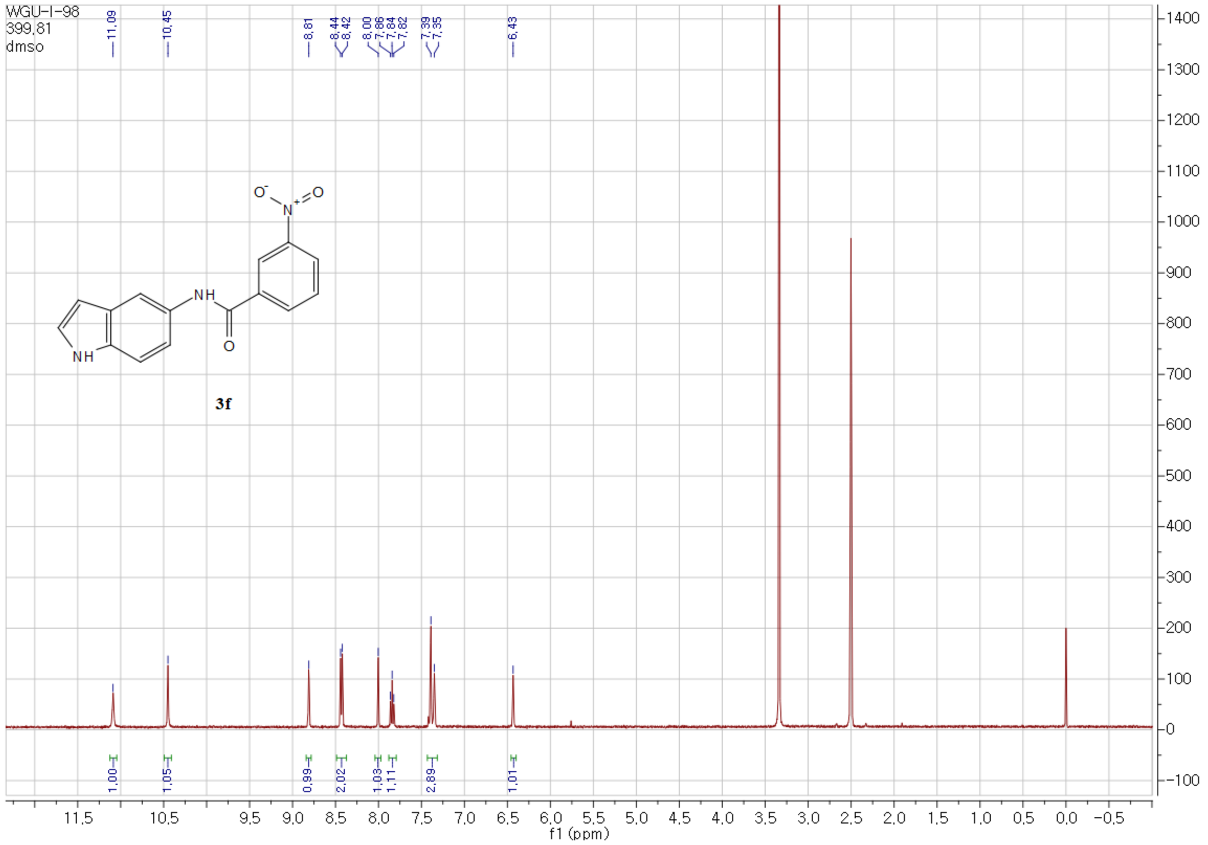
WGU-I-98
399,81
dmso
11,09
10,45
8,81
8,44
8,42
8,00
7,86
7,84
7,82
7,39
7,35
6,43
1,00
1,05
0,99
2,02
1,03
1,11
2,89
1,01
3f
f1 (ppm)

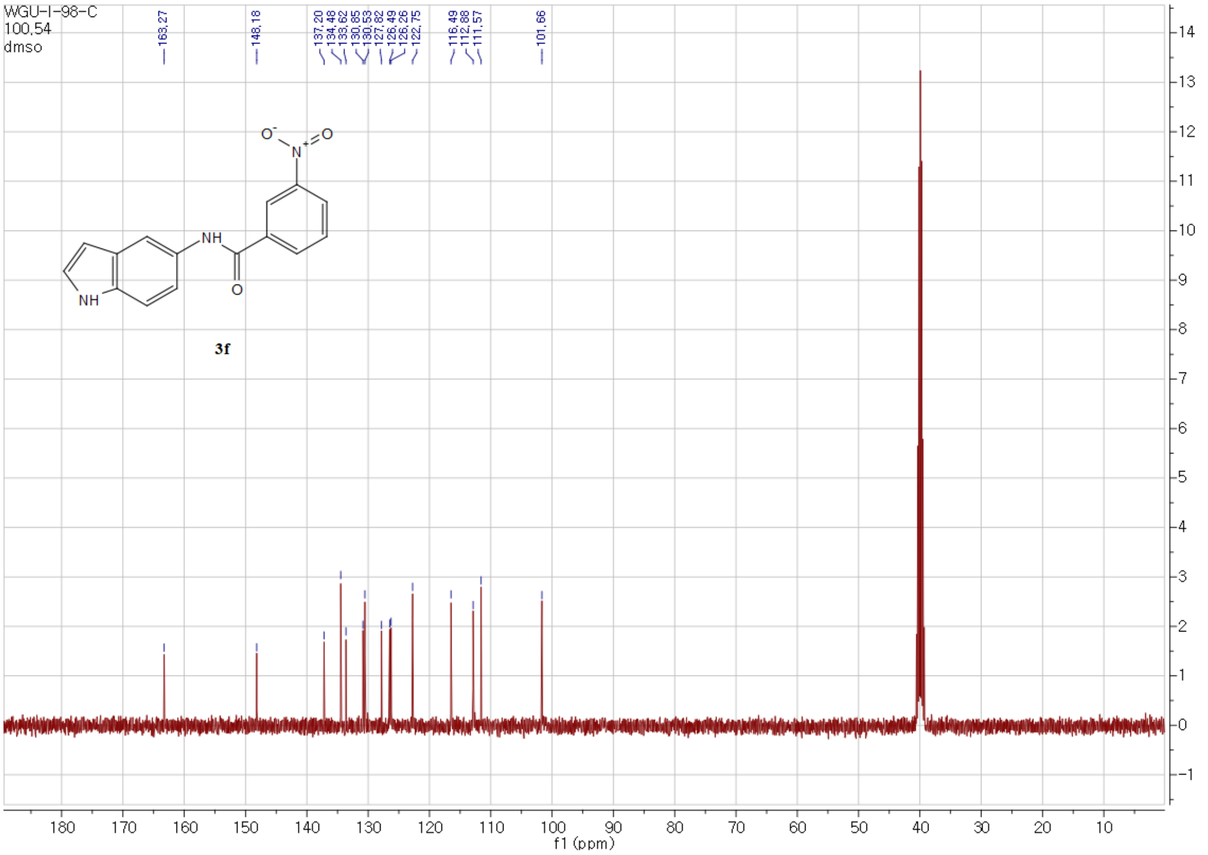
WGU-I-98-C
100,54
dmso
163,27
148,18
137,20
134,48
133,62
130,85
130,53
127,82
126,49
126,26
122,75
116,49
112,88
111,57
101,66
3f
f1 (ppm)

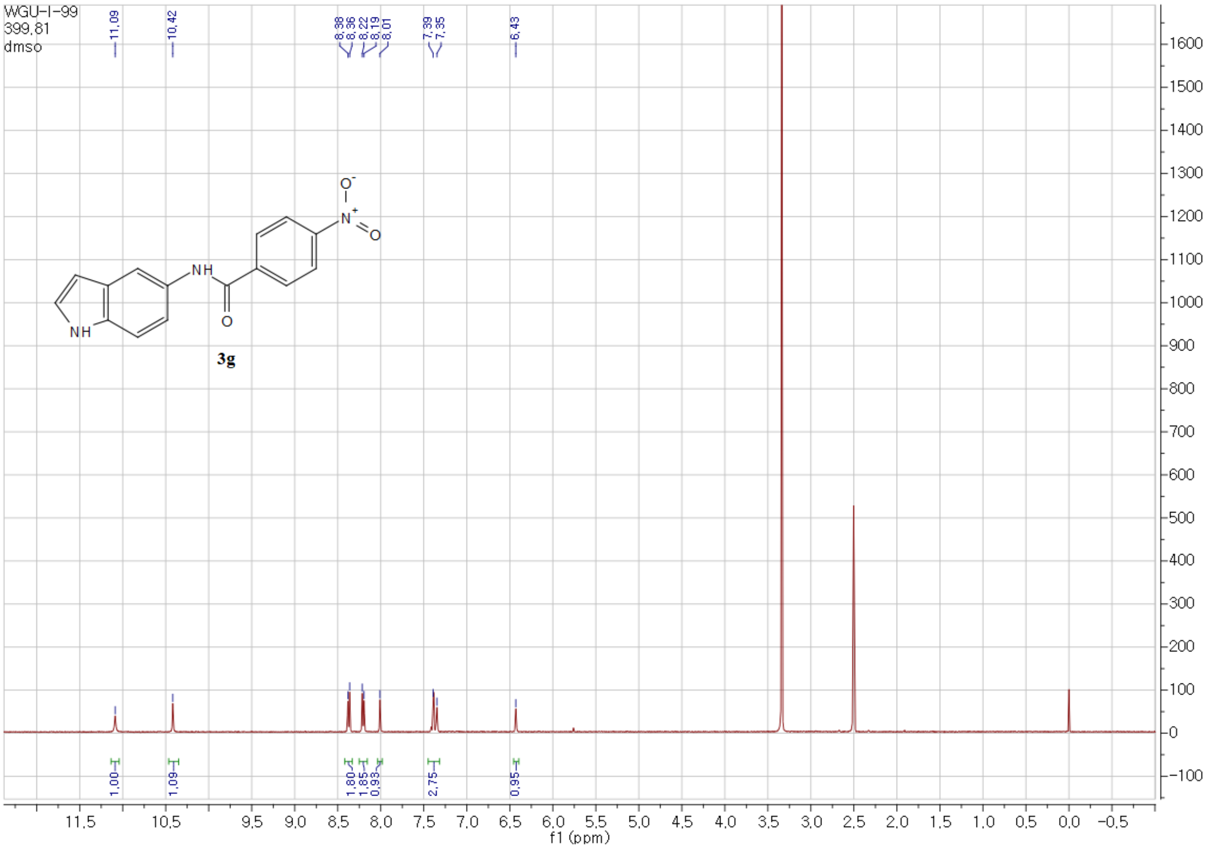
WGU-I-99
399.81
dmso
11.09
10.42
8.38
8.36
8.22
8.19
8.01
7.39
7.35
6.43
1.00
1.09
1.80
1.85
0.93
2.75
0.95
3g
f1 (ppm)

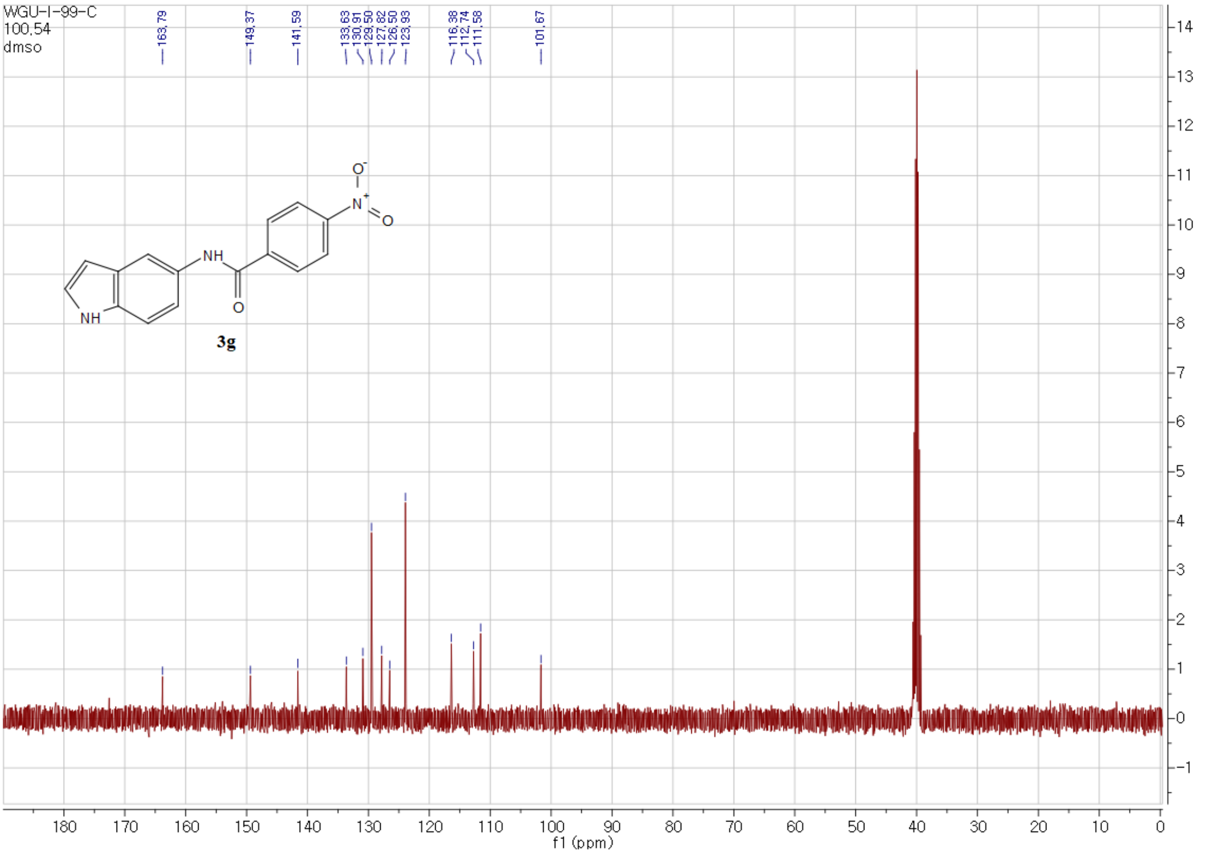
WGU-I-99-C
100.54
dmso
163.79
149.37
141.59
133.63
130.91
129.50
127.82
126.50
123.93
116.38
112.74
111.58
101.67
3g
f1 (ppm)

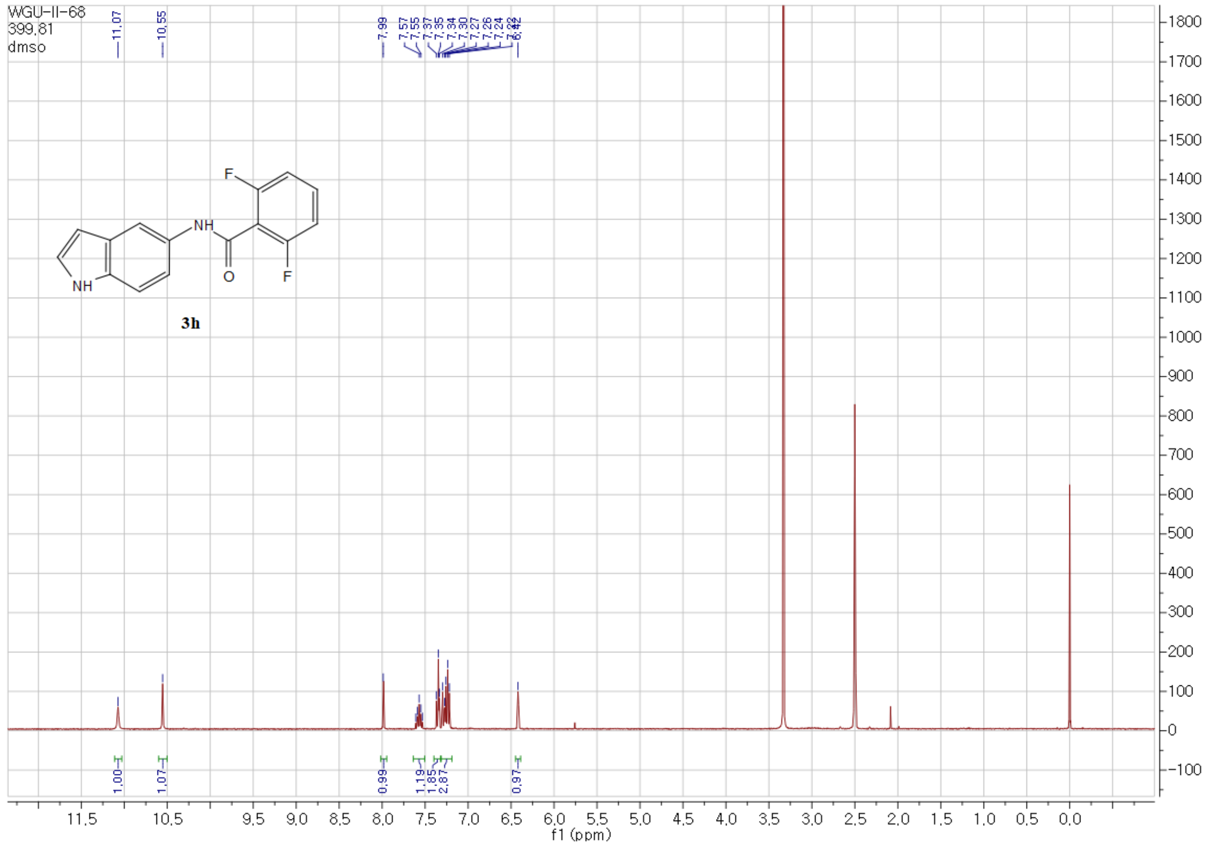
WGU-II-68
399.81
dmso
11.07
10.55
7.99
7.57
7.55
7.37
7.35
7.34
7.30
7.27
7.26
7.24
7.22
6.42
F
NH
O
F
NH
3h
1.00
1.07
0.99
1.19
1.85
2.87
0.97
f1 (ppm)

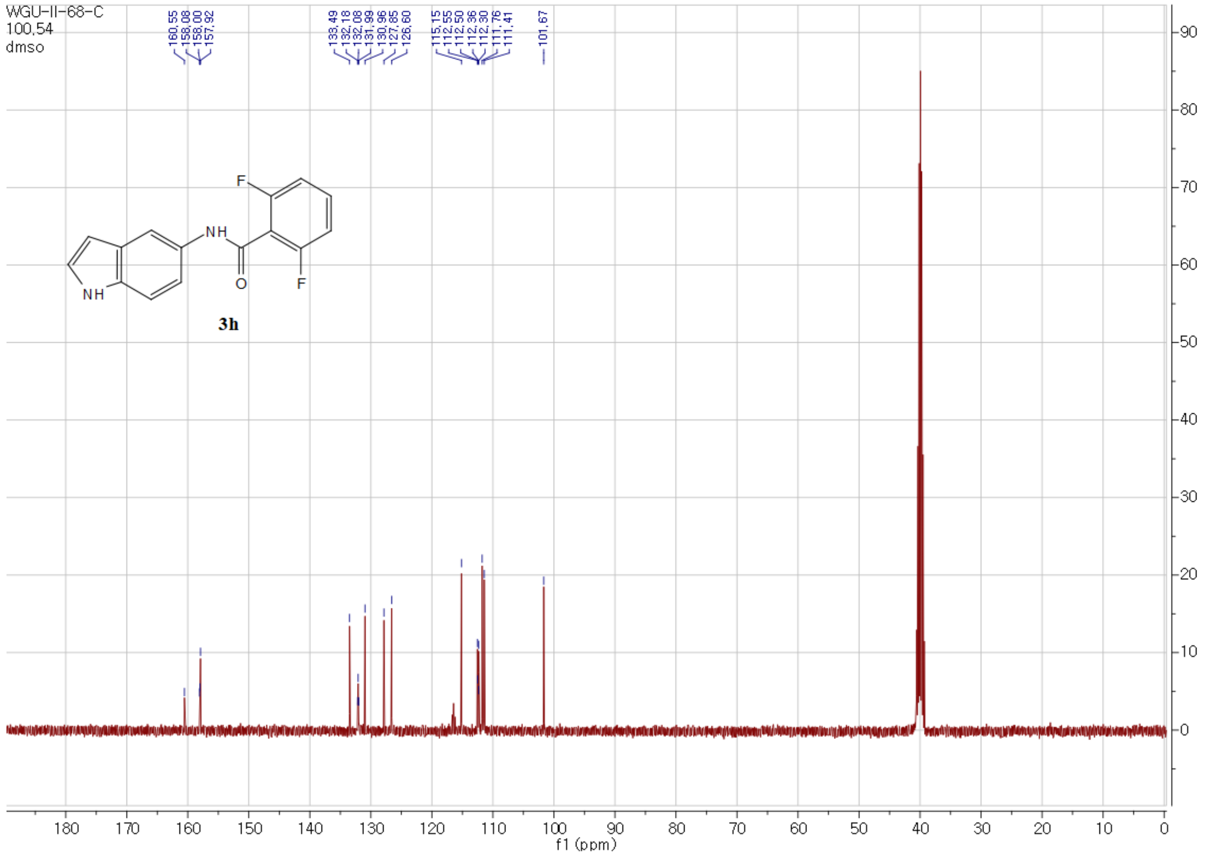
WGU-II-68-C
100.54
dmso
160.55
158.08
158.00
157.92
133.49
132.18
132.08
131.99
130.96
127.85
126.60
115.15
112.55
112.50
112.36
112.30
111.76
111.41
101.67
F
NH
O
F
NH
3h
f1 (ppm)

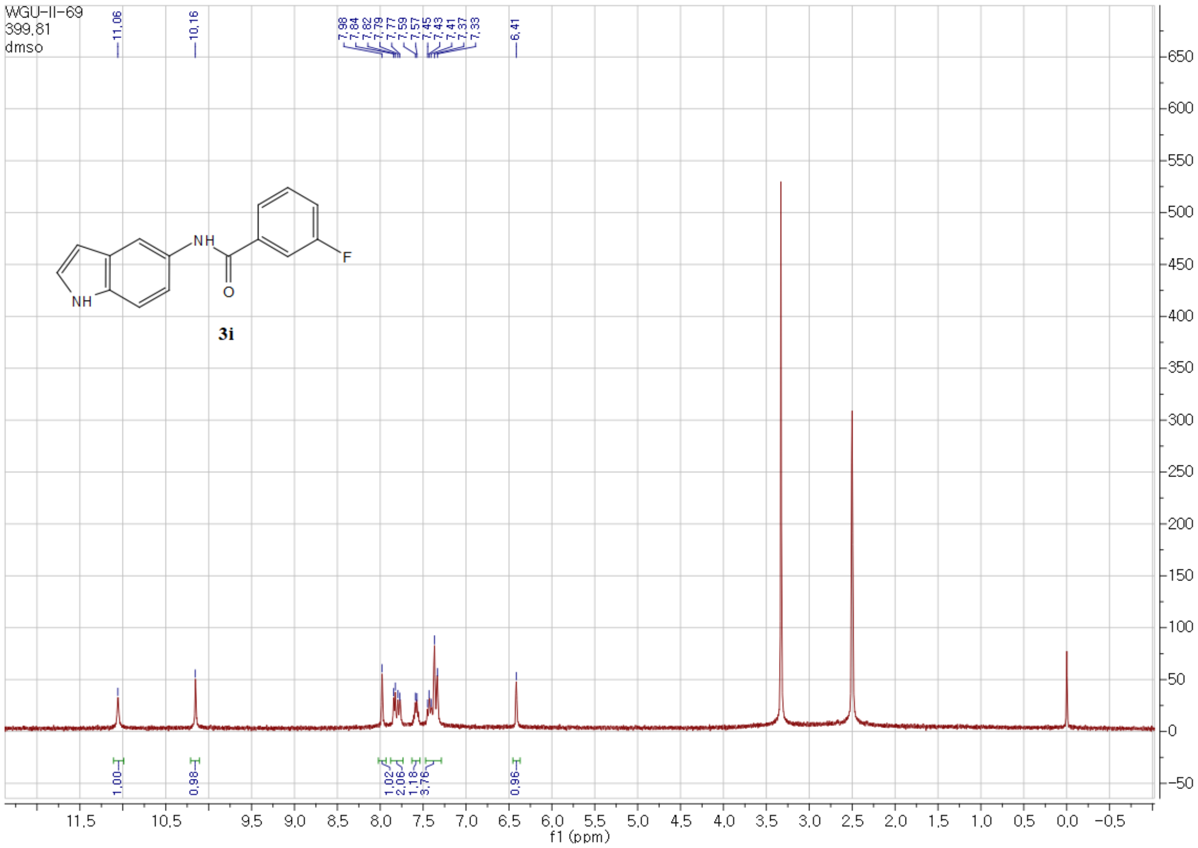
WGU-II-69
399.81
dmso
11.06
10.16
7.98 7.84 7.82 7.79 7.77 7.59 7.57 7.45 7.43 7.41 7.37 7.33
6.41
NH
O
F
NH
3i
1.00
0.98
1.02
2.06
1.18
3.76
0.96
f1 (ppm)

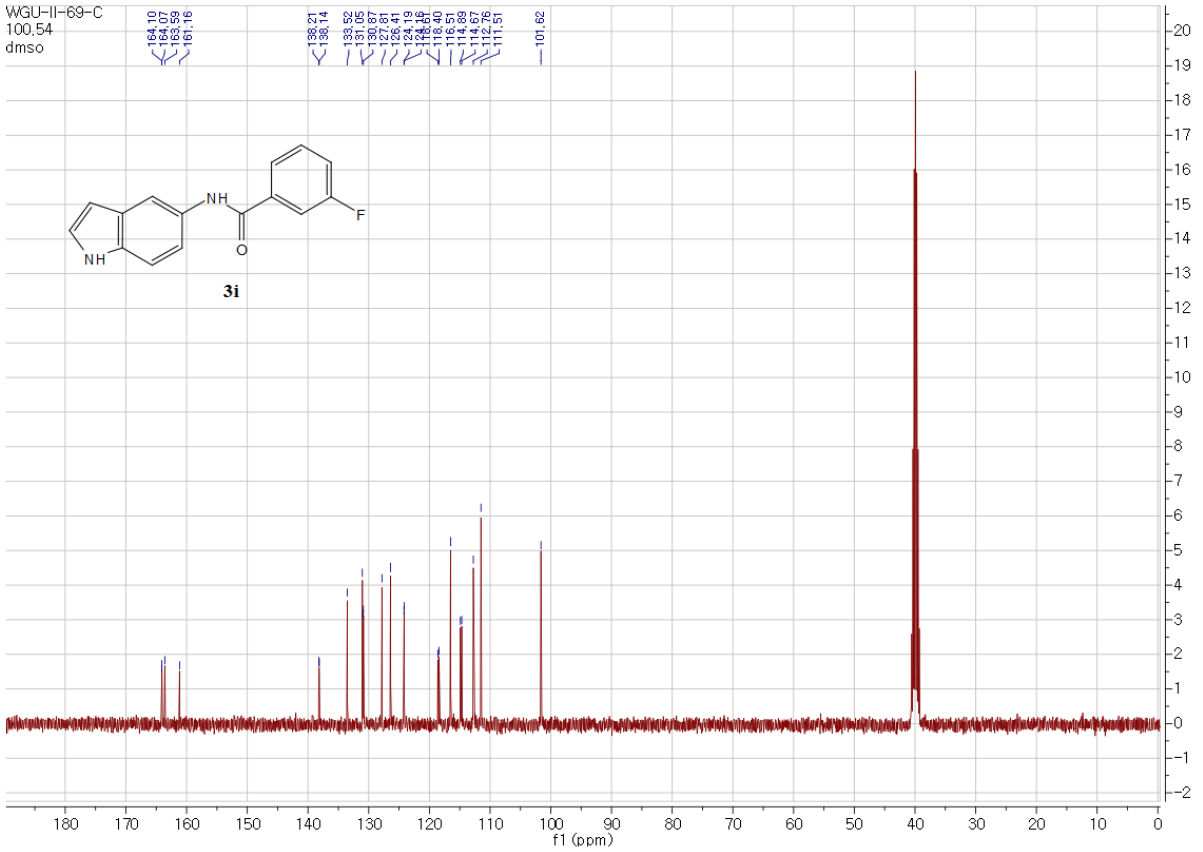
WGU-II-69-C
100.54
dmso
164.10 164.07 163.59 161.16
138.21 138.14
133.52 131.05 130.87 127.81 126.41 124.19 118.40 116.51 114.89 114.67 112.76 111.51
101.62
NH
O
F
NH
3i
f1 (ppm)

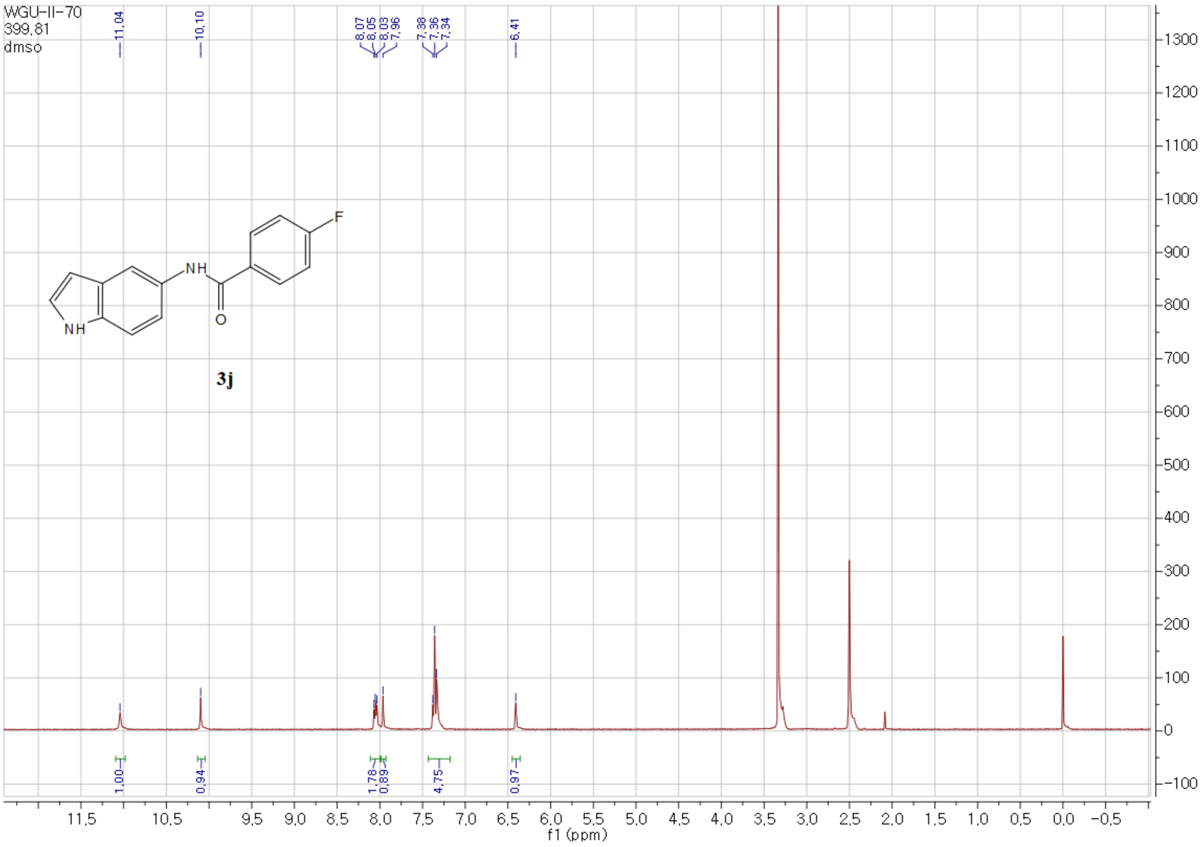
WGU-II-70
399.81
dmso
3j
f1 (ppm)

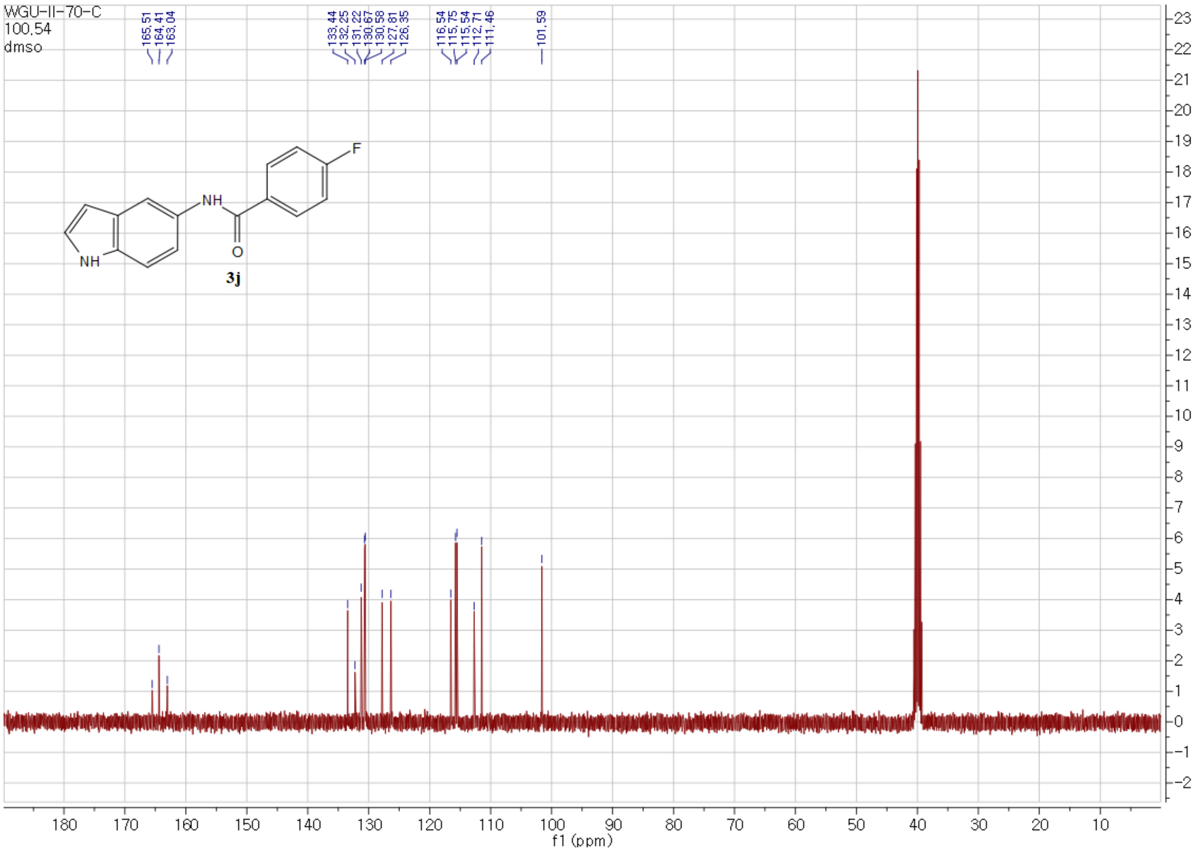
WGU-II-70-C
100.54
dmso
3j
f1 (ppm)

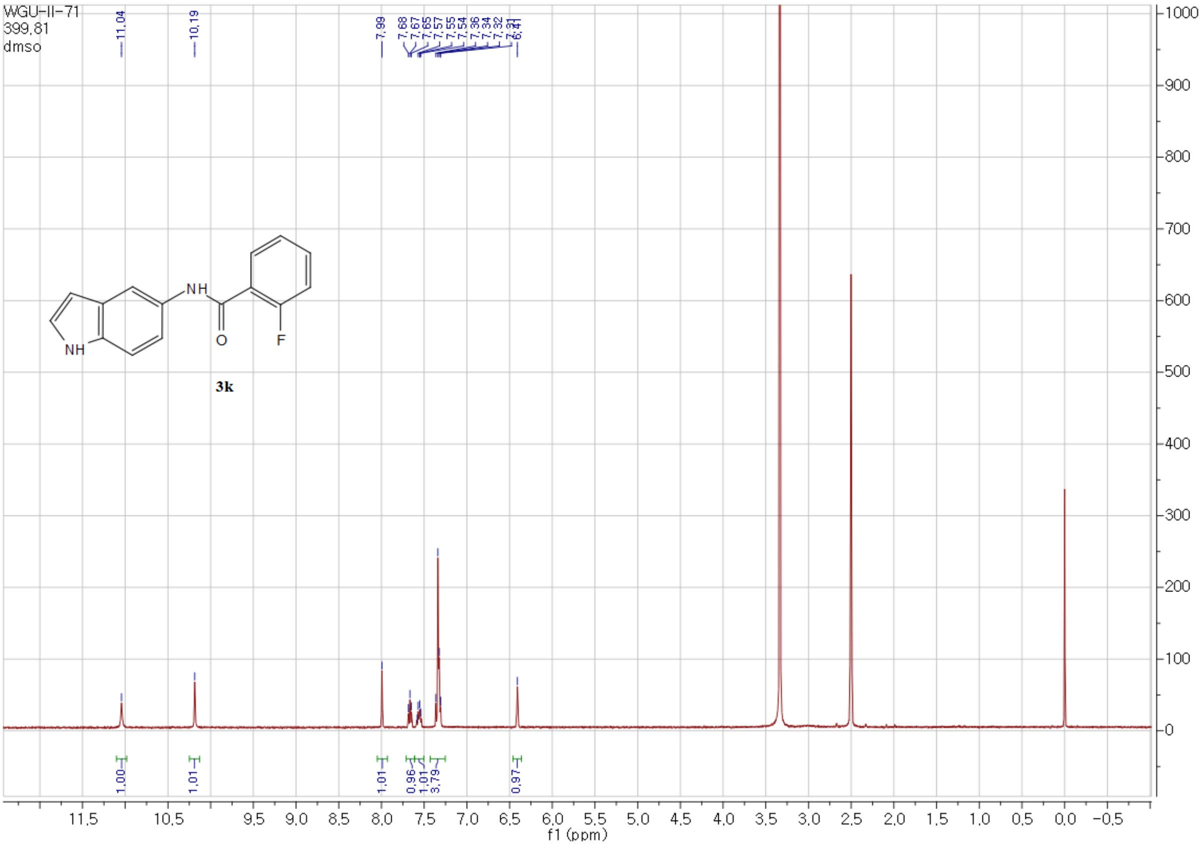
WGU-II-71
399.81
dmso
3k
f1 (ppm)

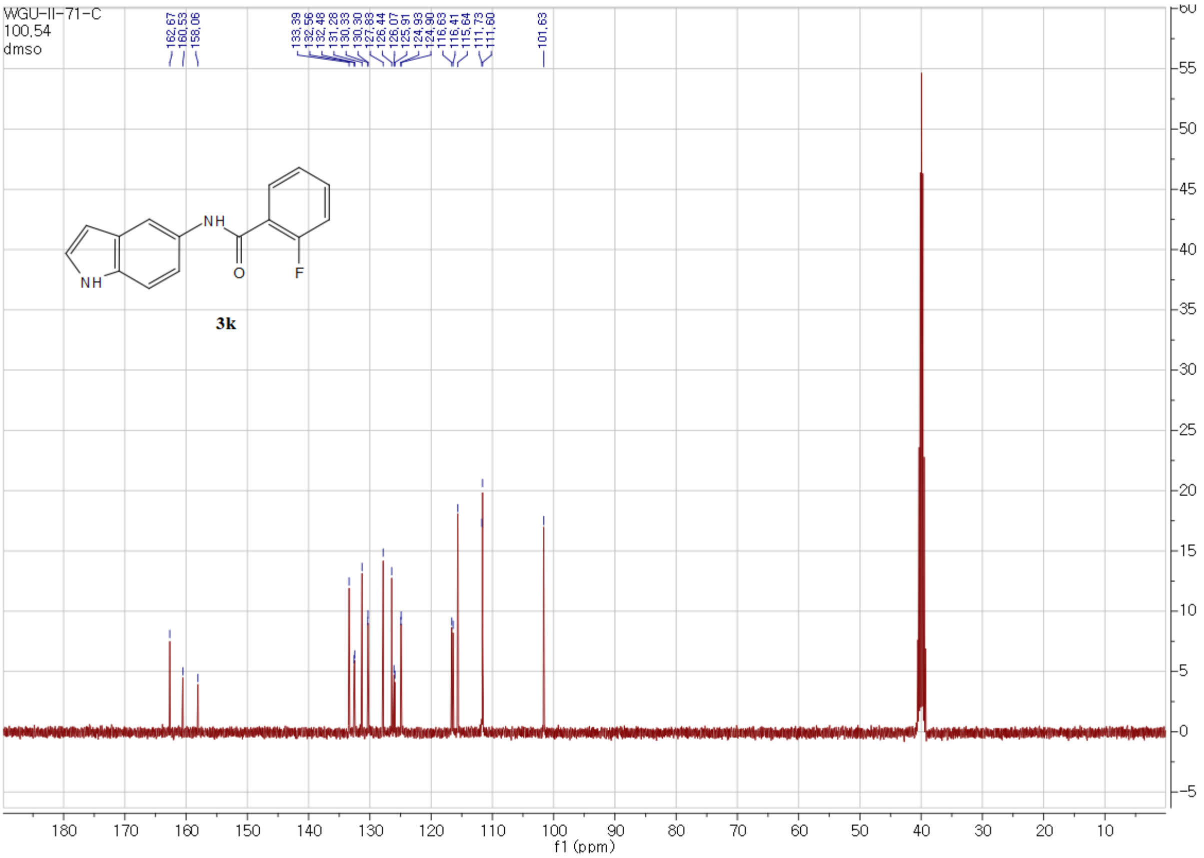
WGU-II-71-C
100.54
dmso
3k
f1 (ppm)

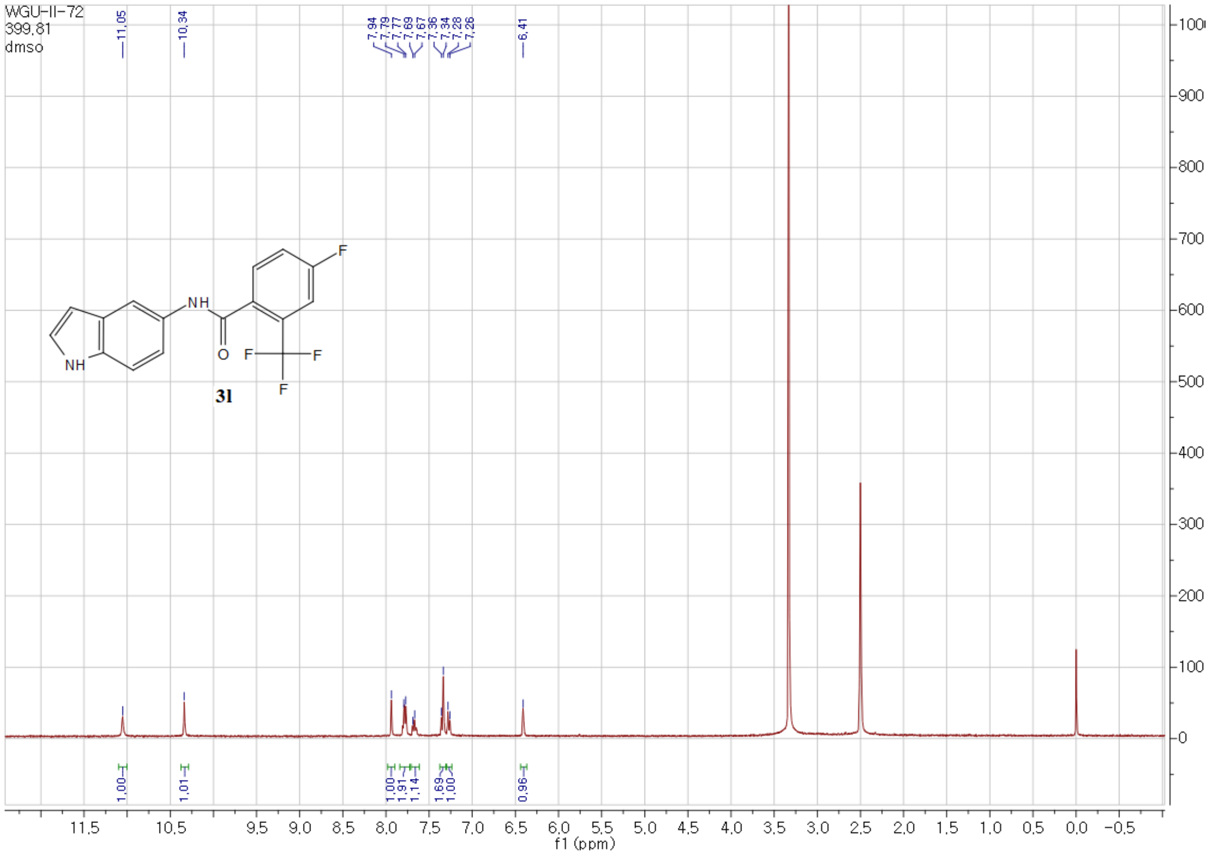
WGU-II-72
399.81
dmso
11.05
10.34
7.94
7.79
7.77
7.69
7.67
7.36
7.34
7.28
7.26
6.41
1.00
1.01
1.00
1.91
1.14
1.69
1.00
0.96
NH
O
F
NH
31
f1 (ppm)

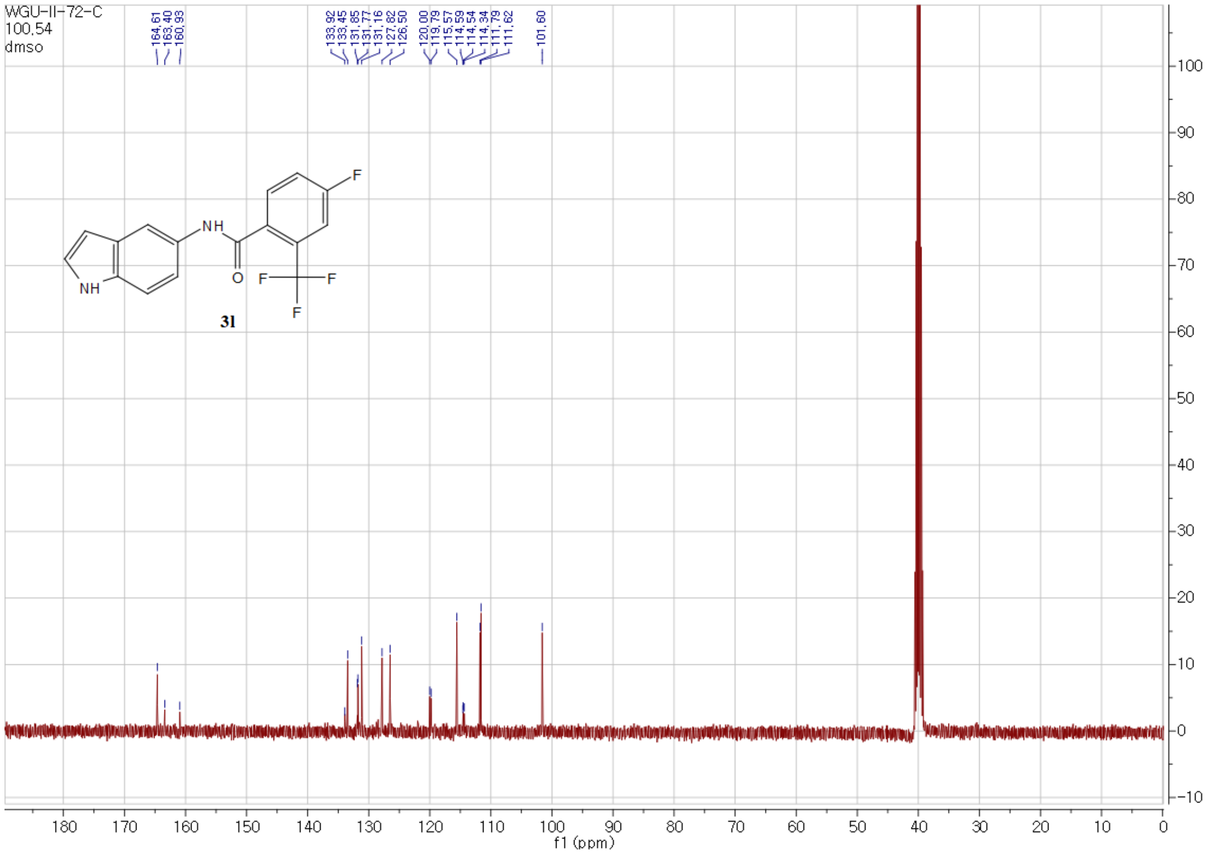
WGU-II-72-C
100.54
dmso
164.61
163.40
160.93
133.92
133.45
131.85
131.77
131.16
127.82
126.50
120.00
119.79
115.57
114.59
114.54
114.34
111.79
111.62
101.60
NH
O
F
NH
31
f1 (ppm)

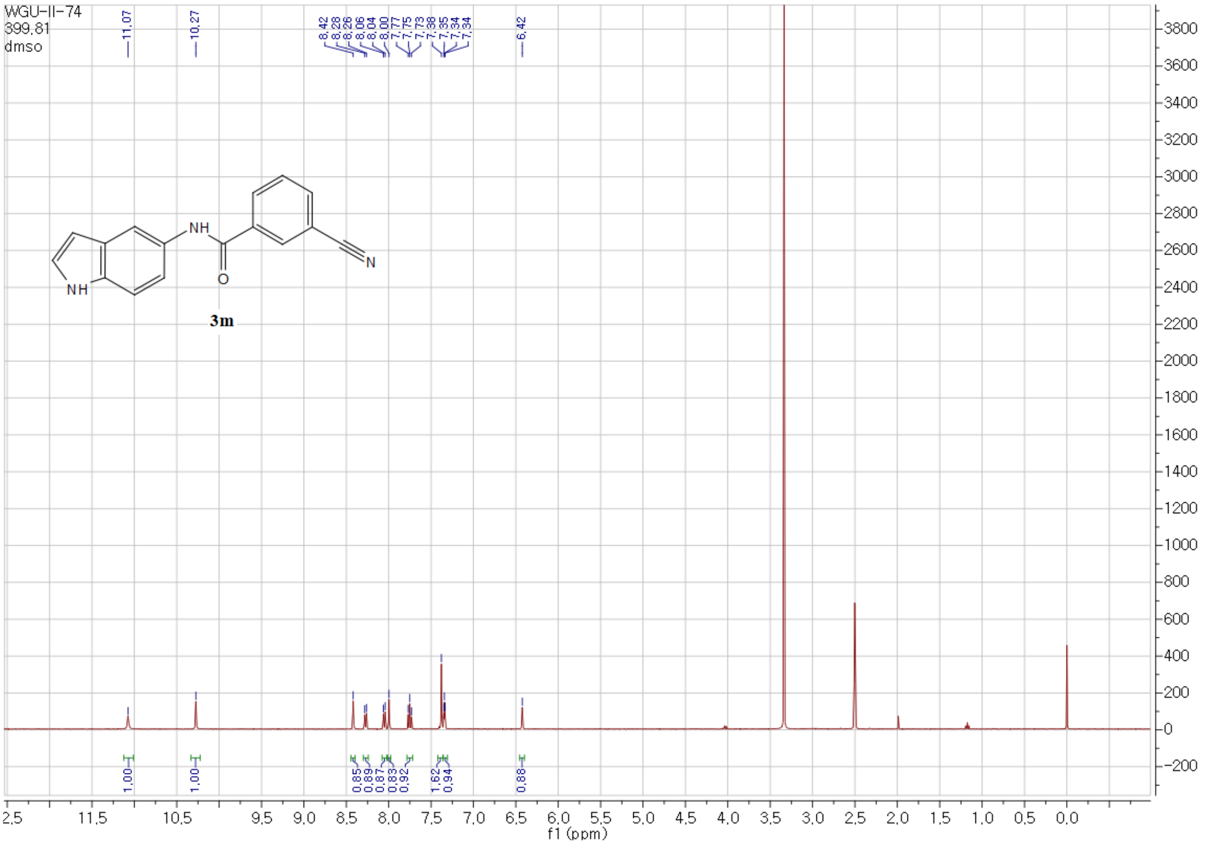
WGU-II-74
399.81
dmso
11.07
10.27
8.42
8.28
8.26
8.06
8.04
8.00
7.77
7.75
7.73
7.38
7.35
7.34
7.34
6.42
NH
NH
O
N
3m
1.00
1.00
0.85
0.89
0.87
0.83
0.92
1.62
0.94
0.88
f1 (ppm)

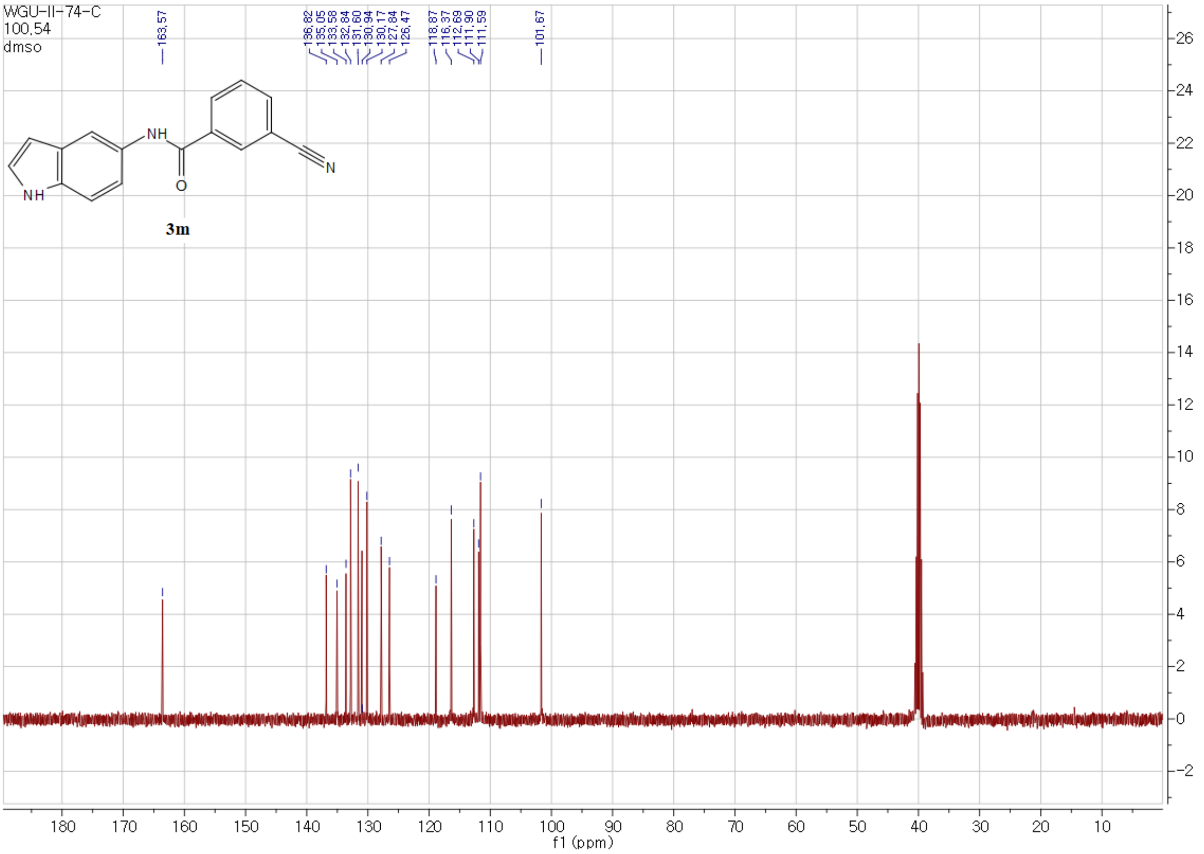
WGU-II-74-C
100.54
dmso
163.57
136.82
135.05
133.58
132.84
131.60
130.94
130.17
127.84
126.47
118.87
116.37
112.69
111.90
111.59
101.67
NH
NH
O
N
3m
f1 (ppm)

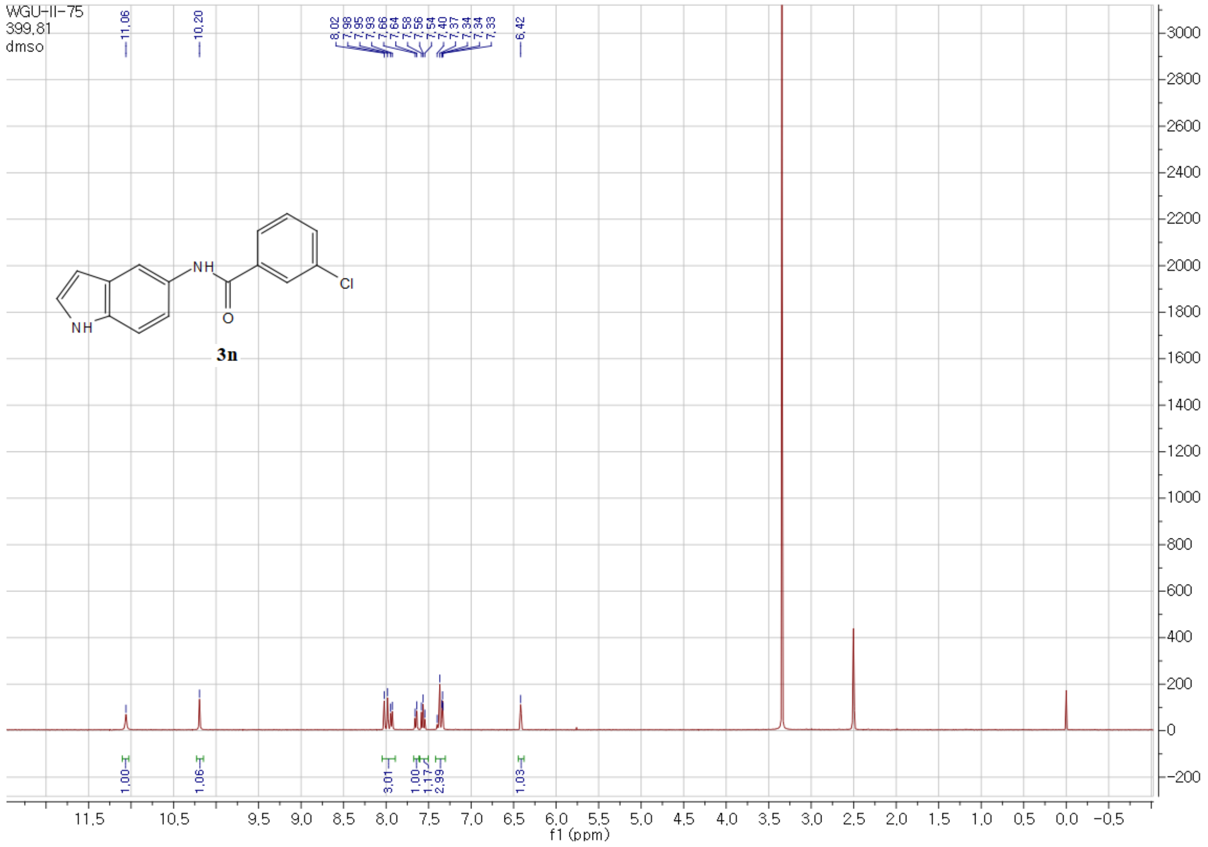
WGU-II-75
399,81
dmso
11,06
10,20
8,02
7,98
7,95
7,93
7,66
7,64
7,58
7,56
7,54
7,40
7,37
7,34
7,33
6,42
NH
Cl
O
NH
3n
1,00
1,06
3,01
1,00
1,17
2,99
1,03
f1 (ppm)

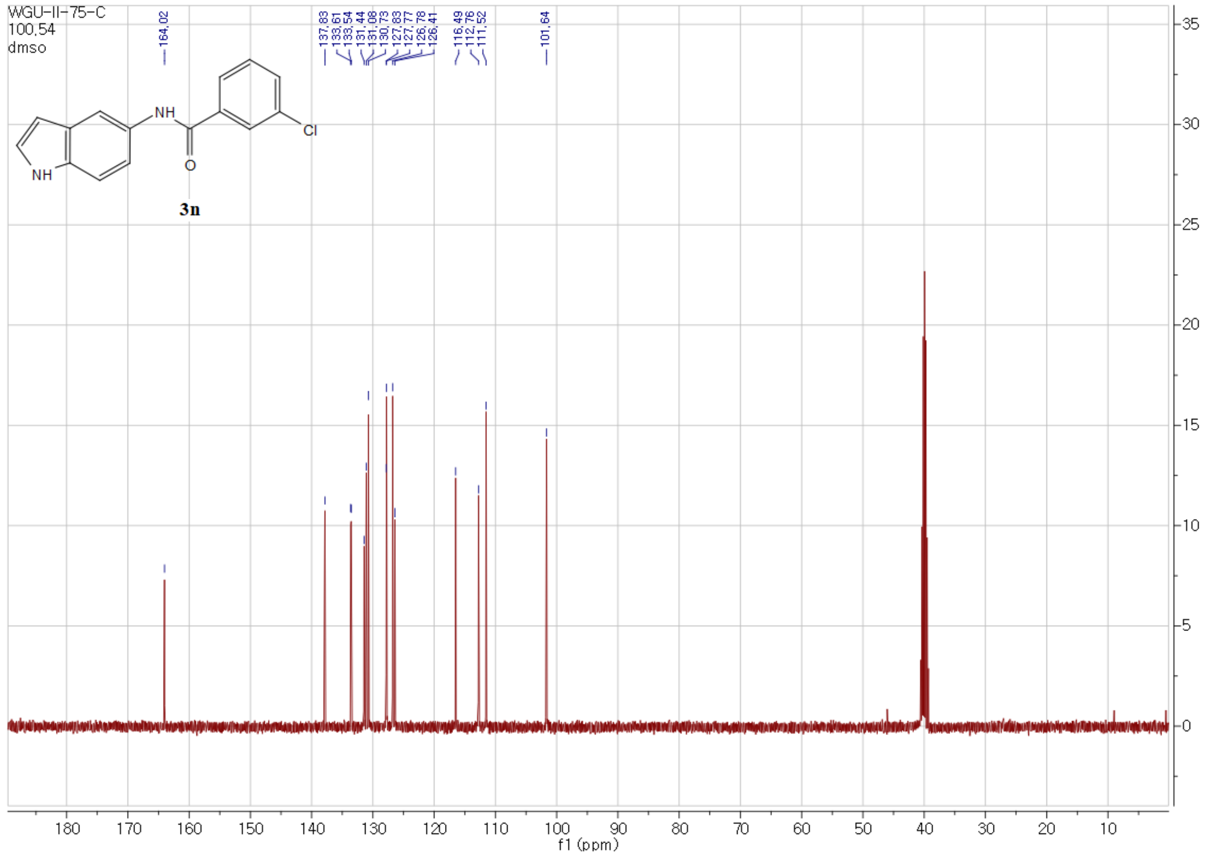
WGU-II-75-C
100,54
dmso
164,02
137,83
133,61
133,54
131,44
131,08
130,73
127,83
127,77
126,78
126,41
116,49
112,76
111,52
101,64
NH
Cl
O
NH
3n
f1 (ppm)

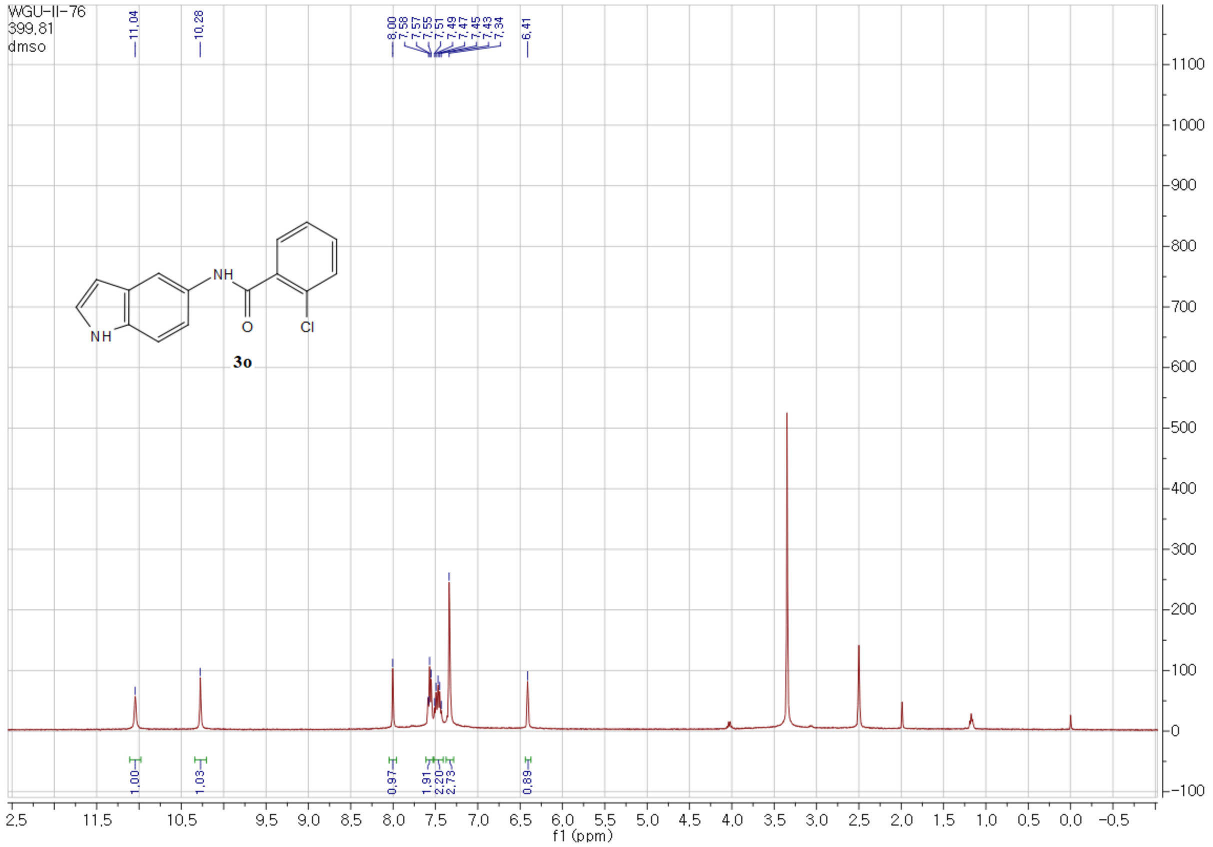
WGU-II-76
399.81
dmso
11.04
10.28
8.00
7.58
7.57
7.55
7.51
7.49
7.47
7.45
7.43
7.34
6.41
NH
NH
O
Cl
3o
1.00
1.03
0.97
1.91
2.20
2.73
0.89
f1 (ppm)

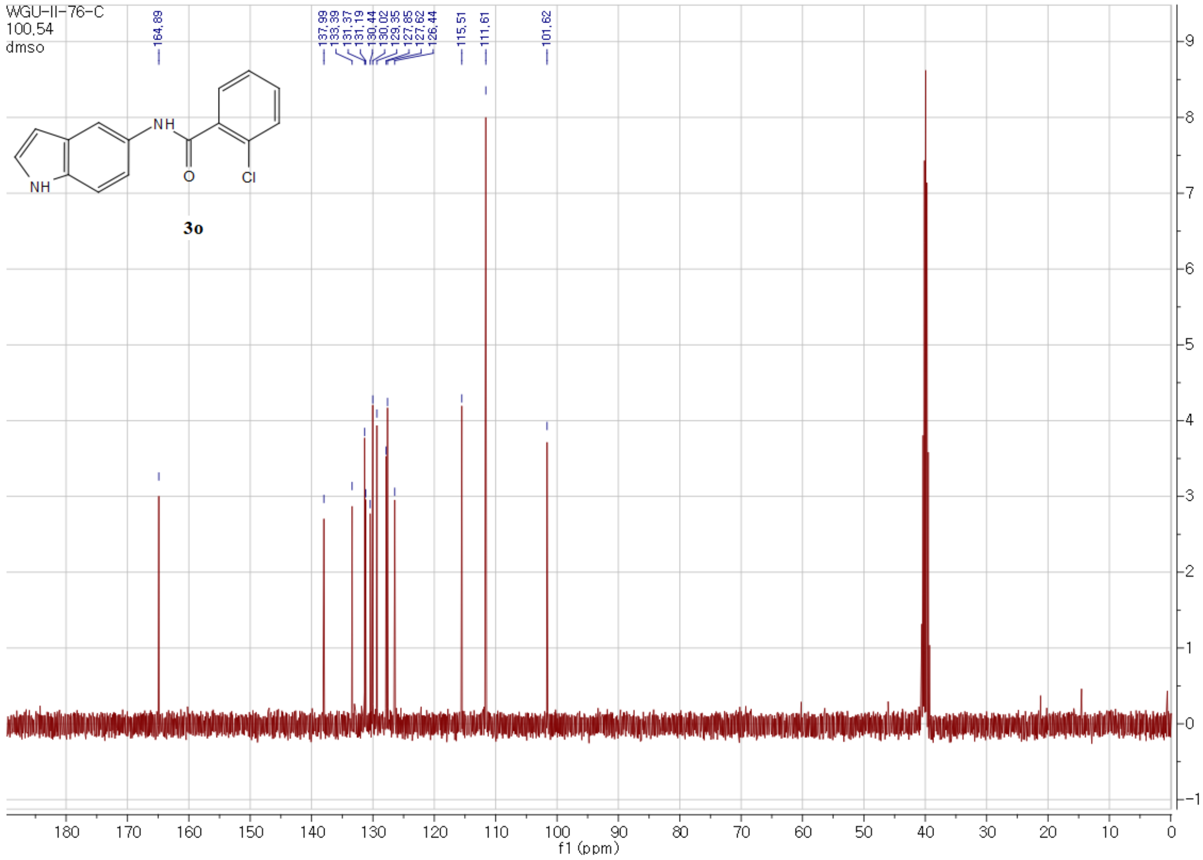
WGU-II-76-C
100.54
dmso
164.89
137.99
133.39
131.37
131.19
130.44
130.02
129.35
127.85
127.62
126.44
115.51
111.61
101.62
NH
NH
O
Cl
3o
f1 (ppm)

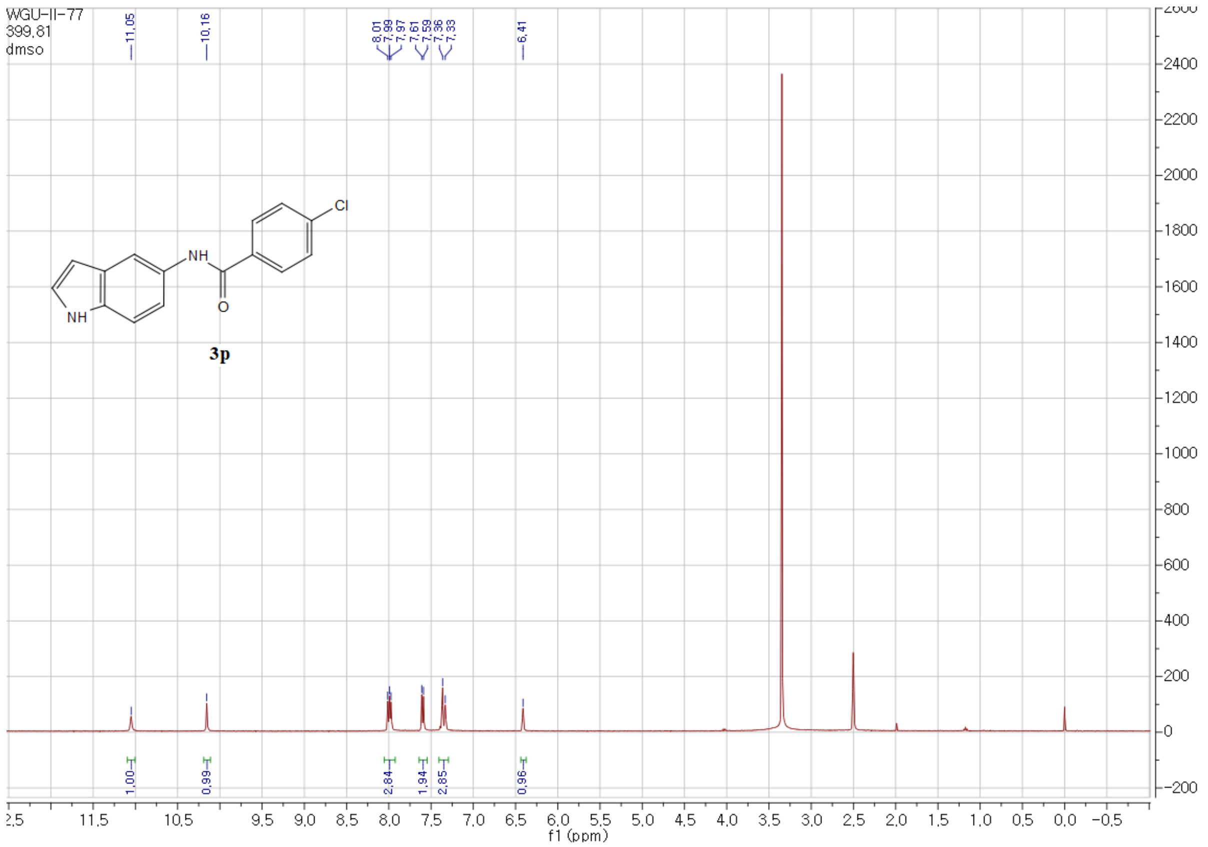
WGU-II-77
399.81
dmso
11.05
10.16
8.01
7.99
7.97
7.61
7.59
7.36
7.33
6.41
NH
Cl
NH
O
3p
1.00
0.99
2.84
1.94
2.85
0.96
f1 (ppm)

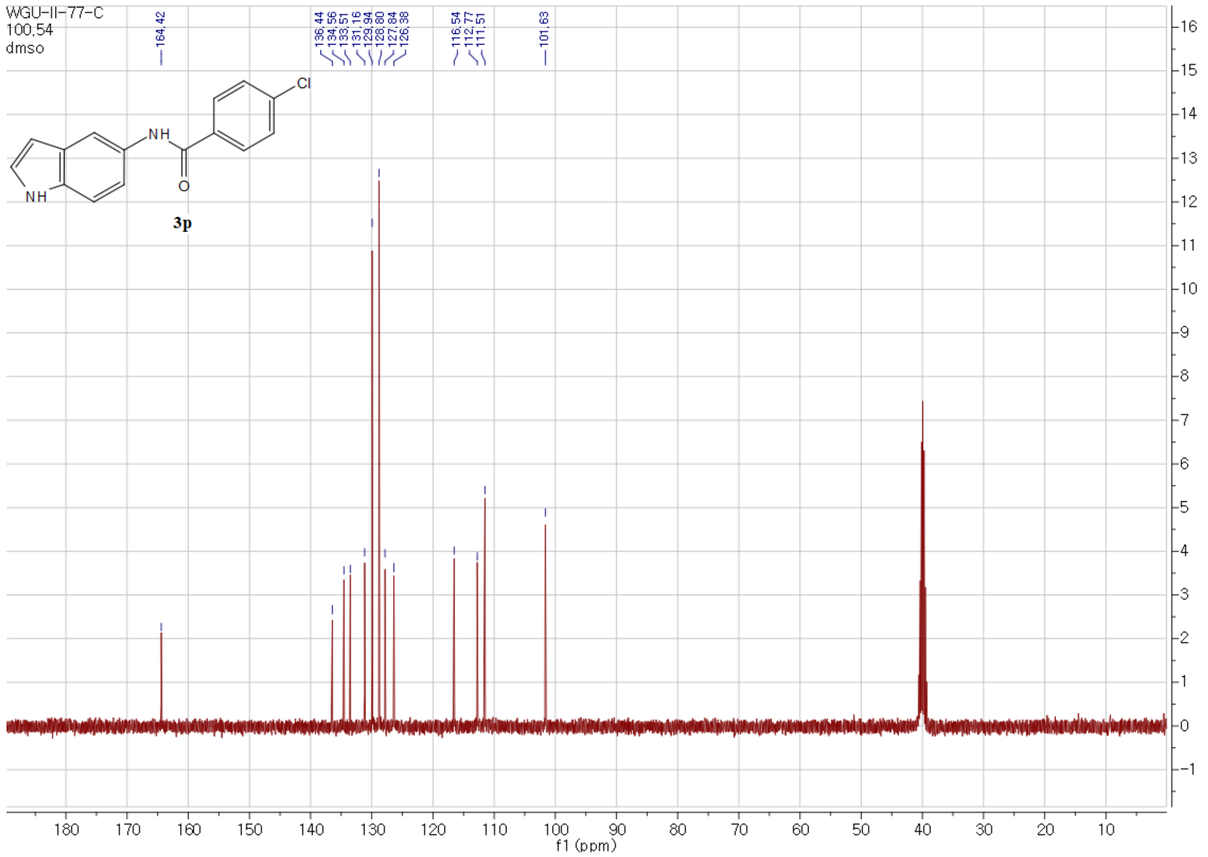
WGU-II-77-C
100.54
dmso
164.42
136.44
134.56
133.51
131.16
129.94
128.80
127.84
126.38
116.54
112.77
111.51
101.63
NH
Cl
NH
O
3p
f1 (ppm)

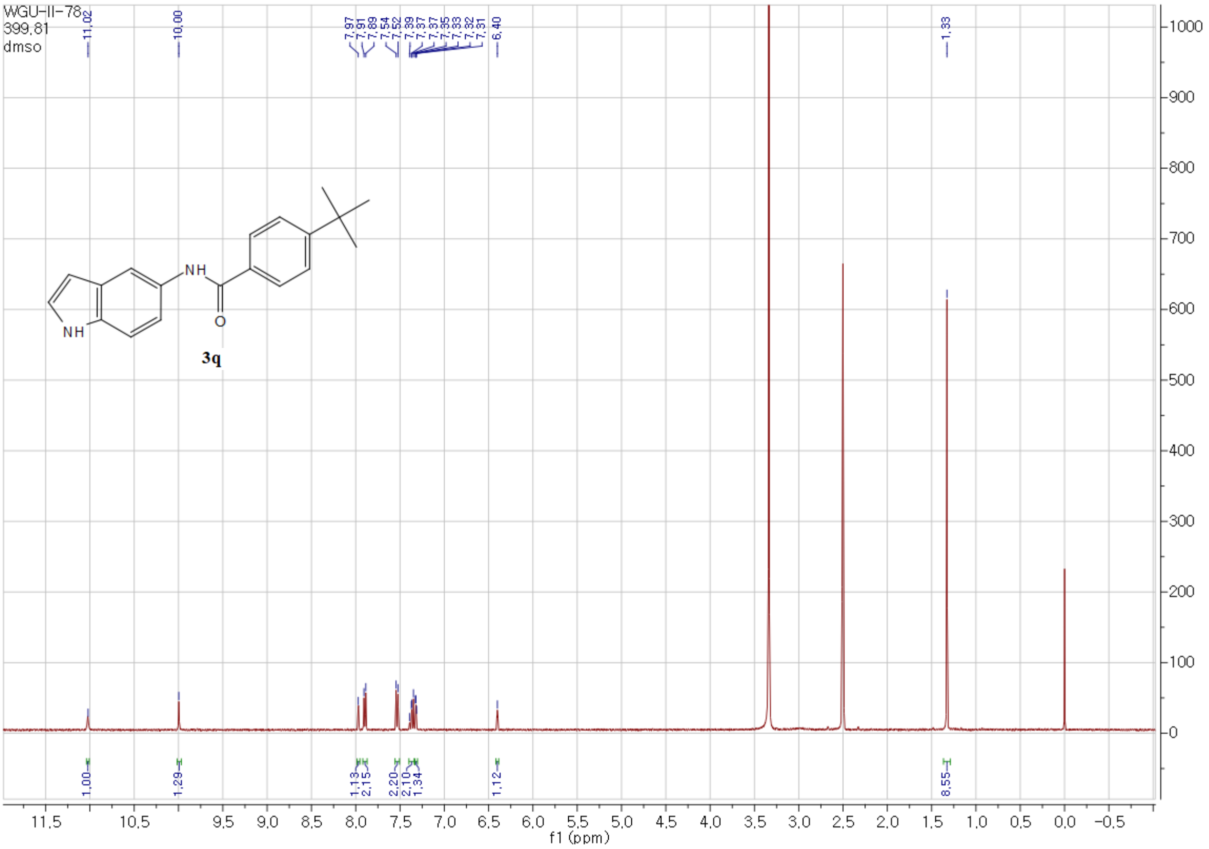
WGU-II-78
399.81
dmso
11.02
10.00
7.97 7.91 7.89 7.54 7.52 7.39 7.37 7.37 7.35 7.33 7.32 7.31
6.40
1.33
NH
NH
O
3q
1.00
1.29
1.13 2.15
2.20 2.10 1.34
1.12
8.55
f1 (ppm)

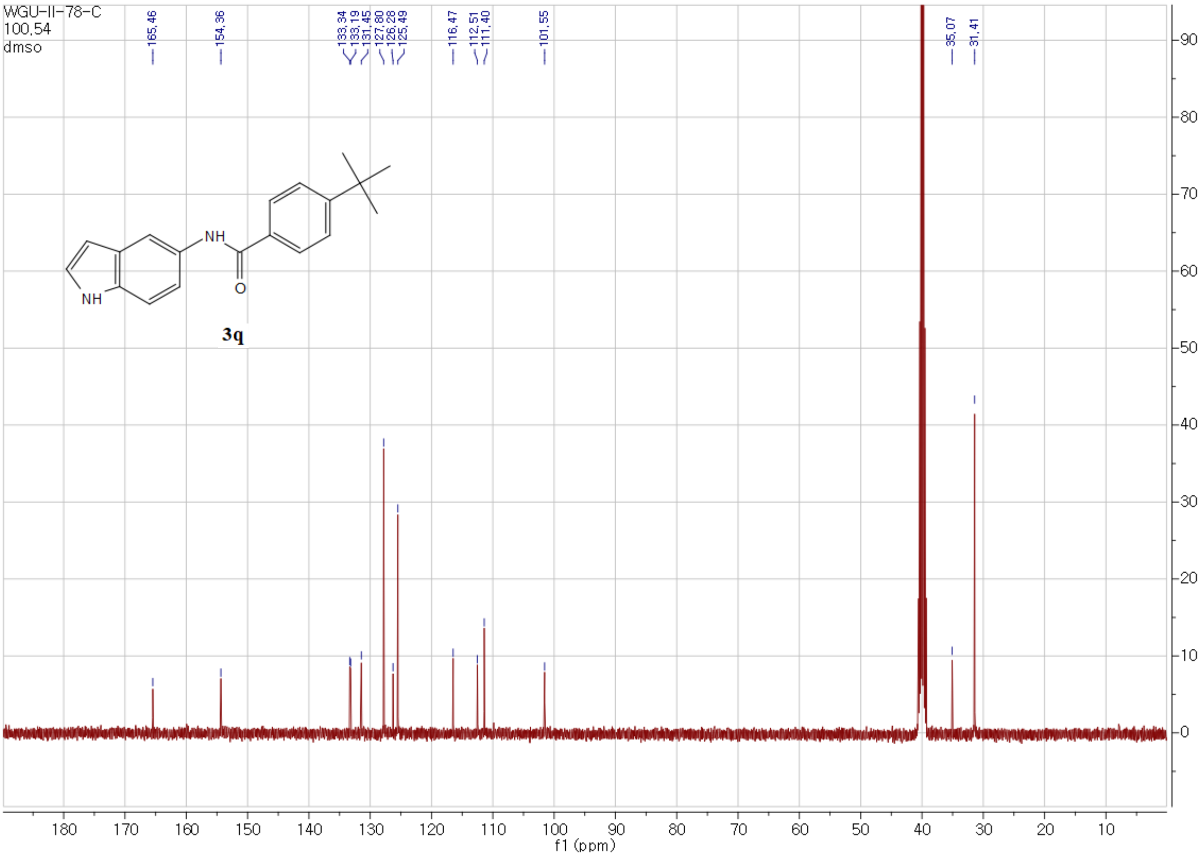
WGU-II-78-C
100.54
dmso
165.46
154.36
133.34 133.19 131.45 127.80 126.28 125.49
116.47
112.51 111.40
101.55
35.07
31.41
NH
NH
O
3q
f1 (ppm)

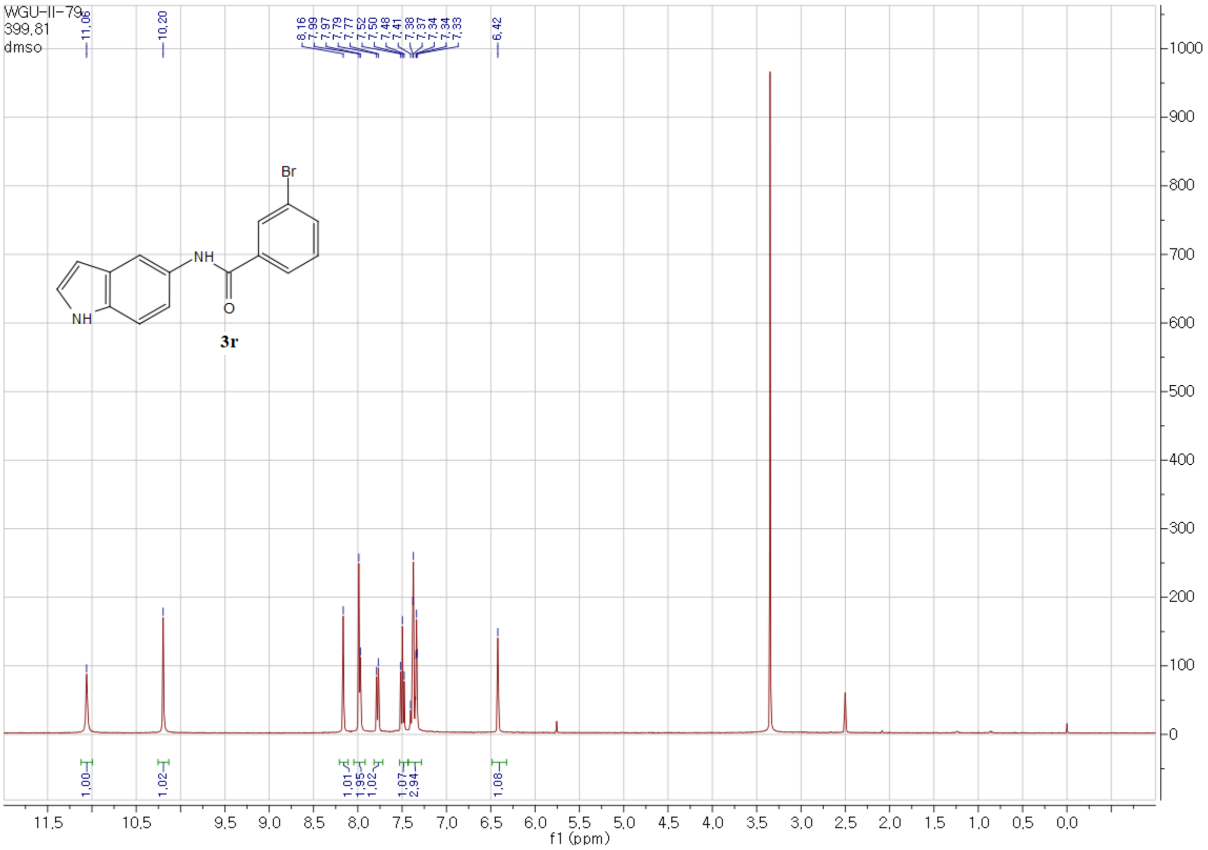
WGU-II-79
399.81
dmso
11.06
10.20
8.16 7.99 7.97 7.79 7.77 7.52 7.50 7.48 7.41 7.38 7.37 7.34 7.34 7.33
6.42
Br
NH
NH
O
1.00
1.02
1.01 1.95 1.02
1.07 2.94
1.08
f1 (ppm)

3r

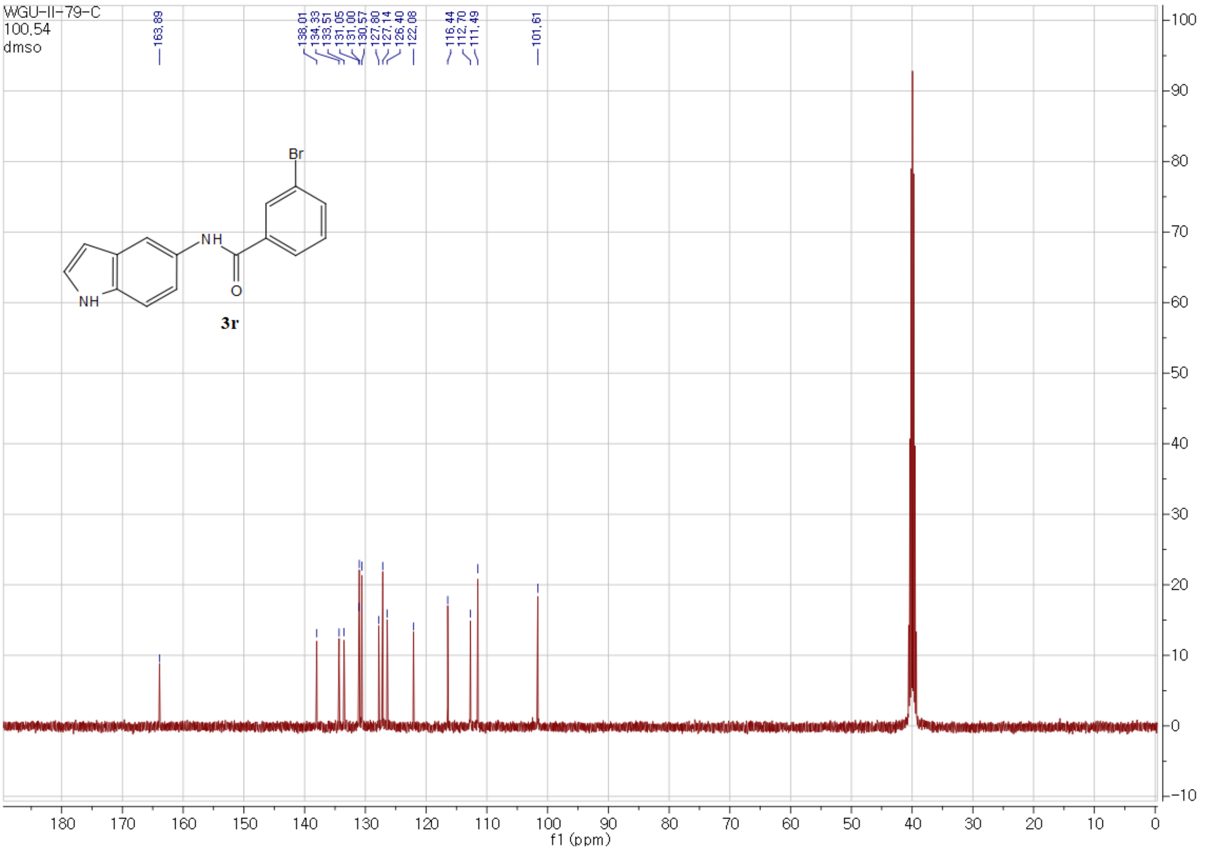
WGU-II-79-C
100.54
dmso
163.89
138.01 134.33 133.51 131.05 131.00 130.57 127.80 127.14 126.40 122.08
116.44 112.70 111.49
101.61
Br
NH
NH
O
f1 (ppm)

3r

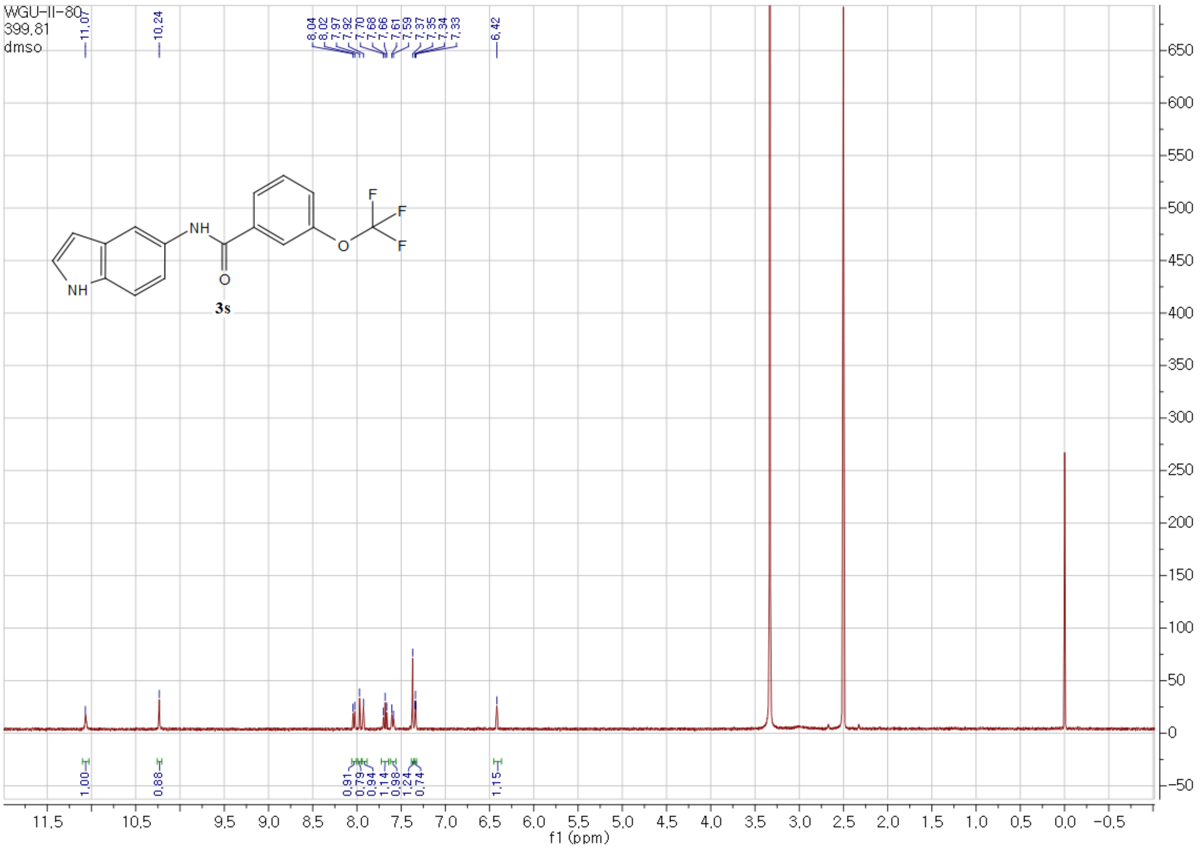
WGU-II-80
399.81
dmso
11.07
10.24
8.04 8.02 7.97 7.92 7.70 7.68 7.66 7.61 7.59 7.37 7.35 7.34 7.33
6.42
1.00 0.88 0.91 0.79 0.94 1.14 0.98 1.24 0.74 1.15
NH
O
F F F
3s
f1 (ppm)

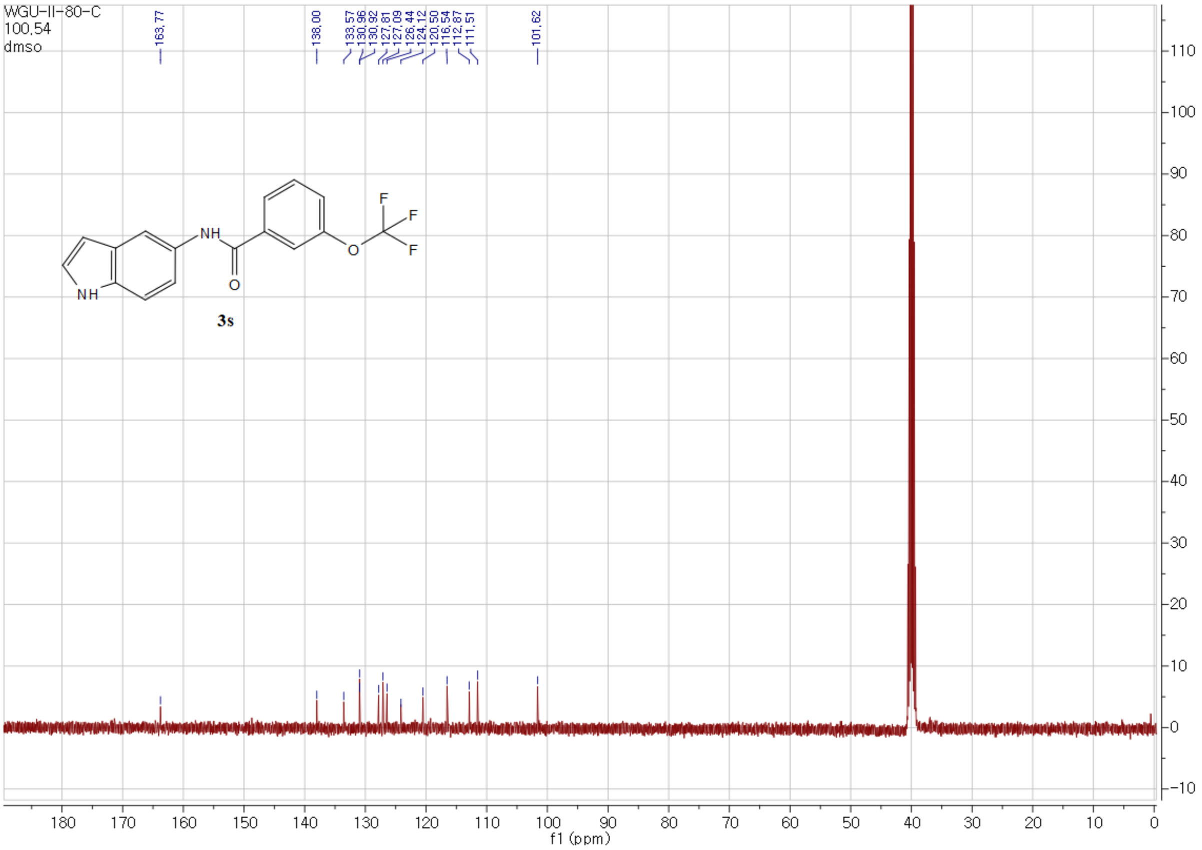
WGU-II-80-C
100.54
dmso
163.77
138.00
133.57 130.96 130.92 127.81 127.09 126.44 124.12 120.50 116.54 112.87 111.51
101.62
NH
O
F F F
3s
f1 (ppm)

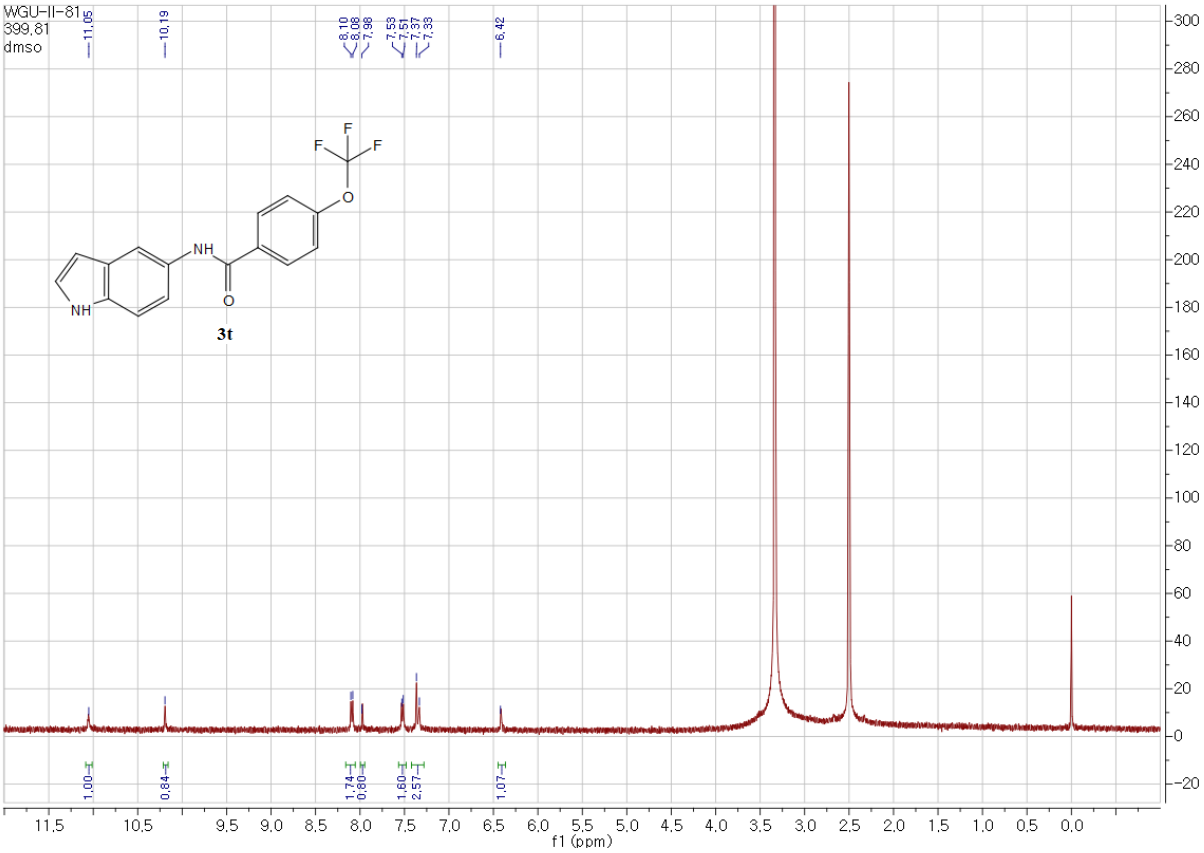
WGU-II-81
399,81
dmso
11,05
10,19
8,10
8,08
7,98
7,53
7,51
7,37
7,33
6,42
1,00
0,84
1,74
0,80
1,60
2,57
1,07
F
F
F
O
NH
O
NH
3t
f1 (ppm)

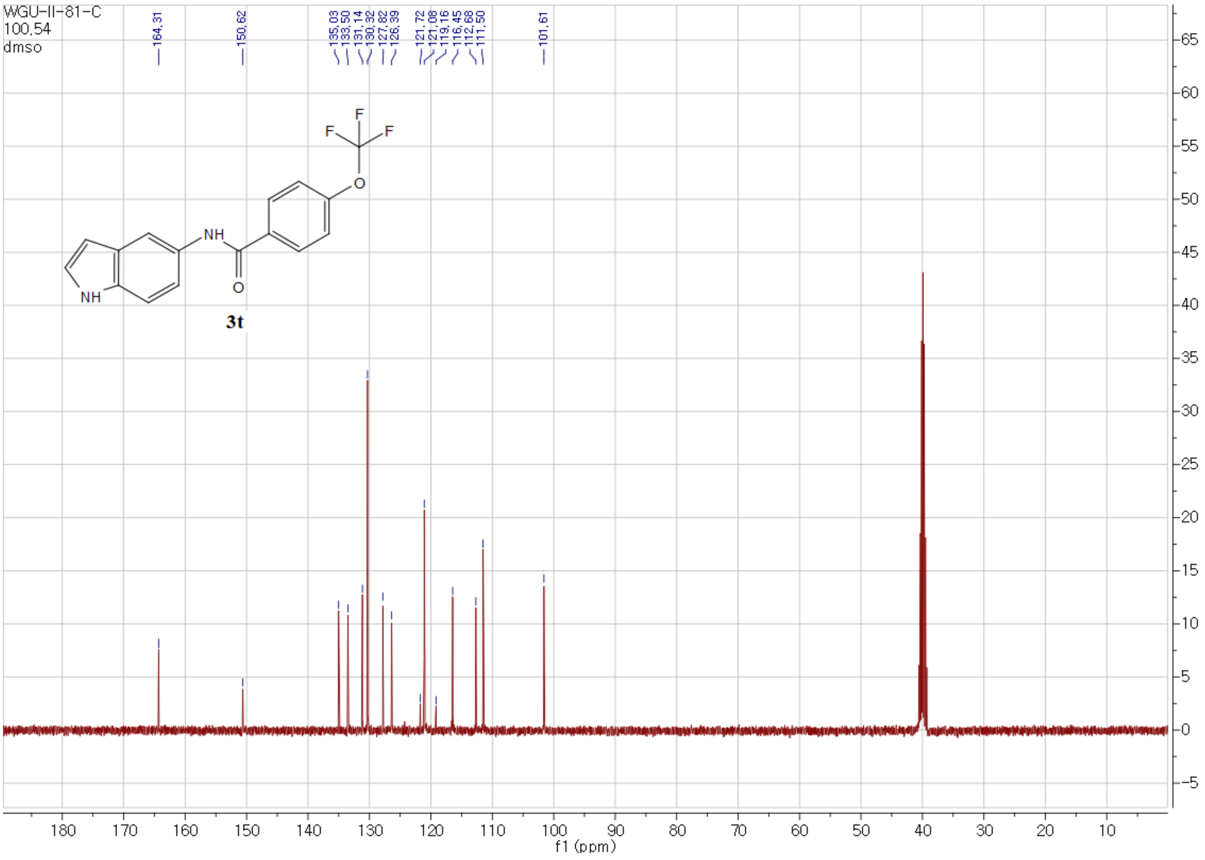
WGU-II-81-C
100,54
dmso
164,31
150,62
135,03
133,50
131,14
130,32
127,82
126,39
121,72
121,08
119,16
116,45
112,68
111,50
101,61
F
F
F
O
NH
O
NH
3t
f1 (ppm)

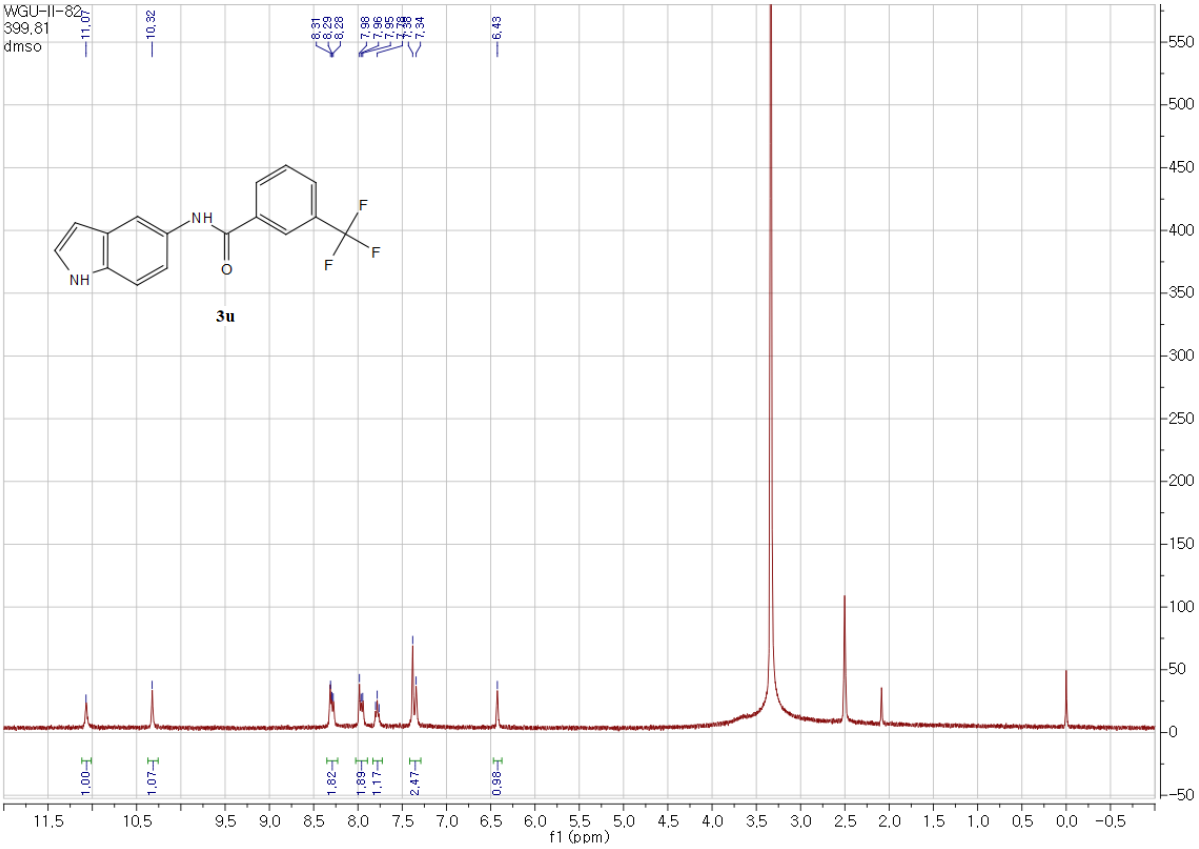
WGU-II-82
399.81
dmso
11.07
10.32
8.31
8.29
8.28
7.98
7.96
7.95
7.38
7.34
6.43
NH
F
F
F
O
NH
3u
1.00
1.07
1.82
1.89
1.17
2.47
0.98
f1 (ppm)

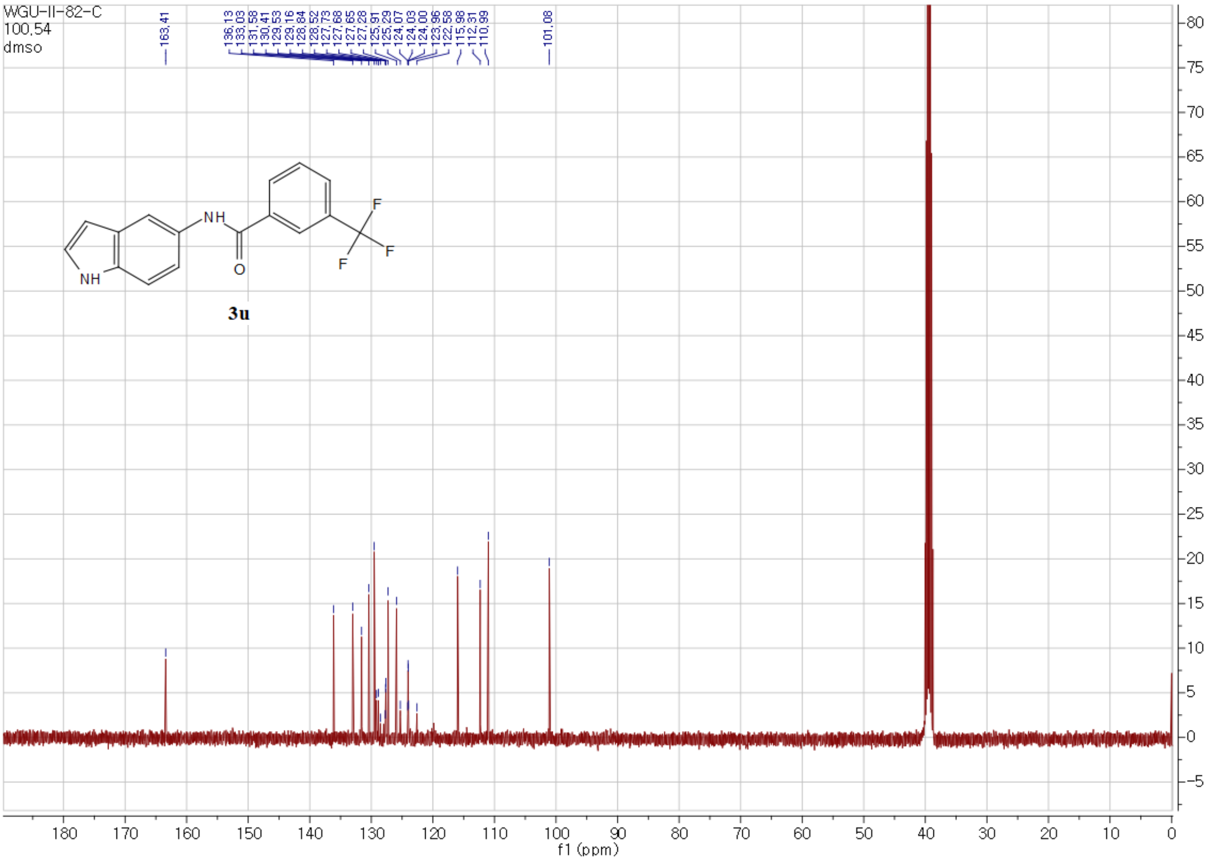
WGU-II-82-C
100.54
dmso
163.41
136.13
133.03
131.58
130.41
129.53
129.16
128.84
128.52
127.73
127.68
127.65
127.28
125.91
125.29
124.07
124.03
124.00
123.96
122.58
115.98
112.31
110.99
101.08
NH
F
F
F
O
NH
3u
f1 (ppm)

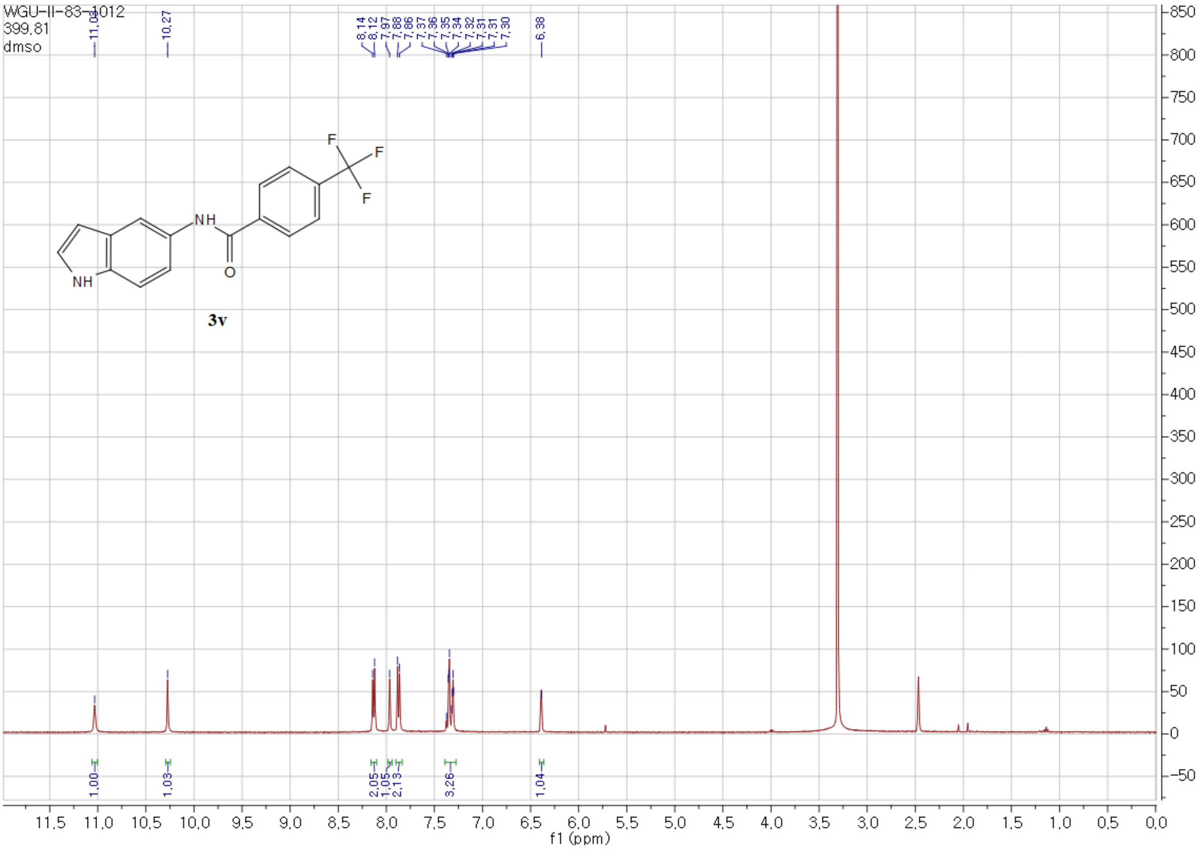
WGU-II-83-1012
399,81
dmso
11,04
10,27
8,14
8,12
7,97
7,88
7,86
7,37
7,36
7,35
7,34
7,32
7,31
7,30
6,38
1,00
1,03
2,05
1,05
2,13
3,26
1,04
f1 (ppm)
3v

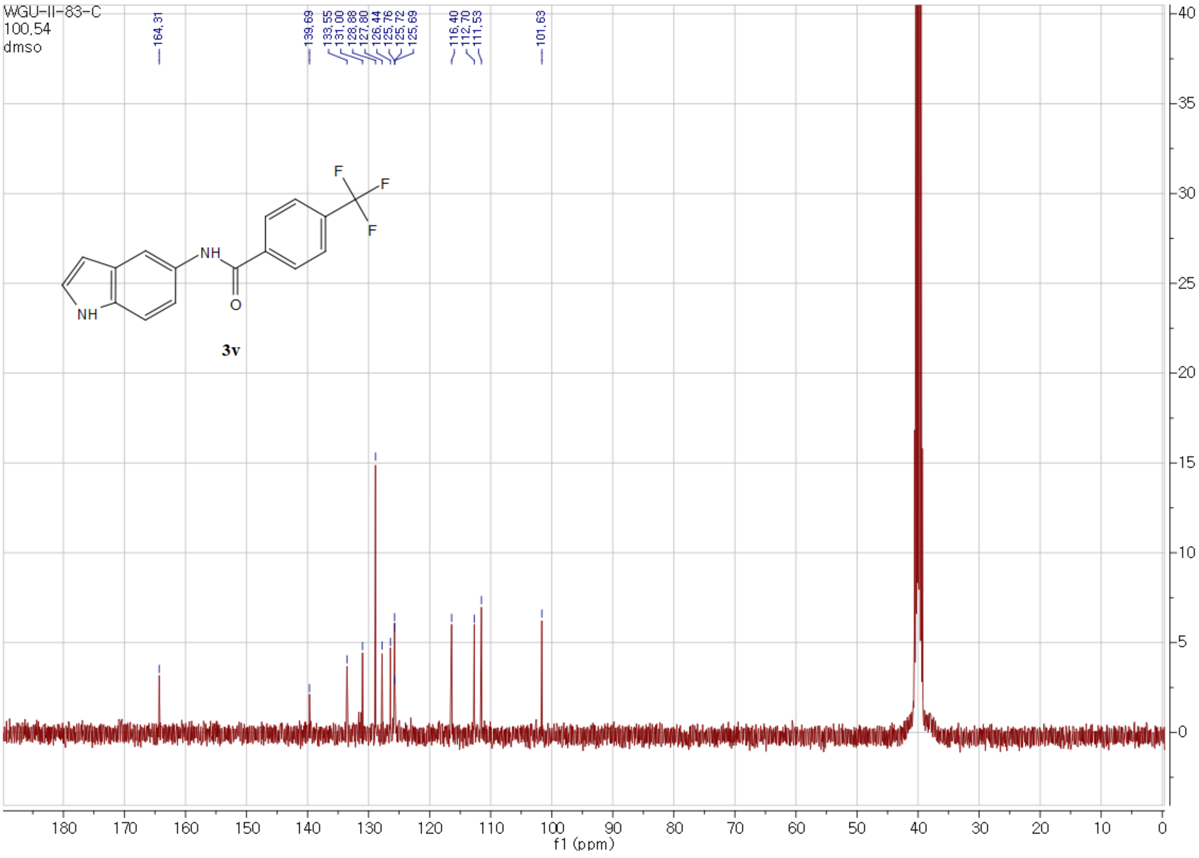
WGU-II-83-C
100,54
dmso
164,31
139,69
133,55
131,00
128,88
127,80
126,44
125,76
125,72
125,69
116,40
112,70
111,53
101,63
f1 (ppm)
3v

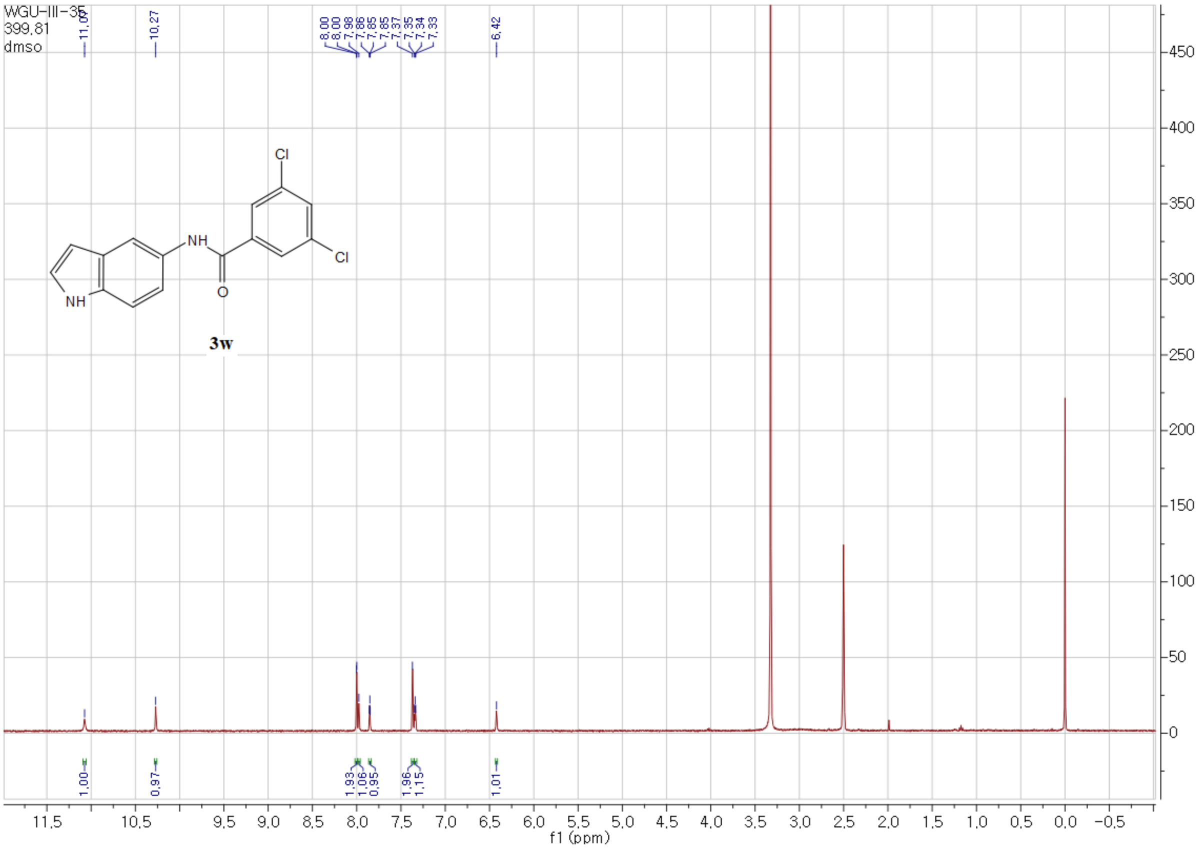

WGU-III-35
399.81
dmso
10.27
8.00
8.00
7.98
7.86
7.85
7.85
7.37
7.35
7.34
7.33
6.42
Cl
NH
Cl
NH
O
3w
1.00
0.97
1.93
1.06
0.95
1.96
1.15
1.01
f1 (ppm)

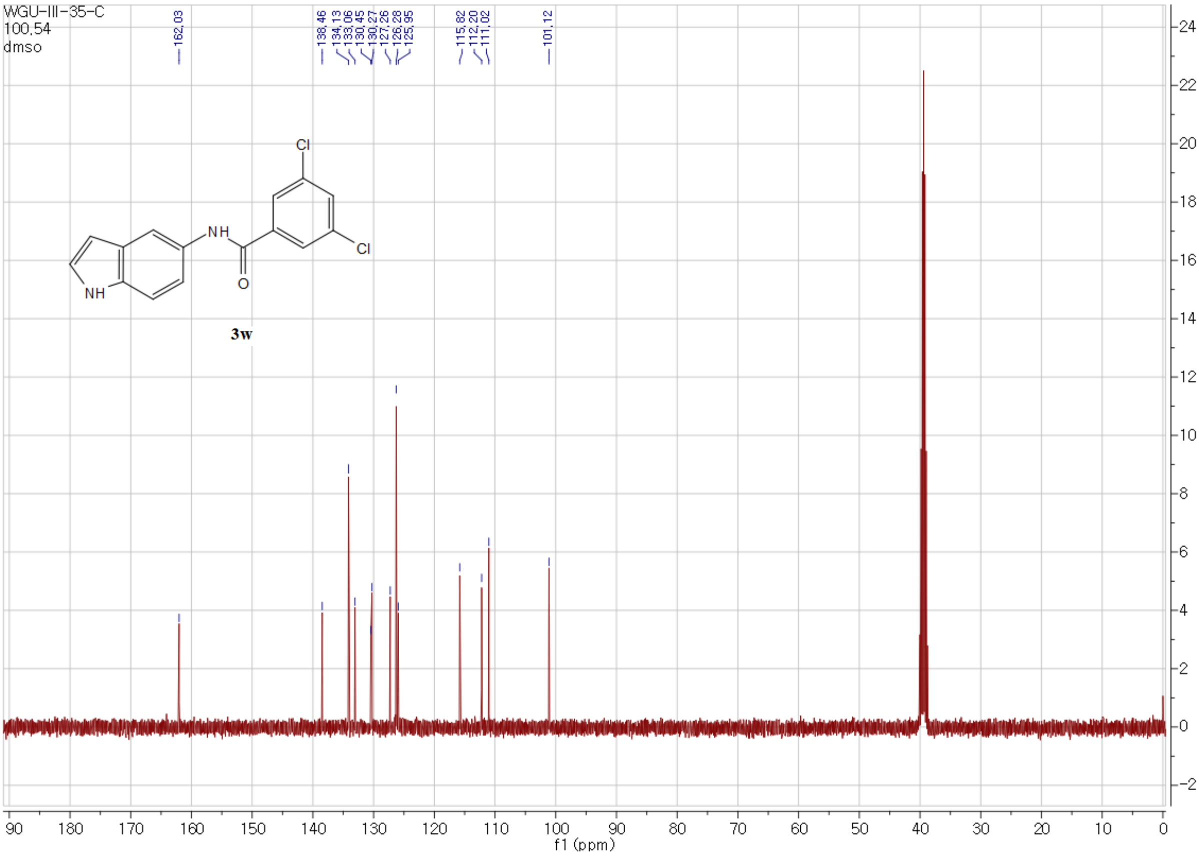

WGU-III-35-C
100.54
dmso
162.03
138.46
134.13
133.06
130.45
130.27
127.26
126.28
125.95
115.82
112.20
111.02
101.12
Cl
NH
Cl
NH
O
3w
f1 (ppm)

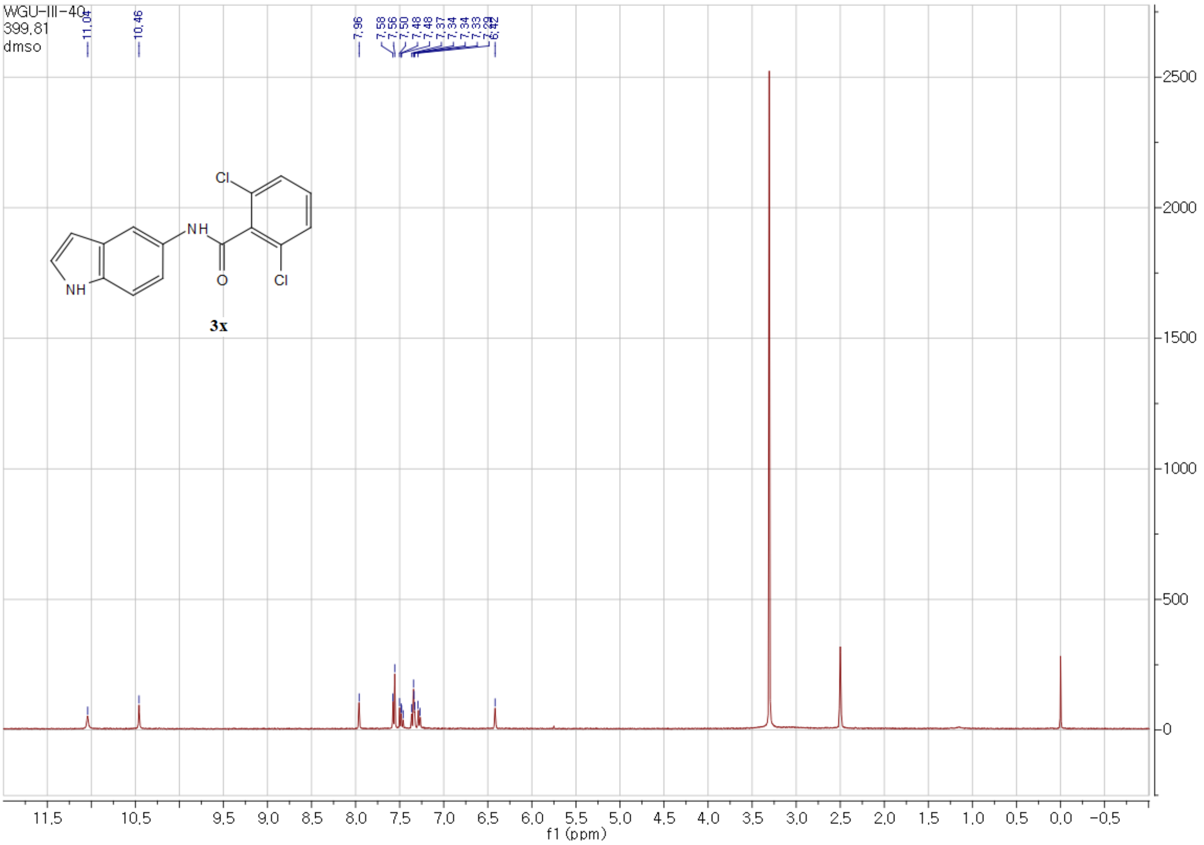
WGU-III-40
399,81
dmso
11,04
10,46
7,96
7,58
7,56
7,50
7,48
7,48
7,37
7,34
7,34
7,33
7,29
6,42
3x
f1 (ppm)

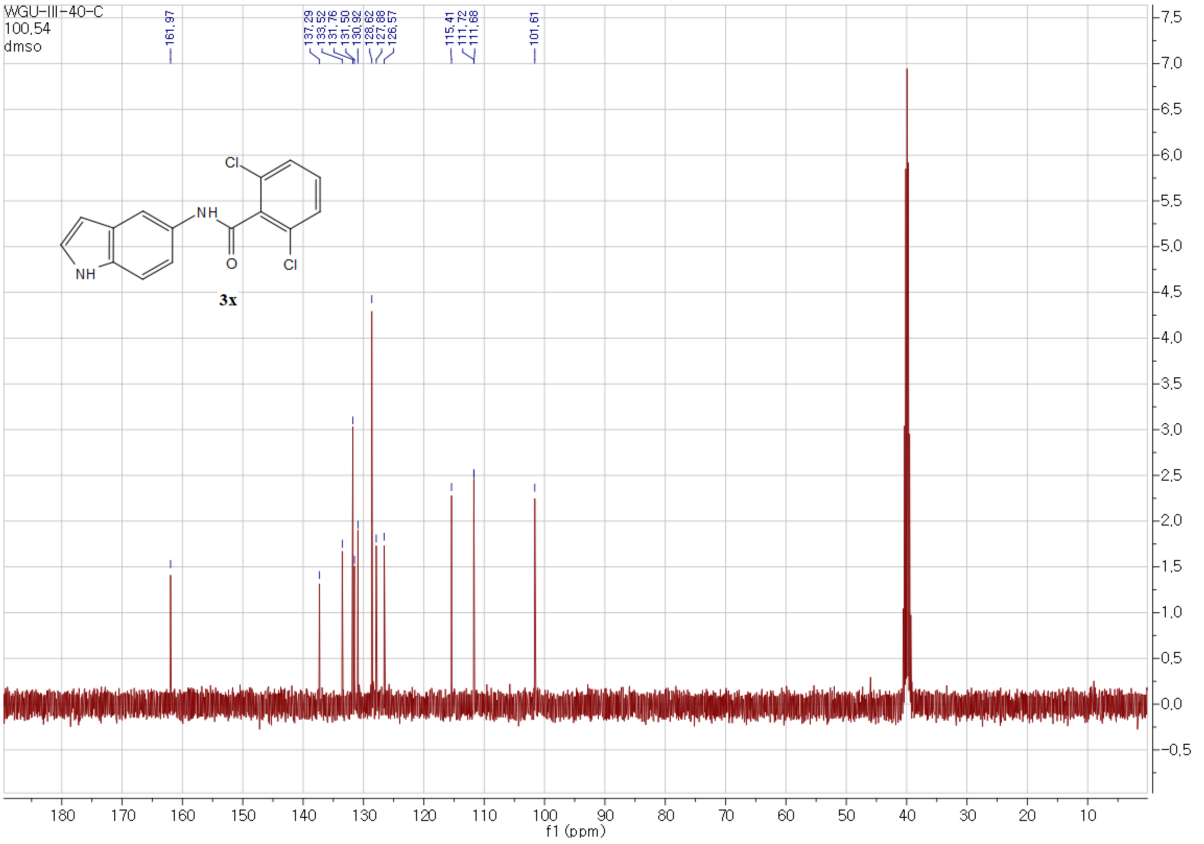
WGU-III-40-C
100,54
dmso
161,97
137,29
133,52
131,76
131,50
130,92
128,62
127,88
126,57
115,41
111,72
111,68
101,61
3x
f1 (ppm)

3. HPLC charts

	Name	Retention Time (min)	Purity1 Angle	Purity1 Threshold	PDA Match1 Spect. Name	PDA Match1 Angle	PDA Match1 Threshold	PDA Match1 Lib. Name	Area (µV*sec)	% Area	Height (µV)	Int Type	Amount
1		10.468							21369342	99.65	2667895	bb	
2		12.999							74866	0.35	8142	bb	

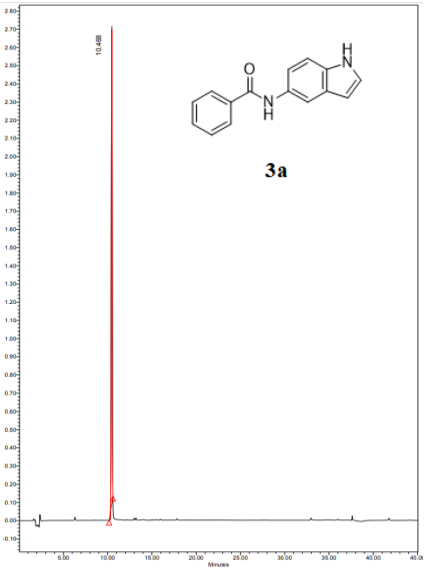

	Name	Retention Time (min)	Purity1 Angle	Purity1 Threshold	PDA Match1 Spect. Name	PDA Match1 Angle	PDA Match1 Threshold	PDA Match1 Lib. Name	Area (µV*sec)	% Area	Height (µV)	Int Type	Amount
1		7.894							372433	3.47	66153	bb	
2		11.570							10218485	95.11	1487935	bb	
3		16.582							65919	0.61	12858	bb	
4		18.601							87183	0.81	19133	bb	

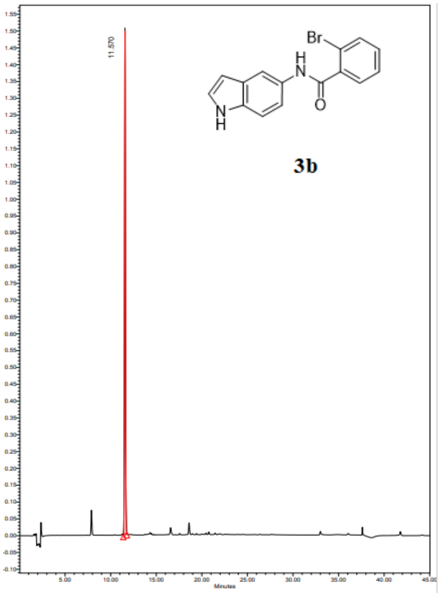

	Name	Retention Time (min)	Purity1 Angle	Purity1 Threshold	PDA Match1 Spect. Name	PDA Match1 Angle	PDA Match1 Threshold	PDA Match1 Lib. Name	Area (µV*sec)	% Area	Height (µV)	Int Type	Amount
1		10.394							51134	0.28	12275	bb	
2		13.713							22653	0.12	6132	bb	
3		14.288							18249683	99.60	2516306	bb	

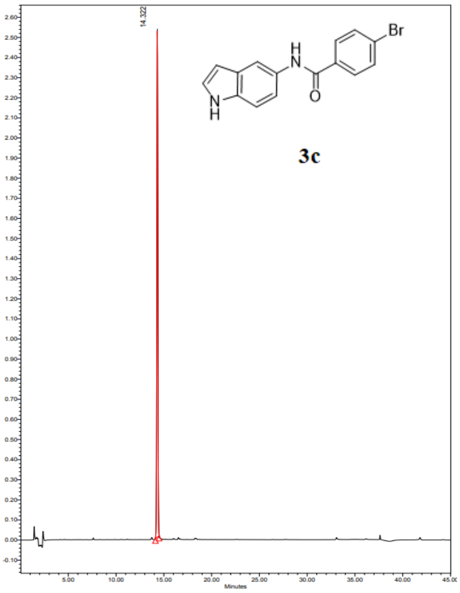

	Name	Retention Time (min)	Purity1 Angle	Purity1 Threshold	PDA Match1 Spect. Name	PDA Match1 Angle	PDA Match1 Threshold	PDA Match1 Lib. Name	Area (µV*sec)	% Area	Height (µV)	Int Type	Amount
1		7.622							26515	0.14	7036	bb	
2		13.735							14448	0.08	3609	bb	
3		14.322							18816968	99.78	2501866	bb	

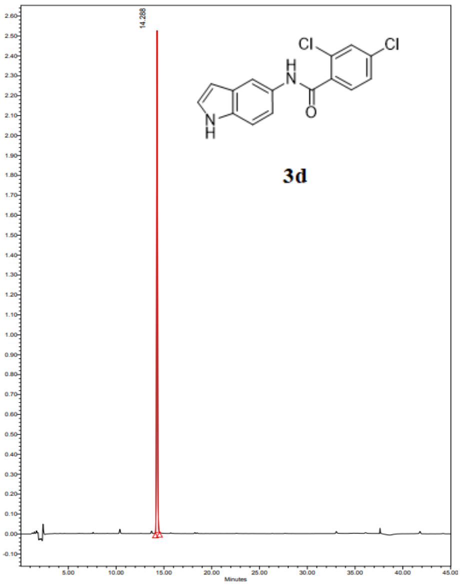

	Name	Retention Time (min)	Purity1 Angle	Purity1 Threshold	PDA Match1 Spect. Name	PDA Match1 Angle	PDA Match1 Threshold	PDA Match1 Lib. Name	Area (µV*sec)	% Area	Height (µV)	Int Type	Amount
1		9.583							142467	0.92	23767	bb	
2		14.529							15290432	99.08	1694714	bb	

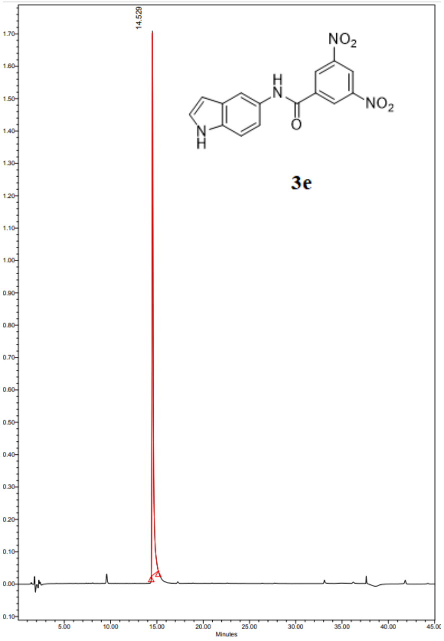

	Name	Retention Time (min)	Purity1 Angle	Purity1 Threshold	PDA Match1 Spect. Name	PDA Match1 Angle	PDA Match1 Threshold	PDA Match1 Lib. Name	Area (µV*sec)	% Area	Height (µV)	Int Type	Amount
1		7.391							117104	0.43	21760	bb	
2		12.240							26980267	99.57	2433709	bb	

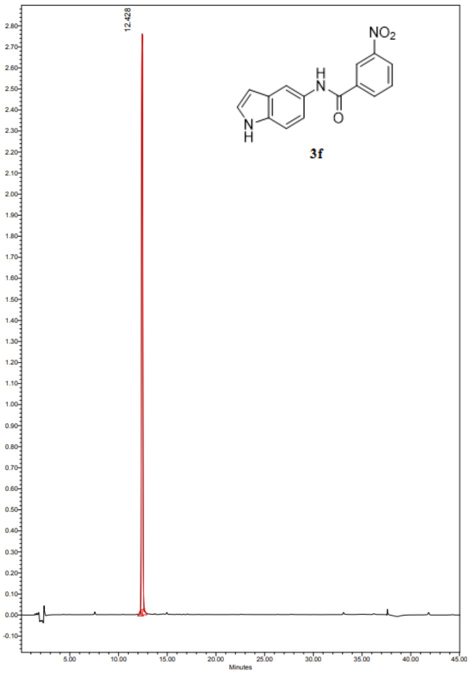

	Name	Retention Time (min)	Purity1 Angle	Purity1 Threshold	PDA Match1 Spect. Name	PDA Match1 Angle	PDA Match1 Threshold	PDA Match1 Lib. Name	Area (µV*sec)	% Area	Height (µV)	Int Type	Amount
1		7.988							9603	0.04	2618	bb	
2		12.492							22455206	99.96	2734281	bb	

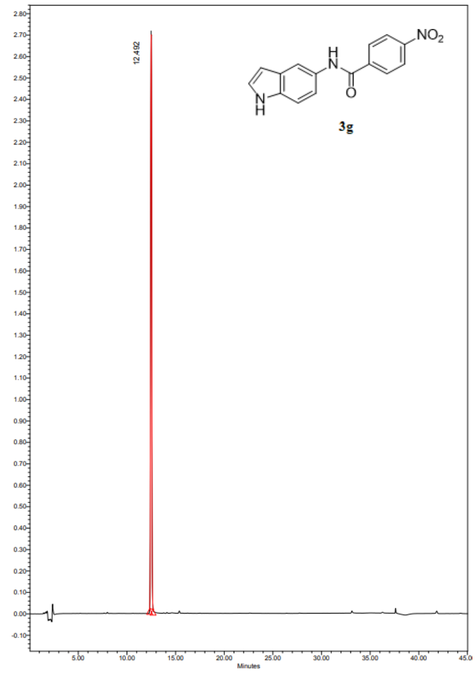

	Name	Retention Time (min)	Purity1 Angle	Purity1 Threshold	PDA Match1 Spect. Name	PDA Match1 Angle	PDA Match1 Threshold	PDA Match1 Lib. Name	Area (µV*sec)	% Area	Height (µV)	Int Type	Amount
1		10.743							28109667	100.00	2491791	bb	

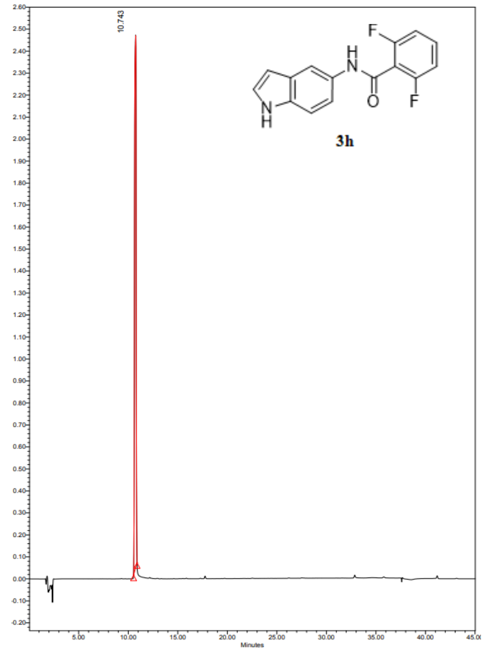

	Name	Retention Time (min)	Purity1 Angle	Purity1 Threshold	PDA Match1 Spect. Name	PDA Match1 Angle	PDA Match1 Threshold	PDA Match1 Lib. Name	Area (µV*sec)	% Area	Height (µV)	Int Type	Amount
1		11.445							4063	0.02	848	bb	
2		11.831							18559470	99.94	2423051	bb	
3		14.755							7772	0.04	2766	bb	

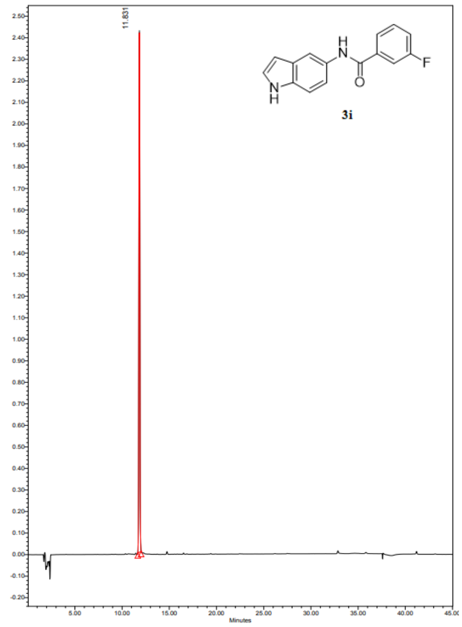

	Name	Retention Time (min)	Purity1 Angle	Purity1 Threshold	PDA Match1 Spect. Name	PDA Match1 Angle	PDA Match1 Threshold	PDA Match1 Lib. Name	Area (µV*sec)	% Area	Height (µV)	Int Type	Amount
1		10.993							4330	0.02	992	bb	
2		11.486							23117939	99.27	2536209	bb	
3		12.155							105518	0.45	8055	bb	
4		14.499							60075	0.26	11099	bb	

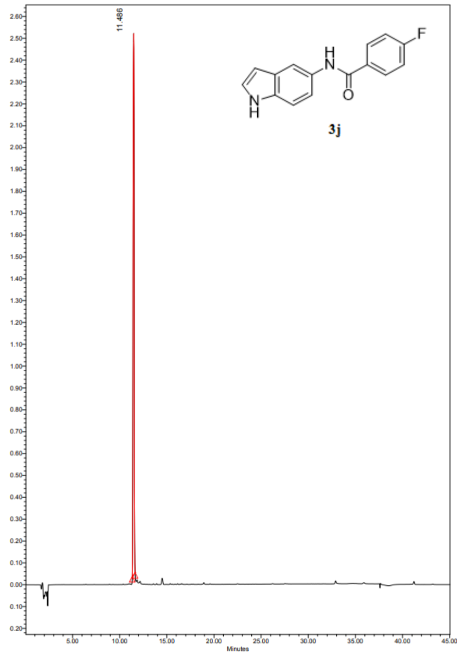

	Name	Retention Time (min)	Purity1 Angle	Purity1 Threshold	PDA Match1 Spect. Name	PDA Match1 Angle	PDA Match1 Threshold	PDA Match1 Lib. Name	Area (μV*sec)	% Area	Height (μV)	Int Type	Amount
1		11.214							25516432	99.98	2505335	bb	
2		11.866							5432	0.02	1299	bb	

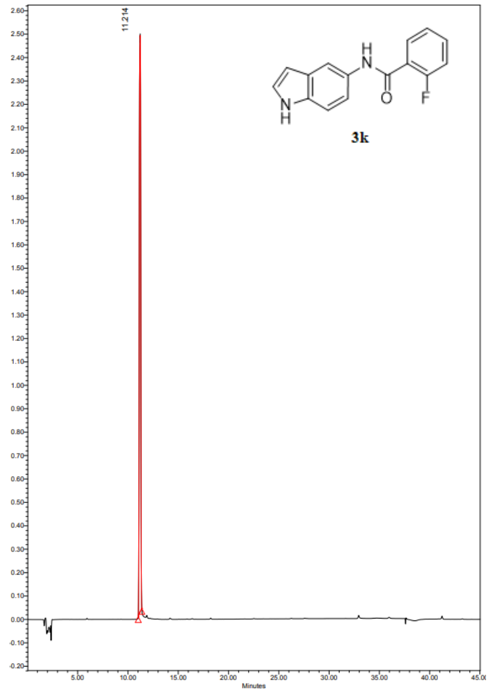

	Name	Retention Time (min)	Purity1 Angle	Purity1 Threshold	PDA Match1 Spect. Name	PDA Match1 Angle	PDA Match1 Threshold	PDA Match1 Lib. Name	Area (μV*sec)	% Area	Height (μV)	Int Type	Amount
1		11.195							247702	1.09	48572	bb	
2		13.051							25392	0.11	5154	bb	
3		13.350							22510345	98.80	2477503	bb	

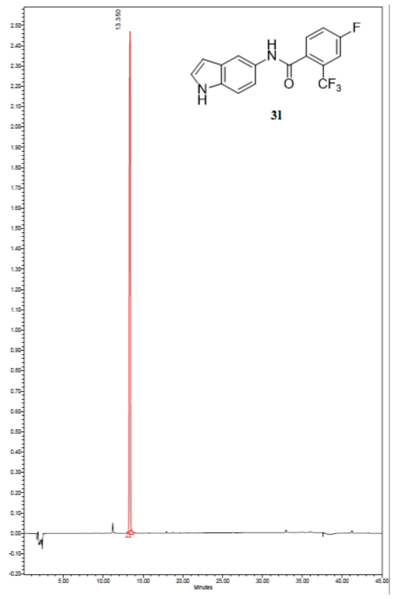

	Name	Retention Time (min)	Purity1 Angle	Purity1 Threshold	PDA Match1 Spect. Name	PDA Match1 Angle	PDA Match1 Threshold	PDA Match1 Lib. Name	Area (µV*sec)	% Area	Height (µV)	Int Type	Amount
1		5.348							341917	1.19	49608	bb	
2		7.726							120456	0.42	28160	bb	
3		10.964							27885896	97.06	2589526	bb	
4		12.796							302127	1.05	19118	bb	
5		15.733							81303	0.28	16951	bb	

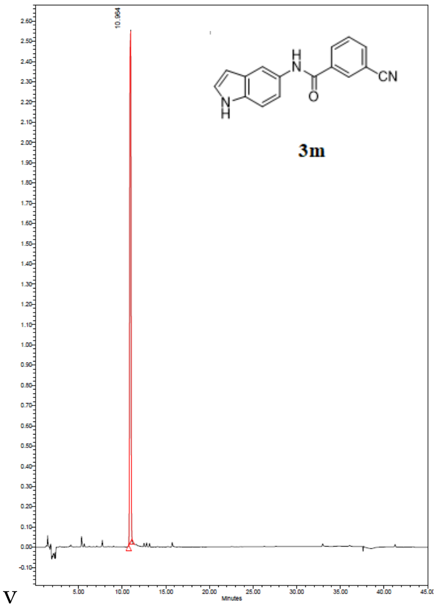

V

	Name	Retention Time (min)	Purity1 Angle	Purity1 Threshold	PDA Match1 Spect. Name	PDA Match1 Angle	PDA Match1 Threshold	PDA Match1 Lib. Name	Area (µV*sec)	% Area	Height (µV)	Int Type	Amount
1		11.230							64483	0.23	15474	bb	
2		13.164							67342	0.24	9602	bb	
3		13.875							27736031	98.79	2555833	bb	
4		17.374							207675	0.74	19992	bb	

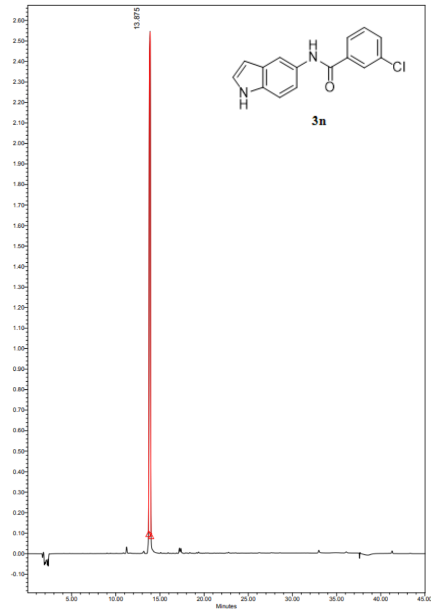

	Name	Retention Time (min)	Purity1 Angle	Purity1 Threshold	PDA Match1 Spect. Name	PDA Match1 Angle	PDA Match1 Threshold	PDA Match1 Lib. Name	Area (µV*sec)	% Area	Height (µV)	Int Type	Amount
1		7.374							131151	0.33	25900	bb	
2		11.355							38566049	96.81	2617879	bb	
3		14.045							1138043	2.86	86959	bb	

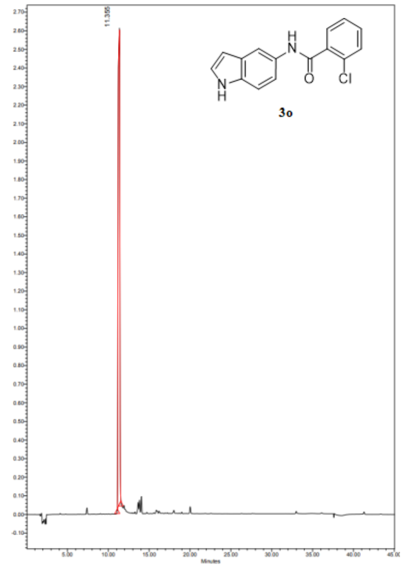

	Name	Retention Time (min)	Purity1 Angle	Purity1 Threshold	PDA Match1 Spect. Name	PDA Match1 Angle	PDA Match1 Threshold	PDA Match1 Lib. Name	Area (µV*sec)	% Area	Height (µV)	Int Type	Amount
1		9.718							135831	0.44	26761	bb	
2		13.361							30485	0.10	5746	bb	
3		13.748							30382869	97.74	2605835	bb	
4		14.257							55625	0.18	11829	bb	
5		15.267							93708	0.30	15387	bb	
6		16.472							43692	0.14	10354	bb	
7		17.614							343782	1.11	30899	bb	

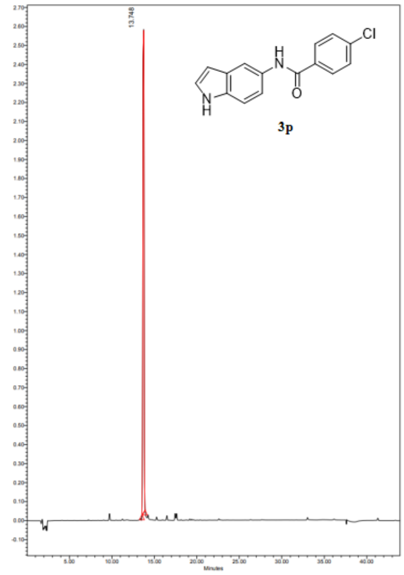

	Name	Retention Time (min)	Purity1 Angle	Purity1 Threshold	PDA Match1 Spect. Name	PDA Match1 Angle	PDA Match1 Threshold	PDA Match1 Lib. Name	Area (µV*sec)	% Area	Height (µV)	Int Type	Amount
1		17.291							23093841	100.00	2401255	bb	

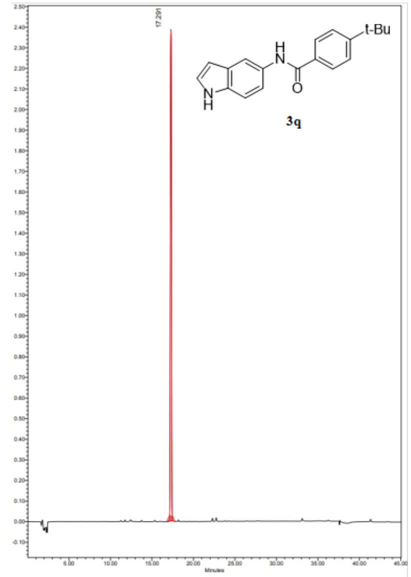

	Name	Retention Time (min)	Purity1 Angle	Purity1 Threshold	PDA Match1 Spect. Name	PDA Match1 Angle	PDA Match1 Threshold	PDA Match1 Lib. Name	Area (µV*sec)	% Area	Height (µV)	Int Type	Amount
1		14.501							26882669	99.80	2440766	bb	
2		20.866							53116	0.20	11742	bb	

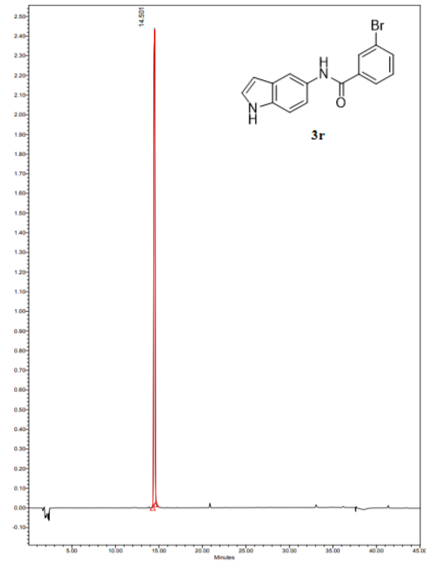

	Name	Retention Time (min)	Purity1 Angle	Purity1 Threshold	PDA Match1 Spect. Name	PDA Match1 Angle	PDA Match1 Threshold	PDA Match1 Lib. Name	Area (µV*sec)	% Area	Height (µV)	Int Type	Amount
1		15.821							7902091	100.00	1219456	bb	

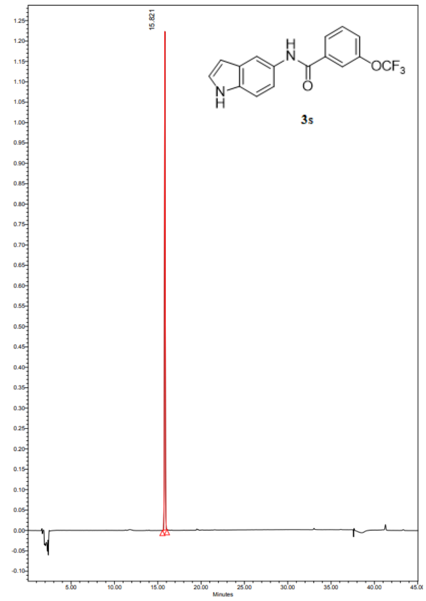

	Name	Retention Time (min)	Purity1 Angle	Purity1 Threshold	PDA Match1 Spect. Name	PDA Match1 Angle	PDA Match1 Threshold	PDA Match1 Lib. Name	Area (µV*sec)	% Area	Height (µV)	Int Type	Amount
1		13.518							30816	0.14	7913	bb	
2		15.710							21523563	99.65	2411299	bb	
3		19.739							44224	0.20	5786	bb	

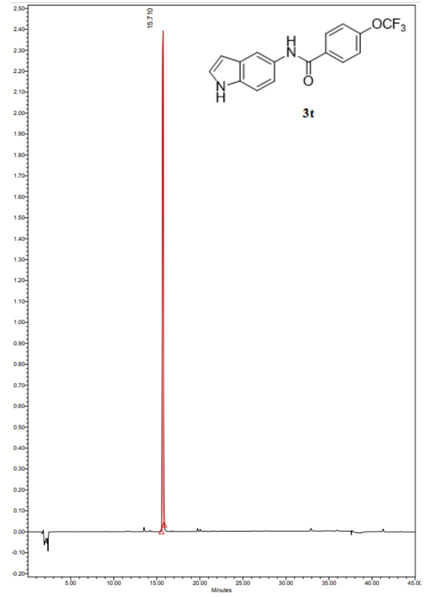

	Name	Retention Time (min)	Purity1 Angle	Purity1 Threshold	PDA Match1 Spect. Name	PDA Match1 Angle	PDA Match1 Threshold	PDA Match1 Lib. Name	Area (µV*sec)	% Area	Height (µV)	Int Type	Amount
1		15.174							13095325	99.74	1931903	bb	
2		17.541							33569	0.26	7442	bb	

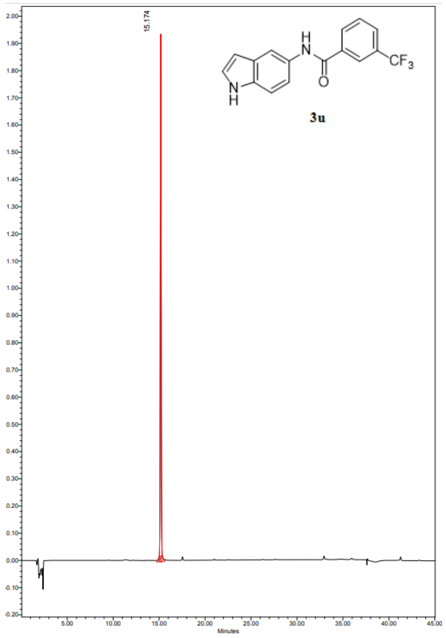

	Name	Retention Time (min)	Purity1 Angle	Purity1 Threshold	PDA Match1 Spect. Name	PDA Match1 Angle	PDA Match1 Threshold	PDA Match1 Lib. Name	Area (µV*sec)	% Area	Height (µV)	Int Type	Amount	Units
1		15.191							5061562	99.98	775599	bb		
2		20.883							832	0.02	-271	bb		

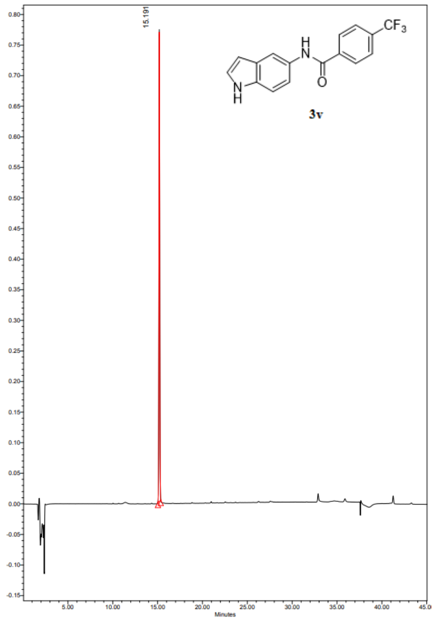

	Name	Retention Time (min)	Purity1 Angle	Purity1 Threshold	PDA Match1 Spect. Name	PDA Match1 Angle	PDA Match1 Threshold	PDA Match1 Lib. Name	Area (µV*sec)	% Area	Height (µV)	Int Type	Amount
1		14.209							4338	0.04	352	bb	
2		17.439							9987329	99.96	1442664	bb	

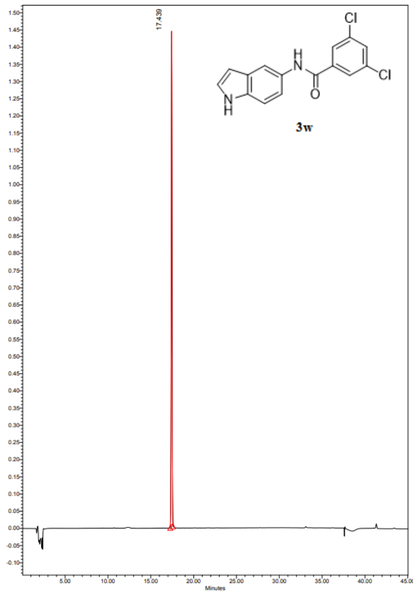

	Name	Retention Time (min)	Purity1 Angle	Purity1 Threshold	PDA Match1 Spect. Name	PDA Match1 Angle	PDA Match1 Threshold	PDA Match1 Lib. Name	Area (µV*sec)	% Area	Height (µV)	Int Type	Amount
1		12.478							25571048	100.00	1767966	bb	

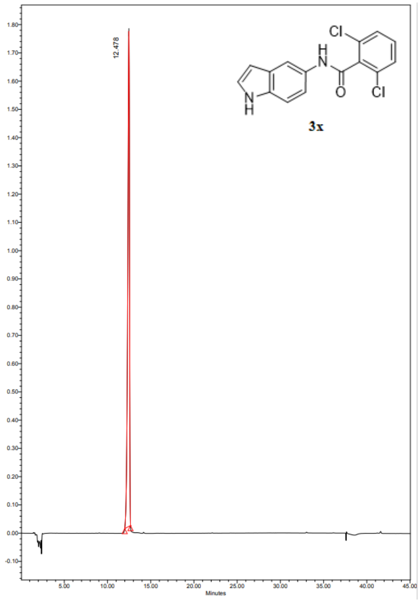

4. HRMS charts

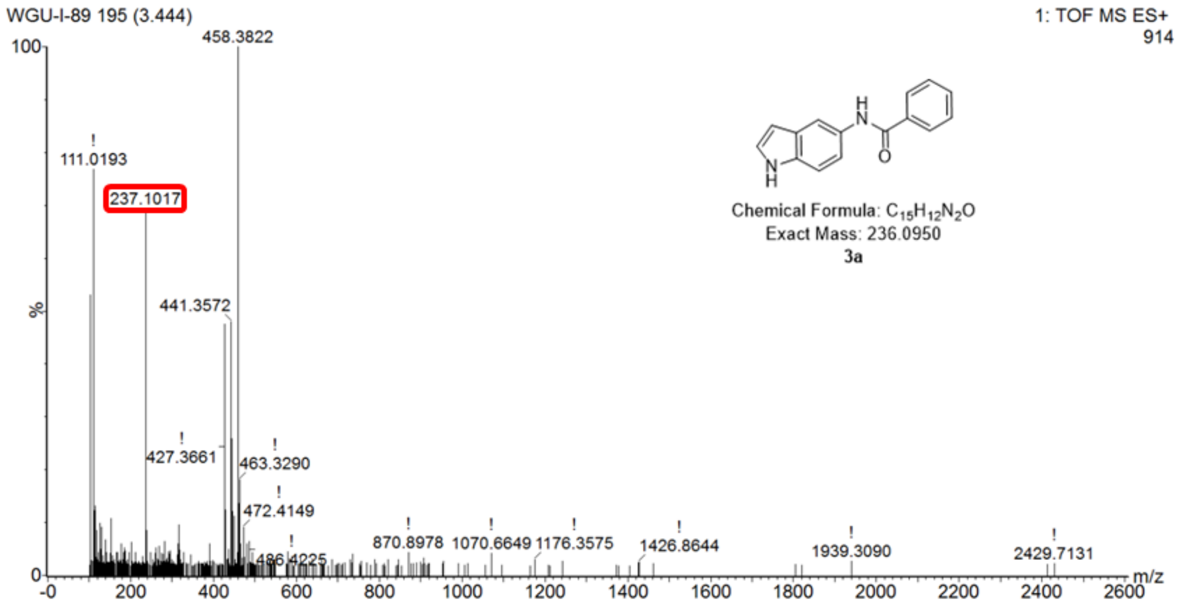

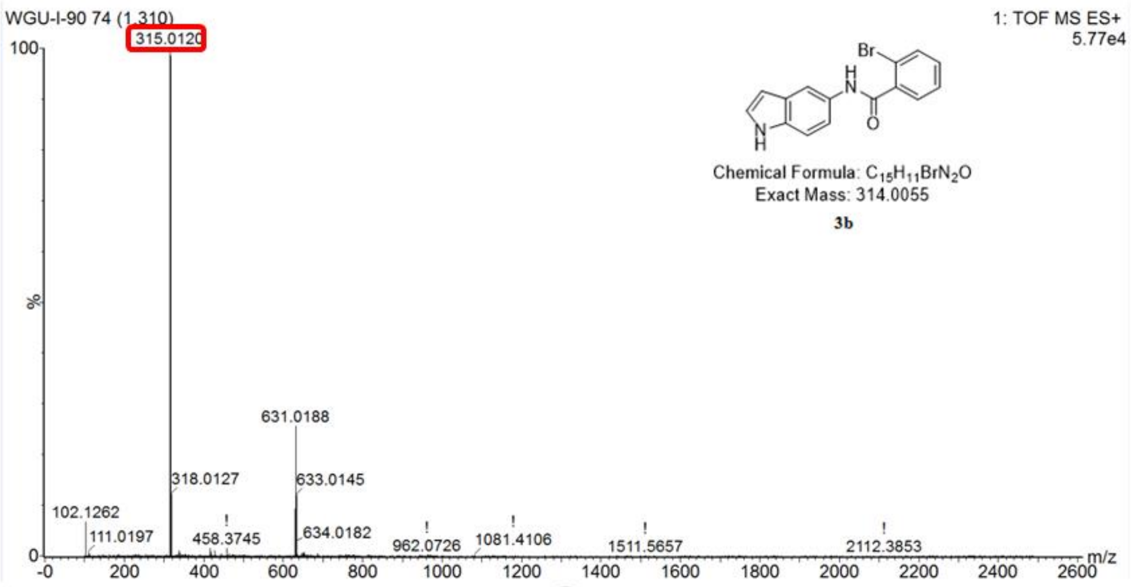

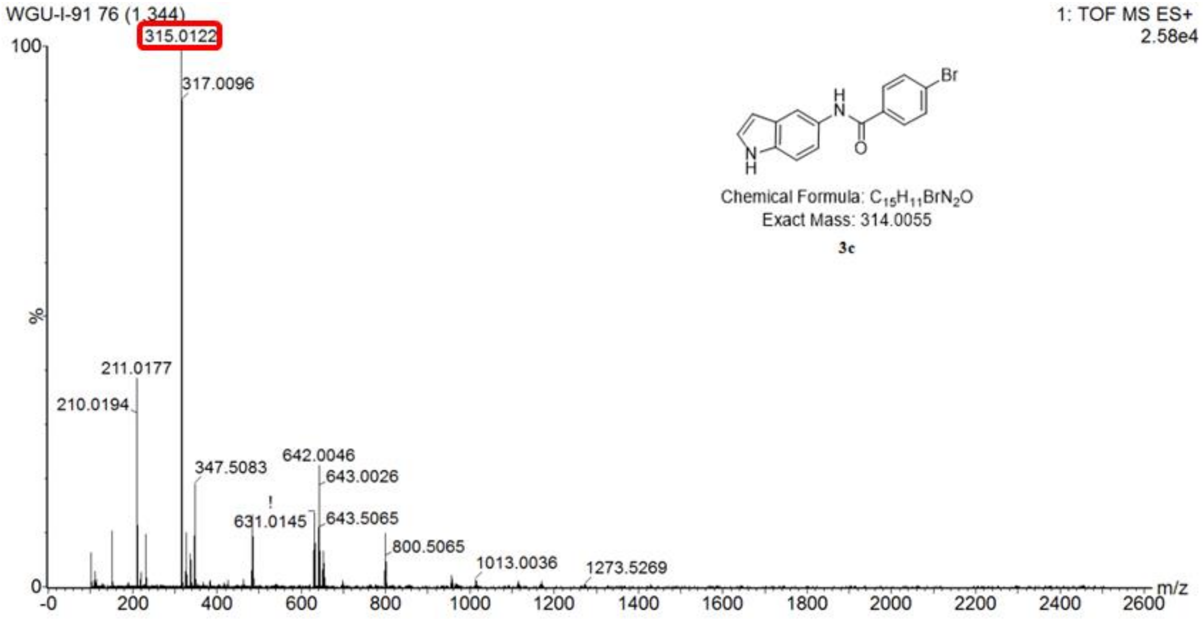
WGU-I-91 76 (1.344)
1: TOF MS ES+
2.58e4
315.0122
317.0096
211.0177
210.0194
347.5083
642.0046
643.0026
631.0145
643.5065
800.5065
1013.0036
1273.5269
Chemical Formula: $C_{15}H_{11}BrN_2O$
Exact Mass: 314.0055
3c
m/z
%

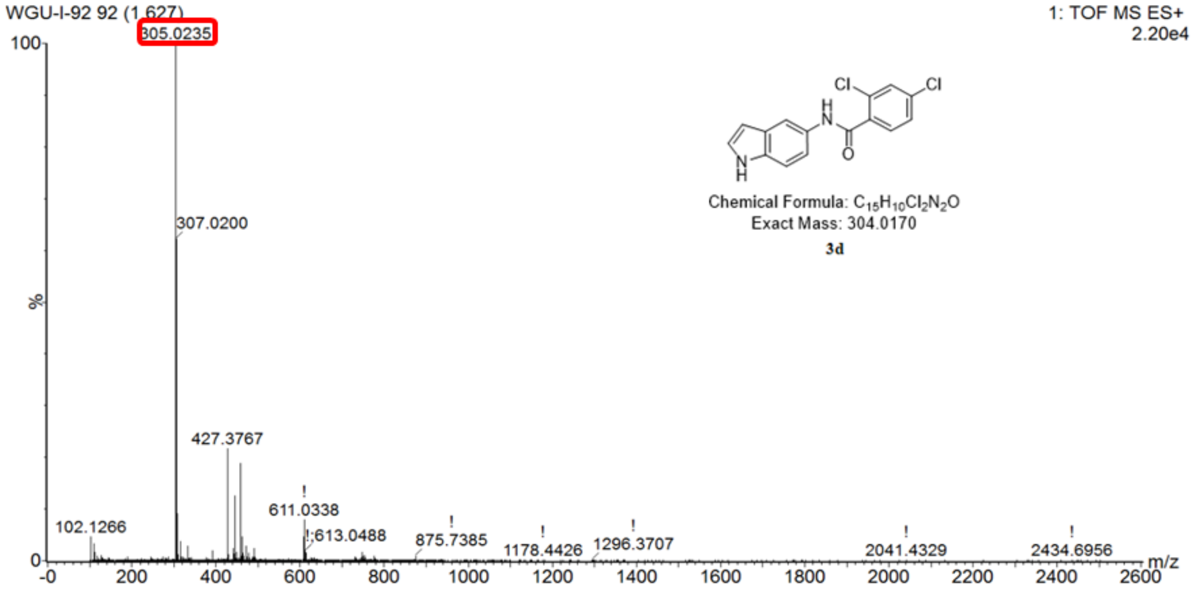
WGU-I-92 92 (1.627)
1: TOF MS ES+
2.20e4
305.0235
307.0200
427.3767
611.0338
613.0488
102.1266
875.7385
1178.4426
1296.3707
2041.4329
2434.6956
Chemical Formula: $C_{15}H_{10}Cl_2N_2O$
Exact Mass: 304.0170
3d
m/z
%

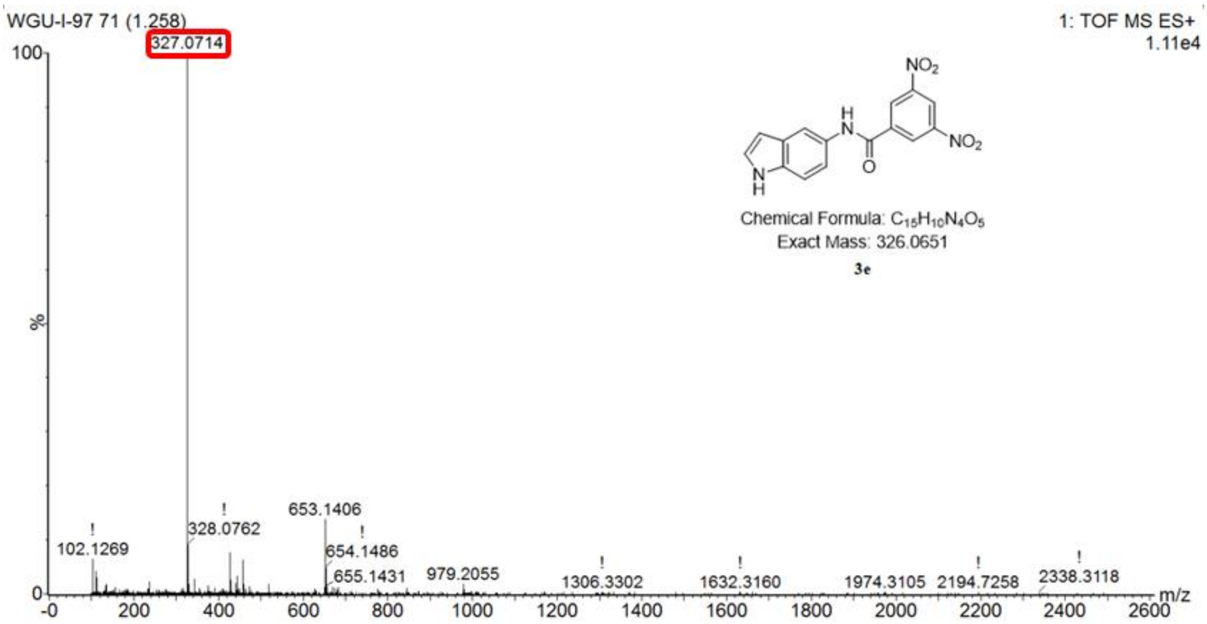

WGU-I-97 71 (1.258)
1: TOF MS ES+
1.11e4
327.0714
328.0762
102.1269
653.1406
654.1486
655.1431
979.2055
1306.3302
1632.3160
1974.3105
2194.7258
2338.3118
%
m/z
NO2
NO2
Chemical Formula: C15H10N4O5
Exact Mass: 326.0651
3e

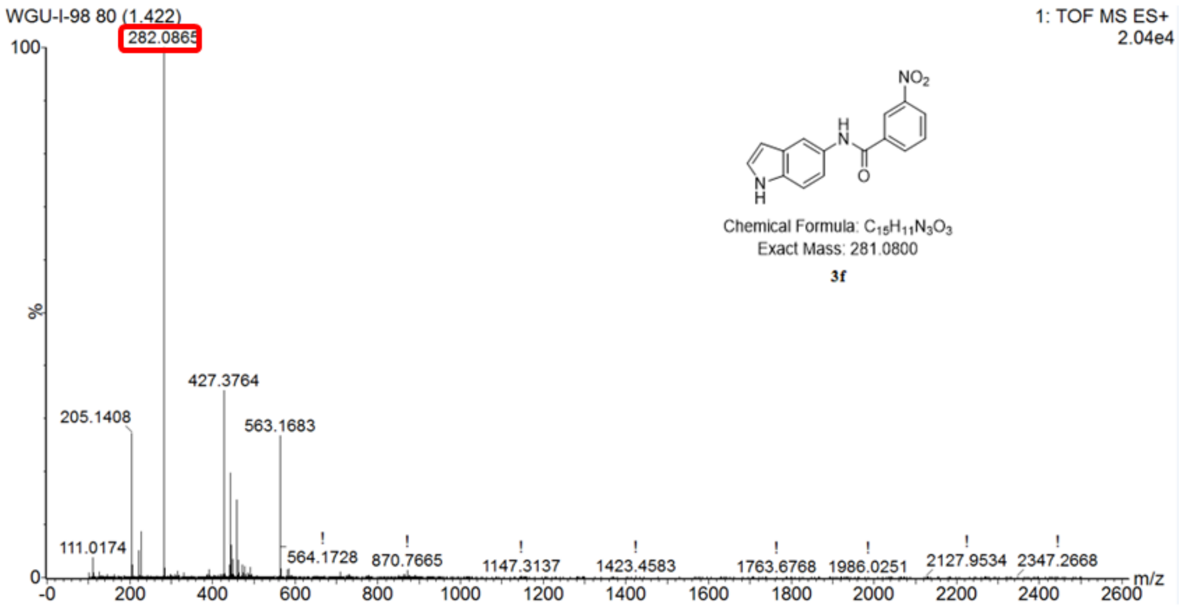

WGU-I-98 80 (1.422)
1: TOF MS ES+
2.04e4
282.0865
205.1408
427.3764
563.1683
111.0174
564.1728
870.7665
1147.3137
1423.4583
1763.6768
1986.0251
2127.9534
2347.2668
%
m/z
NO2
Chemical Formula: C15H11N3O3
Exact Mass: 281.0800
3f

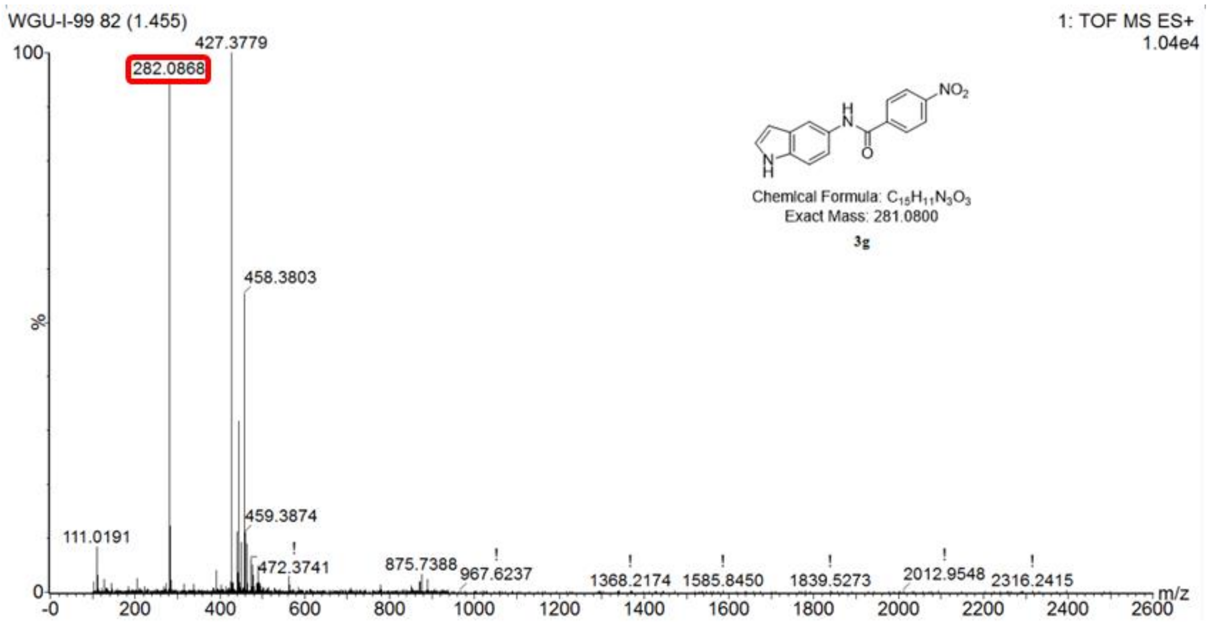
WGU-I-99 82 (1.455)
1: TOF MS ES+
1.04e4
100
%
0
427.3779
282.0868
458.3803
459.3874
111.0191
472.3741
875.7388
967.6237
1368.2174
1585.8450
1839.5273
2012.9548
2316.2415
-0
200
400
600
800
1000
1200
1400
1600
1800
2000
2200
2400
2600
m/z
NO2
H
N
O
N
H
Chemical Formula: $C_{15}H_{11}N_3O_3$
Exact Mass: 281.0800
3g

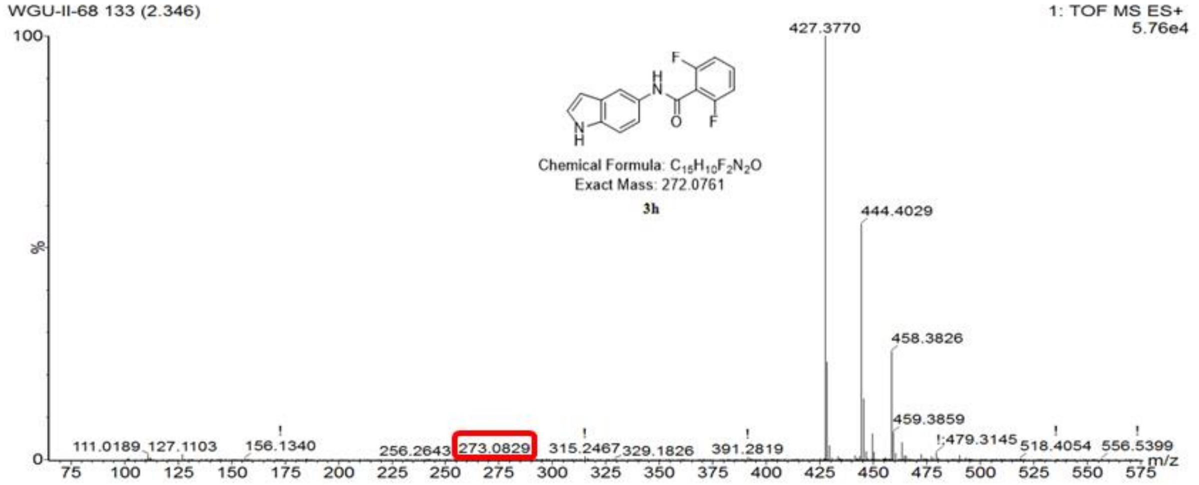
WGU-II-68 133 (2.346)
1: TOF MS ES+
5.76e4
100
%
0
427.3770
444.4029
458.3826
459.3859
111.0189
127.1103
156.1340
256.2643
273.0829
315.2467
329.1826
391.2819
479.3145
518.4054
556.5399
75
100
125
150
175
200
225
250
275
300
325
350
375
400
425
450
475
500
525
550
575
m/z
F
H
N
O
F
N
H
Chemical Formula: $C_{15}H_{10}F_2N_2O$
Exact Mass: 272.0761
3h

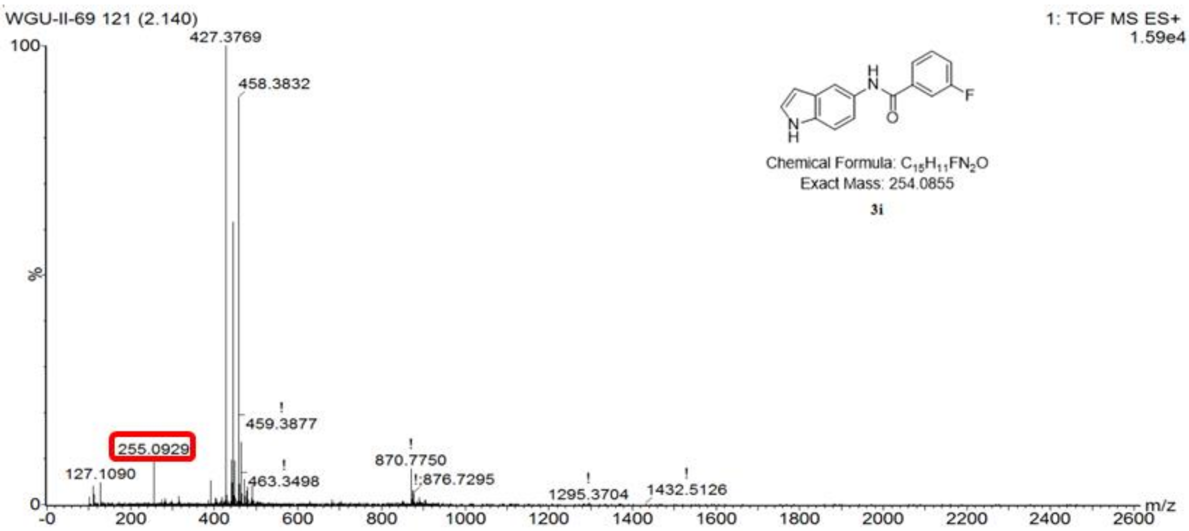
WGU-II-69 121 (2.140)
1: TOF MS ES+
1.59e4
427.3769
458.3832
459.3877
255.0929
127.1090
463.3498
870.7750
876.7295
1295.3704
1432.5126
Chemical Formula: C15H11FN2O
Exact Mass: 254.0855
3i
%
m/z

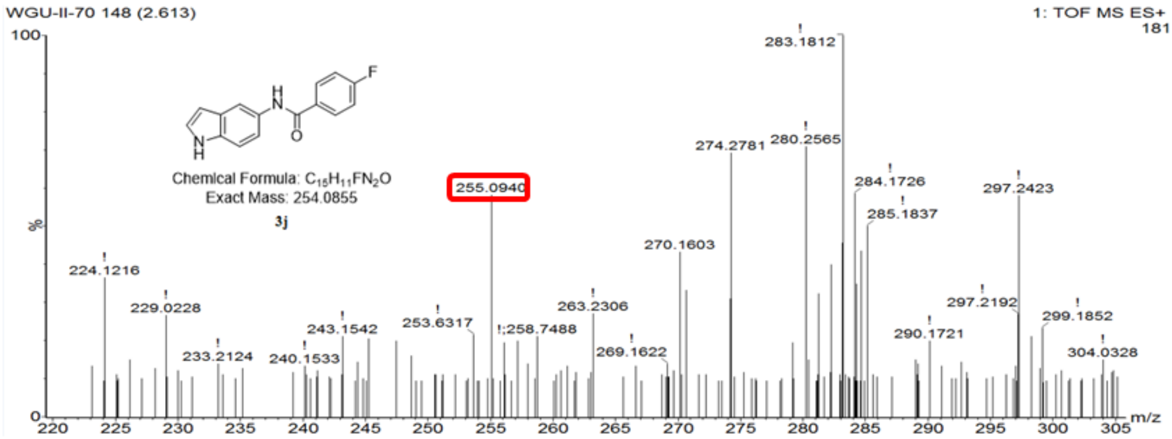
WGU-II-70 148 (2.613)
1: TOF MS ES+
181
283.1812
274.2781
280.2565
284.1726
285.1837
297.2423
255.0940
270.1603
224.1216
229.0228
233.2124
240.1533
243.1542
253.6317
258.7488
263.2306
269.1622
290.1721
297.2192
299.1852
304.0328
Chemical Formula: C15H11FN2O
Exact Mass: 254.0855
3j
%
m/z

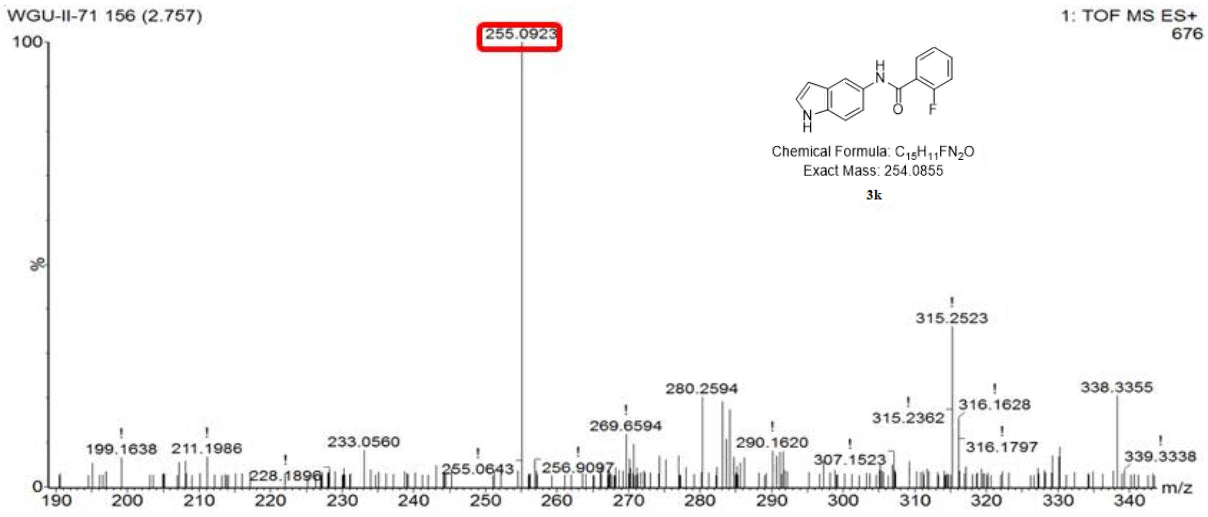

WGU-II-71 156 (2.757)
1: TOF MS ES+
676
100
%
0
255.0923
315.2523
280.2594
338.3355
316.1628
315.2362
269.6594
290.1620
316.1797
339.3338
307.1523
199.1638
211.1986
233.0560
228.1896
255.0643
256.9097
190
200
210
220
230
240
250
260
270
280
290
300
310
320
330
340
m/z
H
N
O
F
Chemical Formula: $C_{15}H_{11}FN_2O$
Exact Mass: 254.0855
3k

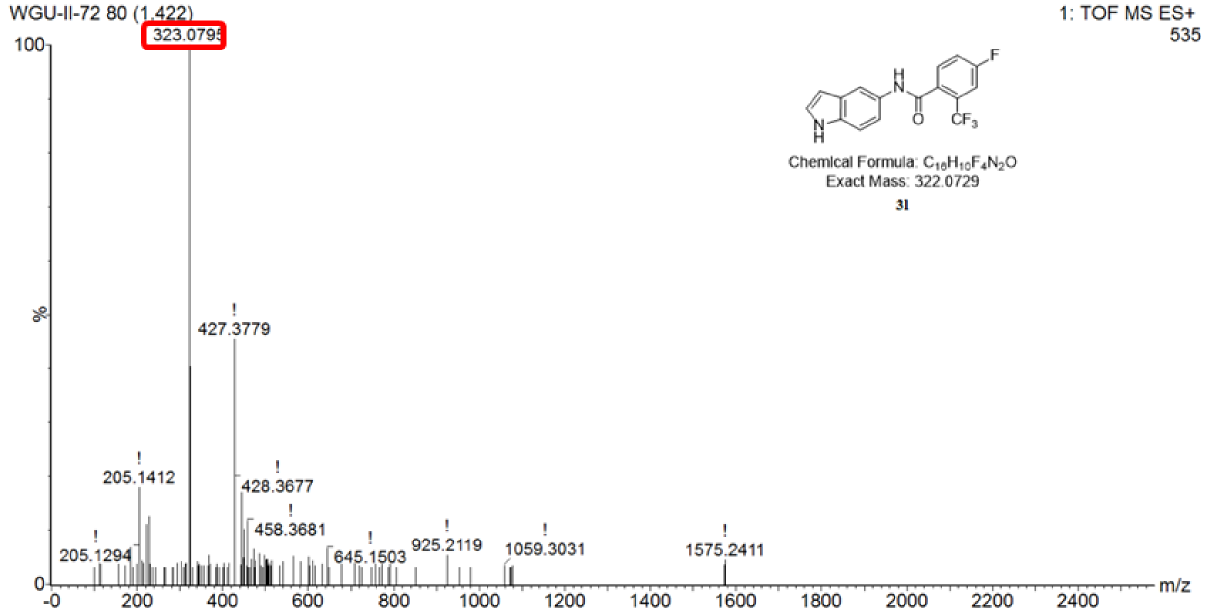

WGU-II-72 80 (1.422)
1: TOF MS ES+
535
100
%
0
323.0795
427.3779
205.1412
428.3677
458.3681
205.1294
645.1503
925.2119
1059.3031
1575.2411
-0
200
400
600
800
1000
1200
1400
1600
1800
2000
2200
2400
m/z
H
N
O
F
CF_3
Chemical Formula: $C_{16}H_{10}F_4N_2O$
Exact Mass: 322.0729
3l

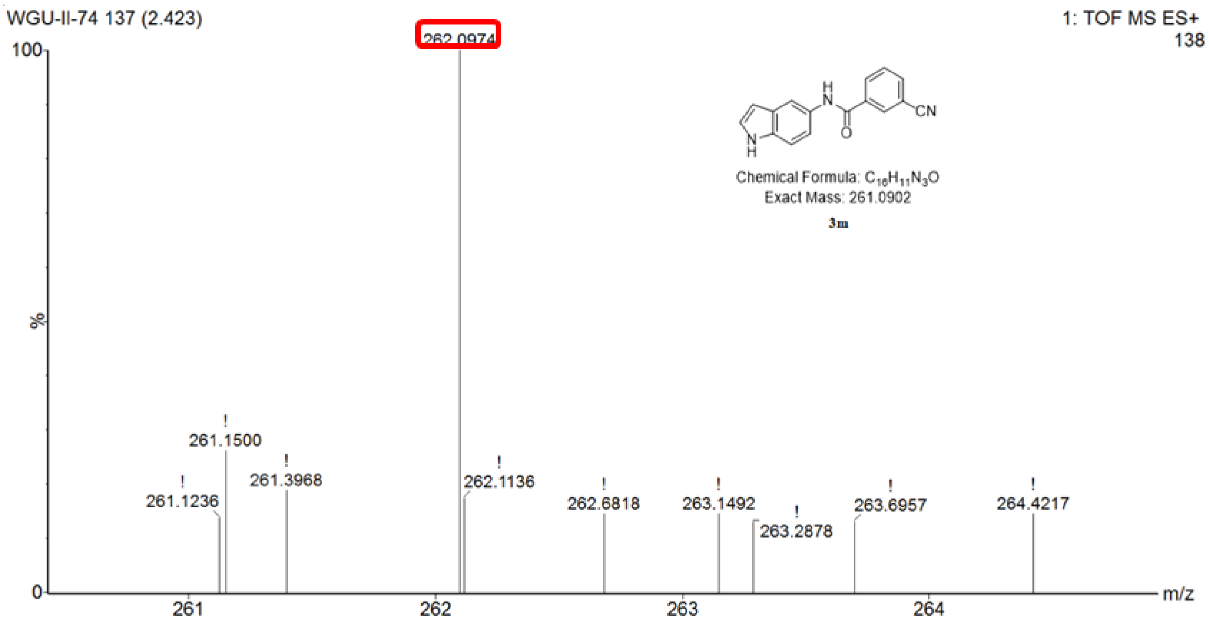
WGU-II-74 137 (2.423)
1: TOF MS ES+
138
262.0974
Chemical Formula: $C_{16}H_{11}N_3O$
Exact Mass: 261.0902
3m
261.1236
261.1500
261.3968
262.1136
262.6818
263.1492
263.2878
263.6957
264.4217
m/z
%

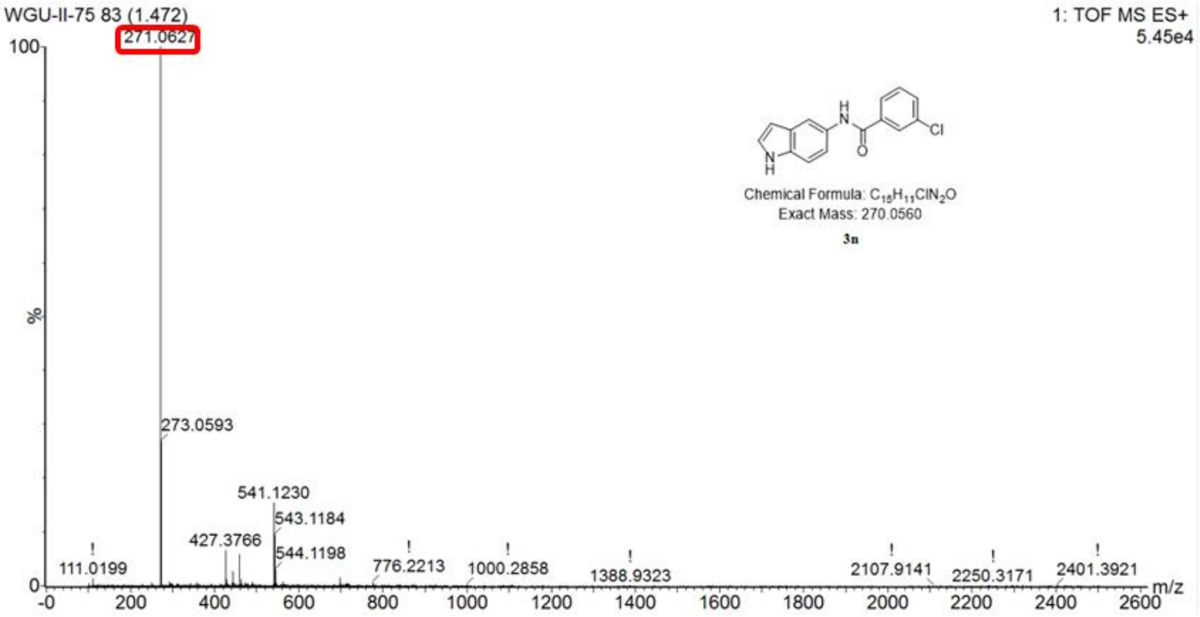
WGU-II-75 83 (1.472)
1: TOF MS ES+
5.45e4
271.0627
Chemical Formula: $C_{15}H_{11}ClN_2O$
Exact Mass: 270.0560
3n
273.0593
541.1230
543.1184
427.3766
544.1198
111.0199
776.2213
1000.2858
1388.9323
2107.9141
2250.3171
2401.3921
m/z
%

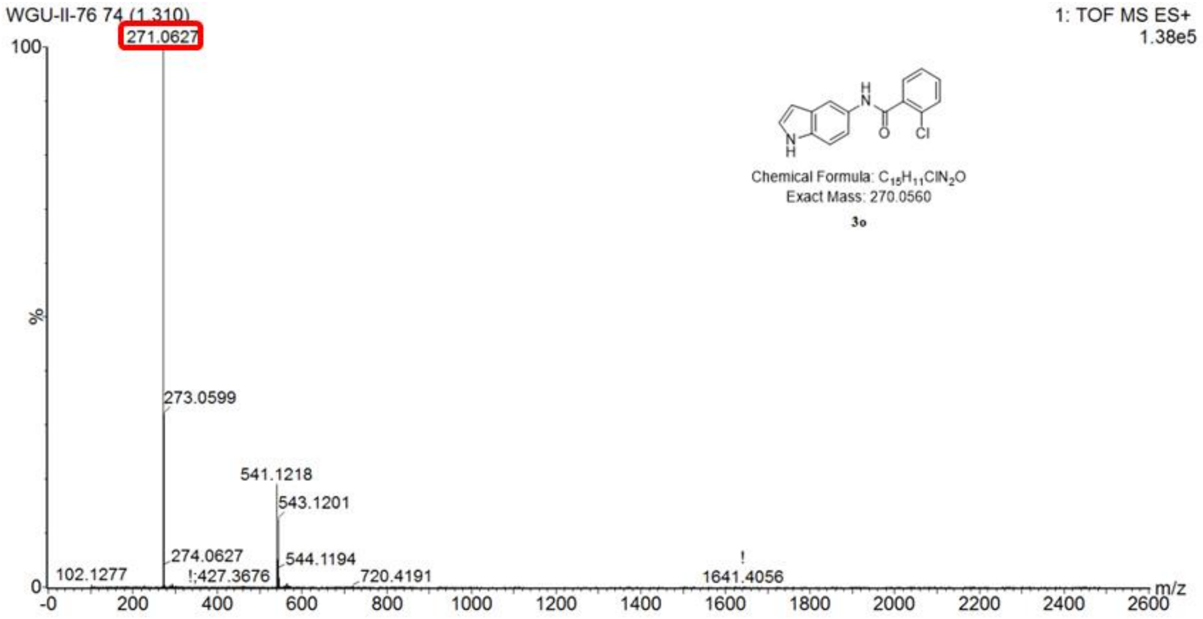
WGU-II-76 74 (1.310)
1: TOF MS ES+
1.38e5
271.0627
273.0599
541.1218
543.1201
274.0627
544.1194
102.1277
427.3676
720.4191
1641.4056
Chemical Formula: $C_{15}H_{11}ClN_2O$
Exact Mass: 270.0560
3o
m/z
%

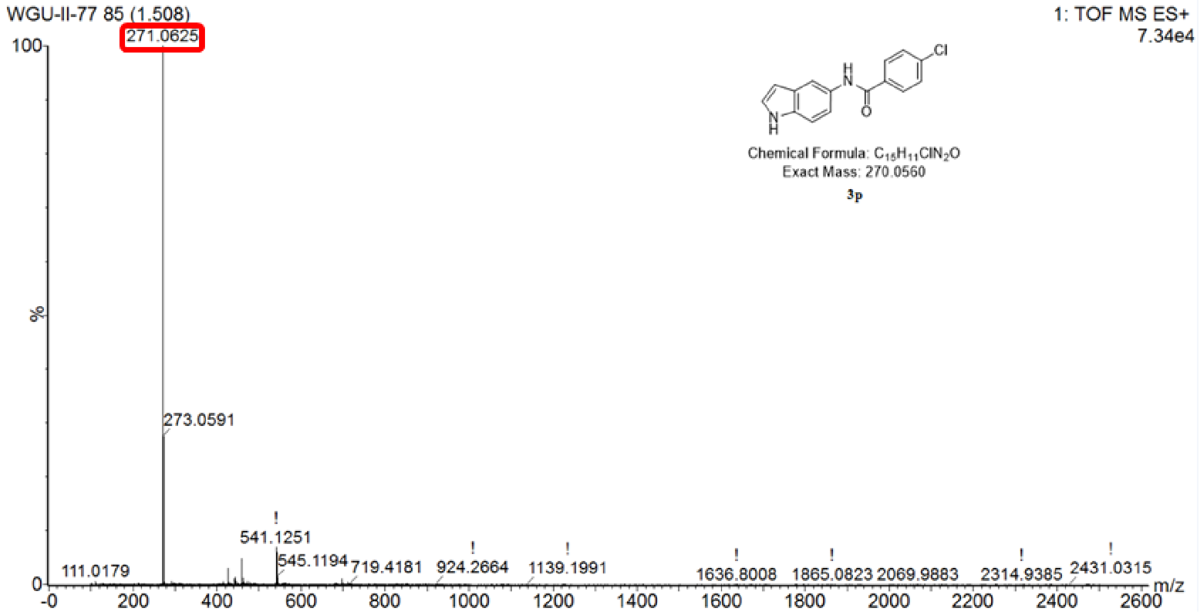
WGU-II-77 85 (1.508)
1: TOF MS ES+
7.34e4
271.0625
273.0591
541.1251
545.1194
111.0179
719.4181
924.2664
1139.1991
1636.8008
1865.0823
2069.9883
2314.9385
2431.0315
Chemical Formula: $C_{15}H_{11}ClN_2O$
Exact Mass: 270.0560
3p
m/z
%

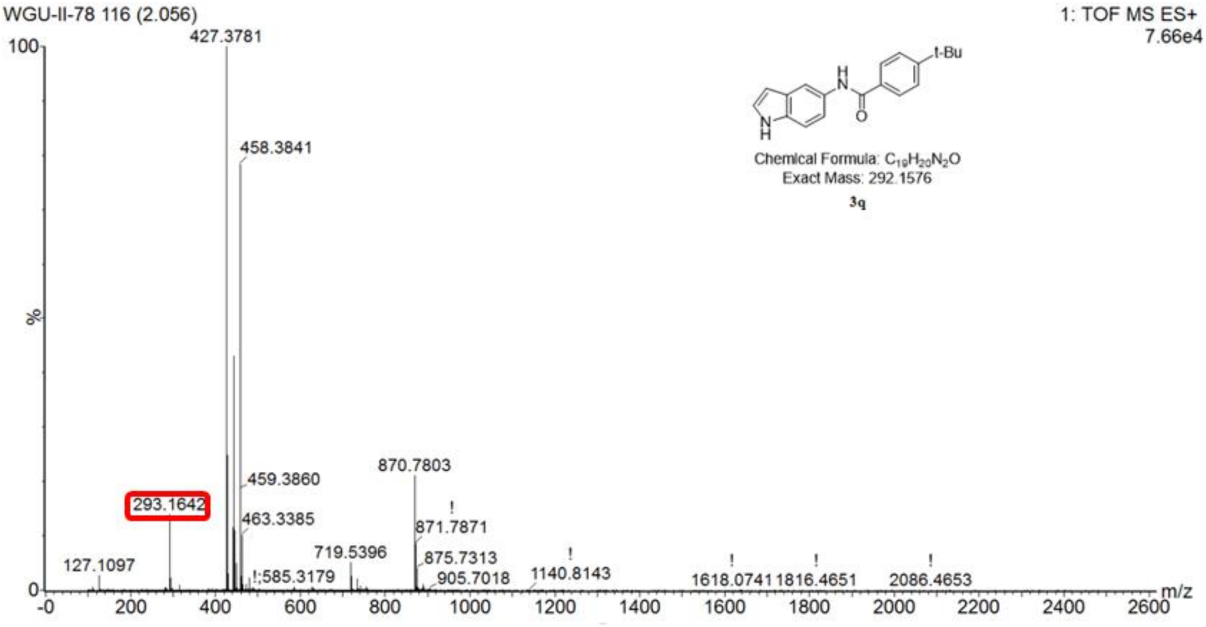
WGU-II-78 116 (2.056)
1: TOF MS ES+
7.66e4
t-Bu
Chemical Formula: $C_{19}H_{20}N_2O$
Exact Mass: 292.1576
3q
427.3781
458.3841
459.3860
293.1642
463.3385
870.7803
871.7871
127.1097
719.5396
875.7313
585.3179
905.7018
1140.8143
1618.0741
1816.4651
2086.4653
%
m/z

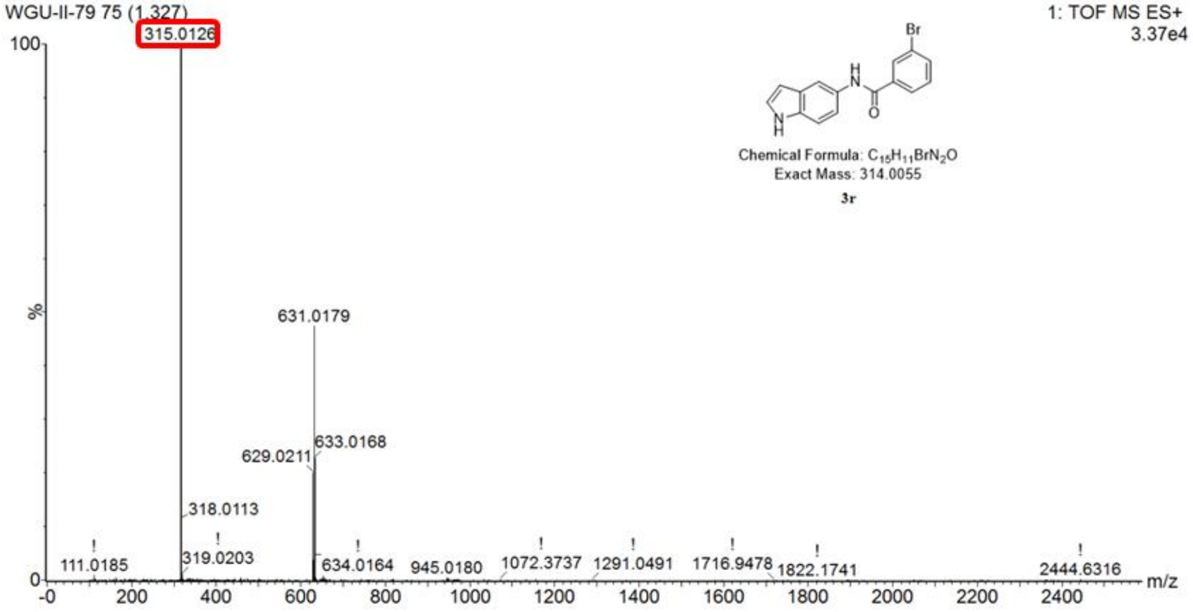
WGU-II-79 75 (1.327)
1: TOF MS ES+
3.37e4
Br
Chemical Formula: $C_{15}H_{11}BrN_2O$
Exact Mass: 314.0055
3r
315.0126
631.0179
633.0168
629.0211
318.0113
111.0185
319.0203
634.0164
945.0180
1072.3737
1291.0491
1716.9478
1822.1741
2444.6316
%
m/z

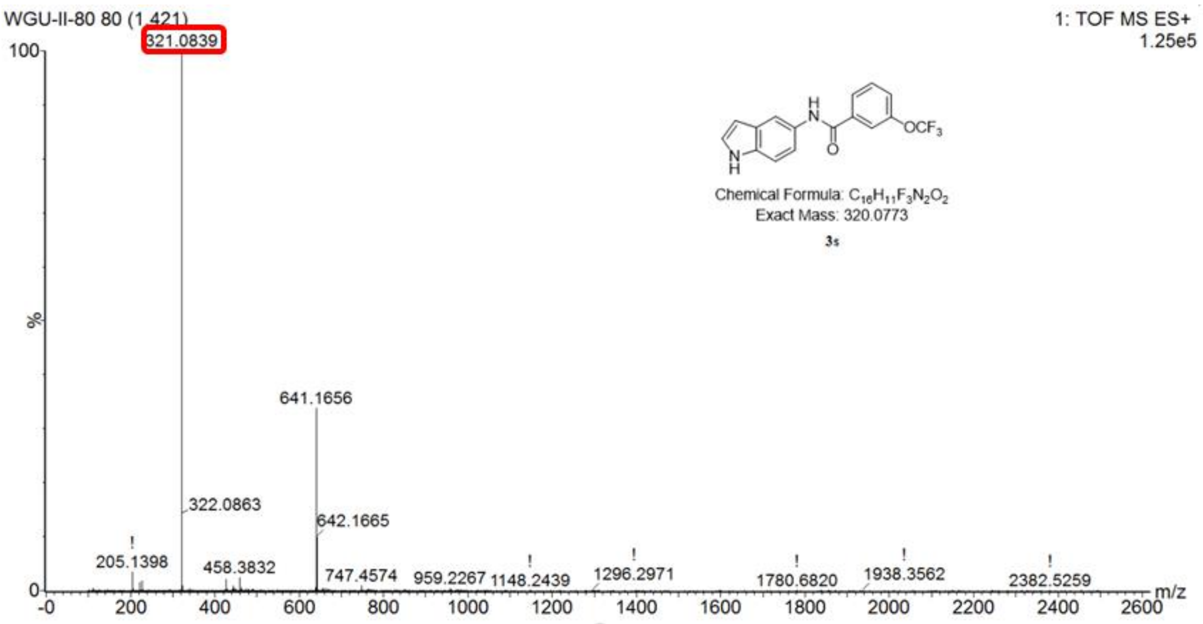
WGU-II-80 80 (1.421)
1: TOF MS ES+
1.25e5
321.0839
322.0863
641.1656
642.1665
205.1398
458.3832
747.4574
959.2267
1148.2439
1296.2971
1780.6820
1938.3562
2382.5259
100
%
0
m/z
OCF3
Chemical Formula: C16H11F3N2O2
Exact Mass: 320.0773
3s

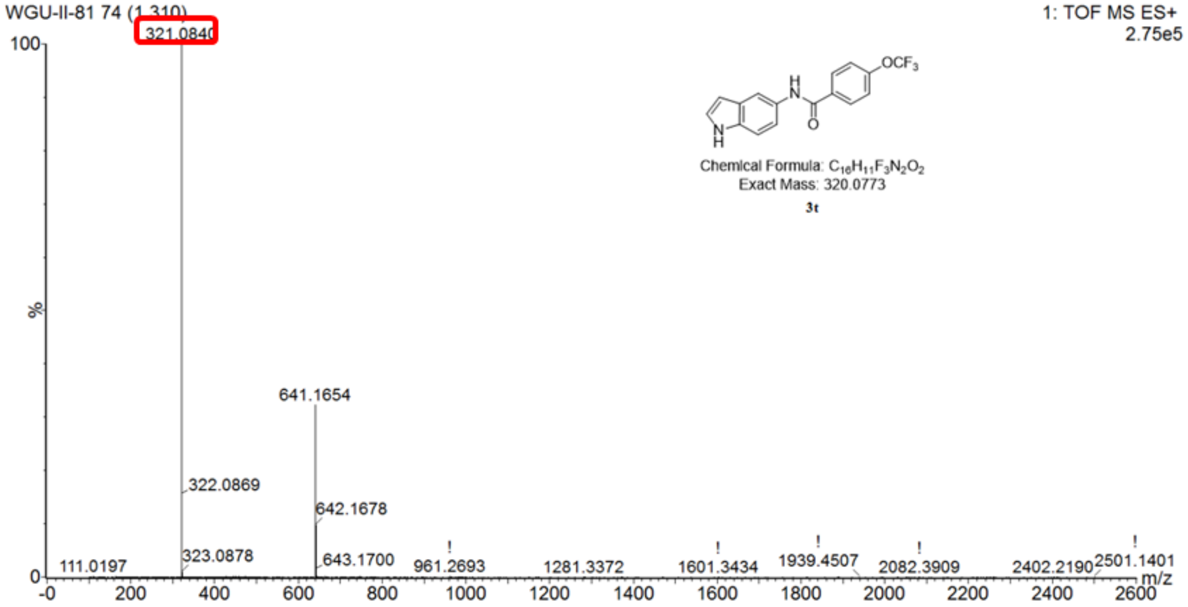
WGU-II-81 74 (1.310)
1: TOF MS ES+
2.75e5
321.0840
322.0869
323.0878
641.1654
642.1678
643.1700
111.0197
961.2693
1281.3372
1601.3434
1939.4507
2082.3909
2402.2190
2501.1401
100
%
0
m/z
OCF3
Chemical Formula: C16H11F3N2O2
Exact Mass: 320.0773
3t

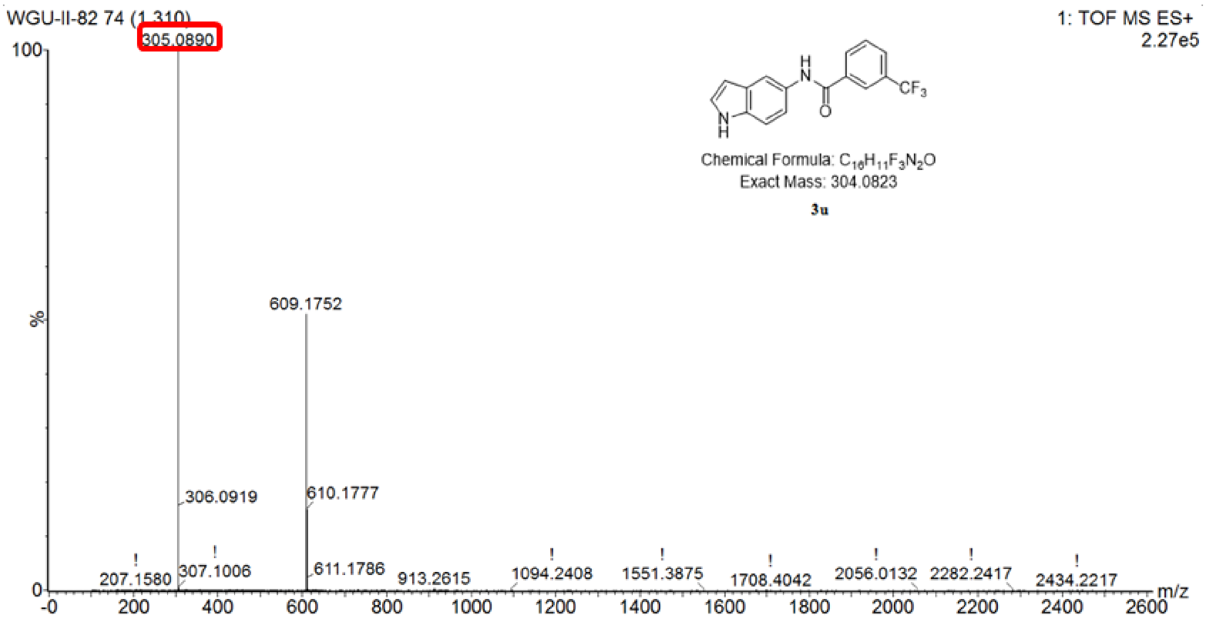

WGU-II-82 74 (1.310)
1: TOF MS ES+
2.27e5
305.0890
609.1752
306.0919
610.1777
207.1580
307.1006
611.1786
913.2615
1094.2408
1551.3875
1708.4042
2056.0132
2282.2417
2434.2217
Chemical Formula: C16H11F3N2O
Exact Mass: 304.0823
3u
%
m/z

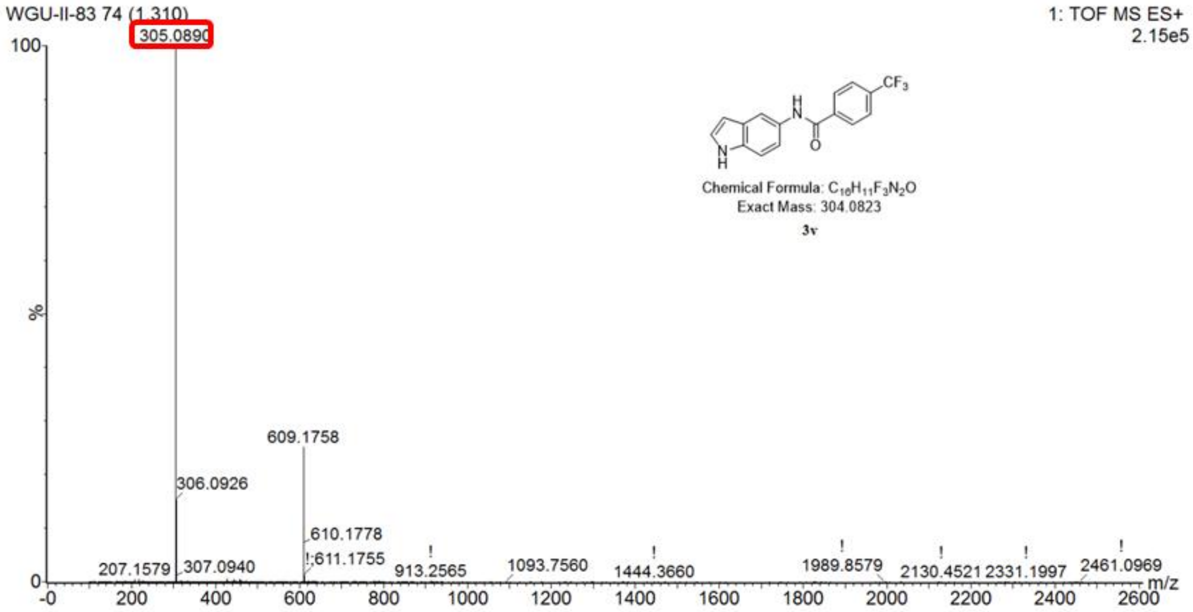

WGU-II-83 74 (1.310)
1: TOF MS ES+
2.15e5
305.0890
609.1758
306.0926
610.1778
207.1579
307.0940
611.1755
913.2565
1093.7560
1444.3660
1989.8579
2130.4521
2331.1997
2461.0969
Chemical Formula: C16H11F3N2O
Exact Mass: 304.0823
3v
%
m/z

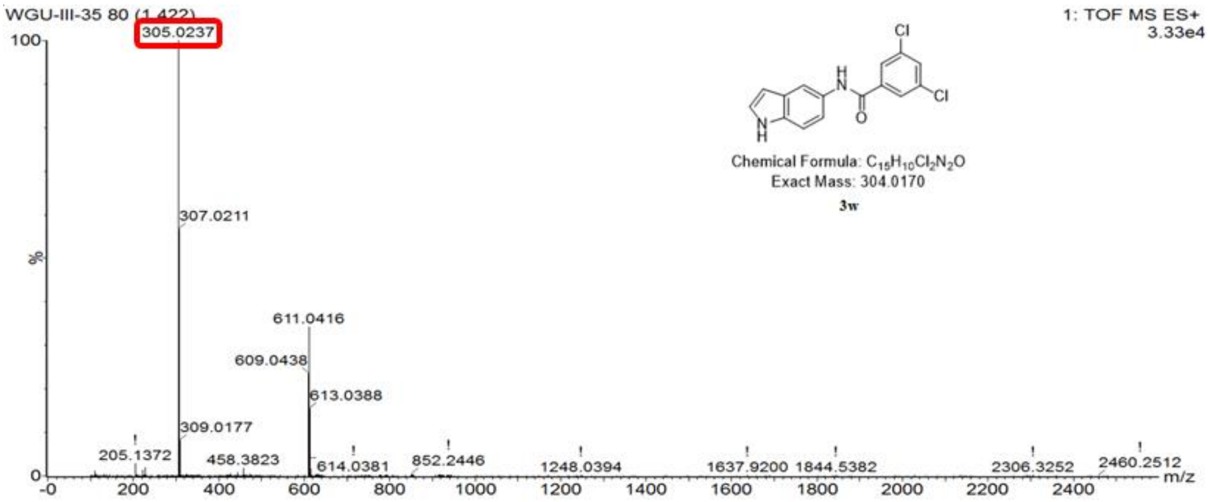
WGU-III-35 80 (1.422)
1: TOF MS ES+
3.33e4
305.0237
307.0211
611.0416
609.0438
613.0388
309.0177
205.1372
458.3823
614.0381
852.2446
1248.0394
1637.9200
1844.5382
2306.3252
2460.2512
m/z
%
Chemical Formula: $C_{15}H_{10}Cl_2N_2O$
Exact Mass: 304.0170
3w

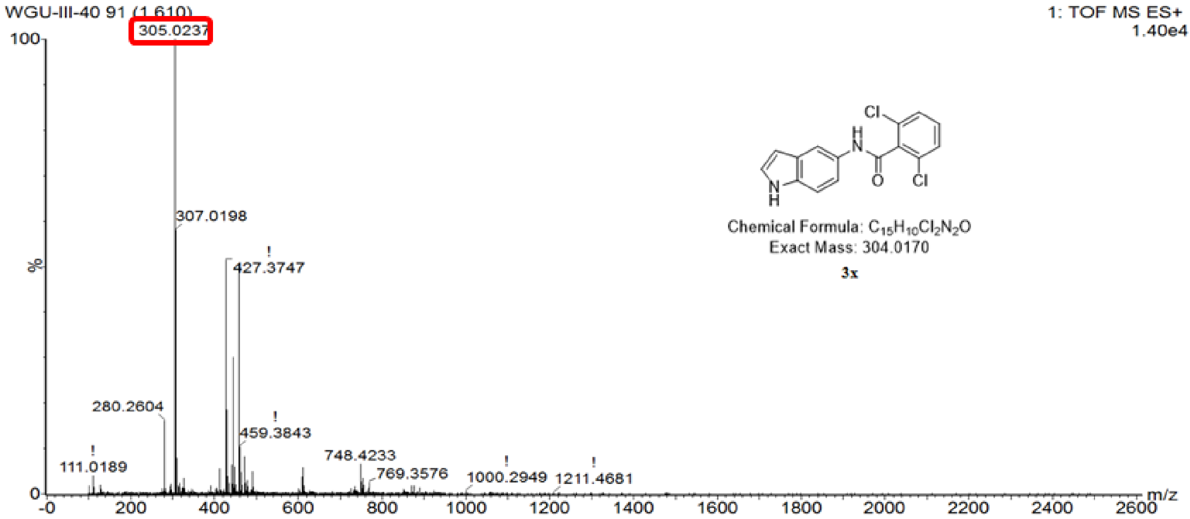
WGU-III-40 91 (1.610)
1: TOF MS ES+
1.40e4
305.0237
307.0198
427.3747
280.2604
459.3843
111.0189
748.4233
769.3576
1000.2949
1211.4681
m/z
%
Chemical Formula: $C_{15}H_{10}Cl_2N_2O$
Exact Mass: 304.0170
3x

5. IR charts

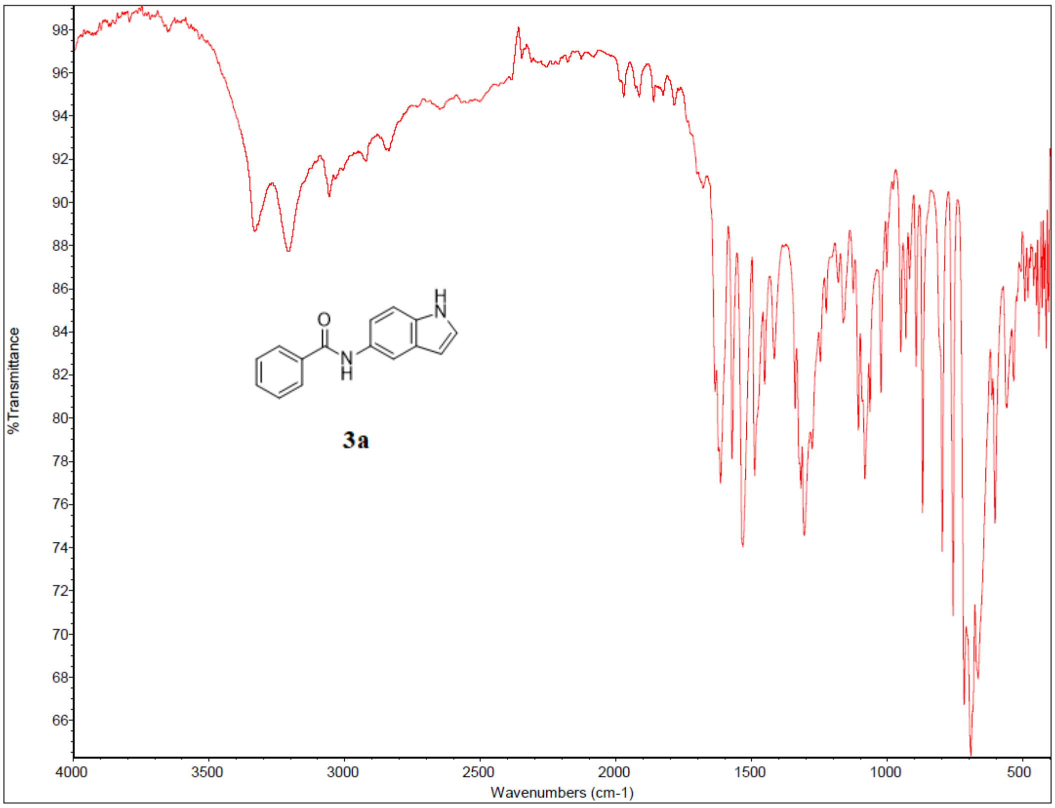

%Transmittance
Wavenumbers (cm-1)
3a

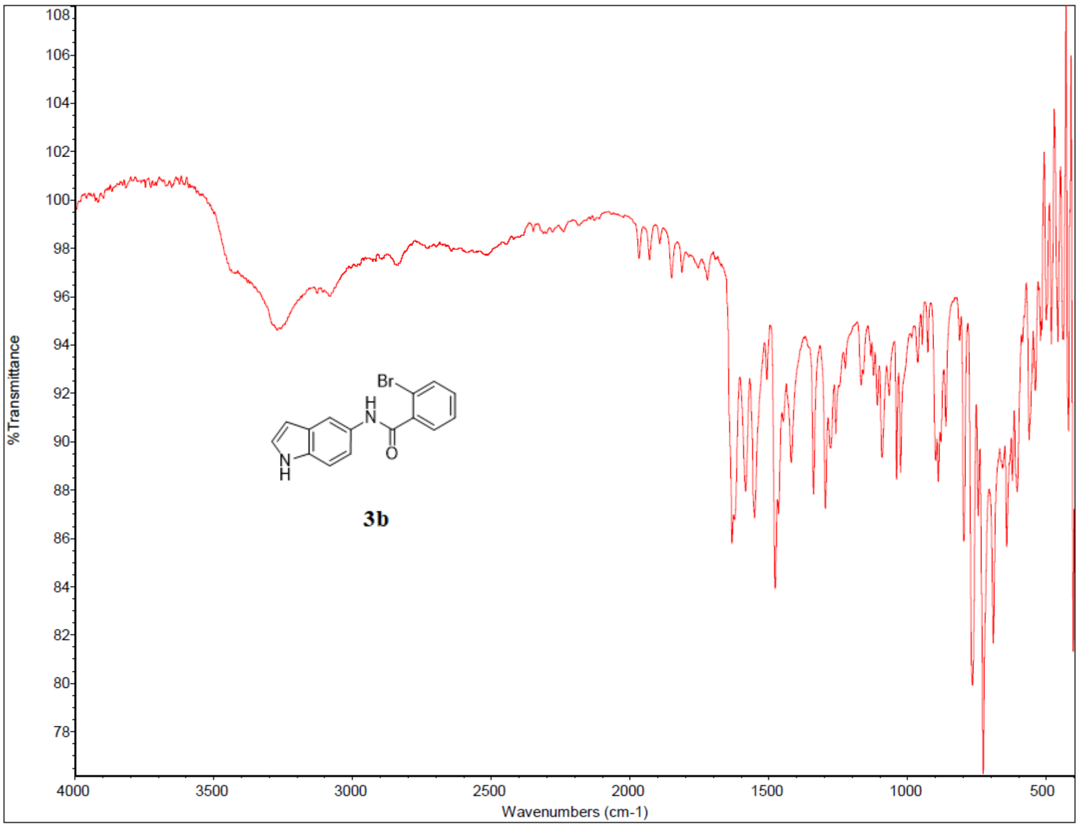

%Transmittance
Wavenumbers (cm-1)
3b

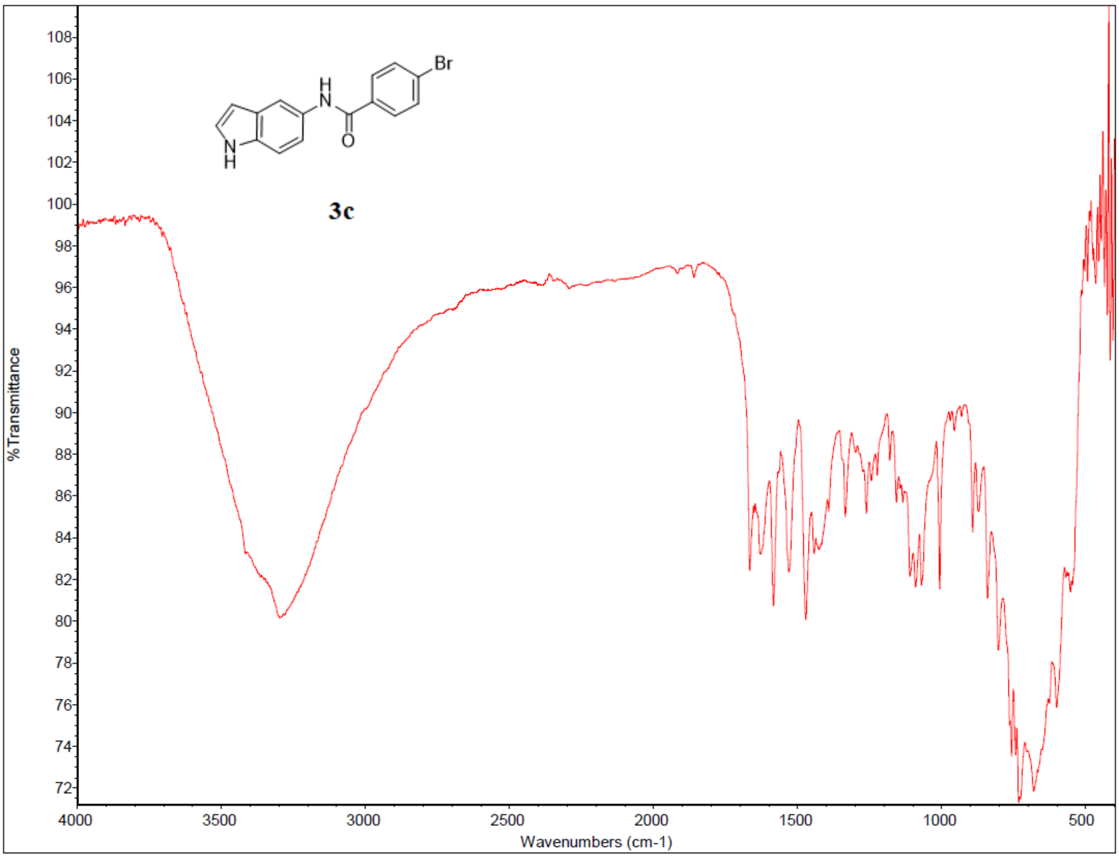

3c
%Transmittance
Wavenumbers (cm-1)

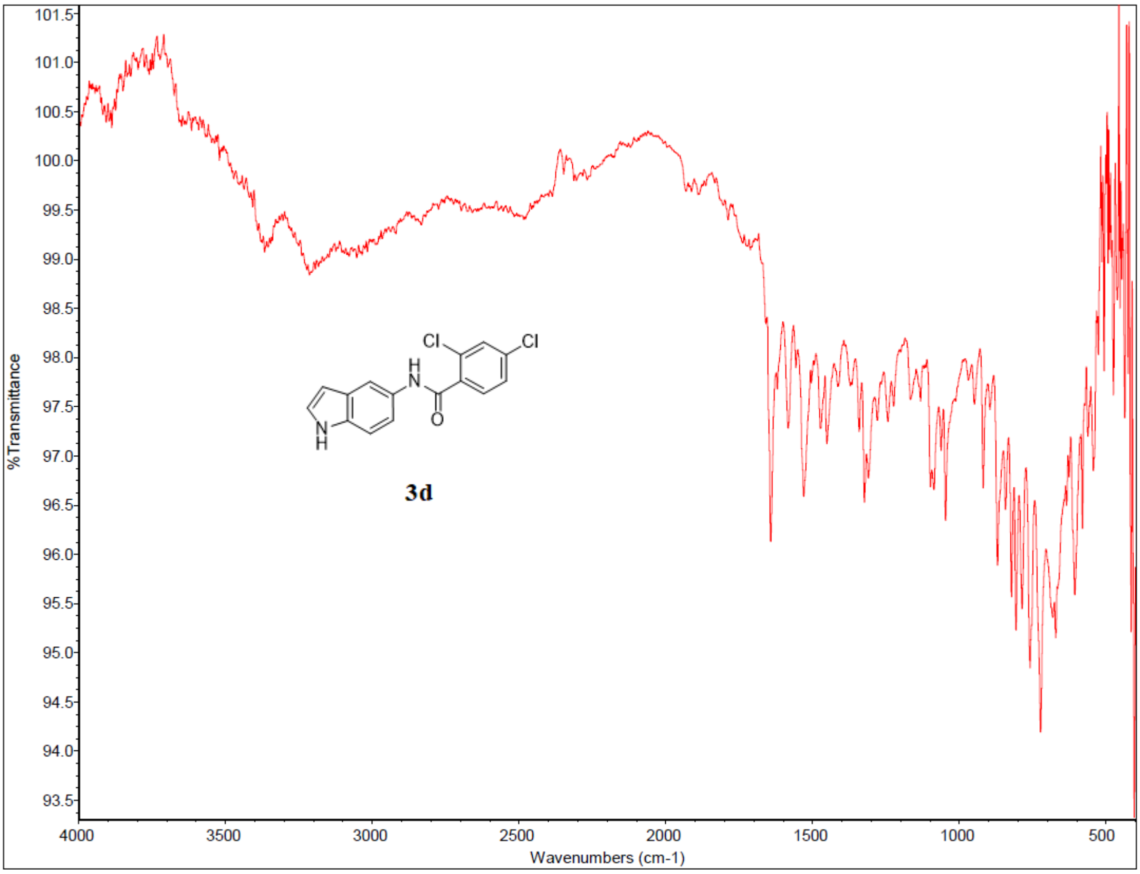

3d
%Transmittance
Wavenumbers (cm-1)

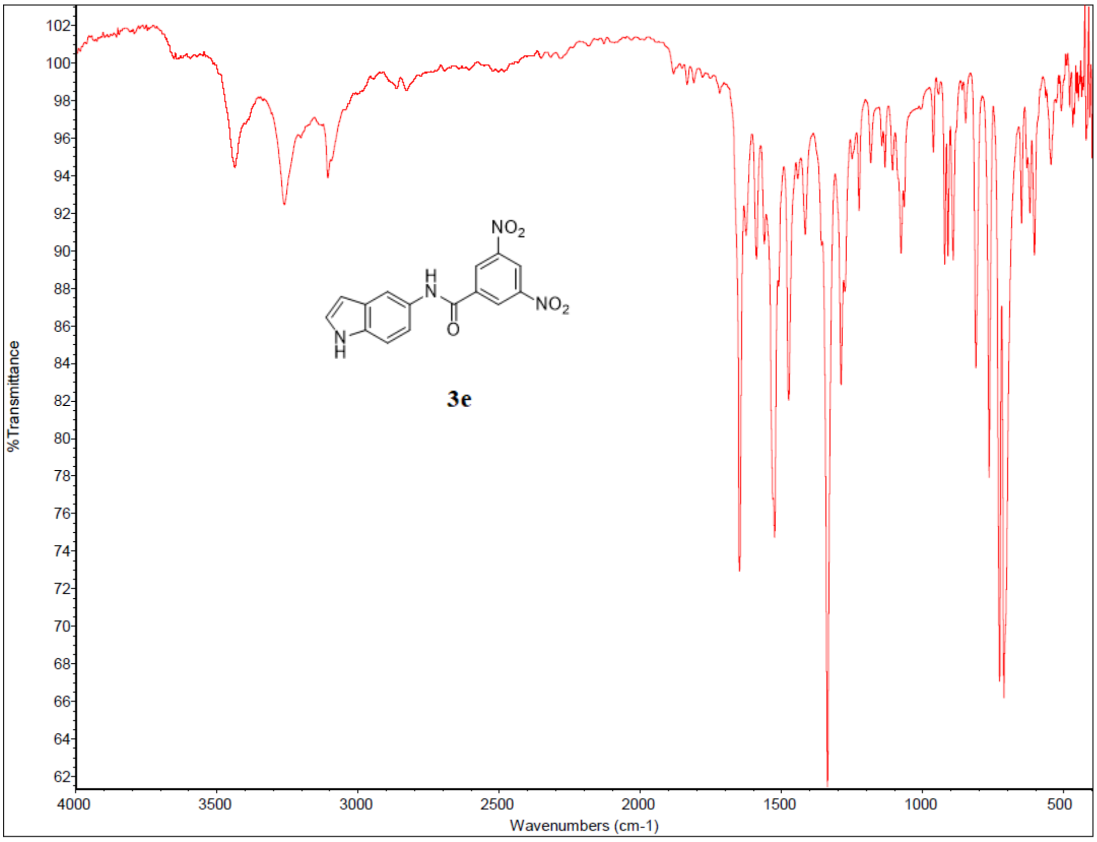

%Transmittance
Wavenumbers (cm-1)
NO_2
NO_2
3e

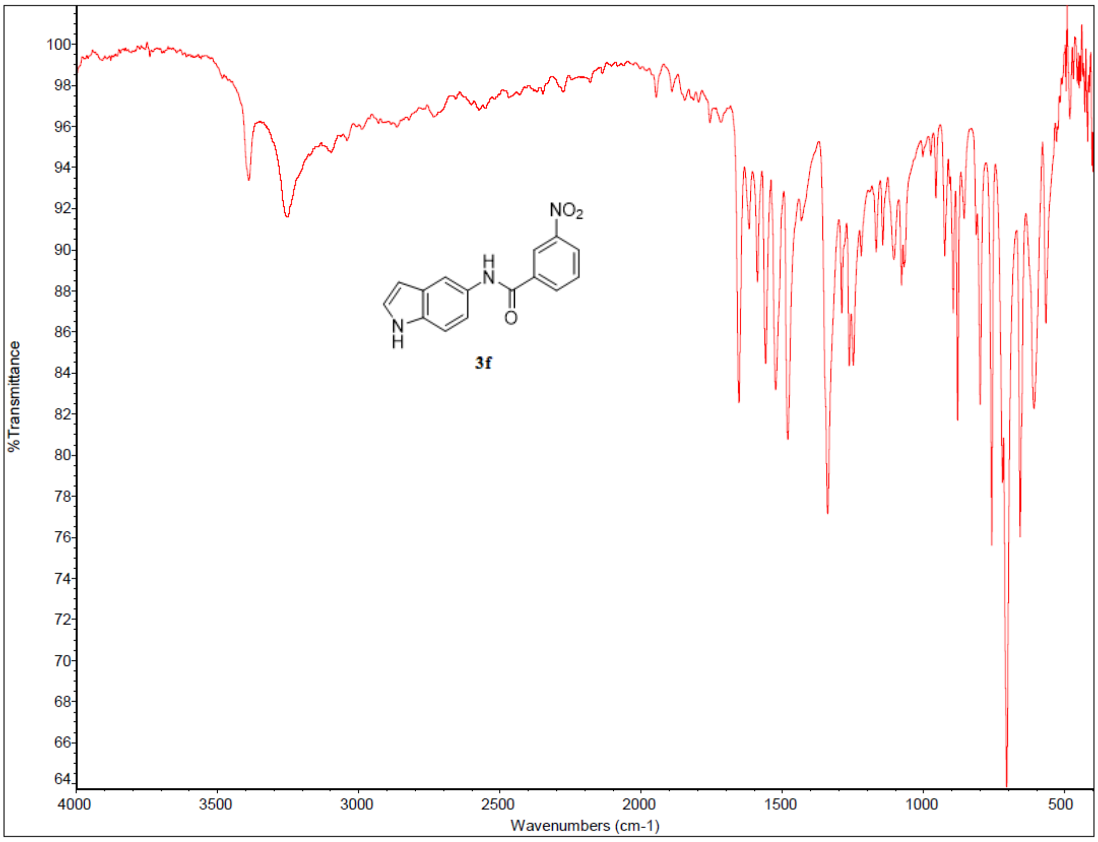

%Transmittance
Wavenumbers (cm-1)
NO_2
3f

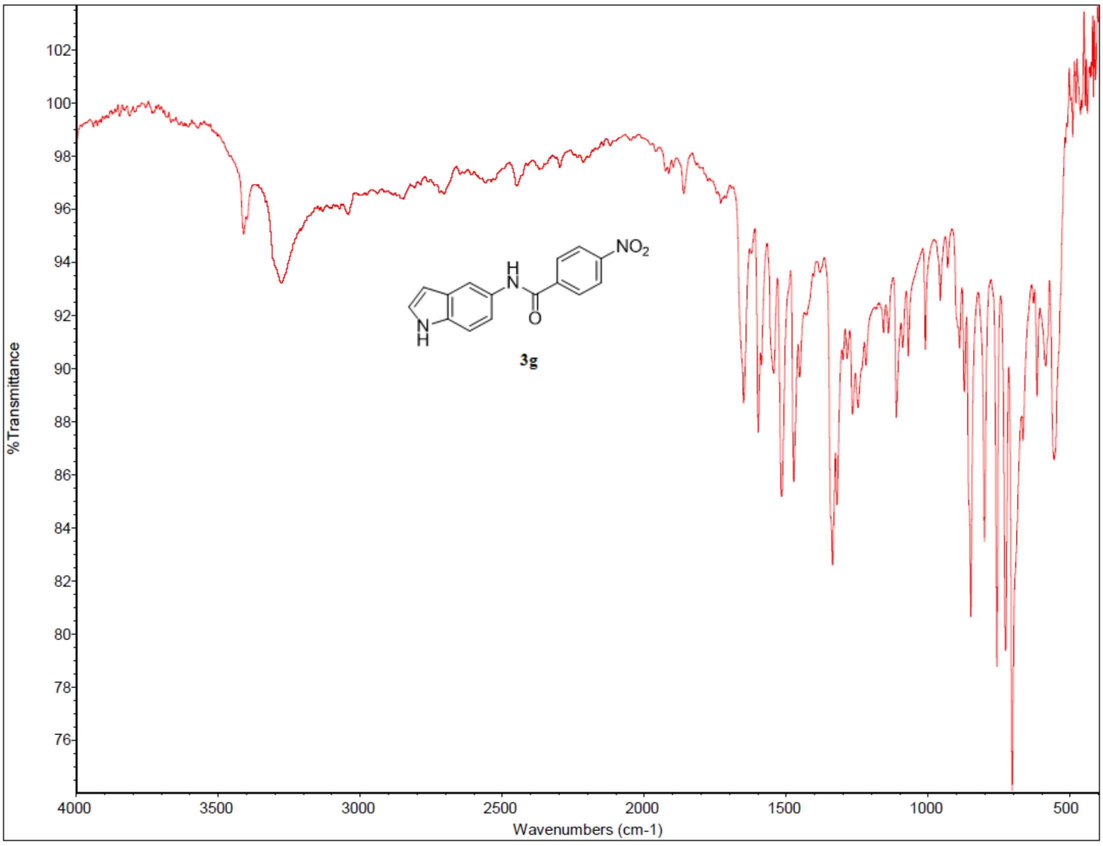
%Transmittance
Wavenumbers (cm-1)
NO2
H
N
O
N
H
3g

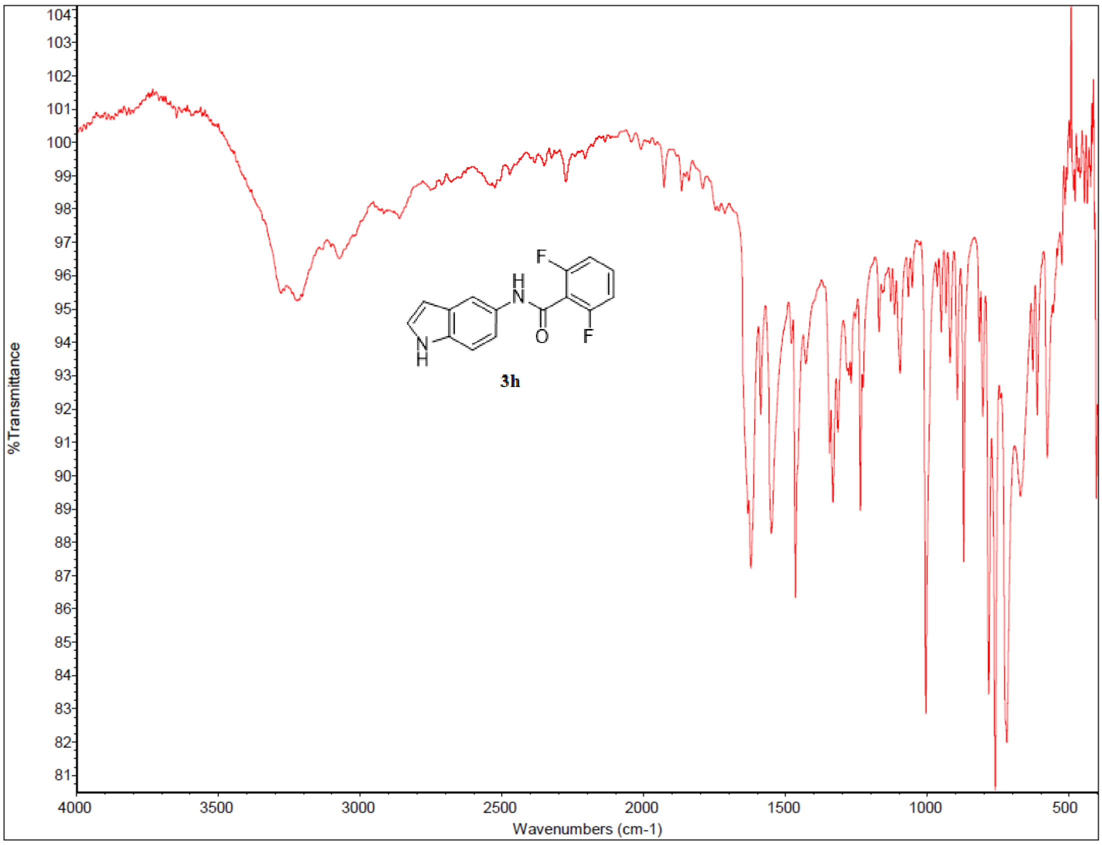
%Transmittance
Wavenumbers (cm-1)
F
H
N
O
F
N
H
3h

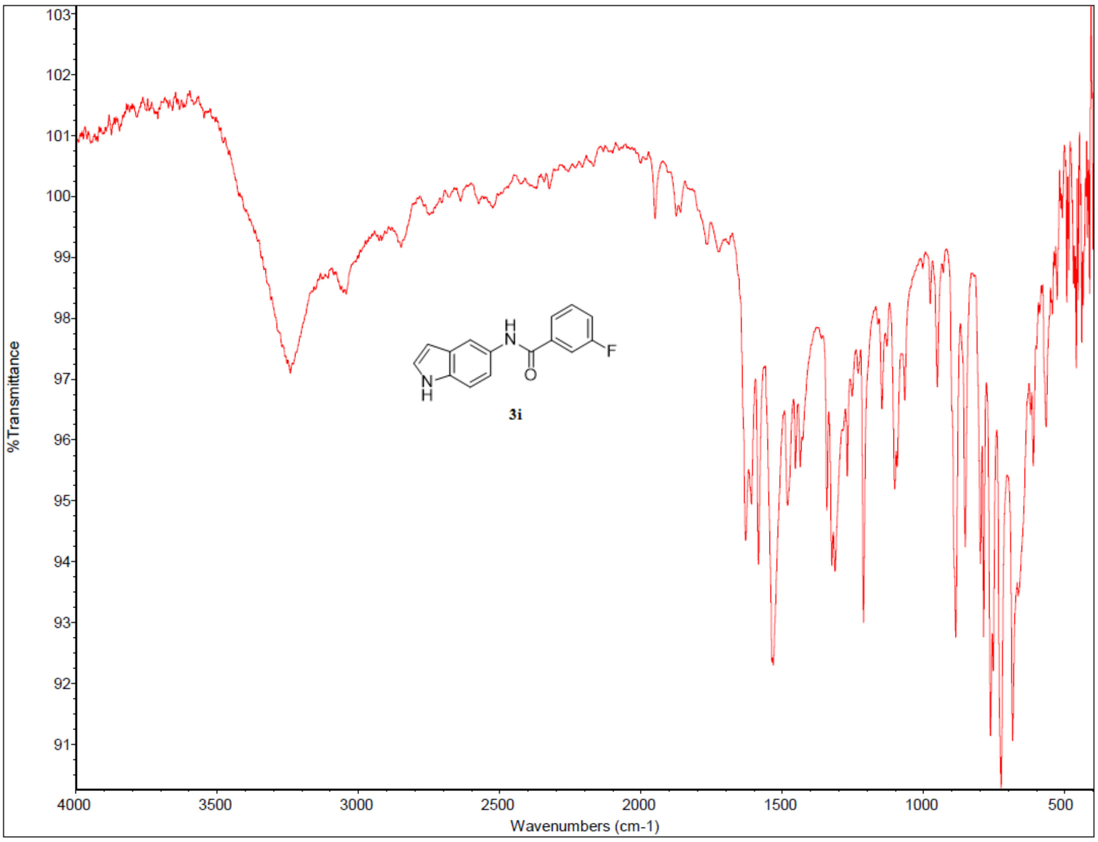

%Transmittance
Wavenumbers (cm-1)
3i

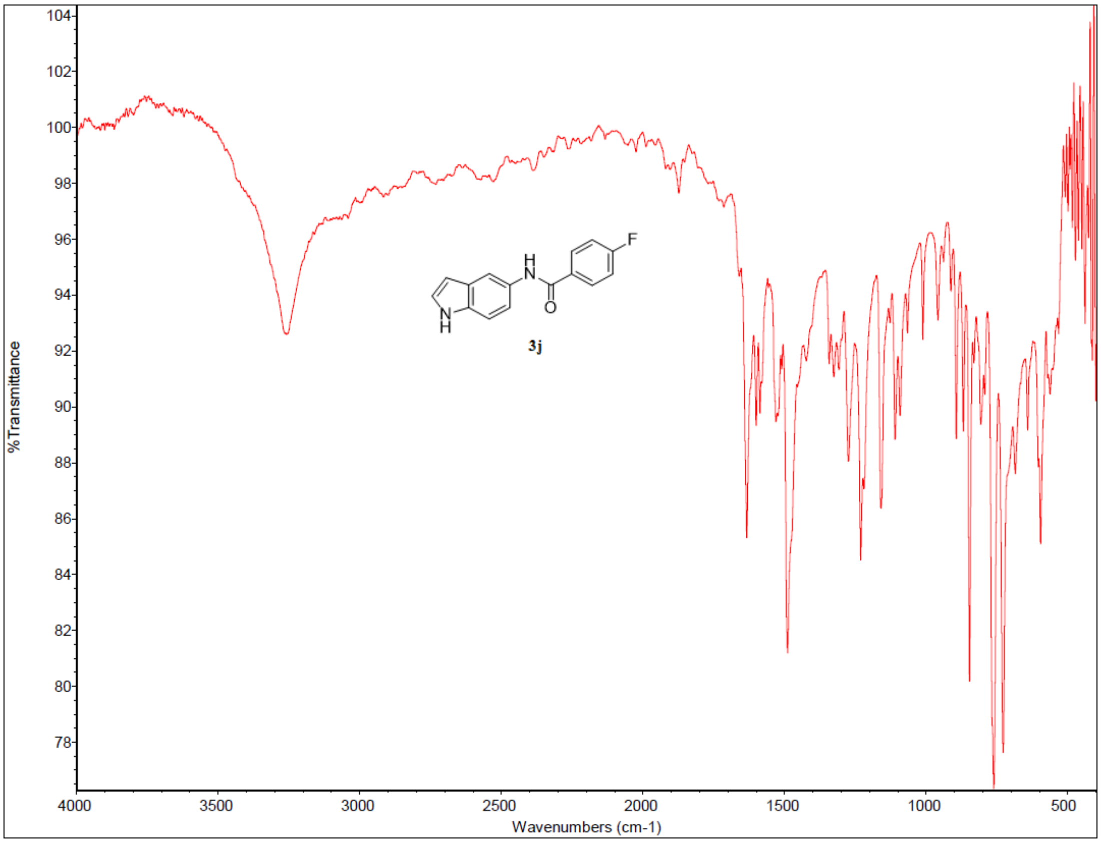

%Transmittance
Wavenumbers (cm-1)
3j

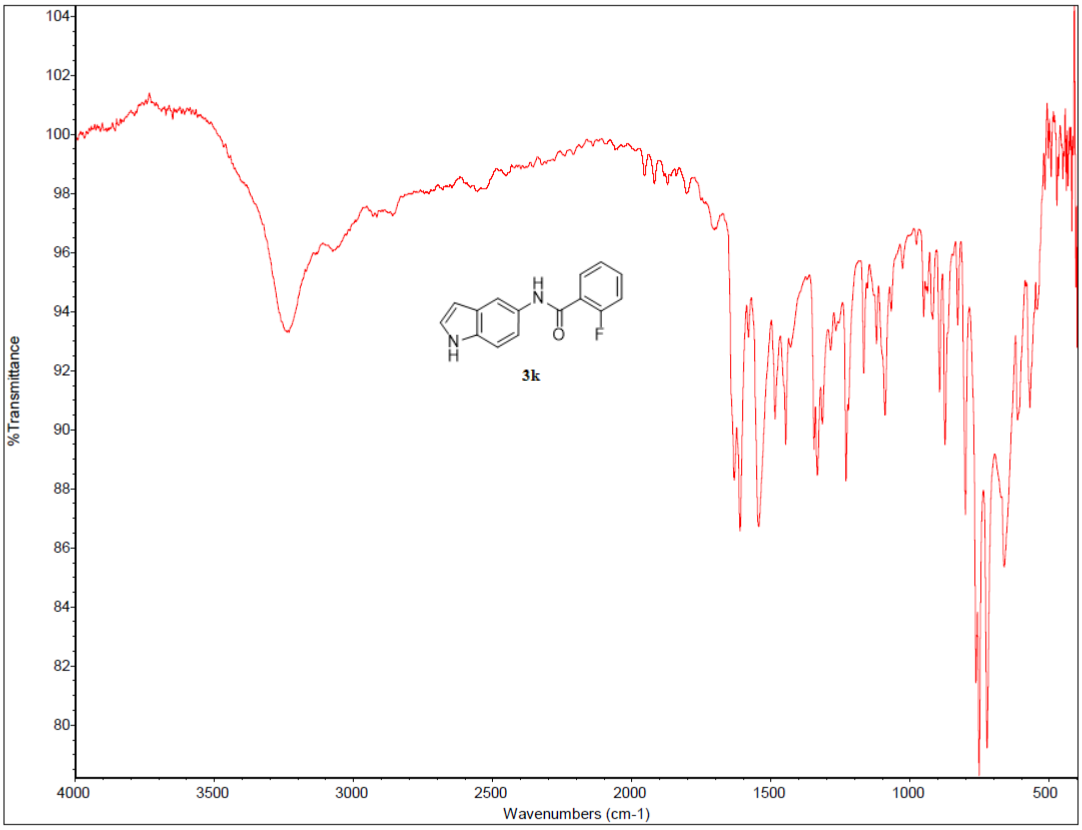

%Transmittance
Wavenumbers (cm-1)

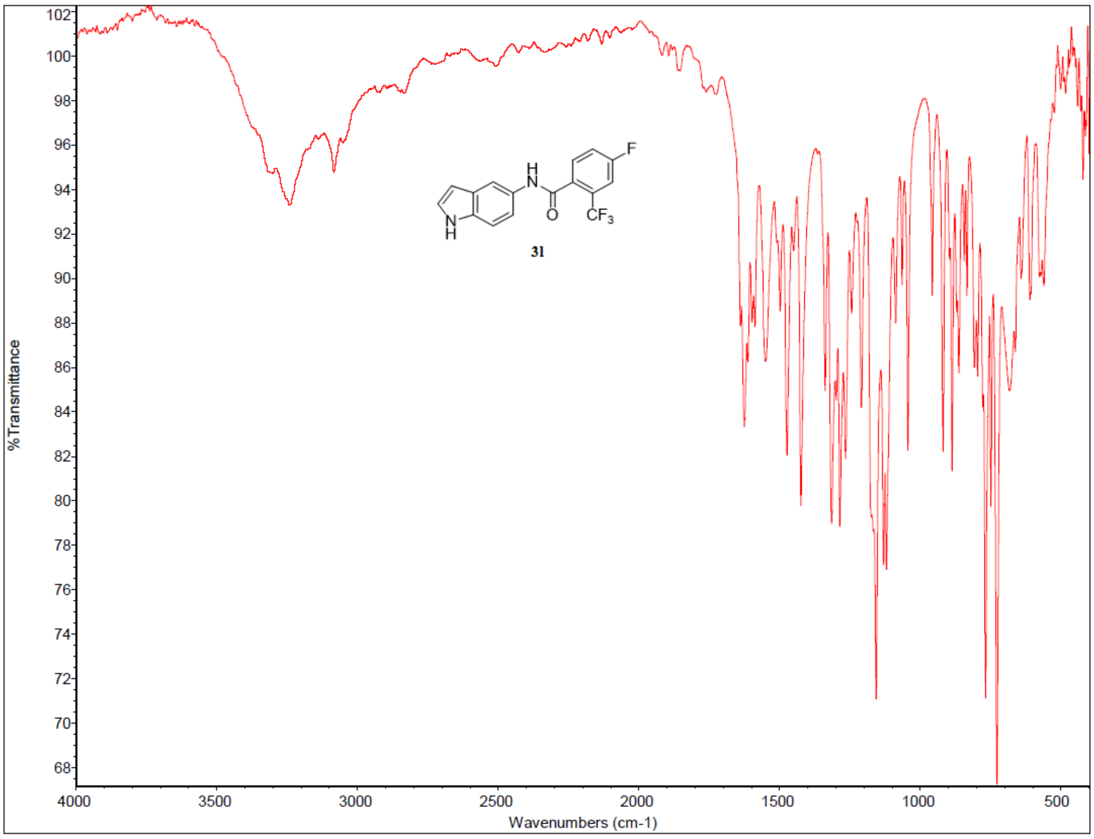

%Transmittance
Wavenumbers (cm-1)

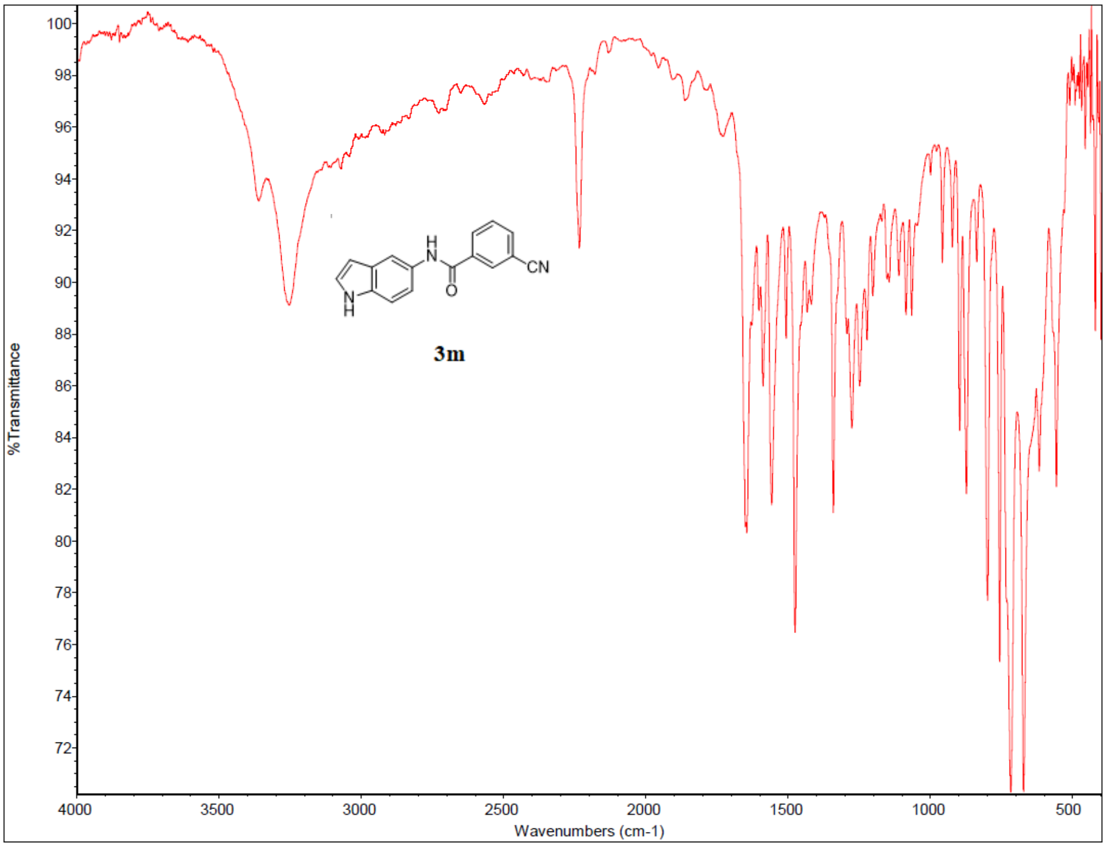

%Transmittance
Wavenumbers (cm-1)
3m
CN

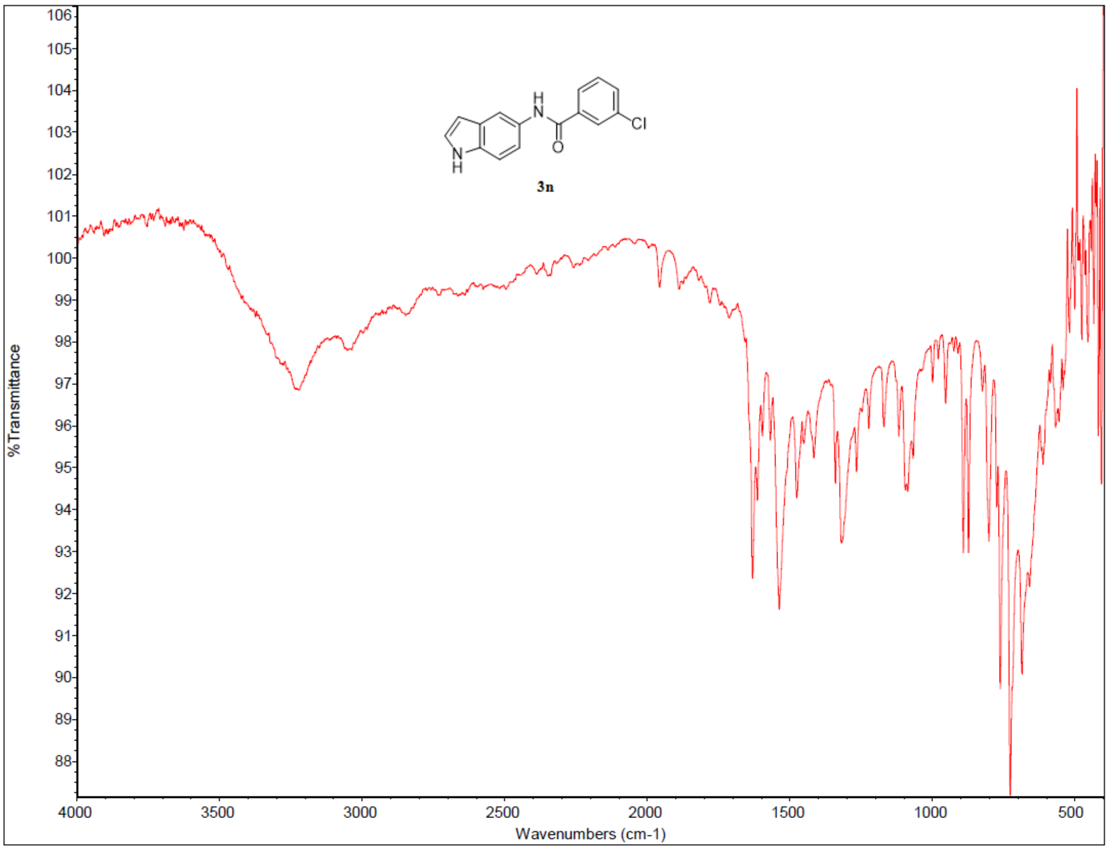

%Transmittance
Wavenumbers (cm-1)
3n
Cl

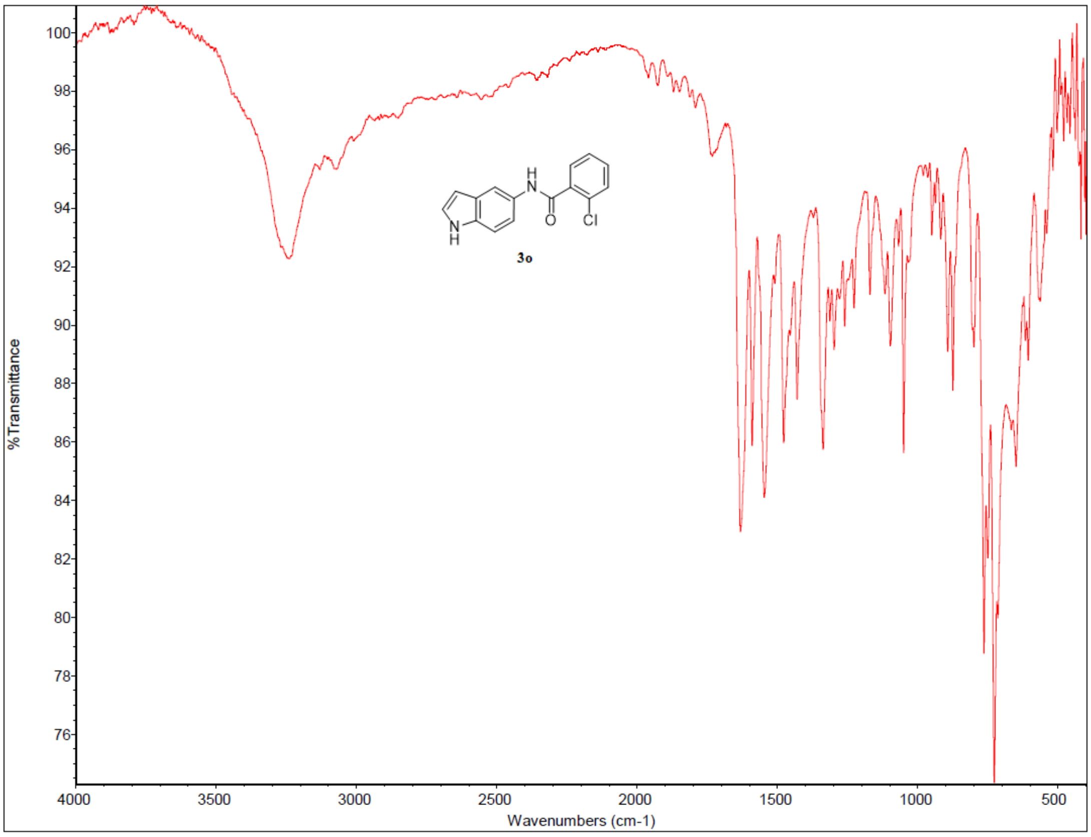

%Transmittance
Wavenumbers (cm-1)
3o

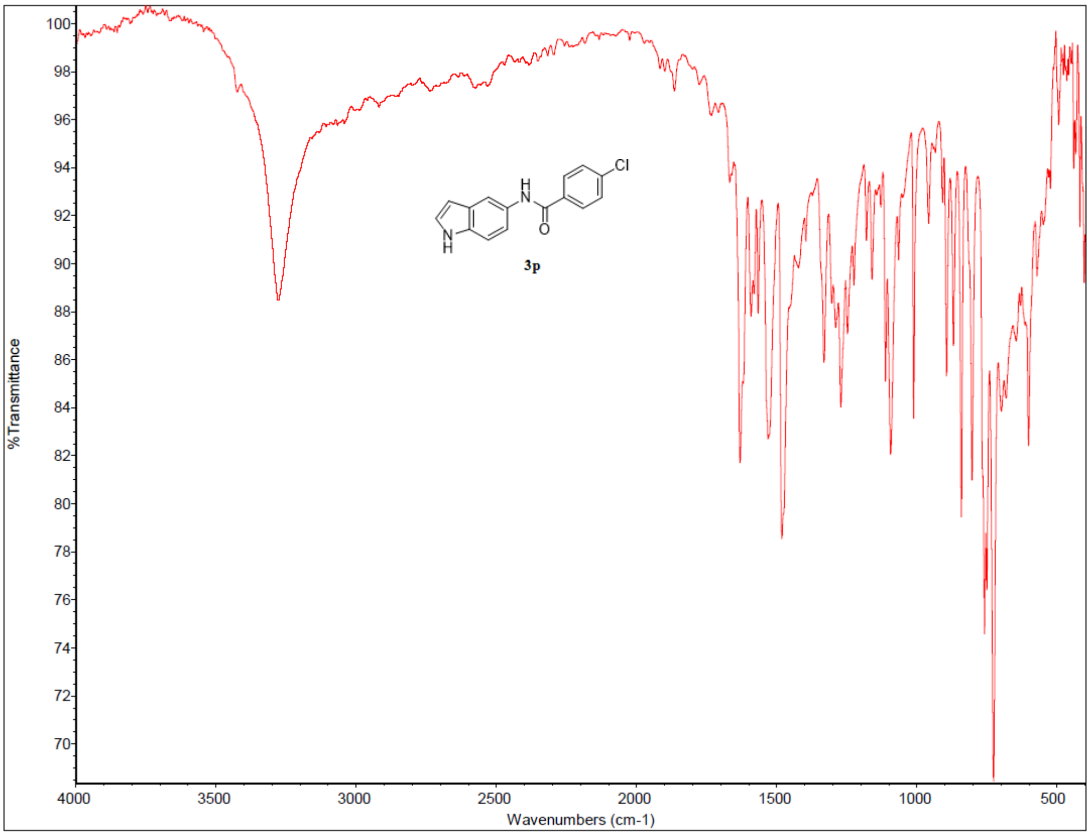

%Transmittance
Wavenumbers (cm-1)
3p

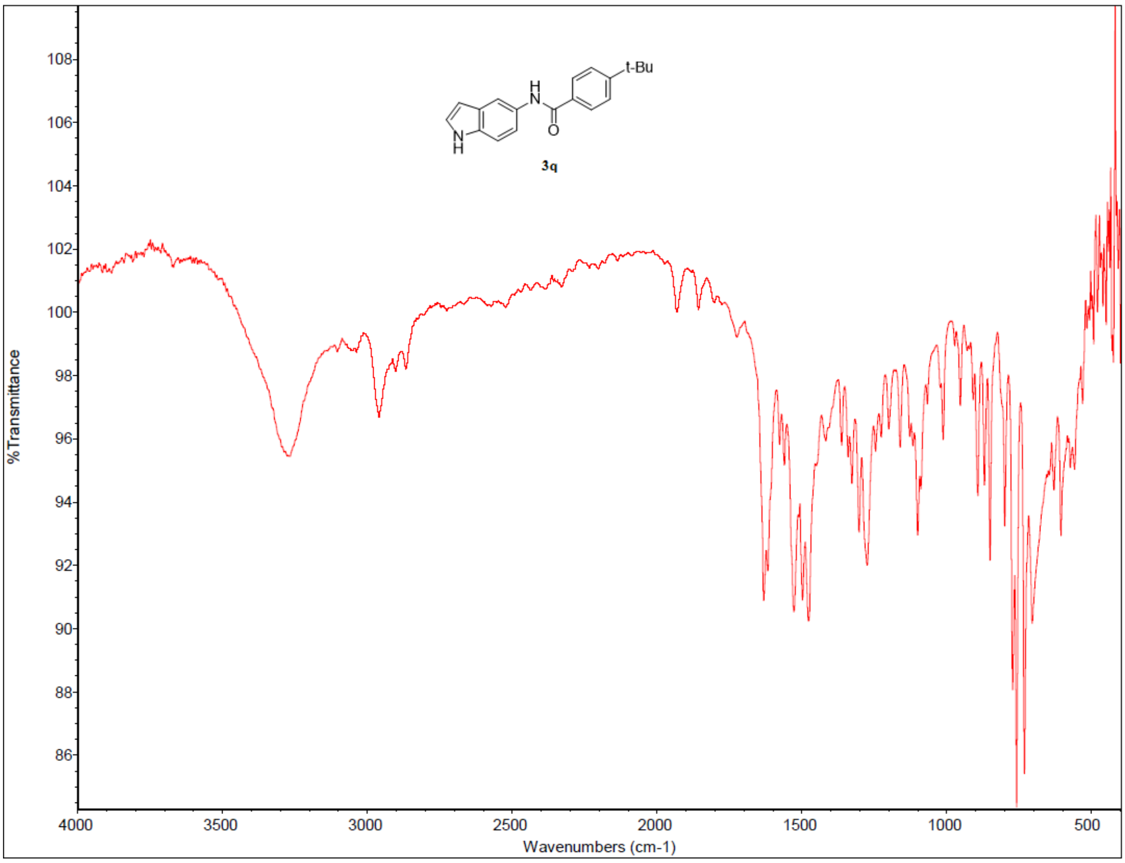

t-Bu
H
N
O
N
H
3q
%Transmittance
108
106
104
102
100
98
96
94
92
90
88
86
4000
3500
3000
2500
2000
1500
1000
500
Wavenumbers (cm-1)

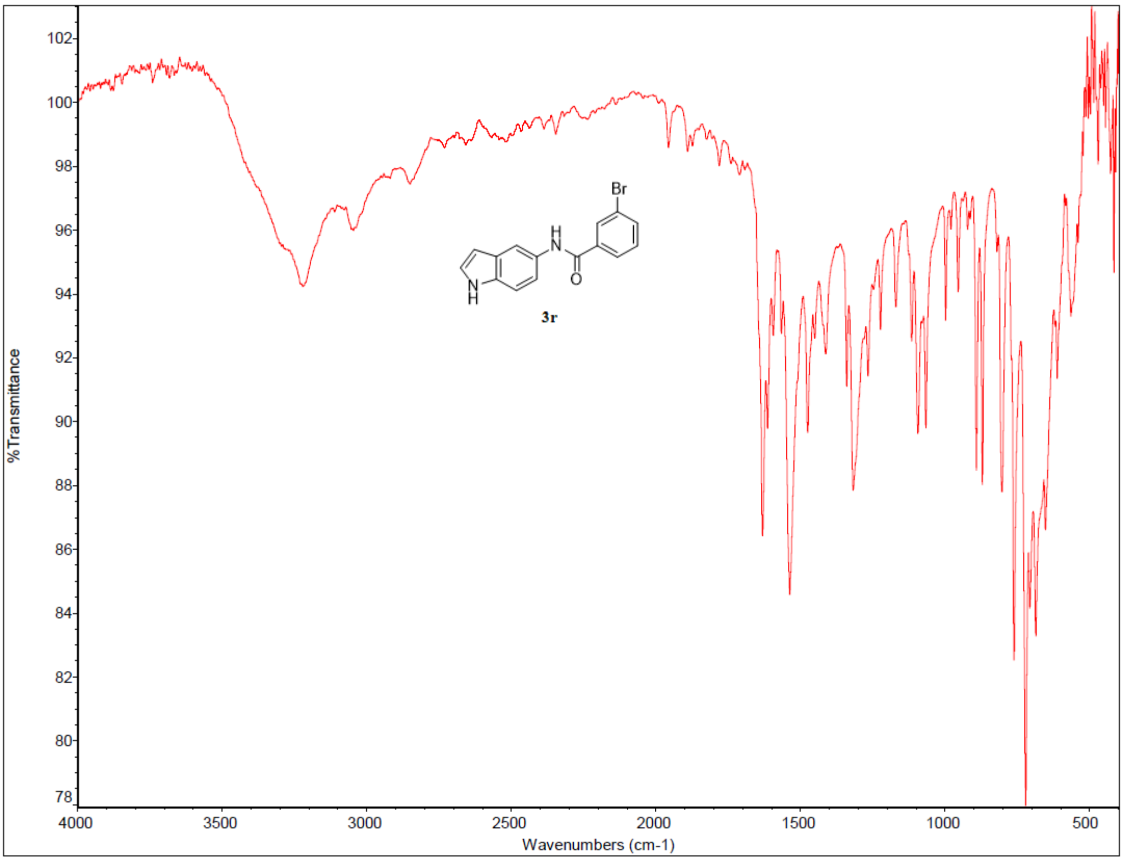

Br
H
N
O
N
H
3r
%Transmittance
102
100
98
96
94
92
90
88
86
84
82
80
78
4000
3500
3000
2500
2000
1500
1000
500
Wavenumbers (cm-1)

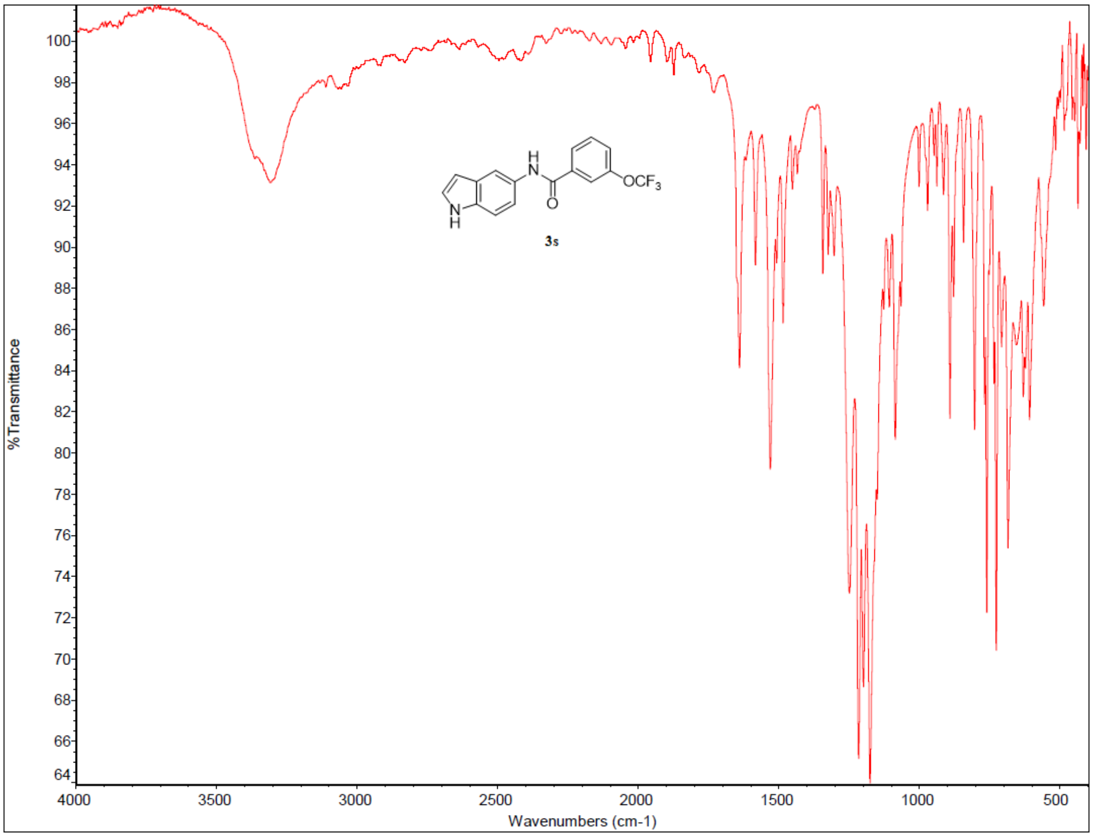

OCF$_3$
H
N
O
N
H
3s
%Transmittance
Wavenumbers (cm-1)

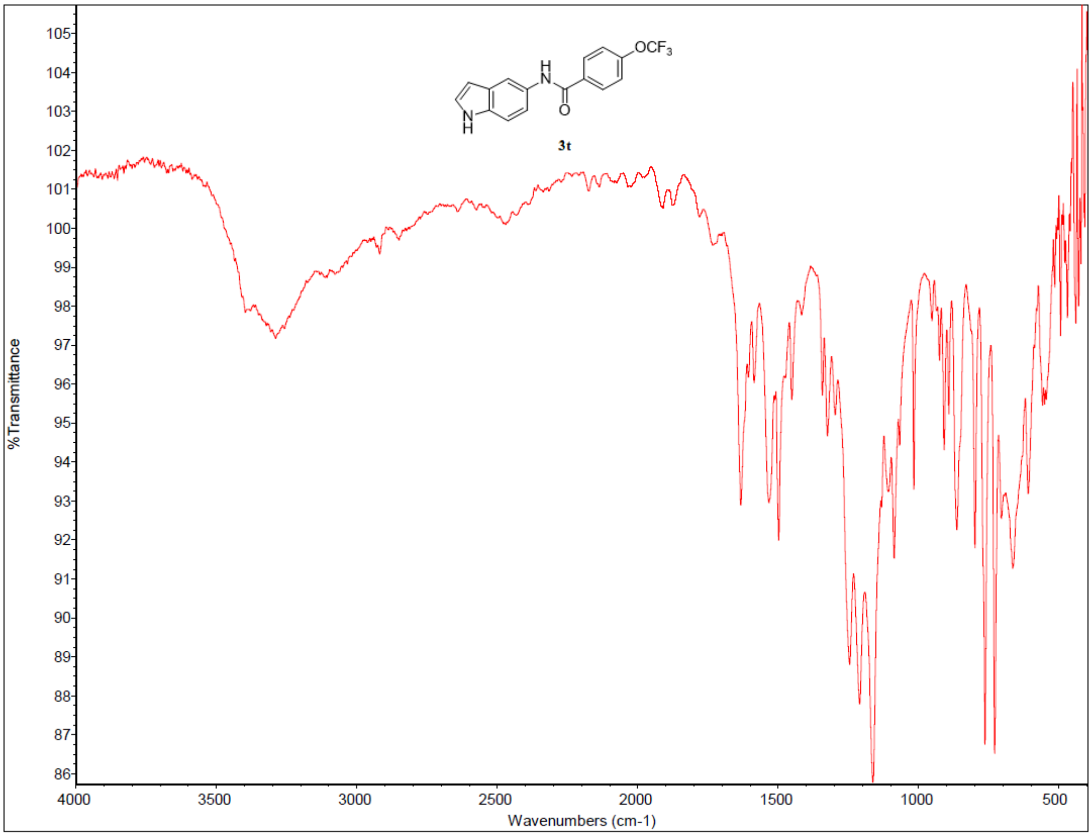

OCF$_3$
H
N
O
N
H
3t
%Transmittance
Wavenumbers (cm-1)

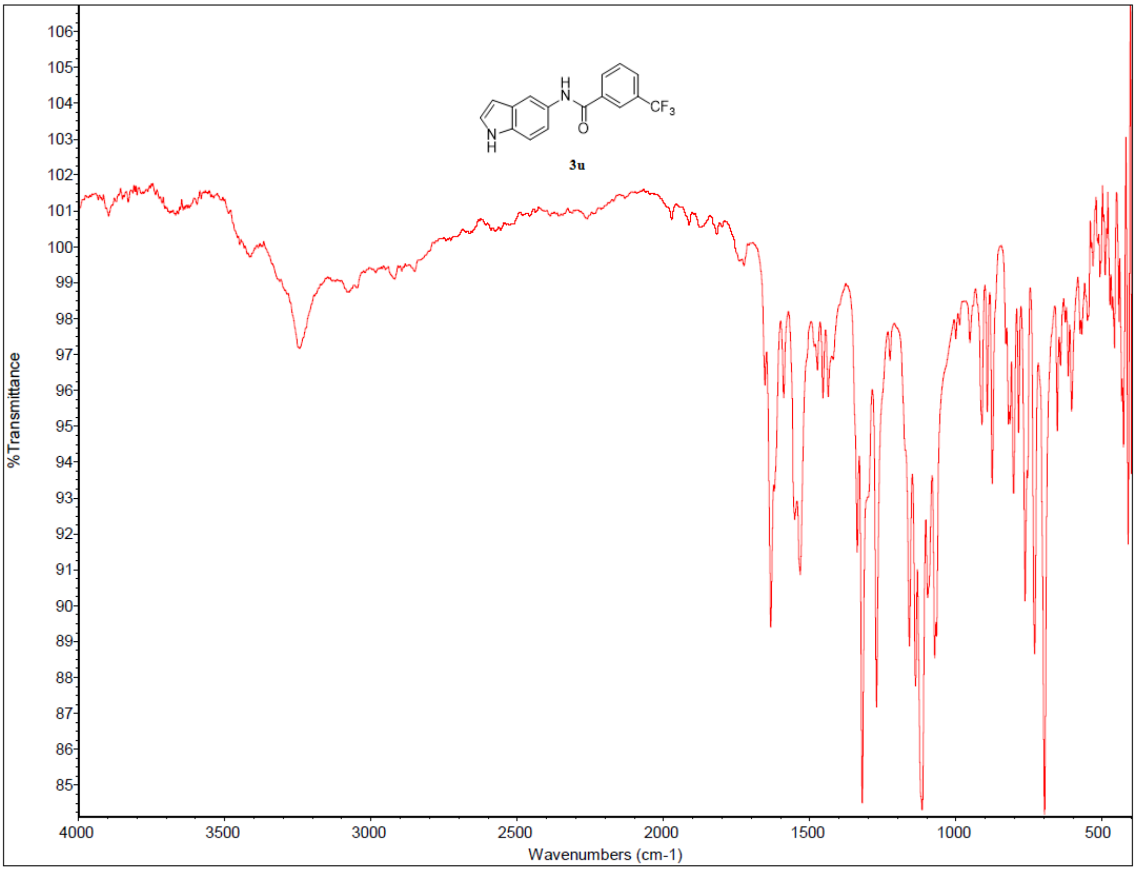
3u
%Transmittance
Wavenumbers (cm-1)

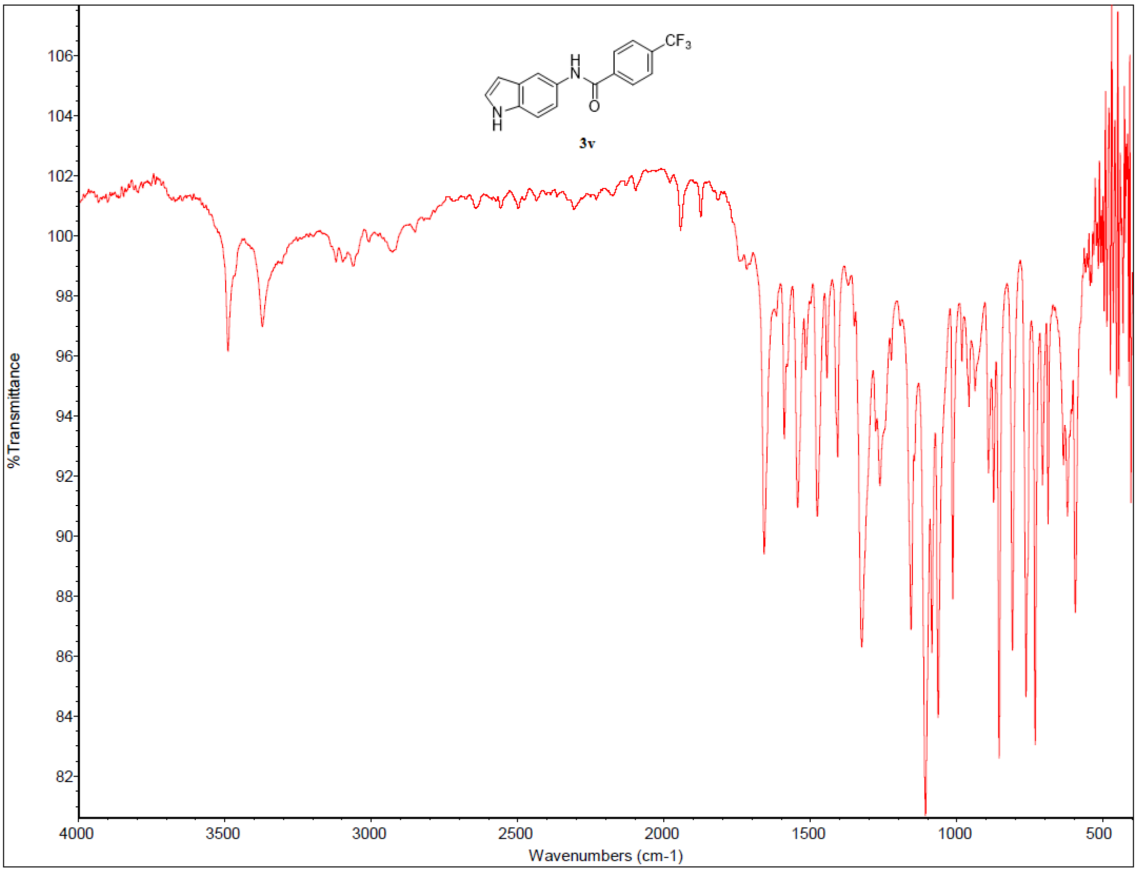
3v
%Transmittance
Wavenumbers (cm-1)

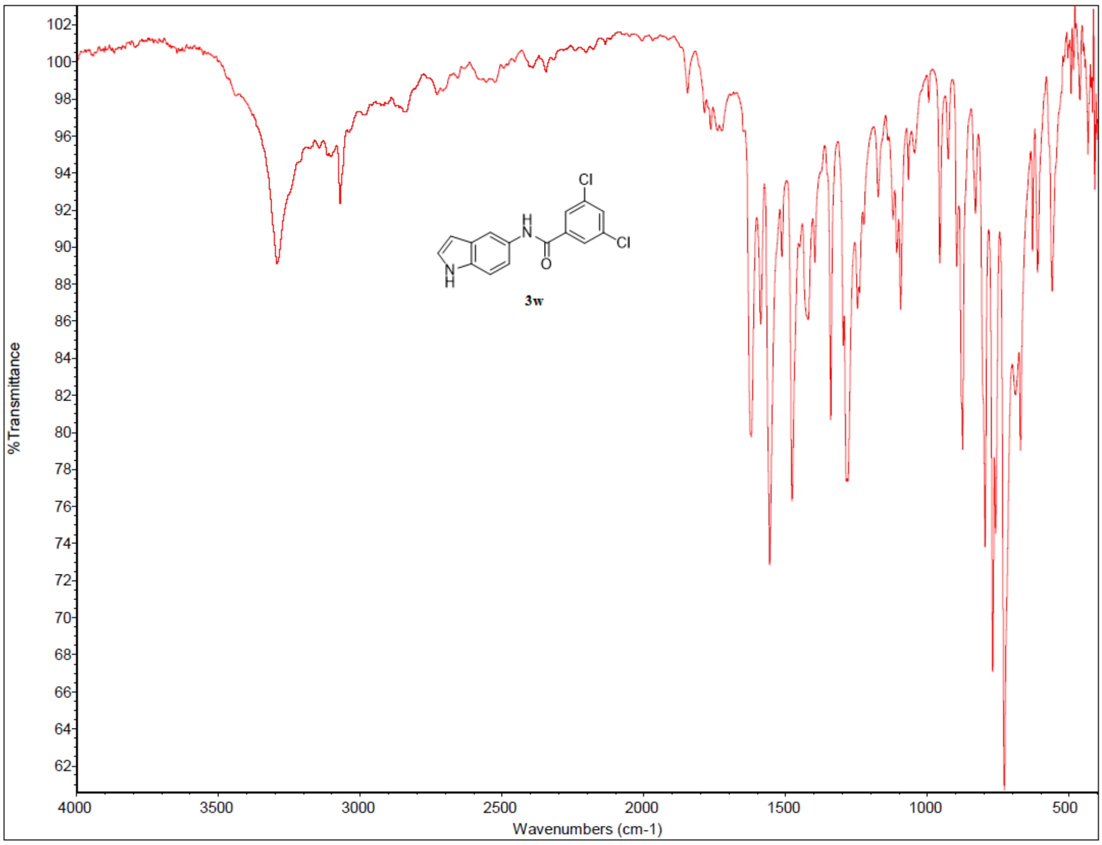
Cl
H
N
O
Cl
N
H
%Transmittance
Wavenumbers (cm-1)
4000
3500
3000
2500
2000
1500
1000
500

3w

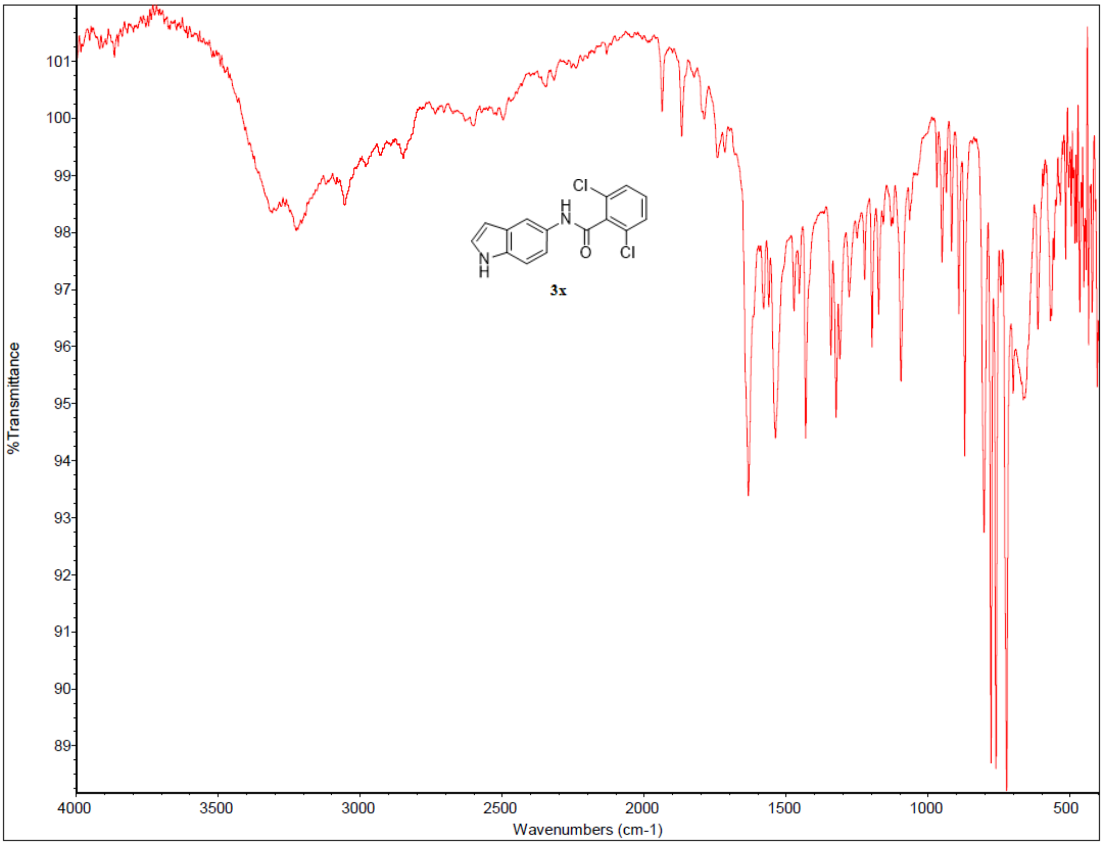
Cl
H
N
O
Cl
N
H
%Transmittance
Wavenumbers (cm-1)
4000
3500
3000
2500
2000
1500
1000
500

3x

6. Materials and methods of monoamine oxidase (MAO) enzyme assay

The inhibitory activity of MAO-A, B enzyme of the compounds was evaluated based on previously described method [1]. The MAO activity was measured in relative fluorescence units (RFU) evoked by peroxidase-catalyzed oxidation of Amplex Red® to resorufin, in which H_2O_2 generated by MAO reaction was used as the electron donor. Human recombinant MAO-A (*h*MAO-A) and MAO-B (*h*MAO-B) enzyme expressed in insect cells were obtained from Sigma Aldrich. The 2 μL of test compound in DMSO (final concentration: 1 nM – 10 μM) was treated with 98 μL of *h*MAO enzyme solution in 50 mM sodium phosphate buffer (pH 7.4, final protein amounts: ~1.25 μg protein/well for MAO-A and ~2.5 μg protein/well for MAO-B) on 96-well black plate and incubated for 15 min at 37 °C. Then 100 μL of reaction working solution which is mixed solution of 400 μM Amplex Red® (Cayman, final concentration: 200 μM), 2 U/mL horseradish peroxidase (Sigma-Aldrich, final concentration: 1 U/mL) and 2 mM substrate (*p*-tyramine for MAO-A, benzylamine for MAO-B, Sigma-Aldrich, final concentration: 1 mM) in 50 mM sodium phosphate buffer (pH 7.4) were added and incubated for 20 min at 37 °C in the dark. The fluorescent intensity was quantified using a microplate reader (SpectraMax®*i*3, Molecular Device) with an excitation at 545 nm and an emission at 590 nm. The 50% inhibitory concentrations (IC_{50}) of compounds were determined as the mean ± S.E.M. in triplicate from the dose-response inhibition curves using SigmaPlot® 13.0.

References

1. Choi, J.W.; Jang, B.K.; Cho, N.C.; Park, J.H.; Yeon, S.K.; Ju, E.J.; Lee, Y.S.; Han, G.; Pae, A.N.; Kim, D.J., et al. Synthesis of a series of unsaturated ketone derivatives as selective and reversible monoamine oxidase inhibitors. *Bioorganic & medicinal chemistry* **2015**, *23*, 6486-6496, doi:10.1016/j.bmc.2015.08.012.
